# Neem leaf powder (*Azadirachta indica*) mitigates oxidative stress and pathological alterations triggered by lead toxicity in Nile tilapia (*Oreochromis niloticus*)

**DOI:** 10.1038/s41598-023-36121-4

**Published:** 2023-06-06

**Authors:** Nermeen M. Abu-Elala, Marwa S. Khattab, Huda O. AbuBakr, Samah Helmy, Ahmed Hesham, Nehal A. Younis, Mahmoud A. O. Dawood, Mohammed F. El Basuini

**Affiliations:** 1 Department of Aquatic Animal Medicine, Faculty of Veterinary Medicine, Cairo University, Giza, Egypt; 2 Faculty of Veterinary Medicine, King Salman International University, South Sinai, Egypt; 3 Department of Pathology, Faculty of Veterinary Medicine, Cairo University, Giza, 12211 Egypt; 4 Department of Biochemistry and Molecular Biology, Faculty of Veterinary Medicine, Cairo University, Giza, 12211 Egypt; 5 Department of Immunology, Animal Health Research Institute, Dokki, Giza, Egypt; 6 Department of Chemistry, Faculty of Science, Suez Canal University, Ismailia, 41522 Egypt; 7 Middle East for Veterinary Vaccine (MEVAC), El-Salihya El-Gededa, 44671 El-Sharkia Egypt; 8 Department of Animal Production, Faculty of Agriculture, Kafrelsheikh University, Kafr El-Sheikh, 33516 Egypt; 9 The Center for Applied Research on the Environment and Sustainability, The American University in Cairo, Cairo, 11835 Egypt; 10 Faculty of Agriculture, Tanta University, Tanta, 31527 Egypt; 11 Faculty of Desert Agriculture, King Salman International University, South Sinai, 46618 Egypt

**Keywords:** Ichthyology, Freshwater ecology, Animal physiology

## Abstract

This study investigated the clinical and pathological symptoms of waterborne lead toxicity in wild Nile tilapia collected from a lead-contaminated area (the Mariotteya Canal: Pb = 0.6 ± 0.21 mg L^−1^) and a farmed fish after 2 weeks of experimental exposure to lead acetate (5–10 mg L^−1^) in addition to evaluating the efficacy of neem leaf powder (NLP) treatment in mitigating symptoms of lead toxicity. A total of 150 fish (20 ± 2 g) were alienated into five groups (30 fish/group with three replicates). G1 was assigned as a negative control without any treatments. Groups (2–5) were exposed to lead acetate for 2 weeks at a concentration of 5 mg L^−1^ (G2 and G3) or 10 mg L^−1^ (G4 and G5). During the lead exposure period, all groups were reared under the same conditions, while G3 and G5 were treated with 1 g L^−1^ NLP. Lead toxicity induced DNA fragmentation and lipid peroxidation and decreased the level of glutathione and expression of heme synthesis enzyme delta aminolaevulinic acid dehydratase (ALA-D) in wild tilapia, G2, and G4. NLP could alleviate the oxidative stress stimulated by lead in G3 and showed an insignificant effect in G5. The pathological findings, including epithelial hyperplasia in the gills, edema in the gills and muscles, degeneration and necrosis in the liver and muscle, and leukocytic infiltration in all organs, were directly correlated with lead concentration. Thus, the aqueous application of NLP at 1 g L^−1^ reduced oxidative stress and lowered the pathological alterations induced by lead toxicity.

## Introduction

Aquaculture is thought to be a practical way to replace and conserve overfished stocks and endangered fish species, as well as to bridge the gap between production and human demand^1–3^. Aquaculture has significantly increased the amount of seafood produced since the 1970s, yet there are still several challenges for the industry. A variety of interrelated factors, such as the aquatic environment, nutrition, and the farmed stock, influence how effectively aquaculture operations operate. Sustainable aquaculture is built on maximizing these variables^4^. The employment of sustainable and eco-friendly techniques to boost aquaculture's efficacy and mitigate environmental stressors has become of interest recently^5^.

For a very long period, the best methods to increase growth, development, immunity, and treat infections were chemotherapy and antibiotics. However, the continued use of conventional chemotherapy in aquaculture was constrained by a number of negative consequences on the fish's natural immunity and ecology^6,7^. Eco-friendly approaches have been made available for the aquaculture sector as an alternative^8–11^. Exogenous enzymes, beneficial microorganisms, and medicinal plants are the ideal tactics for aquatic organisms' health and production^12–15^. The aquatic milieu is a sump for many environmental contaminants^16–18^. Lead is a non-fundamental element that enters the aquatic ecosystem from various sources, such as mining and industrial processes^19,20^. Lead is a redox inactive metal that can accumulate in the tissues and organs of aquatic organisms and can persist in water and sediments for a long time^21–23^. Oxidative stress is the central mechanism of lead-stimulated toxicity. Increasing reactive oxygen species (ROS) generation beyond the ability of the antioxidant system origins lipid peroxidation in the cell membranes of various organs, protein and DNA oxidation, enzyme deactivation, alterations in gene expression, and alterations in cellular redox status^24,25^. Structures of the antioxidant system in fish comprise enzymes and low-molecular-weight antioxidants^26^. Superoxide dismutase (SOD), catalase (CAT), glutathione peroxidase (GPx), and glutathione-s-transferase (GST) are the primary antioxidant enzymes and serve as crucial markers of oxidative stress^2,4,27^. In addition, reductions in glutathione (GSH) and oxidized glutathione disulfide (GSSG) perform a crucial function in non-enzymatic antioxidant defense^28^. Lead modifies the hematopoietic system by inhibiting hemoglobin synthesis and restricting essential enzymes in the heme synthesis pathway. It also lowers the lifespan of circulating erythrocytes by boosting the fragility of cell membranes^29^. Lead downregulates three key enzymes necessary for synthesizing heme, the most prominent of which is delta aminolaevulinic acid dehydratase (ALA-D), also identified as porphobilinogen synthase. ALA-D is a cytosolic enzyme that catalyzes the second phase of heme synthesis by forming porphobilinogen from delta-aminolevulinic acid (ALA)^30,31^. Although ALA-D is expressed in all tissues, erythrocytes and the liver have the highest expression levels^32,33^. The downregulation or inactivation of the ALA-D enzyme is employed clinically to measure the level of lead toxicity^29,34,35^. Water pollution results in various pathological changes in fish tissue, the severity of which can be associated with the degree of water pollution^36,37^. The two organs most affected are the gills, which come into straight contact with water pollutants, and the liver, which is involved in detoxification. Heavy metal bioaccumulation can also affect other organs^38–40^.

Nile tilapia (*Oreochromis niloticus*) is Egypt's most widely consumed farmed freshwater fish^10,40,41^. The Mariotteya Canal is a heavy metal-contaminated body of water^42^. Tilapia cultured at this contaminated site is considered a suitable bioindicator of metal contamination and present a potential risk to human well-being^21–23^. An efficient lowering of lead at least below the regulatory level in domestic and industrial wastewater is significant. The adsorption of heavy metals by agricultural materials has been widely explored^43^. Neem (*Azadirachta indica*) is a promising therapeutic plant that manages fish predators and treats many bacterial and parasitic fish diseases. It has an adsorbent capacity for various metals such as lead and cadmium^44–46^. The raw plant materials (leaves) are harvested and applied immediately or after drying and grinding. A simple way to prepare aqueous neem extract is to soak plant material in water. The raw neem leaf powder (NLP) and its aqueous extract exhibit more benefits on animals and fish rather than harmful effects^47^. Bhattacharyya and Sharma^44^ found that 1.2 g L^−1^ NLP could eliminate as much as 93% of lead in 96 h from a solution of 300 mg L^−1^ using batch adsorption technology.

This study explored the influence of lead on fish health status through the estimation of lead bioaccumulation in various tissues and assessment of oxidative stress in the liver, gills, and muscles of *O. niloticus*, which reflects the metal contamination of the aquatic environment. We also conducted a histopathological assessment of fish organs collected from a naturally contaminated site and with experimental lead-induced toxicity, explicitly referring to the potential role of aqueous exposure of neem leaf powder in lowering lead toxicity.

## Material and methods

### Wild fish and water sampling

Surface water and wild Nile tilapia samples were assembled from the Mariotteya Canal in Giza, Egypt, which suffers from severe biological and chemical pollution problems owing to a massive input of untreated domestic, agricultural, and industrial wastes^42^. Three water samples were collected in clean glass bottles (1 L volume) 30 cm below the water’s surface. A total of ten wild Nile tilapia at a mean body weight of 200 ± 30 g were collected from the same area and transported in an ice box to the laboratory. Fish were dissected to obtain liver, gill, and muscle samples. Lead concentration analysis was performed on the water and part of the tissue samples on the sampling day. Parts of the tissues were kept at − 80 °C to evaluate oxidant/antioxidant biomarkers, genotoxicity, and gene expression; another was used for histopathological evaluation.

### Analysis of lead concentration in water and fish samples

Water samples were mixed with nitric acid, purified through a glass filter, and analyzed using an atomic spectrophotometer (Model 3100, Perkin-Elma, Norwalk, CT, USA). Tissue samples were dehydrated at 105 °C for 12 h, burned at 550 °C for 16 h in a muffle furnace, acid-digested (HNO_3_), and diluted with deionized water to an established volume using the dry-ash procedure recommended by Omar et al.^48^. Lead concentration in fish tissues was stated in mg kg^−1^.

### Preparation of neem as a bio-sorbent material and GC–MS analysis

Mature *A. indica* leaves were collected from Agriculture Research Center, Egypt. Leaves were thoroughly washed to eliminate dust and impurities and then dried at room heat and in an oven at 50 °C for 3 days. The dried leaves were crushed and sieved to collect the fine neem leaf powder (NLP). The powder was washed with purified water to remove the pigment and turbidity, re-dried, and stored in a glass jar as a bio-sorbent^46^. The NLP was analyzed using a direct capillary column TG–5MS and a GC-TSQ mass spectrometer (Thermo Fisher Scientific, Austin, TX, USA), and the chemical composition of the ethanolic neem extract was determined. The temperature of the column oven was initially held at 60 °C, expanded to 250 °C at 6 °C min^−1^, held for 1 min, and then elevated to 300 °C at 30 °C min^−1^. The injector temperature was maintained at 270 °C. Pure helium was utilized as a carrier gas at a steady flow rate of 1 mL min^−1^. Using an Autosampler AS3000 and GC in split mode, diluted samples of 1 µl were automatically injected with a solvent delay of 4 min. In full scan mode, EI mass spectra covering the *m/z* range of 50–650 were gathered at ionization voltages of 70 eV.

### Fish and experimental design

**Figure 1:**
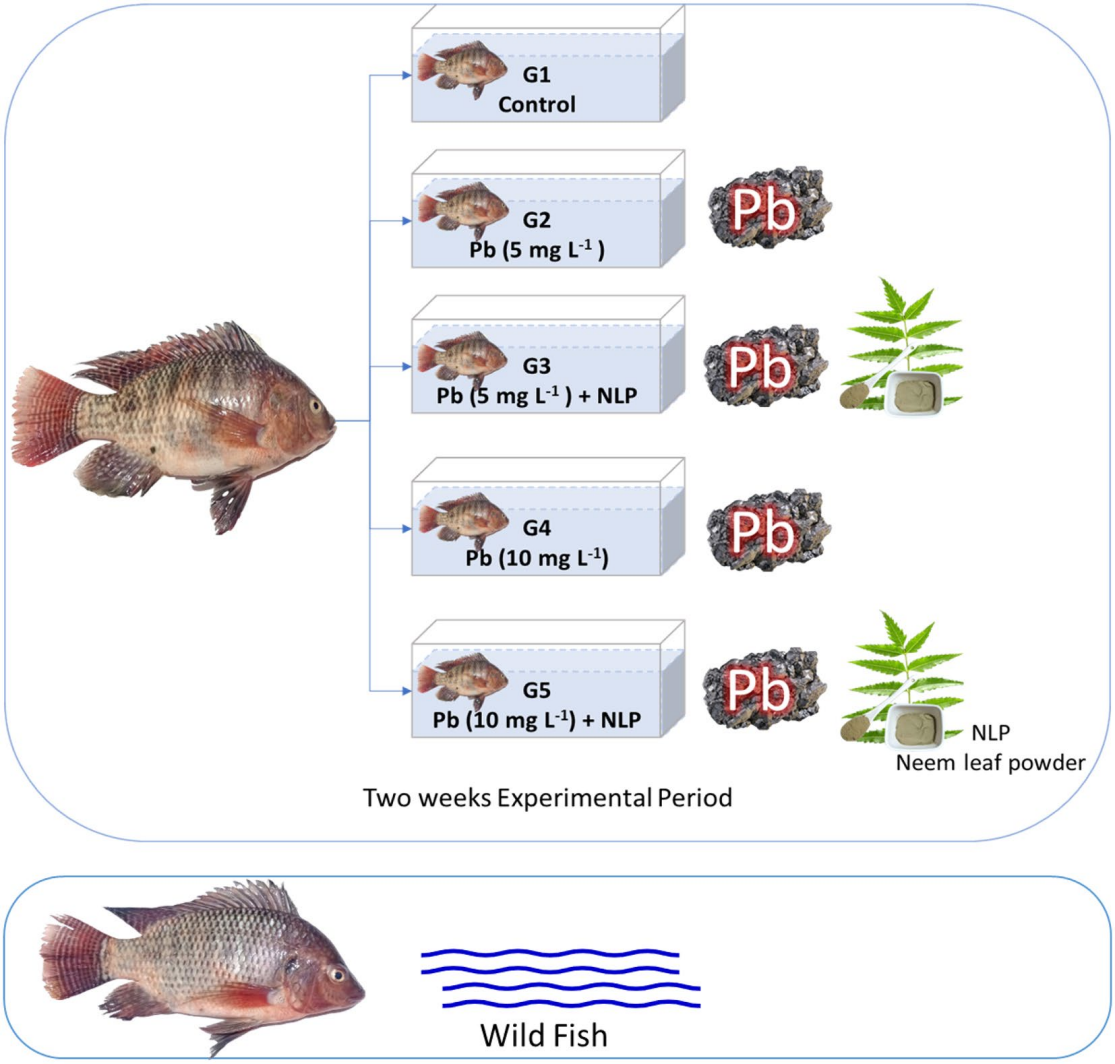
Scheme of the experiment.

A total of 150 mono-sex male *O. niloticus* weighing 20 ± 2 g, 2-month age were purchased from a private fish farm in Sharkia governorate, Egypt. They were distributed in 15 glass aquaria (40 × 30 × 100 cm) with a total volume of 60 L water. Two weeks post-acclimation, the fish were distributed into five groups (30 fish/ group with three replicates). G1 was assigned as a negative control without any treatments. Groups (2–5) were exposed to lead acetate dissolved in rearing water for two weeks at a concentration of 5 mg L^−1^ (G2 and G3) or 10 mg L^−1^ (G4 and G5)^49^. During the lead exposure period, all groups were reared under the same conditions, while G3 and G5 were treated with 1 g L^−1^ NLP^44^ (Fig. 1). At the end of the trial, five fish per aquarium were euthanized with Ictyoclove^®^ (France) for tissue collection (liver, gill, and muscle).

### Oxidant/antioxidant biomarkers assessment

Lipid peroxidation was measured by determining the amount of malondialdehyde (MDA) (mM g^-1^ protein) using Thiobarbituric Acid Reactive Substances assay (TBARS)^50^. Glutathione reduction (GSH) (mM g^-1^ protein) was evaluated according to Ellman^51^. The absorbance of produced colors was measured using a UNICO-UV-2100 spectrophotometer.

### DNA fragmentation test for apoptosis assessment

DNA fragmentation was sensed by diphenylamine. The formed blue color was quantified at 578 nm using a UNICO-UV-2100 spectrophotometer. The percentage of DNA fragmentation was stated as % of DNA fragmentation = (O.D Supernatant/O. D Supernatant + O.D Pellet) × 100^52^.

### Delta-aminolevulinic acid dehydratase (ALA-D) gene qRT-PCR

Tissue samples from the liver, gills, and muscles were collected in a triazole solution to measure gene expression. Total RNA was extracted using a QIAmp RNA mini kit (Qiagen, Germany) following the manufacturer’s protocol. The concentration and purity of the total RNA samples were checked using a Nanodrop ND-1000 spectrophotometer. For each sample, cDNA was produced utilizing a PrimeScript RT Reagent Kit (Takara, China) according to the instructions from the manufacturer. An SYBR Green-based PCR was staged in an automated thermal cycler (Bio-Rad) in a volume of 25 µL comprising 2 µL cDNA solution, 12.5 µL SYBR Premix Ex Taq (Takara), 1 µL primer (10 µmol L^−1^) (Table 1), and 8.5 µL ddH_2_O. The cycling reaction was completed according to the manufacturer’s recommendations via standard two-step PCR. Experimental Ct values were normalized to GAPDH as a housekeeping gene, and relative mRNA expression was computed compared to a control sample. Each assay included triplicate samples for each tested cDNA and no-template negative control; the expression relative to the control was assessed using the 2^−ΔΔCT^ method^53^.

**Table 1:** GAPDH and ALA.

Gene	Accession no	Sequence (5' to 3')	Product size (bp)
GAPDH	NM_001279552.1	F: 5'-GCTGTACATGCACTCCAAGG-3' R: 5'-ACTCAAACACACTGCTGCTG-3'	182
ALA-D	XM_003440087.4	F: 5'-GTTGTCACATCATCGCTCCC-3' R: 5'-TCTCTCACATCTCGCTCCAC-3'	250

D primer sequences of Nile tilapia.

### Histopathological assessment

Tissue samples from the gills, liver, kidneys, spleen, brain, and muscles of wild fish and the liver, gills, and muscles of experimental fish were collected and fixed in 10% neutral formalin buffer. Fixed samples were then treated using the paraffin embedding practice, sectioned utilizing Leica 2135, Germany microtome into 3–4 µm thick sections, and stained using H&E stain. A semi-quantitative scoring system of lesions in experimental fish was performed. A score of 0 indicated the absence of lesions, 1 indicated mild lesions (1–25% of tissue affected), 2 indicated moderate lesions (25–50% of tissue affected), and 3 demonstrated severe lesions (51%–100% of tissue affected).

### Statistical analysis

Statistics evaluation was completed using one-way ANOVA in SPSS version 21. Microscopic lesion scores were analyzed statistically using the Kruskal–Wallis test and a Mann–Whitney U test. The lesion scores are presented in a boxplot. *P-*values < 0.05 were counted as significant.

### Approval for animal experiments

The Institutional Animal Care and Use Committee (IACUC) (Vet CU 01122022596—Date of approval: 01/12/2022) and the ethical committee of the faculty of agriculture at Tanta University approved the experimental protocol and all methods in the present study for treating animals for scientific purposes (Approval No. AY_2019-2020_/Session 6/2020.01.13). All experiments were performed in accordance with relevant guidelines and regulations. Our reporting of research involving animals follows the recommendations of the ARRIVE guidelines. The plant materials used in this study were collected, examined, and botanically identified in the Faculty of Desert Agriculture, King Salman International University, Egypt, following the institutional guidelines and legislation (Voucher Specimen Number: *Neem*/DA-1/2023).

## Results

Water collected from the Mariotteya Canal was contaminated with a high lead level (Table 2). The mean level of lead was 0.6 ± 0.21 mg L^−1^ (*P* ˂ 0.01), which is much higher than the permissible limit (0.05 ppm). The rates of lead bioaccumulation were significantly elevated in the liver, gills, and muscles of *O. niloticus* from the Mariotteya Canal.

**Table 2:** Concentration of lead in water and tissue samples of wild *O. niloticus* collected from Mariotteya Nile stream.

Sample	Lead concentration	*P*-value	FAO permissible limit
Water	0.611 ± 0.21 mg L^−1^	˂ 0.01	0.05 mg L^−1^
Muscle	4.69 ± 1.2 mg kg^−1^	˂ 0.05	0.5–2 mg kg^−1^
Liver	11.08 ± 2.3 mg kg^−1^	˂ 0.01	
Gills	9.60 ± 3.1 mg kg^−1^	˂ 0.01	

Values are presented as mean ± SE (n = 3).

### Lipid peroxidation (MDA) concentration (mM g^−1^ protein)

**Figure 2:**
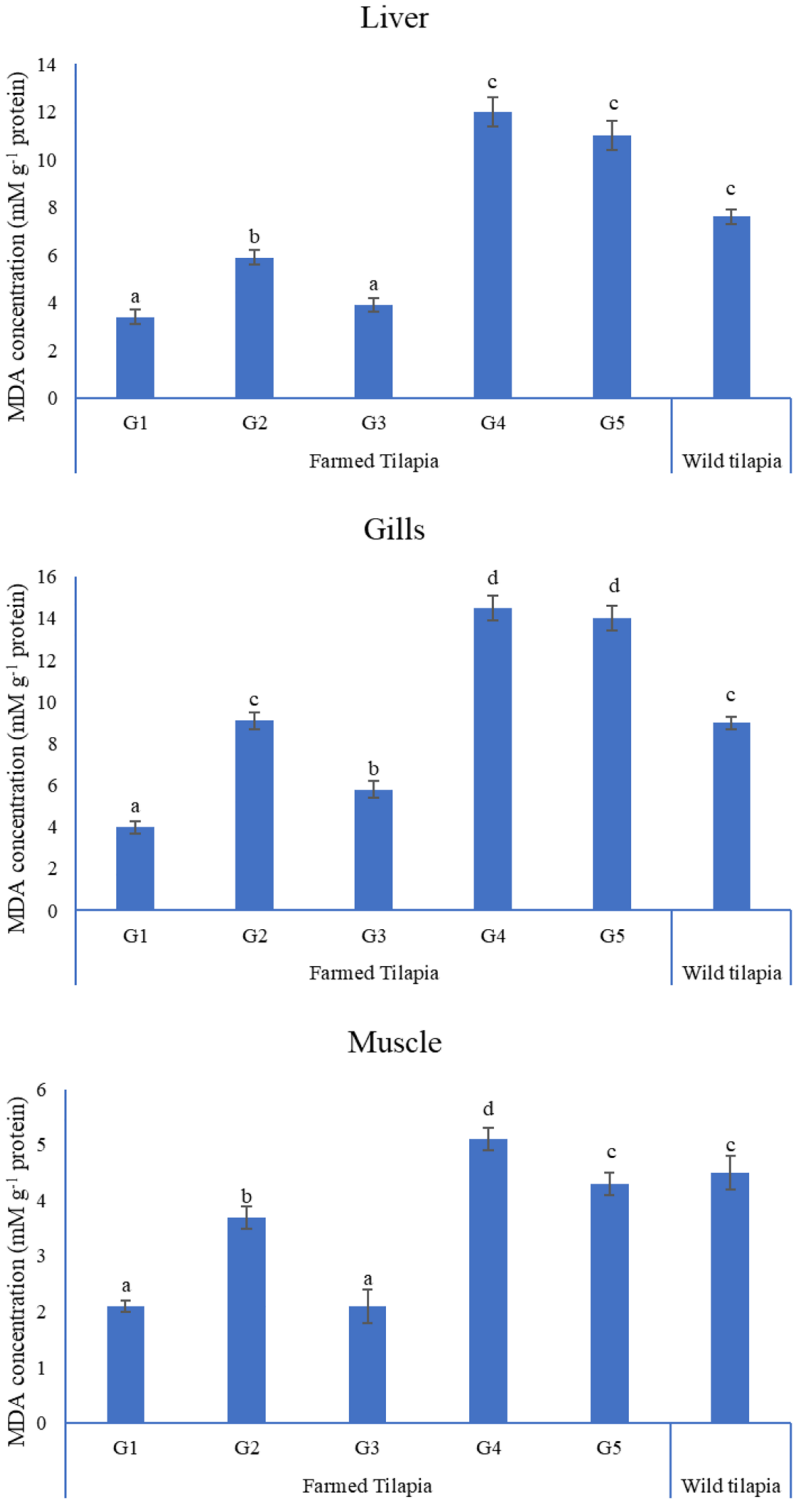
MDA concentration (mM g^−1^ protein) in tissues of experimental and wild tilapia. Bars with unique superscripts differ at (P < 0.05).

The lipid peroxidation concentration in all tissues of G2 was substantially increased compared to the control group, while that in G3 was significantly decreased to near control values. In addition, all tissues in wild tilapia demonstrated a significantly elevated concentration of MDA compared to the experimental control and G2, except for the gills, where the concentrations of lead were statistically insignificant. Exposure to 1 g L^−1^ NLP did not mitigate the hazardous effects of high lead acetate concentration in G5 (Fig. 2).

### Reduced glutathione (GSH) concentration (mM g^−1^ protein)

**Figure 3:**
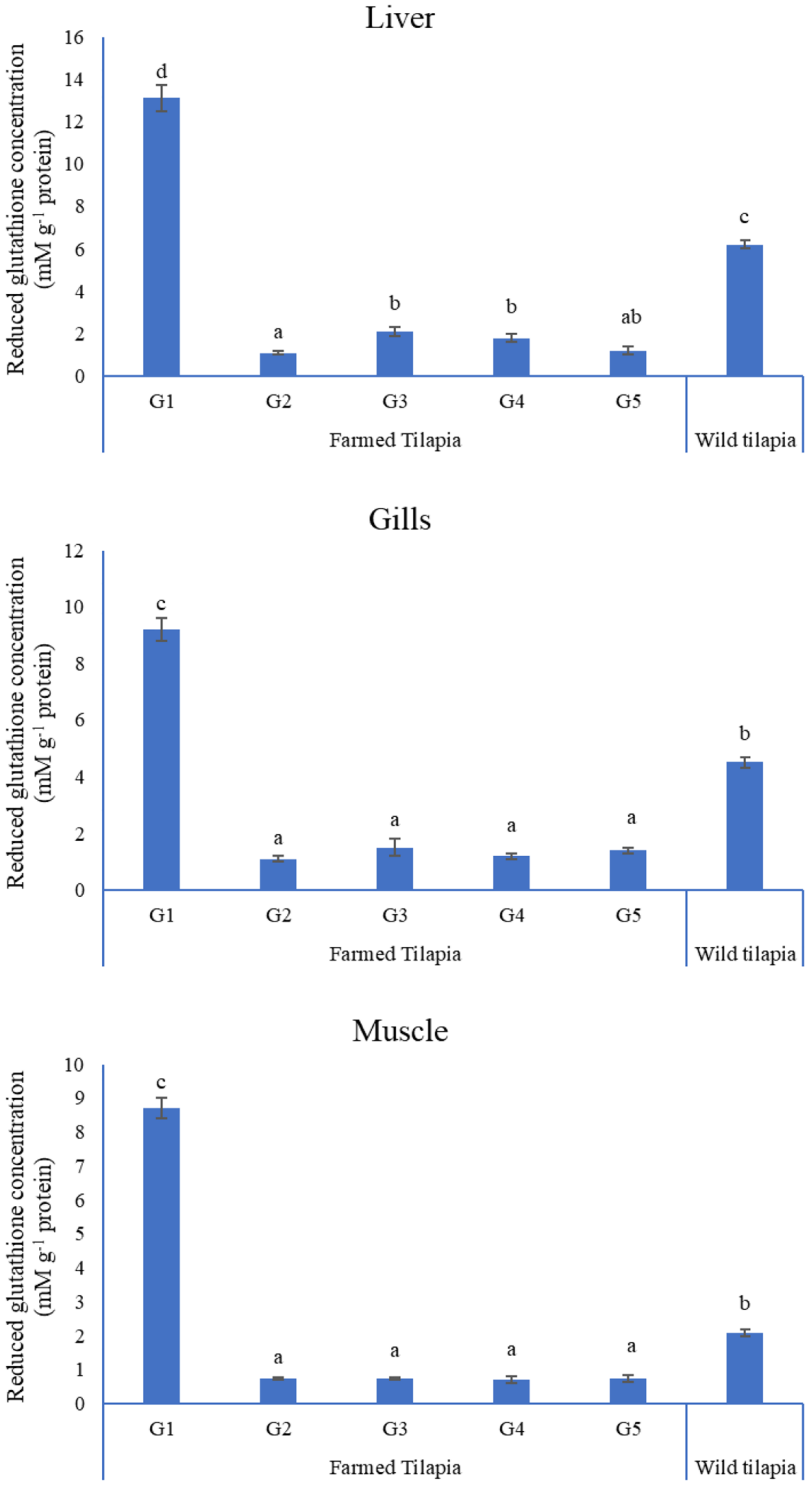
Reduced glutathione concentration (mM g^−1^ protein) in tissues of experimental and wild tilapia. Bars with different letters alter at (P < 0.05).

The level of GSH in the liver tissue of G3 was significantly increased compared to G2, with no significant alterations in other tissues of the same group. However, the content of GSH in all wild tissues was substantially higher than that in our treatment groups (G2, G3, G4, and G5) and lower than that of the control (G1) (Fig. 3).

### DNA fragmentation findings

**Figure 4:**
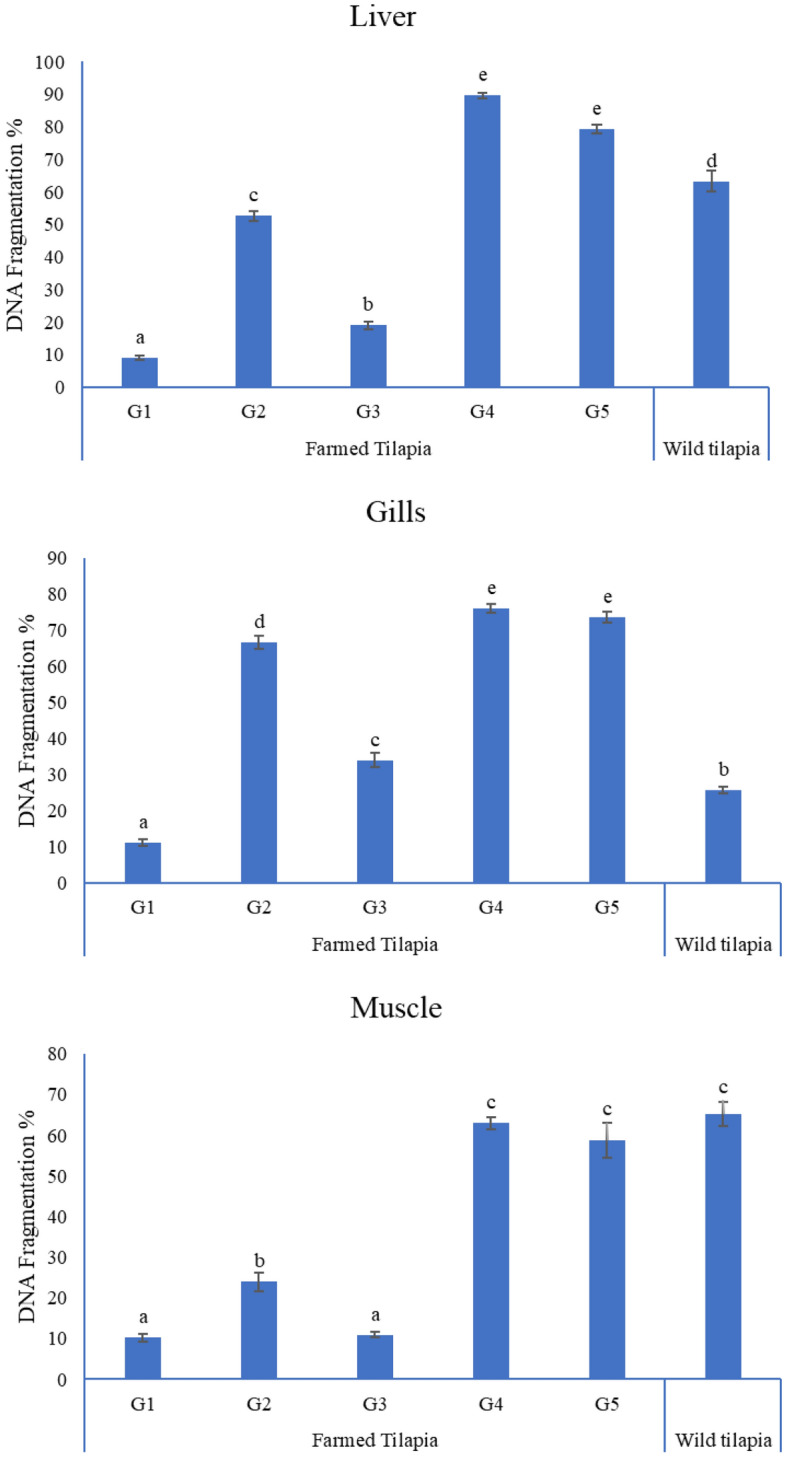
DNA fragmentation % in tissues of experimental and wild Nile tilapia. Bars showed different superscripts alter at (P < 0.05).

The percentage of DNA fragmentation in all tissues of G2 was significantly increased compared to the control group but significantly decreased in the liver, gills, and muscles of G3 compared to G2. Adding 1 g L^−1^ NLP did not reduce the genotoxic effect of 10 mg L^−1^ lead acetate in G5. Meanwhile, the DNA fragmentation percentage in all wild tilapia tissues was significantly higher than in the control (Fig. 4).

### ALA-D gene qRT-PCR

**Figure 5:**
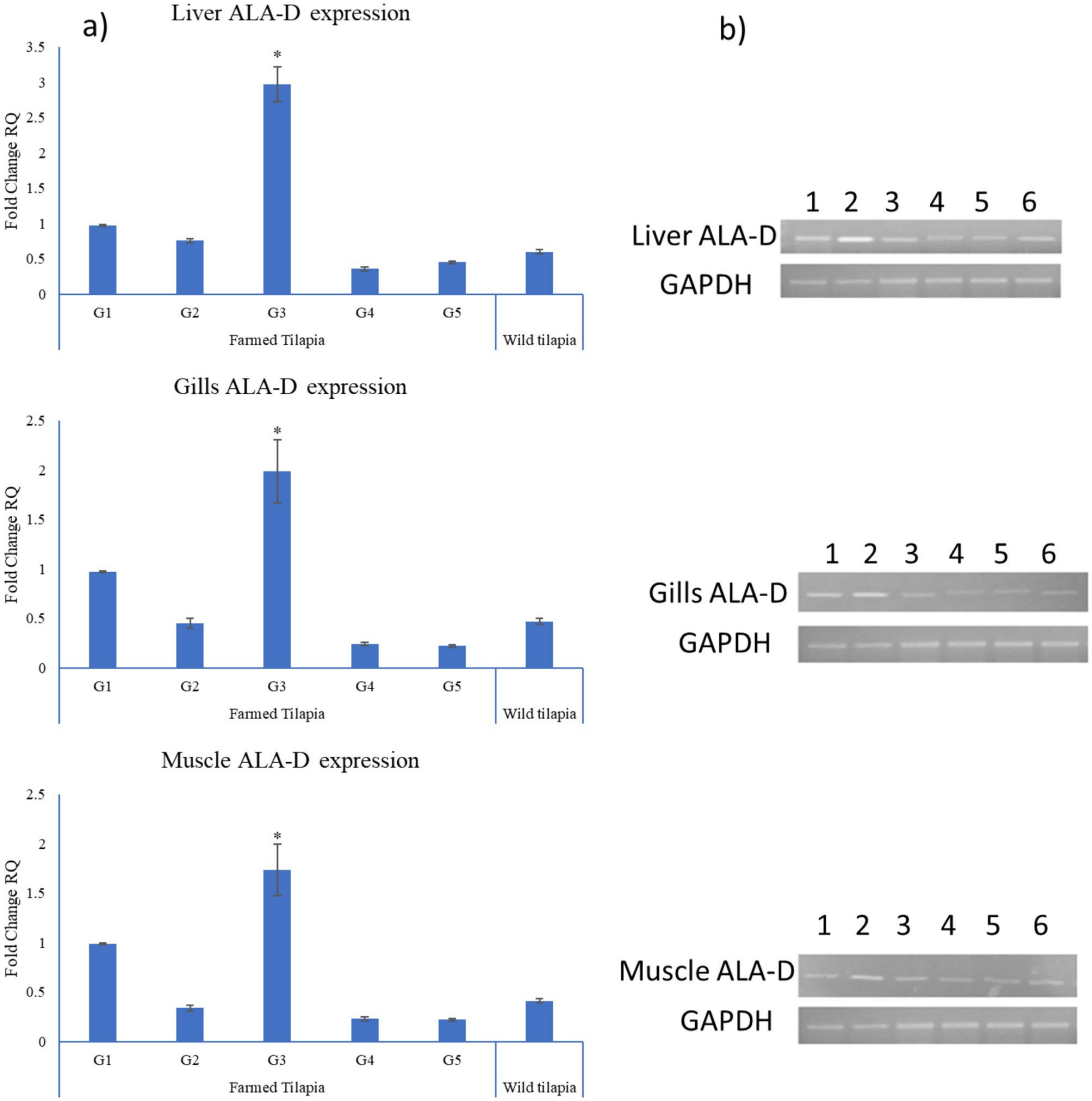
Quantitative RT-PCR of ALA-D gene expression of various groups and wild tilapia. (**a**) Evaluation of ALA-D gene expression in the liver, gills, and muscle organs in various groups and wild tilapia compared with the control. Values are expressed as means ± SE; n = 5. Bars bearing * are different at P < 0.05. (**b**) Cropped gel of electrophoretic mobility of quantitative RT-PCR products of ALA-D and GAPDH (internal control) genes on 2% agarose gel. Lane 1 = G1; Lane 2 = G3; Lane 3 = G2; Lane 4 = G5; Lane 5 = G4; Lane 6 = wild tilapia.

The ALAD gene was significantly overexpressed in G3 by 3-, 2-, and 1.8-fold in the liver, gills, and muscle, respectively, compared to other treatment groups. The expression of the ALAD gene was downregulated in all tissue samples of G2, G4, G5, and wild tilapia compared to the control (Fig. 5).

### Histopathological findings

**Figure 6:**
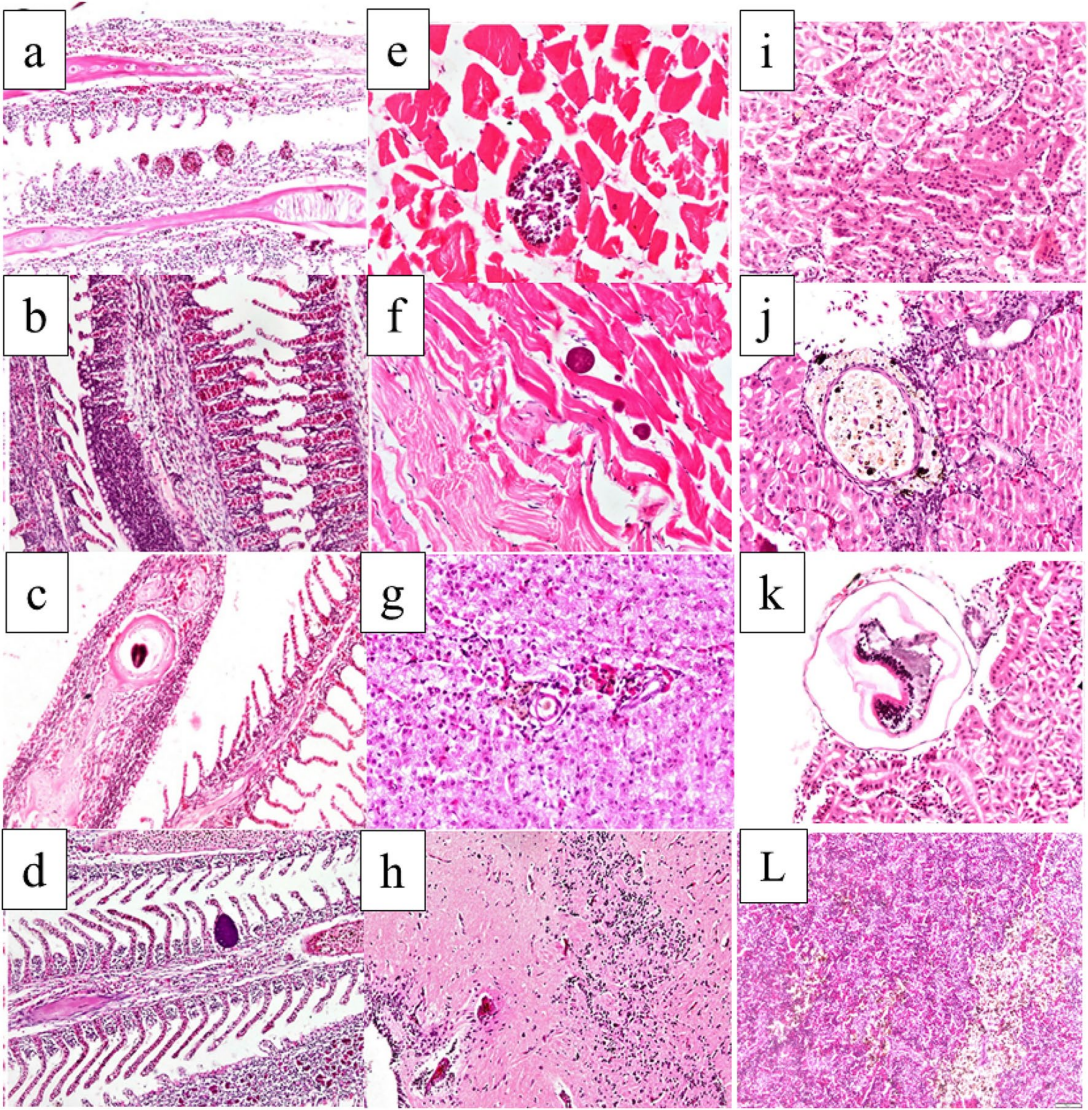
(**a–d**) Gills, *Oreochromis niloticus*. (**a**) Presence of aneurysm in the lamellar blood vessels at the tips of secondary gill lamellae, epithelial hyperplasia, inflammatory cell infiltration, and cartilaginous degeneration. (**b**) Severe epithelial and mucous cell hyperplasia, a fusion of secondary gill lamellae, and inflammatory cell infiltration. (**c**) Presence of encysted metacercaria in the cartilage of primary gill lamellae. (**d**) Presence of epitheliocystis in addition to severe inflammatory cell infiltration, including mononuclear and eosinophilic granular cells. (**e,f**) Muscle. (**e**) Presence of parasitic cyst inside the muscle bundles (*Myxobolus* spp.) together with atrophy and degeneration of muscle bundles. (**f**) Presence of multiple variable-sized rounded parasites between the muscle fibers (*Ichthyphonus* spp.) (H&E stain, ×400). (**g**) Liver and (**h**) brain of *Oreochromis niloticus*. (**g**) Eosinophilic granular cell infiltration in the portal area and solitary hepatocyte necrosis with karyopyknosis (H&E stain, ×400). (**h**) Perivascular lymphocytic cuffing and extensive gliosis (H&E stain, ×200). (**i–k**) Kidney and (**l**) spleen of *Oreochromis niloticus*. (**i**) Swelling of renal tubular epithelium and narrowing of the lumen. (**j**) Granuloma surrounded by delicate fibrous tissue and melanomacrophages. (**k**) Subcapsular encysted metacercaria surrounded by eosinophilic granular cells (H&E stain, ×400). (**l**) Activation of melanomacrophage centers in the spleen (H&E stain, ×200).

Fish samples from the Mariotteya Canal showed variable lesions in different organs. Microscopy of the gills revealed aneurysms at the tips of secondary gill lamellae, severe epithelial hypertrophy, and massive leukocytic infiltration in primary gill lamellae (Fig. 6a) in addition to areas of severe epithelial and mucous cell hyperplasia, with a fusion of secondary gill lamellae (Fig. 6b). Parasitic infestations were observed in the gills, including variable-sized encysted metacercaria enclosed in fibrous connective tissue capsules and surrounded by eosinophilic granular cells (EGC) and mononuclear cells in the branchial cartilage. The branchial cartilage was distorted, necrosed, and fragmented (Fig. 6c). Solitary epitheliocystis, an aggregation of bacteria in the branchial epithelium, was uncommonly observed in the branchial epithelium (Fig. 6d). Microscopy of the muscles showed different parasitic infestations identified based on their morphological appearance. There was encysted metacercaria accompanied by minimal tissue reaction and parasitic cysts inside the muscle bundles (*Myxobolus* spp.) together with atrophy and degeneration of muscle bundles (Fig. 6e). Moreover, multiple variable-sized rounded, purple-colored parasites were observed between the muscle fibers (*Ichthyphonus* spp.) (Fig. 6f). Liver microscopy showed EGC infiltration in the portal area in addition to solitary necrotic cells, which exhibited karyopyknosis (Fig. 6g). EGC was notable in the capsule of the hepatopancreas, along with infiltration of a few melanomacrophages in the hepatopancreas. Brain microscopy revealed perivascular lymphocytic cuffing and diffuse and focal areas of gliosis (Fig. 6h). It also showed neuronal degeneration and meningitis with leukocytic infiltration. Kidney microscopy revealed swelling of the tubular epithelium with lumen narrowing and necrotic cells showing karyopyknosis (Fig. 6i). Multiple granulomas were also observed with necrotic tissue surrounded by melanomacrophages, mononuclear cells, and fibrous capsules (Fig. 6j). Encysted metacercariae surrounded by fibrous capsules and EGC were noted (Fig. 6k). Regarding the spleen, there was activation of the melanomacrophage centers (Fig. 6l).

**Figure 7:**
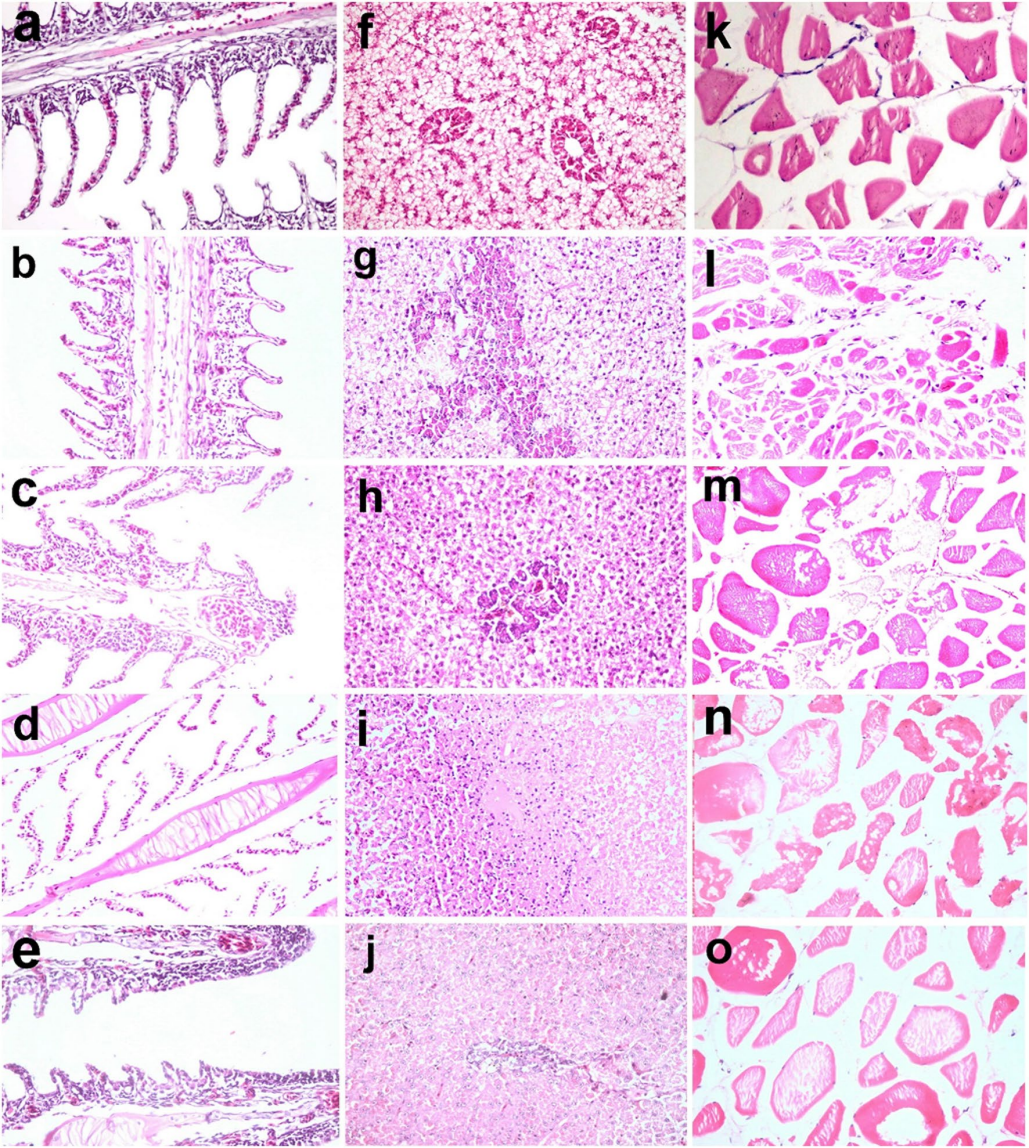
(**a–e**) Gills, (**f–j**) hepatopancreas, and (**k–o**) muscle of *Oreochromis niloticus*. (**a**) Normal histological structure in G1. (**b**) Epithelial hypertrophy of gill lamellae, inflammatory cell infiltration, and edema in primary gill lamellae in G2 compared to (**c**) decreased edema in G3. (**d**) Severe edema and necrosis of cartilage in primary gill lamellae in G4 compared to (**e**) slight edema and epithelial hypertrophy in G5. (**f**) Normal histological structure in G1. (**g**) Severe vacuolar degeneration, karyopyknosis, and necrosis of hepatocytes in G2, (**h**) which was decreased in G3. (**i**) Massive hepatocyte necrosis with intracytoplasmic hyaline bodies and hepatocyte dissociation in G4, (**j**) which was lessened in G5. (**k**) Normal histological structure in G1. (**l**) Degeneration and separation of muscle fibers with and atrophy and necrosis of muscle bundles and a few leukocytic infiltrations in G2. (**m**) Mild edema and disintegration of few muscle bundles in G3. (**n**) Necrosis and loss of muscle bundles in G4. (**o**) Separation and vacuolation of myofibrils in G5 (H&E stain, ×200).

**Figure 8:**
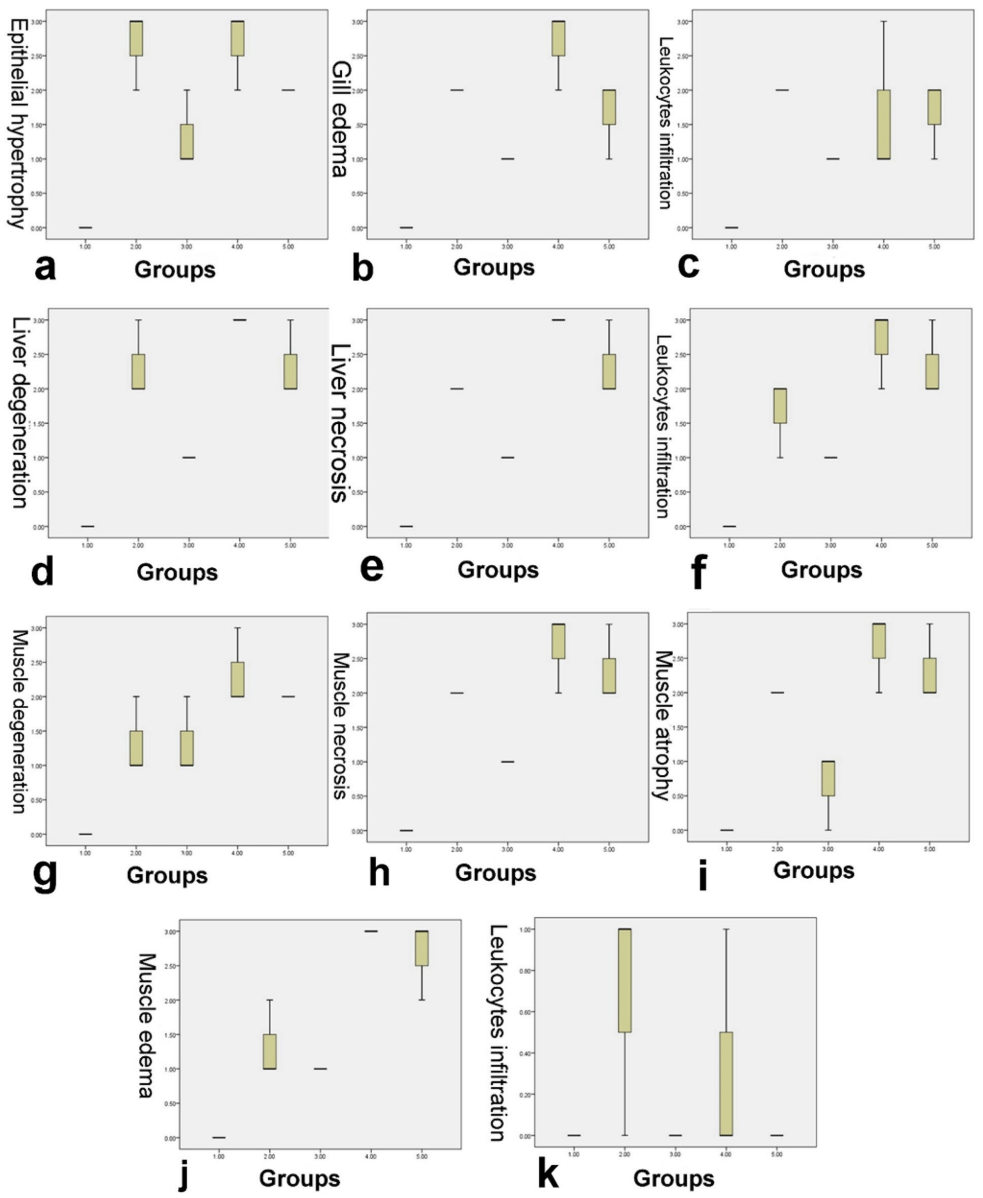
Boxplot of the histopathological scores. (**a–c**) Gill lesion scores. (**d–f**) Liver lesion score. (**g–k**) Muscle lesion scores. The boxes represent the interquartile range (IQR). Thick middle lines are the medians. The maximum and minimum values are represented by thin horizontal lines at the top and bottom.

In the experimental study, lead exposure caused histopathological lesions in fish's gills, liver, and muscles, and its severity intensified with rising lead concentration. Gill microscopy in the control group revealed normal histologic structures (Fig. 7a), whereas in G2, it showed epithelial hypertrophy, epithelial lifting, leukocyte infiltration, and edema in the primary gill lamellae (Fig. 7b). These lesions were decreased in G3 exposed to neem in water (Fig. 7c). In G4 exposed to a high concentration of lead, severe edema and epithelial and cartilage necrosis in the primary gill lamellae were recorded (Fig. 7d), compared to slight edema and epithelial hypertrophy in G5 (Fig. 7e). The microscopic lesion scores in the gills did not show a significant difference between groups except in edema and leukocytic infiltration, which were significantly lower in G3 than in G2 (Fig. 8).

The hepatopancreas microscopy showed typical histological structure in G1 (Fig. 7f) but revealed severe vacuolar degeneration, karyopyknosis, and moderate necrosis of hepatocytes in G2 (Fig. 7g), which was decreased in G3 (Fig. 7h). In G4, there were massive areas of hepatocyte necrosis, solitary hepatocyte necrosis, intracytoplasmic eosinophilic hyaline bodies, and hepatocyte dissociation in addition to necrosis of the pancreatic cells (Fig. 7i). Fewer lesions were observed in G5 (Fig. 7j). The hepatic lesion scores showed a significant difference between groups. Hepatic necrosis and degeneration were considerably decreased in G3 compared to G2 and G5 compared to G4, but not significantly (Fig. 8).

Muscle microscopy in the treatment groups showed more lesions than G1, which had a normal histological structure (Fig. 7k). G2 showed degeneration, separation of muscle fibers, atrophy, and necrosis of muscle bundles, and a few leukocytic infiltrations (Fig. 7L). In contrast, G3 muscles had mild edema and disintegration of a few muscle bundles (Fig. 7m). In G4, necrosis and loss of muscle bundles were more evident than at the lower dose (Fig. 7n). In G5, there was separation and vacuolation of myofibrils, which was less severe than in the previous group (Fig. 7o). The severity of degeneration, necrosis, atrophy, and muscular edema was significantly increased by lead exposure in G2 and G4 compared to the control. Muscle necrosis, atrophy, and leukocytic infiltration were significantly decreased in G3 but not in G5 (Fig. 8).

### GC–Ms analysis report

The chemical structure of the bio-sorbent influences the metal adsorption process. The adsorption of metal ions occurs due to interaction by the reactive functional groups of the biosorbent. The phytochemical components of neem leaf extract are alkaloids, carbohydrates, reducing sugar, flavonoids, glycoside, tannins, phenolic compounds, and saponins (Supplementary file). The GC–MS results showed the presence of 38 compounds. Nepetalactol (Isocalamendiol) was highest in this extract at RT 9.76 (9.39%), followed by 7-methyl-*Z*-tetradecen-1-ol acetate at RT 10.66 (7.59%), ethanimidothoic acid at RT 5.33 (6.92%), sarreroside at RT 15.87 (6.48%), d-gala-l-ido-octonic amide at RT 7.05 (6.27%), ledene oxide-(II) at RT 14.29 (6.01%), stevioside at RT 6.77 (5.92%), carophyllene oxide at RT 17.16 (4.78%), 1,3,5-triazine-2,4-diamine at RT 11.75 (3.99%), tridecanedial at RT 6.30 (3.92%), and other miscellaneous long-chain compounds (38.73%).

## Discussion

The concentrations of heavy metals in water bodies near industrial areas and the aquatic species that inhabit such places have been studied to evaluate the degree of contamination^54^. Lead concentration in the Mariotteya Canal was found to exceed the permissible limit recommended by the FAO^42^, which is similar to the concentration recorded in the present trial. The toxicity of sublethal concentrations of lead in various organs is mainly mediated by the heightened production of ROS, which subsequently causes the oxidation of biomolecules and pathological alterations.

Lead distribution in the wild tilapia organs we examined was in the following order: liver ˃ gills ˃ muscle. The highest lead concentration (11.08 ± 2.3 mg kg^−1^) appeared in the liver, while the lowest was found in the muscles (4.69 ± 1.2 mg kg^−1^). In contrast, the highest lead concentration was detected in the gills of *C. gariepinus*, *O. niloticus,* and rainbow trout^55^. This might be due to the difference in exposure time, the concentration of lead in the aquatic ecosystem, the gills' adaptive mechanism, and the liver's bio accumulative nature.

MDA, GSH, and DNA fragmentation are valuable biomarkers of lead-triggered oxidative stress in aquatic animals^56^. In the present work, the DNA fragmentation % and MDA (mM g^−1^ protein) concentration in all fish tissues after lead exposure were significantly increased, while they were significantly decreased in all tissues in G3. Furthermore, GSH concentration was significantly reduced in the tissues of all lead groups but increased dramatically in the liver of G3 compared to G2, indicating that 1 g L^−1^ NLP could protect fish against 5 mg L^−1^ lead-induced oxidative damage but not against 10 mg L^−1^ lead. Our results demonstrated that the GSH concentration in different tissues of Mariotteya Nile tilapia was substantially higher than in lead-exposed groups. These results agreed with Shaukat et al.^19^, who noted a significant increase in peroxidase activity in the liver and gills of *Labeo rohita* after lead exposure. This finding can be attributed to natural defenses, adaptive mechanisms, and the compensatory action of antioxidant enzymes in different tissues against free radical-induced toxicity^57^. Zhang et al.^58^ stated that intensive oxidative stress might boost and activate gene expression encoding antioxidant enzymes to iterate the imbalance caused by oxidative damage.

MDA levels were raised in the brains of *Clarias batrachus* following 60 d of lead exposure^59^ as well as in *Cyprinus carpio* L., *Oncorhynchus mykiss Walbaum*, and *Acipenser baeri Brandt* spp., and SOD activity was reduced^60^. Moreover, total GSH and GSH/GSSG levels declined after 7 days of lead exposure in the liver of freshwater fish *O. niloticus*^61^. Zhang et al.^58^ attributed the disturbance of the adaptive mechanism of GSH in the liver of goldfish *Carassius auratus* to high GSH consumption, resulting in a reduction of the GSH/GSSG ratio. Other studies of lead exposure have reported a significant reduction in the levels of GSH, SOD, GST, and CAT in the liver of *Clarias gariepinus* and *O. niloticus*^62^**.** Lead causes a significant elevation in GPX activity in the *O. niloticus* liver^61,63^ and the *Clarias batrachus* brain^59^. These results indicate that lead could cause oxidative damage to the cell through the output of ROS by Haber–Weiss and Fenton-type mechanisms that subsequently induce lipid peroxidation, protein modifications, and DNA damage^63^. In addition, MDA elevation reacts with amino groups on proteins and other biomolecules to produce an assortment of adducts^64^. These adducts include cross-linked products^65^ and adducts with DNA bases that are mutagenic^66^ and carcinogenic^67^.

Heme synthesis suppression is one of the significant impacts of lead. Lead inhibits ALAD activity through binding of the sulphydryl (SH) group, which is required for optimal enzyme activity^68^, or dislodges a zinc ion at the metal-binding position that subsequently alternates the enzymes’ quaternary structure^69^. In the present analysis, ALA-D gene expression was significantly depressed in the liver, gills, and muscles of wild and experimental Nile tilapia subjected to different lead concentrations. These results agreed with Li et al.^70^, who showed that waterborne lead exposure results in the downregulation of ALA-D transcription. A high lead concentration in the liver of *Palaemonetes turcorum* is correlated with ALA-D enzyme inhibition^71^. A high level of lead in the liver and blood samples of *Prochilodus lineatus* collected from contaminated water bodies in Buenos Aires, and La Plata with depletion of ALA-D activity was also reported^72^. ALA-D is considered a specific biomarker for lead exposure due to the negative correlations between blood lead concentration and ALA-D inhibition in fish species^73^ and tissues of freshwater invertebrates^74^.

As a consequence of ALA-D inhibition, *δ*-ALA is accumulated in the blood and stimulates the generation of ROS^75^, resulting in oxidative stress^76^. Moreover, oxidative DNA damage due to ALA accumulation occurs through the oxidation of ALA to 4,5-dioxovaleric acid, an efficient alkylating agent of quinine moieties in both the nucleosides and DNA^77^. Likewise, ALA accumulation results in the overproduction of the 8-oxo-7, 8-dihydro-2-deoxyguanosine, and 5-hydroxyl-2-deoxycytidine incriminated in ALA-induced DNA damage^78^ through interaction with zinc-binding sites on the DNA-associated protein protamine^79^. Moreover, lead induces neurotoxicity because ALA is structurally similar to γ-aminobutyric acid, stimulating γ-aminobutyric acid receptors in the nervous system^69^. In the present study, the depression of ALA-D gene expression in different tissues was considered a major biomarker related to environmental pollution.

Fish exposure to metals can induce immune system malfunctions, increasing the mortality rate^80^. Lead generally impairs fish's immune systems and makes them more vulnerable to infections, as Paul et al.^81^ suggested. Fish exposed to 9.4 mg L^−1^ lead acetate for 4 days showed a reduction in phagocytic activity and the phagocytic index after challenge with *S. aureus*, inhibition of antimicrobial substances such as nitric oxide (NO) and myeloperoxidase (MPO), severe damage in the intestinal epithelium, and downregulation in TNF-α transcription. In the current study, granulomas with a central area of necrosis and fibrous capsule were observed in various organs, and these elicited a surrounding inflammatory response. Although little information is available on the bacteria causing granuloma formation in tilapia in Egypt, a wide range of bacterial infections have been reported in other countries. Many causes of granuloma formation have been implicated and reported in tilapia. *Streptococcus agalactiae* may cause granulomatous inflammation in the ovaries and testes^82^. Chronic infection of *Pseudomonas* spp. causes granulomata of epithelioid and encapsulated necrotic tissue^83^. In addition, granulomatous inflammation is linked with *Francisella noatunensis* subsp. *orientalis* in farmed tilapia (*O. niloticus* and *O. aureus*) in China^84^**.**

In the present work, lead caused various lesions in the gills similar to those reported previously^85^. Muscle lesions, such as the separation of muscle bundles and reduced compactness, were also recorded previously^86^. Furthermore, liver lesions such as cytoplasmic inclusions, swelling, hydropic degeneration, and necrosis are typically observed^87^. To decrease the pathological effect of lead toxicity, the neem plant is a possible candidate that can scavenge lead from water, thereby lowering its toxic effects. Neem has many pharmacological properties that can enhance water quality^88,89^. The current study shows that adding neem to water can lower the pathological lesions in various organs induced by lead toxicity. This was very apparent in G3 but not in G5 exposed to the high lead concentration, which might be due to the saturation of the neem by lead in G5. Bhattacharyya and Sharma^44^ found that 1.2 g L^−1^ NLP could eliminate as much as 93% of lead in 96 h from a solution of 300 mg L^−1^ using batch adsorption technology. The absorption constantly improved in the pH range of 2–7, beyond which the adsorption could not be carried out due to the precipitation of the metal. Adsorption of metal cations depends upon the nature of adsorbent surface, the distribution of metal cation and pH of the system. Depending on the pH of the solution, the organic functional groups on the adsorbent surface may become positively or negatively charged. The surface of activated carbon becomes negatively charged at pH values over pHzpc (zero-point charge) because of the ionization of acidic carbon–oxygen surface groups, and surface groups exhibit a potent electrostatic attraction to metal species. By analyzing the chromatography of neem tree extract using GC–Ms, the extract contained many long-chain organic carbon, aliphatic, and aromatic functional groups. The properties of these compounds should be evaluated in the future. Aromatic compounds contain active center groups such as hydroxyl (OH-) and ketones (C=O) and contain high electronegativity of atoms such as nitrogen (N_2_), sulfur (S), and silicon (Si). Heterocyclic compounds, including polysaccharides and carbohydrates, have many active centers and electronegative atoms. All active groups, electronegative atoms, polysaccharide compounds, carbohydrates, and oxygen atoms, in the case of the ionic state, have partial ionization that can adsorb the ionized lead element in solution, and this adsorption is a chemical as well as physical adsorption^90^. The coordination tendency of aromatic electrons to donate groups involving hydroxyl, ether, and phenyl groups is much superior to that in corresponding aliphatic groups. The high tendency for aromatic derivative coordination comes from their higher electron density and ability to participate in conjugation. This conjugation alleviates the produced metal complexes. As a result, the coordination and complex formation tendency of lignin are much higher than that in cellulose and hemicellulose. Therefore, metal uptake by NLP can be linked to its higher lignin content. Furthermore, previous studies have found that the alkaline pH of the aquatic environment increases the dissociation of the functional groups such as OH and fiber carbonaceous C–OH, which consequently increases the metal uptake (adsorption efficiency) of the biosorbent^91^.

## Conclusion

Chemical analysis of water samples and fish organs collected from the Mariotteya Canal showed a high lead concentration exceeding FAO's permissible limits. Sublethal chronic exposure to lead in the aquatic environment results in oxidative stress, genotoxic effects, and various pathological alterations in Nile tilapia at this location. Tissue samples (gills, liver, and muscle) collected from wild tilapia and experimental tilapia exposed to 5–10 mg L^−1^ lead acetate showed an increase in lipid peroxidation and DNA oxidation as well as a reduction in antioxidant defense systems and expression level of ALAD. The pathological lesions were directly correlated with lead concentration. Neem leaf powder at 1 g L^−1^ mitigated the oxidative stress and pathological lesions of 5 mg L^−1^ lead acetate water toxicity in a static exposure regime.

## Supplementary Information

Supplementary Information 1.

Supplementary Information 2.

Supplementary Information 3.

Supplementary Information 4.

Supplementary Information 5.

## Data Availability

The datasets used and/or analysed during the current study available from the corresponding author on reasonable request.

## References

[1] El-Dahhar AA (2022). Evaluation of the nutritional value of *Artemia nauplii* for European seabass (*Dicentrarchus labrax* L.) larvae. Aquac. Fish..

[2] Mourad MM, Shahin SA, El-Ratel IT, El Basuini MF (2022). Effect of treating eggs with coenzyme Q10 (CoQ10) on growth variables, histomorphometry, and antioxidant capacity in red tilapia (*Oreochromis aureus* × *Oreochromis mossambicus*) larvae. Animals.

[3] Hussein EE (2023). Effect of dietary sage (*Salvia officinalis* L.) on the growth performance, feed efficacy, blood indices, non-specific immunity, and intestinal microbiota of European sea bass (*Dicentrarchus labrax*). Aquac. Rep..

[4] El Basuini MF (2022). Growth variables, feed efficacy, survival rate, and antioxidant capacity of European seabass (*Dicentrarchus labrax* L.) larvae treated with coenzyme Q10 or lipoic acid. Aquac. Rep..

[5] Dossou S (2021). Dynamical hybrid system for optimizing and controlling efficacy of plant-based protein in aquafeeds. Complexity.

[6] Schar D (2021). Twenty-year trends in antimicrobial resistance from aquaculture and fisheries in Asia. Nat. Commun..

[7] Zhu X (2022). Improved growth performance, digestive ability, antioxidant capacity, immunity and *Vibrio harveyi* resistance in coral trout (*Plectropomus leopardus*) with dietary vitamin C. Aquac. Rep..

[8] Tadese DA (2022). The role of currently used medicinal plants in aquaculture and their action mechanisms: A review. Rev. Aquac..

[9] Tran NT (2022). Application of heat-killed probiotics in aquaculture. Aquaculture.

[10] El Basuini MF (2022). Dietary Guduchi (*Tinospora cordifolia*) enhanced the growth performance, antioxidative capacity, immune response and ameliorated stress-related markers induced by hypoxia stress in Nile tilapia (*Oreochromis niloticus*). Fish Shellfish Immunol..

[11] El Basuini MF (2021). The influence of dietary coenzyme Q10 and vitamin C on the growth rate, immunity, oxidative-related genes, and the resistance against *Streptococcus agalactiae* of Nile tilapia (*Oreochromis niloticus*). Aquaculture.

[12] Ghodrati M, Hosseini Shekarabi SP, Rajabi Islami H, Shenavar Masouleh A, Shamsaie Mehrgan M (2021). Singular or combined dietary administration of multi-strain probiotics and multi-enzyme influences growth, body composition, digestive enzyme activity, and intestinal morphology in Siberian sturgeon (*Acipenser baerii*). Aquac. Nutr..

[13] Shekarabi SPH, Javarsiani L, Mehrgan MS, Dawood MAO, Adel M (2022). Growth performance, blood biochemistry profile, and immune response of rainbow trout (*Oncorhynchus mykiss*) fed dietary Persian shallot (*Allium stipitatum*) powder. Aquaculture.

[14] Shekarabi SPH, Ghodrati M, Dawood MAO, Masouleh AS, Roudbaraki AF (2022). The multi-enzymes and probiotics mixture improves the growth performance, digestibility, intestinal health, and immune response of Siberian sturgeon (*Acipenser baerii*). Ann. Anim. Sci..

[15] Wan-Mohtar WAAQI, Ibrahim MF, Rasdi NW, Zainorahim N, Taufek NM (2022). Microorganisms as a sustainable aquafeed ingredient: A review. Aquac. Res..

[16] Bashir, I. *et al.* Concerns and threats of contamination on aquatic ecosystems. in *Bioremediation and Biotechnology*. 1–26. 10.1007/978-3-030-35691-0_1 (Springer, 2020).

[17] Teiba I (2020). Diversity of the photosynthetic bacterial communities in highly eutrophicated yamagawa bay sediments. Biocontrol Sci..

[18] Teiba I (2020). Use of purple non-sulfur photosynthetic bacteria (*Rhodobacter sphaeroides*) in promoting ciliated protozoa growth. Biocontrol. Sci..

[19] Shaukat N, Javed M, Ambreen F, Latif F (2018). Oxidative stress biomarker in assessing the lead induced toxicity in commercially important fish, *Labeo rohita*. Pak. J. Zool..

[20] Ali H, Khan E, Ilahi I (2019). Environmental chemistry and ecotoxicology of hazardous heavy metals: Environmental persistence, toxicity, and bioaccumulation. J. Chem..

[21] Bury N (2009). Metal contamination in aquatic environments: Science and lateral management. Freshw. Biol..

[22] Wang W-X (2013). Prediction of metal toxicity in aquatic organisms. Chin. Sci. Bull..

[23] Luoma SN, Rainbow PS (2008). Metal Contamination in Aquatic Environments: Science and Lateral Management.

[24] Livingstone DR (2001). Contaminant-stimulated reactive oxygen species production and oxidative damage in aquatic organisms. Mar. Pollut. Bull..

[25] Lushchak VI (2011). Adaptive response to oxidative stress: Bacteria, fungi, plants and animals. Comp. Biochem. Physiol. C Part Toxicol. Pharmacol..

[26] *The Toxicology of Fishes*. 10.1201/9780203647295 (CRC Press, 2008).

[27] Singhal SS (2015). Antioxidant role of glutathione *S*-transferases: 4-Hydroxynonenal, a key molecule in stress-mediated signaling. Toxicol. Appl. Pharmacol..

[28] Farombi E, Adelowo O, Ajimoko Y (2007). Biomarkers of oxidative stress and heavy metal levels as indicators of environmental pollution in African cat fish (*Clarias gariepinus*) from Nigeria Ogun River. Int. J. Environ. Res. Public Health.

[29] Flora G, Gupta D, Tiwari A (2012). Toxicity of lead: A review with recent updates. Interdiscip. Toxicol..

[30] Bardag-Gorce F, French SW (2011). Delta-aminolevulinic dehydratase is a proteasome interacting protein. Exp. Mol. Pathol..

[31] Bernard A, Lauwerys R (1987). Metal-induced alterations of delta-aminolevulinic acid dehydratase. Ann. N. Y. Acad. Sci..

[32] Nilsson R (2009). Discovery of genes essential for heme biosynthesis through large-scale gene expression analysis. Cell Metab..

[33] Droin C (2021). Space-time logic of liver gene expression at sub-lobular scale. Nat. Metab..

[34] Campana O, Sarasquete C, Blasco J (2003). Effect of lead on ALA-D activity, metallothionein levels, and lipid peroxidation in blood, kidney, and liver of the toadfish Halobatrachus didactylus. Ecotoxicol. Environ. Saf..

[35] Tchounwou PB, Yedjou CG, Patlolla AK, Sutton DJ (2012). Heavy metal toxicity and the environment. Exp. Suppl..

[36] Dane H, Şişman T (2015). Histopathological changes in gill and liver of Capoeta capoeta living in the Karasu River, Erzurum. Environ. Toxicol..

[37] Shahid S (2022). Detecting aquatic pollution using histological investigations of the gills, liver, kidney, and muscles of *Oreochromis niloticus*. Toxics.

[38] Sia Su GL, Ramos GB, Sia Su MLL (2013). Bioaccumulation and histopathological alteration of total lead in selected fishes from Manila Bay, Philippines. Saudi J. Biol. Sci..

[39] Abu-Elala NM (2018). Aquatic environmental risk assessment of chitosan/silver, copper and carbon nanotube nanocomposites as antimicrobial agents. Int. J. Biol. Macromol..

[40] Abu-Elala NM, Shaalan M, Ali SE, Younis NA (2021). Immune responses and protective efficacy of diet supplementation with selenium nanoparticles against cadmium toxicity in *Oreochromis niloticus*. Aquac. Res..

[41] El Basuini MF (2022). Microbial, immune and antioxidant responses of Nile tilapia with dietary nano-curcumin supplements under chronic low temperatures. Aquac. Fish..

[42] Eissa AE, Tharwat NA, Zaki MM (2013). Field assessment of the mid winter mass kills of trophic fishes at Mariotteya stream, Egypt: Chemical and biological pollution synergistic model. Chemosphere.

[43] Ayob S (2021). A review on adsorption of heavy metals from wood-industrial wastewater by oil palm waste. J. Ecol. Eng..

[44] Bhattacharyya KG, Sharma A (2004). Adsorption of Pb(II) from aqueous solution by *Azadirachta indica* (neem) leaf powder. J. Hazard. Mater..

[45] Sharma A, Bhattacharyya KG (2005). Adsorption of chromium (VI) on *Azadirachta indica* (neem) leaf powder. Adsorption.

[46] Sharma A, Bhattacharyya KG (2005). *Azadirachta indica* (neem) leaf powder as a biosorbent for removal of Cd(II) from aqueous medium. J. Hazard. Mater..

[47] Boeke SJ (2004). Safety evaluation of neem (*Azadirachta indica*) derived pesticides. J. Ethnopharmacol..

[48] Omar WA, Mikhail WZA, Abdo HM, Abou El Defan TA, Poraas MM (2015). Ecological risk assessment of metal pollution along Greater Cairo Sector of the River Nile, Egypt, using Nile tilapia, *Oreochromis niloticus*, as bioindicator. J. Toxicol..

[49] Fadl SE (2021). Effect of adding Dunaliella algae to fish diet on lead acetate toxicity and gene expression in the liver of Nile tilapia. Toxin Rev..

[50] Ohkawa H, Ohishi N, Yagi K (1979). Assay for lipid peroxides in animal tissues by thiobarbituric acid reaction. Anal. Biochem..

[51] Ellman GL (1959). Tissue sulfhydryl groups. Arch. Biochem. Biophys..

[52] Abou-Zeid SM, AbuBakr HO, Mohamed MA, El-Bahrawy A (2018). Ameliorative effect of pumpkin seed oil against emamectin induced toxicity in mice. Biomed. Pharmacother..

[53] Livak KJ, Schmittgen TD (2001). Analysis of relative gene expression data using real-time quantitative PCR and the 2^−ΔΔCT^ method. Methods.

[54] Malhat F (2011). Distribution of heavy metal residues in fish from the River Nile tributaries in Egypt. Bull. Environ. Contam. Toxicol..

[55] Al-Balawi HFA (2013). Effects of sub-lethal exposure of lead acetate on histopathology of gills, liver, kidney and muscle and its accumulation in these organs of *Clarias gariepinus*. Braz. Arch. Biol. Technol..

[56] Zhai Q (2017). Dietary Lactobacillus plantarum supplementation decreases tissue lead accumulation and alleviates lead toxicity in Nile tilapia (*Oreochromis niloticus*). Aquac. Res..

[57] Guyonnet D, Siess M-H, Le Bon A-M, Suschetet M (1999). Modulation of phase II enzymes by organosulfur compounds from allium vegetables in rat tissues. Toxicol. Appl. Pharmacol..

[58] Zhang JF, Wang XR, Guo HY, Wu JC, Xue YQ (2004). Effects of water-soluble fractions of diesel oil on the antioxidant defenses of the goldfish, *Carassius auratus*. Ecotoxicol. Environ. Saf..

[59] Maiti AK, Saha NC, Paul G (2010). Effect of lead on oxidative stress, Na^+^ K^+^ ATPase activity and mitochondrial electron transport chain activity of the brain of *Clarias batrachus* L.. Bull. Environ. Contam. Toxicol..

[60] Salaah SM, El-Gaar DM, Gaber HS (2022). Potential effects of dietary chitosan against lead-induced innate immunotoxicity and oxidative stress in Nile tilapia (*Oreochromis niloticus*). Egypt. J. Aquat. Res..

[61] Eroglu A, Dogan Z, Kanak EG, Atli G, Canli M (2015). Effects of heavy metals (Cd, Cu, Cr, Pb, Zn) on fish glutathione metabolism. Environ. Sci. Pollut. Res..

[62] Awoyemi MO, Bawa-Allah AK, Otitoloju AA (2014). Accumulation and anti-oxidant enzymes as biomarkers of heavy metal exposure in *Clarias gariepinus* and *Oreochromis niloticus*. Appl. Ecol. Environ. Sci..

[63] Baysoy E (2012). The effects of increased freshwater salinity in the biodisponibility of metals (Cr, Pb) and effects on antioxidant systems of *Oreochromis niloticus*. Ecotoxicol. Environ. Saf..

[64] Adeyeye EI, Akinyugha NJ, Fesobi ME, Tenabe VO (1996). Determination of some metals in *Clarias gariepinus* (Cuvier and Vallenciennes), *Cyprinus carpio* (L.) and *Oreochromis niloticus* (L.) fishes in a polyculture fresh water pond and their environments. Aquaculture.

[65] Arabi M, Alaeddini MA (2005). Metal-ion-mediated oxidative stress in the gill homogenate of rainbow trout (*Oncorhynchus mykiss*): Antioxidant potential of manganese, selenium, and albumin. Biol. Trace Elem. Res..

[66] Becker JS, Füllner K, Seeling UD, Fornalczyk G, Kuhn AJ (2008). Measuring magnesium, calcium and potassium isotope ratios using ICP-QMS with an octopole collision cell in tracer studies of nutrient uptake and translocation in plants. Anal. Bioanal. Chem..

[67] Canli M, Atli G (2003). The relationships between heavy metal (Cd, Cr, Cu, Fe, Pb, Zn) levels and the size of six Mediterranean fish species. Environ. Pollut..

[68] Wu X (2016). A review of toxicity and mechanisms of individual and mixtures of heavy metals in the environment. Environ. Sci. Pollut. Res..

[69] Warren MJ, Cooper JB, Wood SP, Shoolingin-Jordan PM (1998). Lead poisoning, haem synthesis and 5-aminolaevulinic acid dehydratase. Trends Biochem. Sci..

[70] Li C (2011). Lead exposure suppressed ALAD transcription by increasing methylation level of the promoter CpG islands. Toxicol. Lett..

[71] Cigerci İH, Konuk M, Kutlu M (2010). Lead toxicity and biochemical characterization of δ-ALAD on endemic prawn, *Palaemonetes turcorum*. Ekoloji.

[72] Lombardi PE, Peri SI, VerrengiaGuerrero NR (2010). ALA-D and ALA-D reactivated as biomarkers of lead contamination in the fish *Prochilodus lineatus*. Ecotoxicol. Environ. Saf..

[73] Konuk, M., Hakk, B. & Elif, S. ALAD (Δ-aminolevulinic acid dehydratase) as biosensor for Pb contamination. in *Intelligent and Biosensors*. 10.5772/7162 (InTech, 2010).

[74] Aisemberg J, Nahabedian DE, Wider EA, Verrengia Guerrero NR (2005). Comparative study on two freshwater invertebrates for monitoring environmental lead exposure. Toxicology.

[75] Hermes-Lima M (1995). How do Ca^2+^ and 5-aminolevulinic acid-derived oxyradicals promote injury to isolated mitochondria?. Free Radic. Biol. Med..

[76] Bechara EJ (1996). Oxidative stress in acute intermittent porphyria and lead poisoning may be triggered by 5-aminolevulinic acid. Braz. J. Med. Biol. Res. (Rev. Bras. Pesqui. Med. Biol.).

[77] Douki T (1998). DNA alkylation by 4,5-dioxovaleric acid, the final oxidation product of 5-aminolevulinic acid. Chem. Res. Toxicol..

[78] Douki T (1998). Hydroxyl radicals are involved in the oxidation of isolated and cellular DNA bases by 5-aminolevulinic acid. FEBS Lett..

[79] Quintanilla-Vega B (2000). Lead effects on protamine-DNA binding. Am. J. Ind. Med..

[80] Jaishankar M, Tseten T, Anbalagan N, Mathew BB, Beeregowda KN (2014). Toxicity, mechanism and health effects of some heavy metals. Interdiscip. Toxicol..

[81] Paul N, Chakraborty S, Sengupta M (2014). Lead toxicity on non-specific immune mechanisms of freshwater fish *Channa punctatus*. Aquat. Toxicol..

[82] Soto E (2016). Laboratory-controlled challenges of Nile tilapia (*Oreochromis niloticus*) with *Streptococcus agalactiae*: Comparisons between immersion, oral, intracoelomic and intramuscular routes of infection. J. Comp. Pathol..

[83] Miyazaki T (1998). A simple method to evaluate respiratory burst activity of blood phagocytes from Japanese flounder. Fish Pathol..

[84] Lin Q (2016). An outbreak of granulomatous inflammation associated with *Francisella noatunensis* subsp. *orientalis* in farmed tilapia (*Oreochromis niloticus* × *O. aureus*) in China. Chin. J. Oceanol. Limnol..

[85] Aldoghachi MA, Azirun MS, Yusoff I, Ashraf MA (2016). Ultrastructural effects on gill tissues induced in red tilapia *Oreochromis* sp. by a waterborne lead exposure. Saudi J. Biol. Sci..

[86] Listrat A (2016). How muscle structure and composition influence meat and flesh quality. Sci. World J..

[87] Jarrar BM, Taib NT (2012). Histological and histochemical alterations in the liver induced by lead chronic toxicity. Saudi J. Biol. Sci..

[88] Alzohairy MA (2016). Therapeutics role of *Azadirachta indica* (neem) and their active constituents in diseases prevention and treatment. Evid.-Based Complement. Altern. Med..

[89] van der Nat J, van der Sluis W, de Silva KT, Labadie R (1991). Ethnopharmacognostical survey of *Azadirachta indica* A. Juss (Meliaceae). J. Ethnopharmacol..

[90] Tasanarong T, Patntirapong S, Aupaphong V (2021). The inhibitory effect of a novel neem paste against cariogenic bacteria. J. Clin. Exp. Dent..

[91] Sadeek SA, Negm NA, Hefni HHH, Wahab MMA (2015). Metal adsorption by agricultural biosorbents: Adsorption isotherm, kinetic and biosorbents chemical structures. Int. J. Biol. Macromol..

