# Supplementary figures and images for: Neem leaf powder (Azadirachta indica) mitigates oxidative stress and pathological alterations triggered by lead toxicity in Nile tilapia (Oreochromis niloticus)
Source: Sci Rep. 2023 Jun 6;13:9170. doi: 10.1038/s41598-023-36121-4 (PMC10244493; doi:10.1038/s41598-023-36121-4)

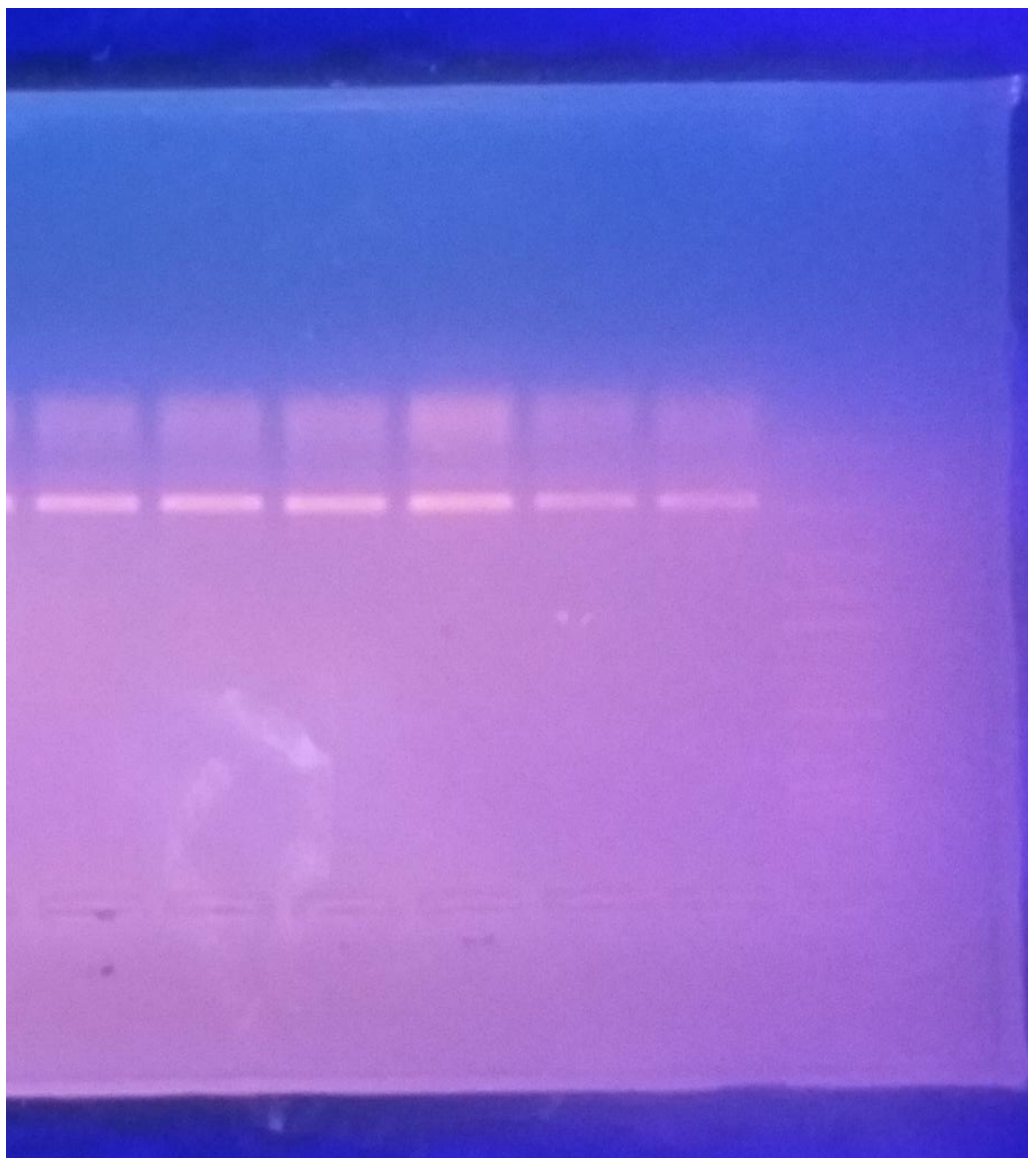

Supplement: Supplementary file 1 — Supplementary Information 1. [file 41598_2023_36121_MOESM1_ESM.pdf]

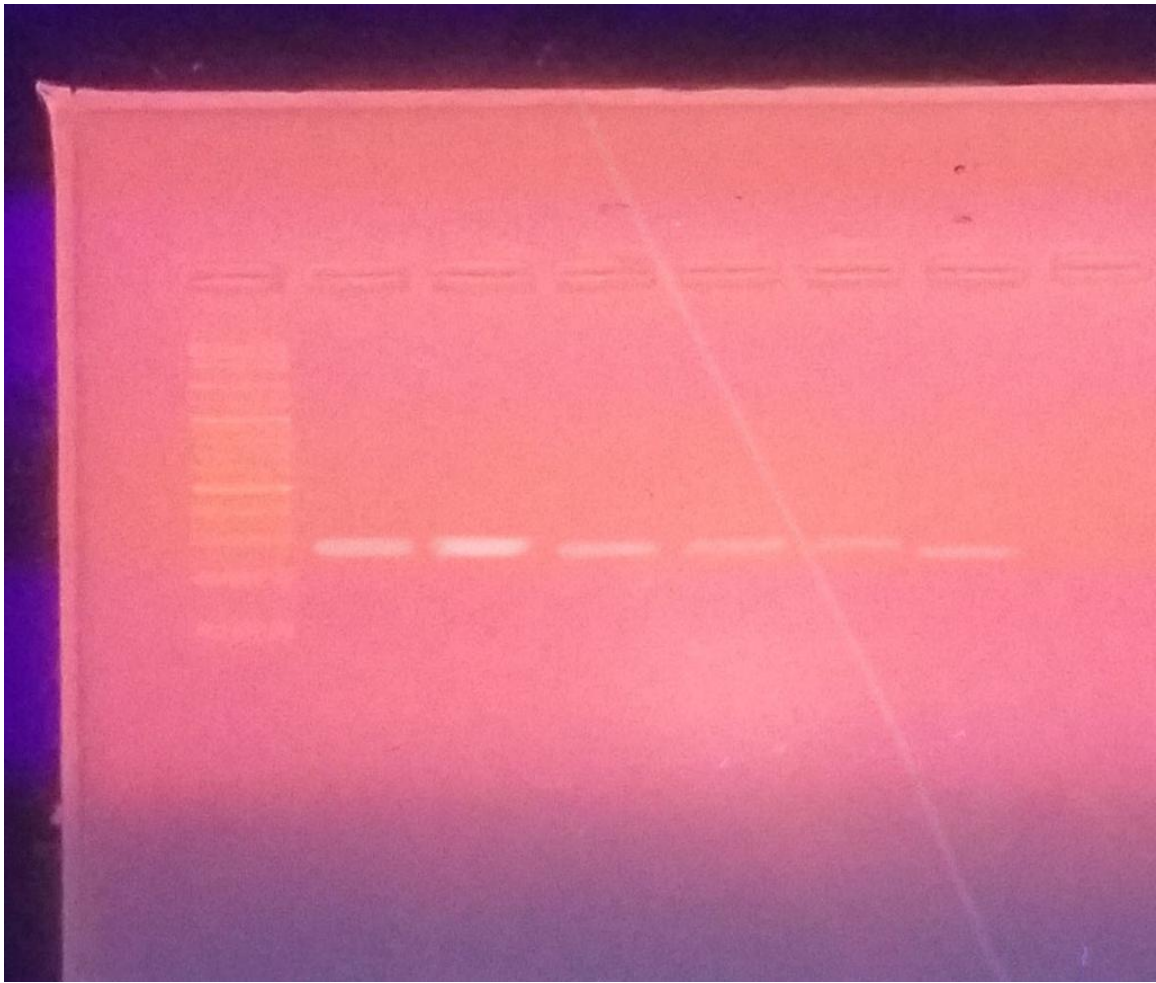

Supplement: Supplementary file 2 — Supplementary Information 2. [file 41598_2023_36121_MOESM2_ESM.pdf]

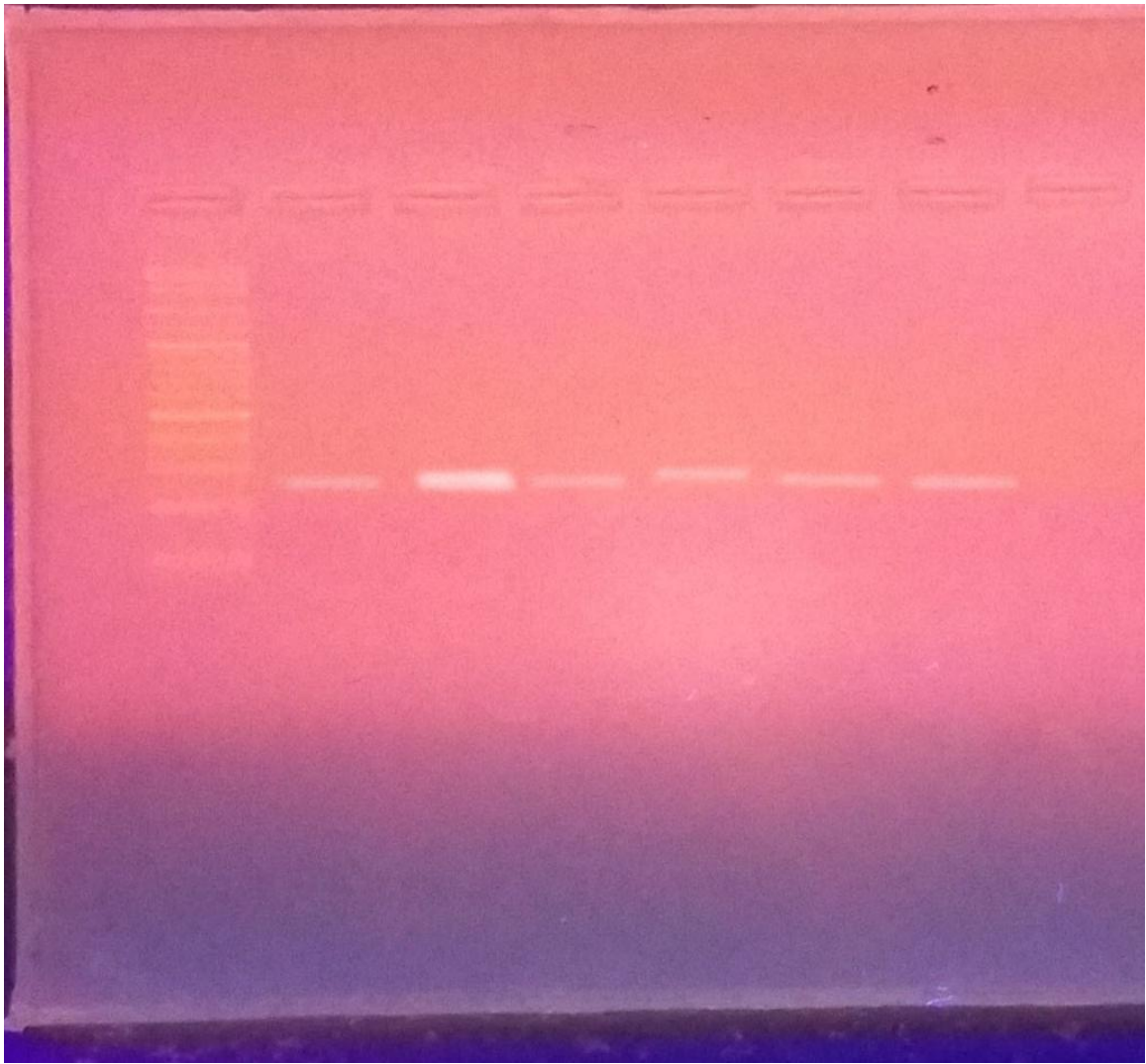

Supplement: Supplementary file 3 — Supplementary Information 3. [file 41598_2023_36121_MOESM3_ESM.pdf]

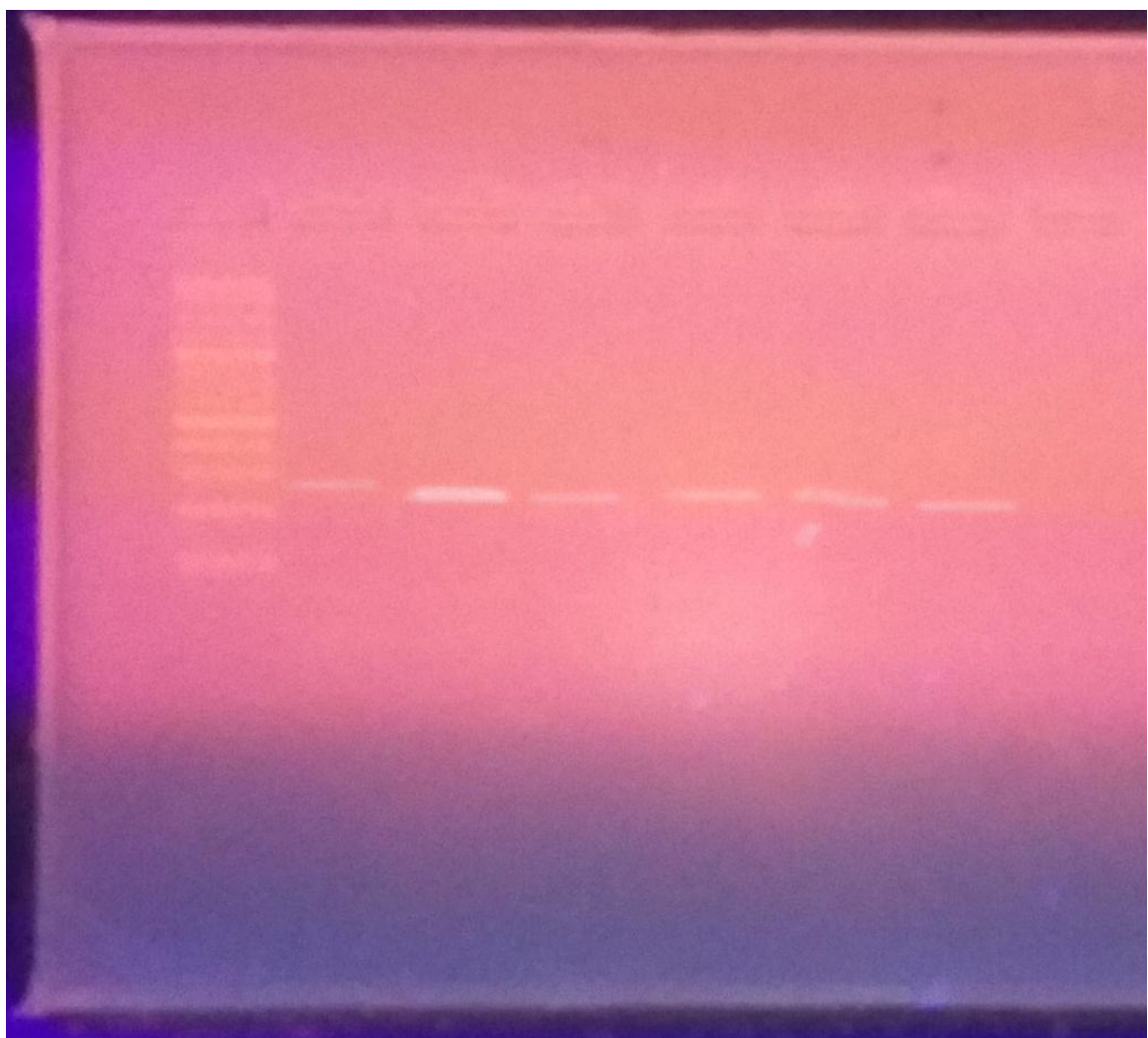

Supplement: Supplementary file 4 — Supplementary Information 4. [file 41598_2023_36121_MOESM4_ESM.pdf]
