# Supplementary material for: Neem leaf powder (Azadirachta indica) mitigates oxidative stress and pathological alterations triggered by lead toxicity in Nile tilapia (Oreochromis niloticus)
Source: Sci Rep. 2023 Jun 6;13:9170. doi: 10.1038/s41598-023-36121-4 (PMC10244493; doi:10.1038/s41598-023-36121-4)

My GC-MS Report

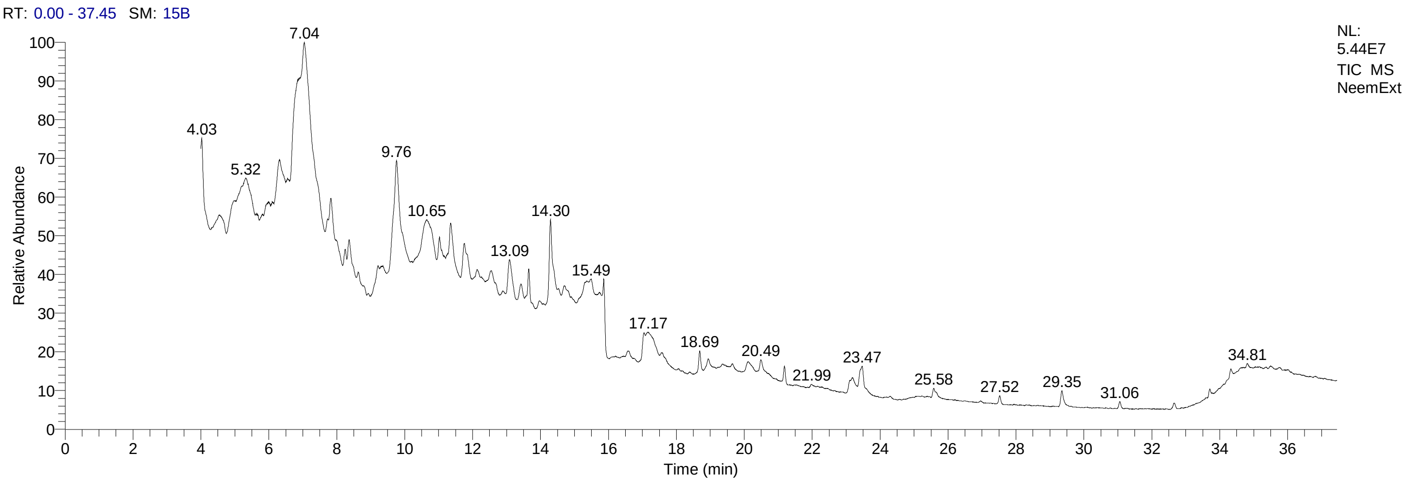

RT	Area %	Peak Area	Peak Height
5.33	6.92	72408794.93	4230064.16
6.30	3.92	40986370.13	4414488.71
6.77	5.92	61995639.07	5519867.16
7.05	6.27	65615101.27	7004025.47
7.73	0.69	7251985.63	2048888.47
7.82	3.01	31495876.43	5406730.17
8.25	1.11	11581099.32	2783388.33
8.36	3.02	31576880.76	4564702.02
8.64	0.55	5722646.64	1406703.17
9.21	1.85	19392399.24	2852042.39
9.29	1.52	15960028.56	2153599.00
9.76	9.39	98285133.25	11326294.51
10.66	7.59	79435343.72	4627702.94
11.02	1.50	15661207.89	3361944.92
11.36	2.72	28494656.19	5118634.67
11.75	3.99	41815950.47	4911118.40
12.15	1.24	12938628.50	1457246.52
12.55	1.30	13619388.66	1906271.73
13.09	3.56	37297580.93	4929969.80
13.42	1.16	12111950.99	2110576.90
13.66	1.93	20151073.02	5664723.14
14.29	6.01	62910920.88	11132434.34
14.71	1.29	13468660.60	1573180.25
15.30	0.66	6943552.72	1739612.73
15.35	0.97	10120410.99	1880928.85
15.48	1.18	12373953.79	2110144.81
15.87	6.48	67816918.86	9171972.89
17.04	1.69	17720420.03	3767385.79
17.16	4.78	50032743.08	3454688.93
18.69	1.18	12359290.24	3312268.42
18.94	0.72	7561879.17	1374219.75
20.49	0.74	7729782.44	1675375.54
21.18	0.94	9854324.73	2783289.85
23.18	0.78	8145697.88	1450321.53
23.47	1.92	20058982.68	3029009.65
25.58	0.51	5313160.21	1364779.12
29.35	1.01	10567705.38	2194975.97

My GC-MS Report

NeemExt #398 RT: 5.33 AV: 1 NL: 2.71E6
T: + c EI Full ms [50.000-750.000]

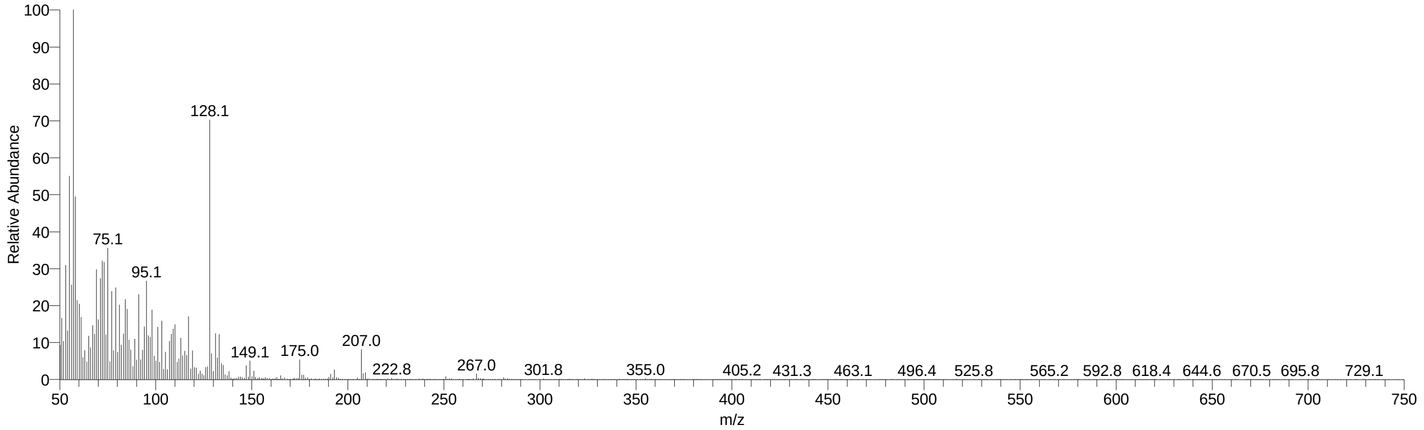

RT	Compound Name	Area %	MF	Molecular Formula	Molecular Weight	Cas #	Library
5.33	9,12,15-OCTADECATRIENOIC ACID, 2-[(TRIMETHYLSILYL)OXY]-1-[[(TRIMETHYLSILYL)OXY]METHYL]ETHYL ESTER, (Z,Z,Z)-	6.92	706	C27H52O4Si2	496	55521-23-8	WileyRegistry8e
5.33	ETHANIMIDOTHIOIC ACID, 2-(DIMETHYLAMINO)-N-[[(METHYLAMINO)CARBONYL]OXY]-2-OXO-, METHYL ESTER	6.92	725	C7H13N3O3S	219	23135-22-0	WileyRegistry8e
5.33	2,5-PIPERAZINEDIONE, 3-[(4-HYDROXYPHENYL)METHYL]-6-METHYL-	6.92	670	C12H14N2O3	234	21754-26-7	WileyRegistry8e
5.33	1-Pyridinepropanoic acid, hexahydro-3-(hydroxymethyl)-	6.92	671	C9H17NO3	187	NA	mainlib
5.33	O,N-PERMETHYLATED AC-ALA-HIS	6.92	705	C15H24N4O4	324	NA	WileyRegistry8e

Compound Structure	Hit Spectrum

Formula C27H52O4Si2, MW 496, CAS# 55521-23-8, Entry# 284835

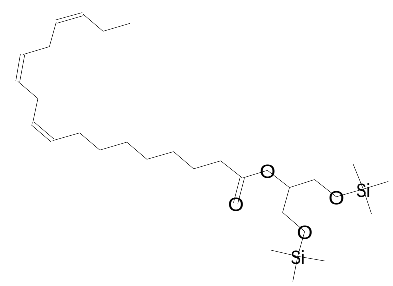

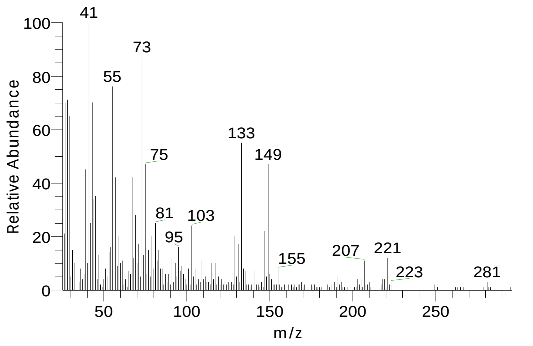

Formula C7H13N3O3S, MW 219, CAS# 23135-22-0, Entry# 105150

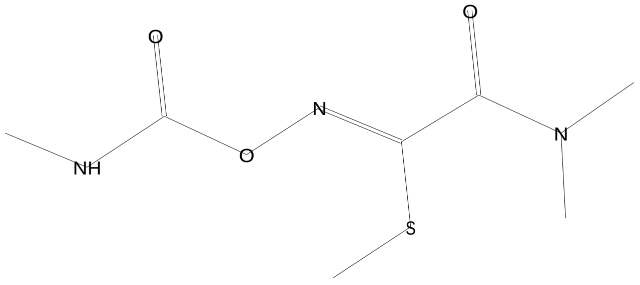

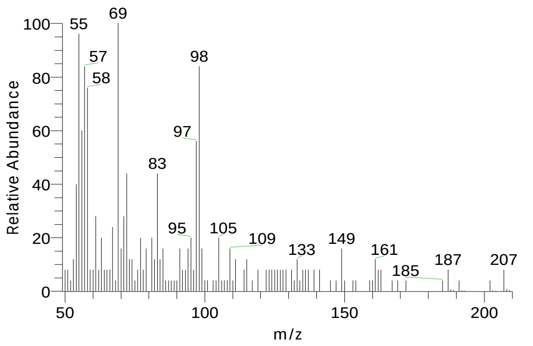

My GC-MS Report

Compound Structure | Hit Spectrum

2,5-PIPERAZINEDIONE, 3-[(4-HYDROXYPHENYL)METHYL]-6-METHYL-
Formula C12H14N2O3, MW 234, CAS# 21754-26-7, Entry# 122333
3-(4-HYDROXYBENZYL)-6-METHYL-2,5-PIPERAZINEDIONE #

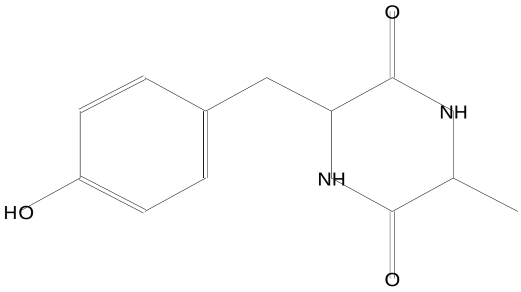

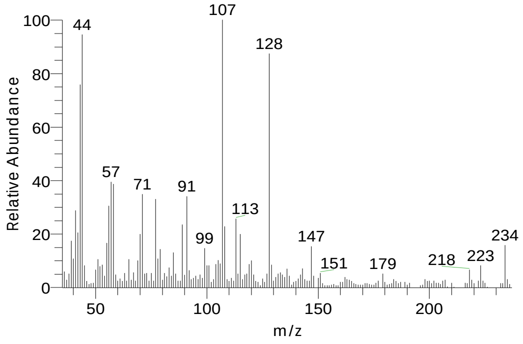

1-Pyridinepropanoic acid, hexahydro-3-(hydroxymethyl)-
Formula C9H17NO3, MW 187, CAS# NA, Entry# 112266
$:28APRMLBQVNDDKNP-UHFFFAOYSA-N

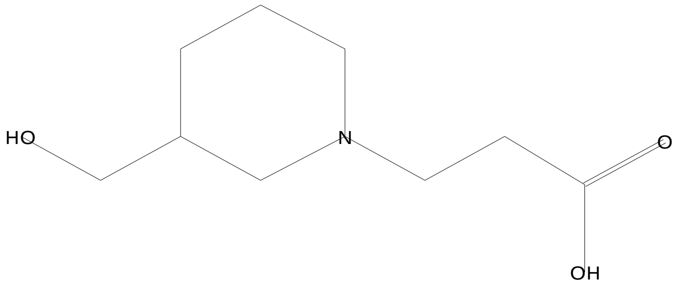

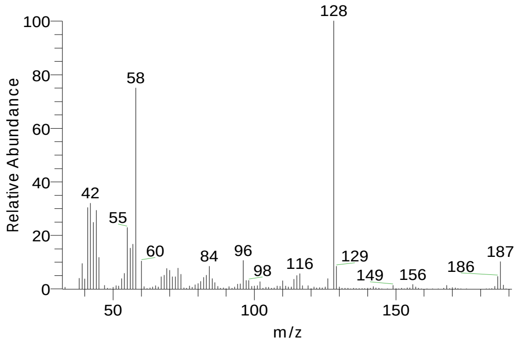

O,N-PERMETHYLATED AC-ALA-HIS
Formula C15H24N4O4, MW 324, CAS# NA, Entry# 209544

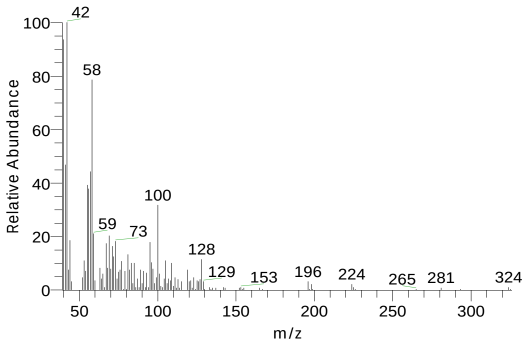

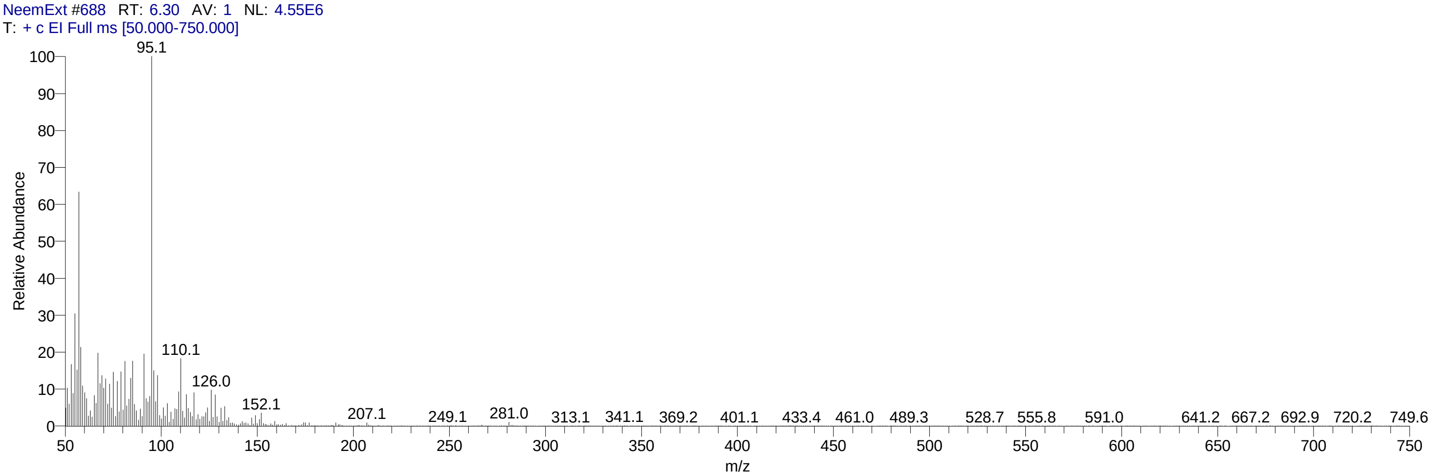

RT	Compound Name	Area %	MF	Molecular Formula	Molecular Weight	Cas #	Library
6.30	10-Methyl-E-11-tridecen-1-ol propionate	3.92	680	C17H32O2	268	NA	mainlib
6.30	TRIDECANEDIAL	3.92	678	C13H24O2	212	63521-76-6	WileyRegistry8e

My GC-MS Report

RT	Compound Name	Area %	MF	Molecular Formula	Molecular Weight	Cas #	Library
6.30	Tridecanedial	3.92	676	C13H24O2	212	63521-76-6	mainlib
6.30	TRIDECANEDIAL	3.92	676	C13H24O2	212	63521-76-6	WileyRegistry8e
6.30	cis-2-Ethyl-2-hexen-1-ol	3.92	747	C8H16O	128	NA	mainlib

Compound Structure | Hit Spectrum

10-Methyl-E-11-tridecen-1-ol propionate
Formula C17H32O2, MW 268, CAS# NA, Entry# 24675
(11E)-10-Methyl-11-tridecenyl propionate #

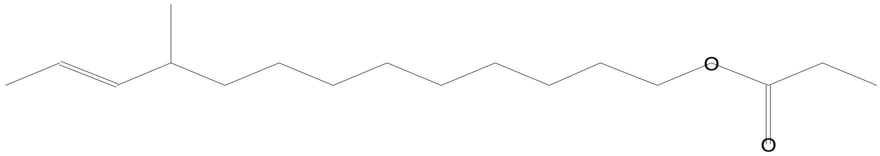

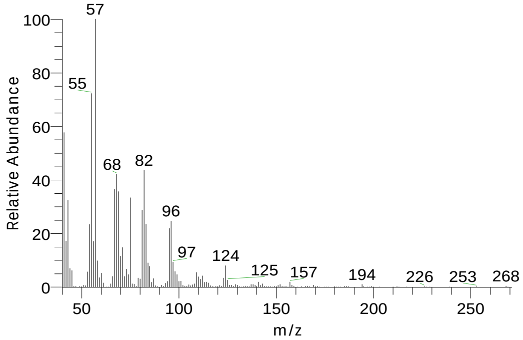

TRIDECANEDIAL
Formula C13H24O2, MW 212, CAS# 63521-76-6, Entry# 364130

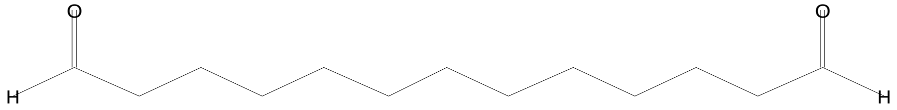

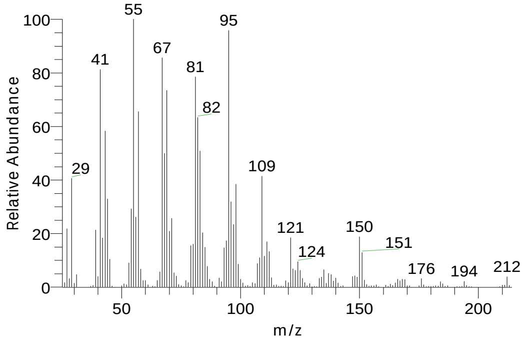

Tridecanedial
Formula C13H24O2, MW 212, CAS# 63521-76-6, Entry# 21023
$:28LUUDAJQADHSYHX-UHFFFAOYSA-N

SI 665, RSI 676, mainlib, Entry# 21023, CAS# 63521-76-6, Tridecanedial

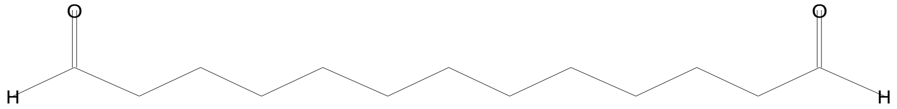

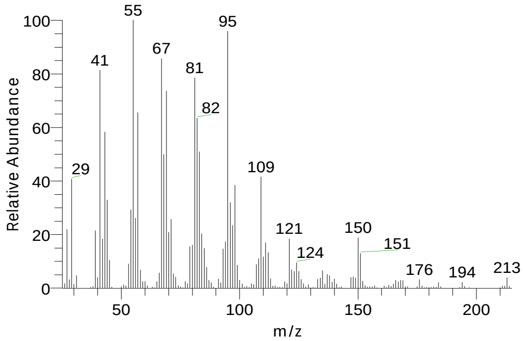

TRIDECANEDIAL
Formula C13H24O2, MW 212, CAS# 63521-76-6, Entry# 366508

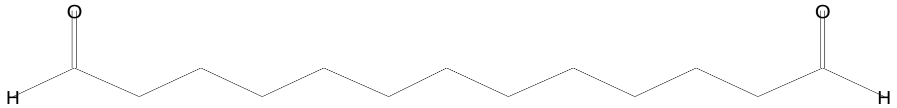

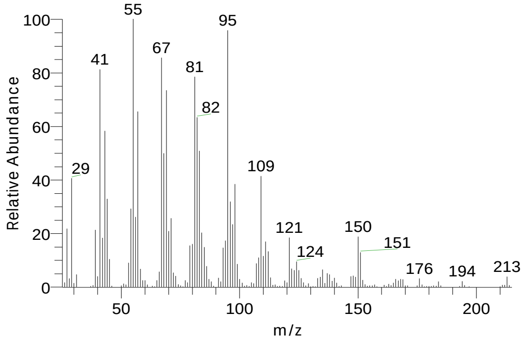

My GC-MS Report

Compound Structure | Hit Spectrum

cis-2-Ethyl-2-hexen-1-ol
Formula C8H16O, MW 128, CAS# NA, Entry# 2031
(2Z)-2-Ethyl-2-hexen-1-ol #

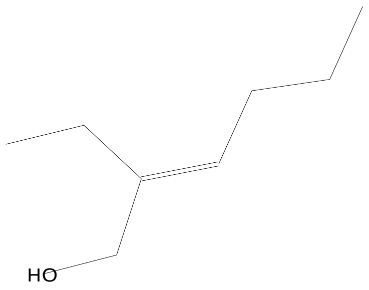

SI 663, RSI 747, mainlib, Entry# 2031, CAS# NA, cis-2-Ethyl-2-hexen-1-ol

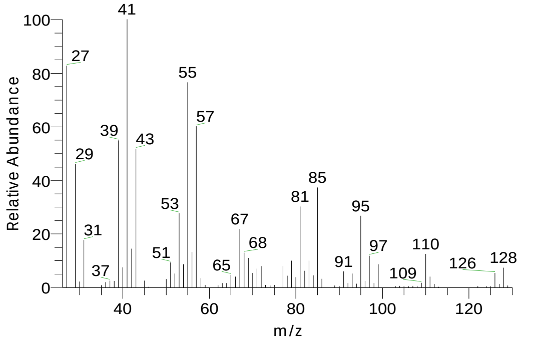

NeemExt #827 RT: 6.77 AV: 1 NL: 4.94E6
T: + c EI Full ms [50.000-750.000]

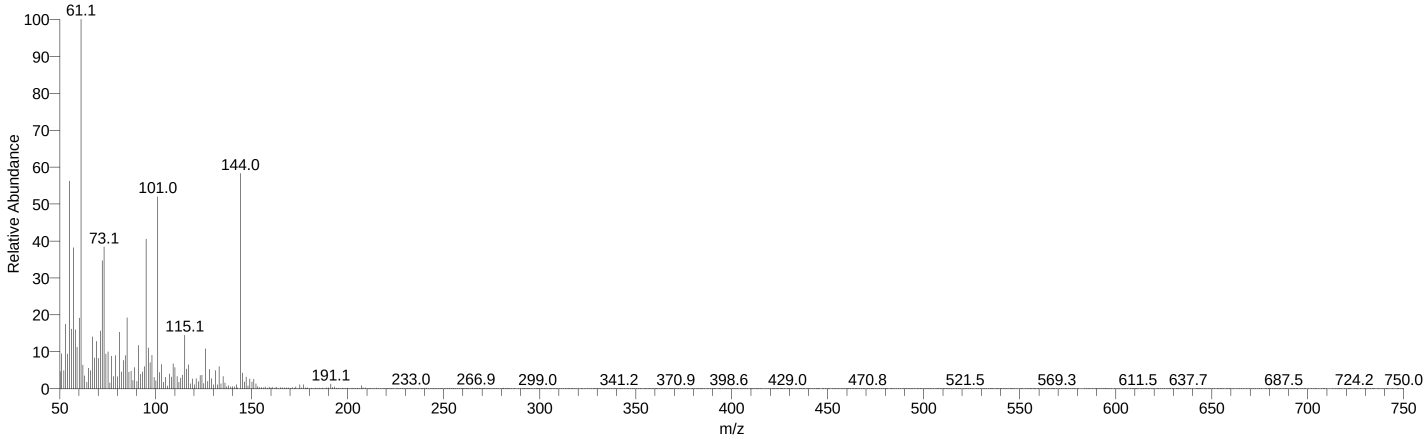

RT	Compound Name	Area %	MF	Molecular Formula	Molecular Weight	Cas #	Library
6.77	2-AMINO-5-GUANIDINO-PENTANOIC ACID	5.92	685	C6H14N4O2	174	74-79-3	WileyRegistry8e
6.77	Stevioside	5.92	680	C38H60O18	804	77-05-4	mainlib
6.77	6-Acetyl-á-d-mannose	5.92	706	C8H14O7	222	NA	mainlib
6.77	l-Gala-l-ido-octonic lactone	5.92	654	C8H14O8	238	NA	mainlib
6.77	d-Gala-l-ido-octonic amide	5.92	661	C8H17NO8	255	33535-07-8	mainlib

Compound Structure | Hit Spectrum

2-AMINO-5-GUANIDINO-PENTANOIC ACID
Formula C6H14N4O2, MW 174, CAS# 74-79-3, Entry# 56662
(L)-ARGININE

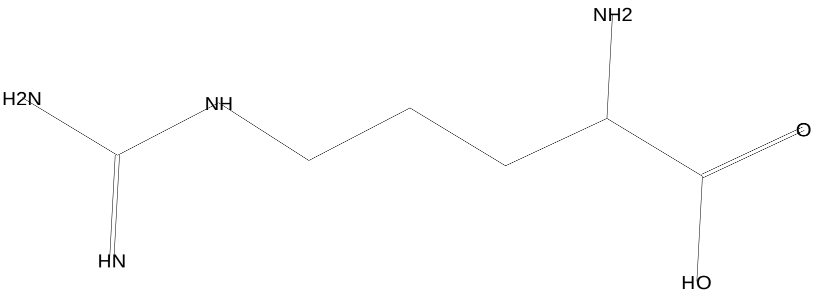

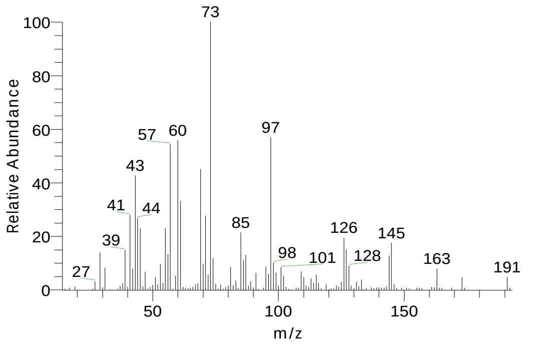

My GC-MS Report

Compound Structure | Hit Spectrum

Stevioside
Formula C38H60O18, MW 804, CAS# 77-05-4, Entry# 30863
1-O-(13-[(2-O-Hexopyranosylhexopyranosyl)oxy]-18-oxokaur-16-en-18-yl)hexopyranose #

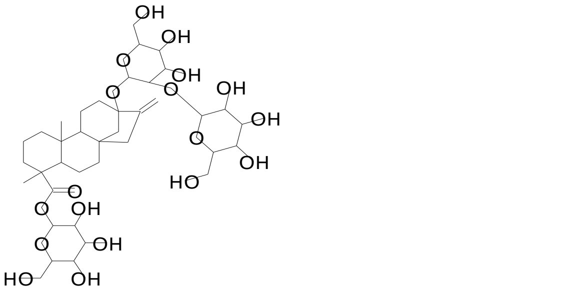

SI 664, RSI 680, mainlib, Entry# 30863, CAS# 77-05-4, Stevioside

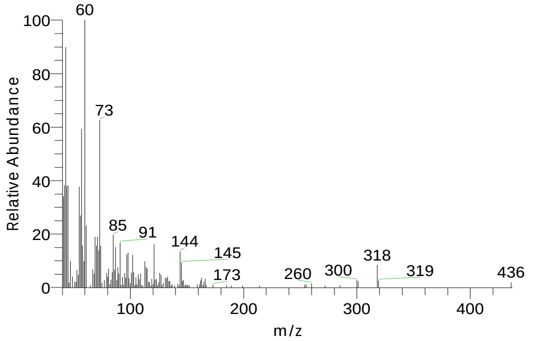

6-Acetyl-á-d-mannose
Formula C8H14O7, MW 222, CAS# NA, Entry# 5869
6-O-Acetylhexopyranose #

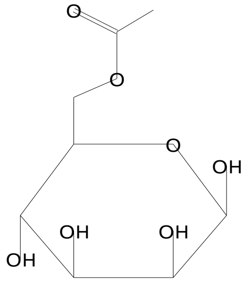

SI 662, RSI 706, mainlib, Entry# 5869, CAS# NA, 6-Acetyl-á-d-mannose

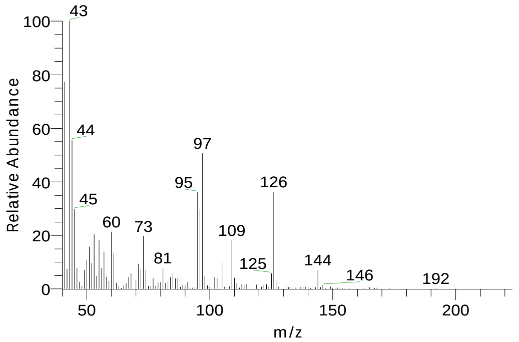

l-Gala-l-ido-octonic lactone
Formula C8H14O8, MW 238, CAS# NA, Entry# 40587
$:28NUYDBDGECBIUPJ-UHFFFAOYSA-N

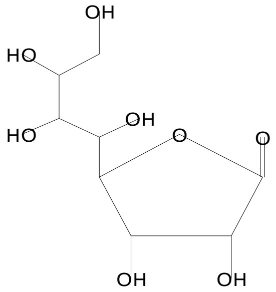

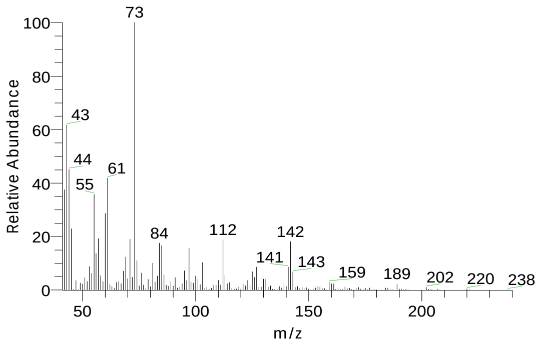

d-Gala-l-ido-octonic amide
Formula C8H17NO8, MW 255, CAS# 33535-07-8, Entry# 40689
$:28JUCFNBKGLVDIFE-UHFFFAOYSA-N

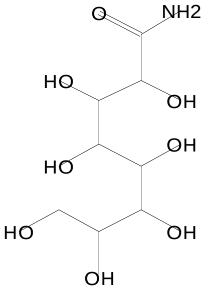

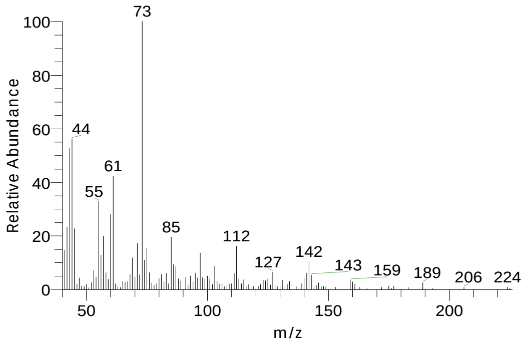

My GC-MS Report

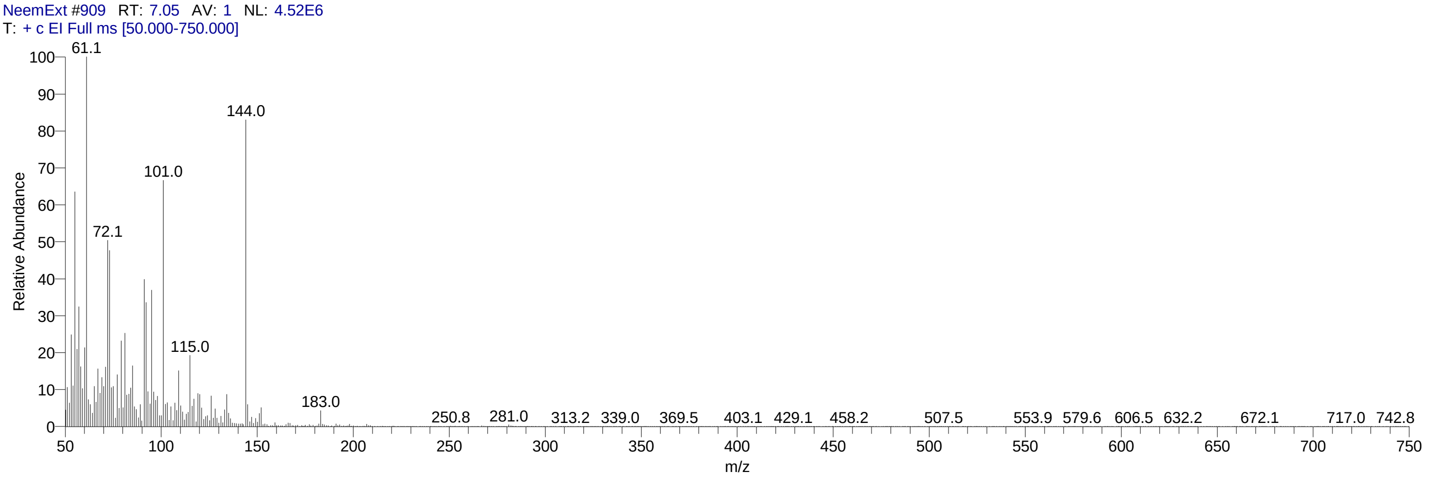

RT	Compound Name	Area %	MF	Molecular Formula	Molecular Weight	Cas #	Library
7.05	d-Gala-l-ido-octonic amide	6.27	642	C8H17NO8	255	33535-07-8	mainlib
7.05	l-Gala-l-ido-octonic lactone	6.27	626	C8H14O8	238	NA	mainlib
7.05	d-Lyxo-d-manno-nononic-1,4-lactone	6.27	623	C9H16O9	268	3080-49-7	mainlib
7.05	1,3-Dioxolane, 4-[[(2-methoxy-4-octadecenyl)oxy]methyl]-2,2-dimethyl-	6.27	707	C25H48O4	412	16725-41-0	mainlib
7.05	1,3-DIOXOLANE, 4-[[(2-METHOXY-4-OCTADECENYL)OXY]METHYL]-2,2-DIMETHYL-	6.27	707	C25H48O4	412	16725-41-0	WileyRegistry8e

Compound Structure | Hit Spectrum

d-Gala-l-ido-octonic amide
Formula C8H17NO8, MW 255, CAS# 33535-07-8, Entry# 40689
$:28JUCFNBKGLVDIFE-UHFFFAOYSA-N

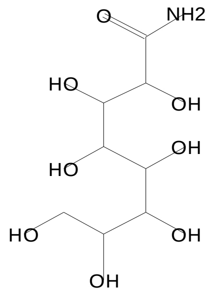

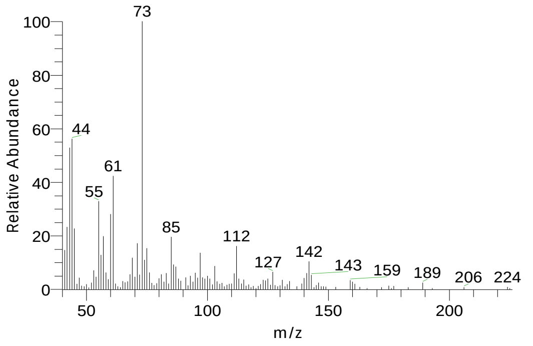

l-Gala-l-ido-octonic lactone
Formula C8H14O8, MW 238, CAS# NA, Entry# 40587
$:28NUYDBDGECBIUPJ-UHFFFAOYSA-N

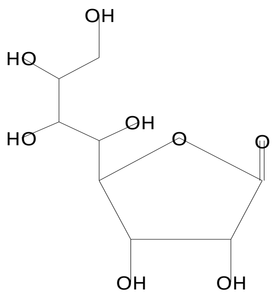

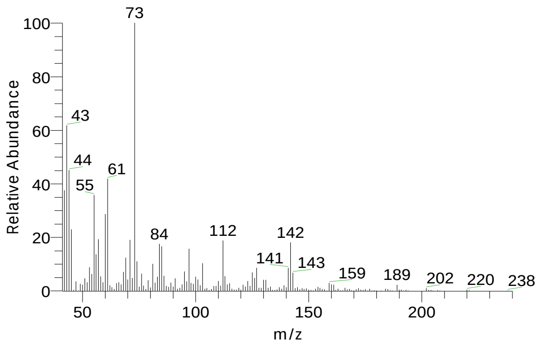

My GC-MS Report

Compound Structure | Hit Spectrum

d-Lyxo-d-manno-nononic-1,4-lactone
Formula C9H16O9, MW 268, CAS# 3080-49-7, Entry# 4535
$:28PWOUTIZYJMAJCL-UHFFFAOYSA-N

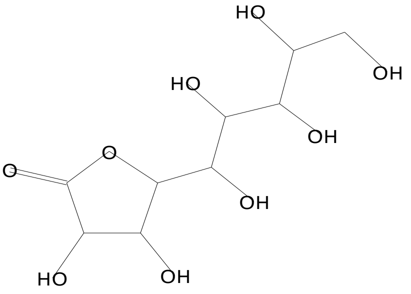

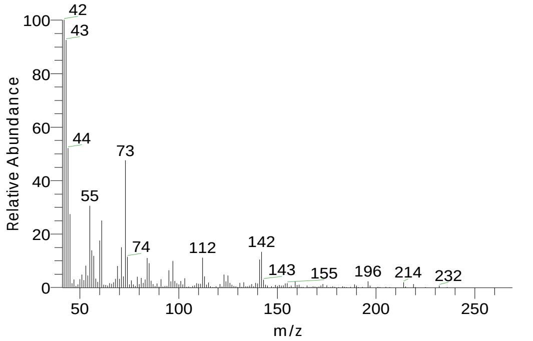

1,3-Dioxolane, 4-[[(2-methoxy-4-octadecenyl)oxy]methyl]-2,2-dimethyl-
Formula C25H48O4, MW 412, CAS# 16725-41-0, Entry# 38578
4-(([(4E)-2-Methoxy-4-octadecenyl]oxy)methyl)-2,2-dimethyl-1,3-dioxolane #

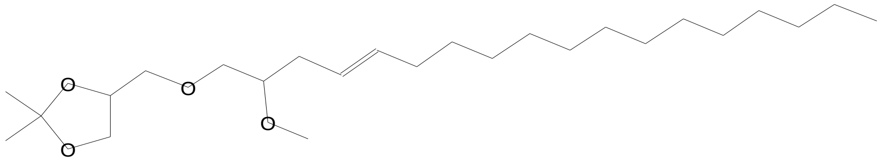

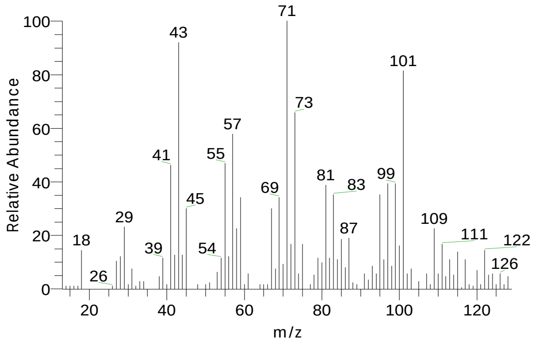

1,3-DIOXOLANE, 4-[[(2-METHOXY-4-OCTADECENYL)OXY]METHYL]-2,2-DIMETHYL-
Formula C25H48O4, MW 412, CAS# 16725-41-0, Entry# 261143
4-(([(4E)-2-METHOXY-4-OCTADECENYL]OXY)METHYL)-2,2-DIMETHYL-1,3-DIOXOLANE #

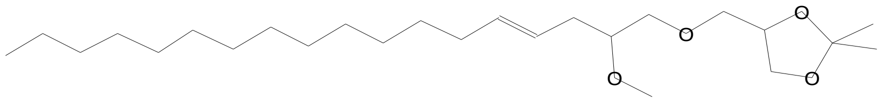

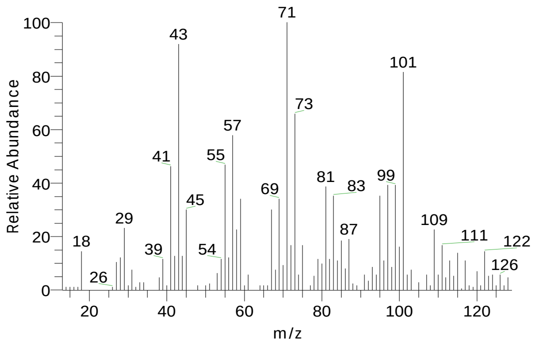

NeemExt #1114 RT: 7.73 AV: 1 NL: 3.04E6
T: + c EI Full ms [50.000-750.000]

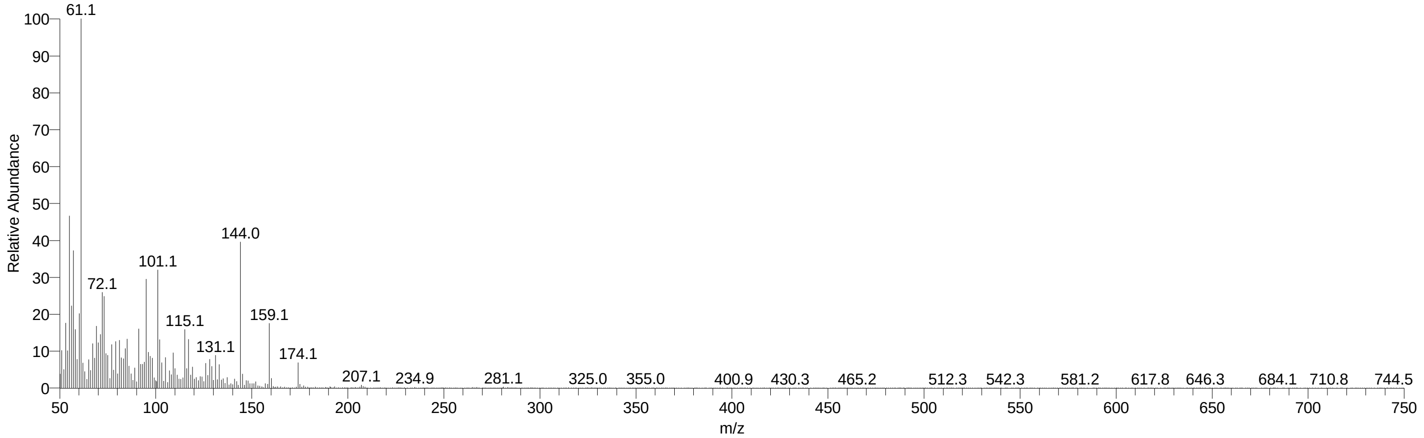

RT	Compound Name	Area %	MF	Molecular Formula	Molecular Weight	Cas #	Library
7.73	9,12,15-Octadecatrienoic acid, 2-(acetyloxy)-1-[(acetyloxy)methyl]ethyl ester, (Z,Z,Z)-	0.69	719	C25H40O6	436	55320-01-9	mainlib

My GC-MS Report

RT	Compound Name	Area %	MF	Molecular Formula	Molecular Weight	Cas #	Library
7.73	9,12,15-OCTADECATRIENOIC ACID, 2-(ACETYLOXY)-1-[(ACETYLOXY)METHYL]ETHYL ESTER, (Z,Z,Z)-	0.69	719	C25H40O6	436	55320-01-9	WileyRegistry8e
7.73	D-glycero-D-gulo-Heptonic acid, ë-lactone	0.69	675	C7H12O7	208	3063-04-5	mainlib
7.73	Curan-17-oic acid, 19,20-dihydroxy-, methyl ester, (19S)-	0.69	639	C20H26N2O4	358	2111-90-2	mainlib
7.73	CURAN-17-OIC ACID, 19,20-DIHYDROXY-, METHYL ESTER, (19S)-	0.69	638	C20H26N2O4	358	2111-90-2	WileyRegistry8e

Compound Structure | Hit Spectrum

9,12,15-Octadecatrienoic acid, 2-(acetyloxy)-1-[(acetyloxy)methyl]ethyl ester, (Z,Z,Z)-
Formula C25H40O6, MW 436, CAS# 55320-01-9, Entry# 7158
2-(Acetyloxy)-1-[(acetyloxy)methyl]ethyl (9E,12E,15E)-9,12,15-octadecatrienoate #

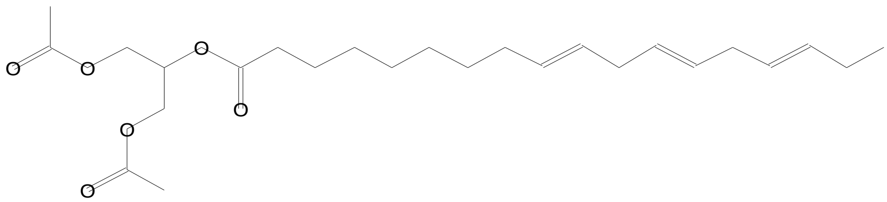

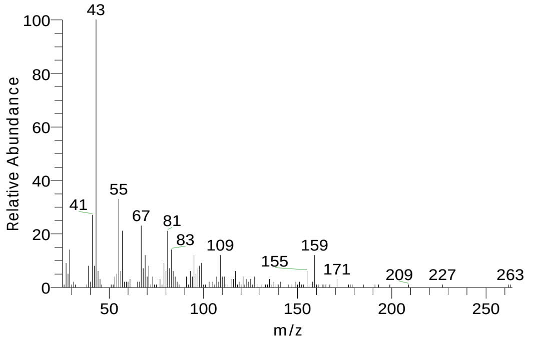

Formula C25H40O6, MW 436, CAS# 55320-01-9, Entry# 270145
2-(ACETYLOXY)-1-[(ACETYLOXY)METHYL]ETHYL (9E,12E,15E)-9,12,15-OCTADECATRIENOATE #

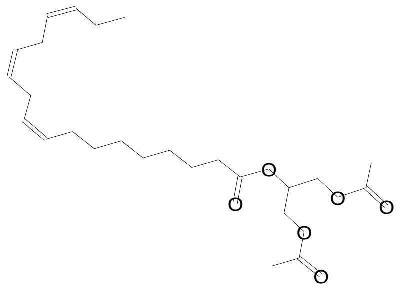

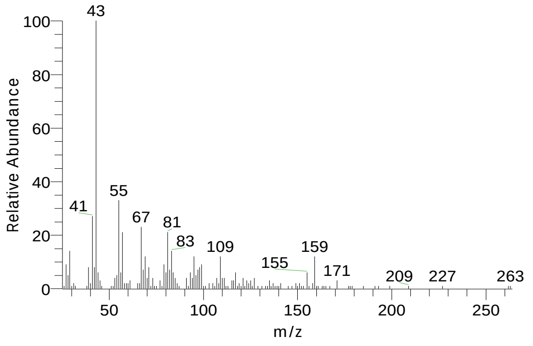

D-glycero-D-gulo-Heptonic acid, ë-lactone
Formula C7H12O7, MW 208, CAS# 3063-04-5, Entry# 40637
$:28VIVCRCODGMFTFY-UHFFFAOYSA-N

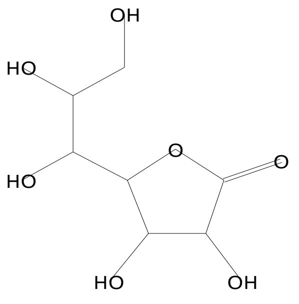

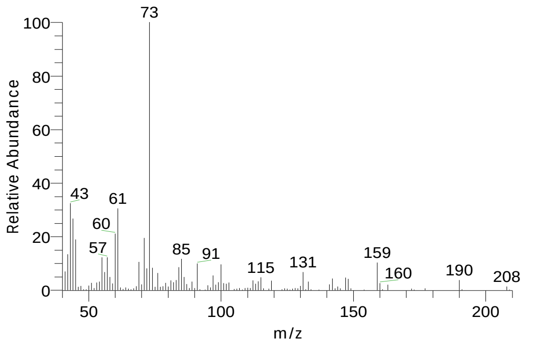

My GC-MS Report

Compound Structure | Hit Spectrum

Curan-17-oic acid, 19,20-dihydroxy-, methyl ester, (19S)-
Formula C20H26N2O4, MW 358, CAS# 2111-90-2, Entry# 26889
Curan-17-oic acid, 19,20-dihydroxy-, methyl ester, (2à,16.xi.,19S)-

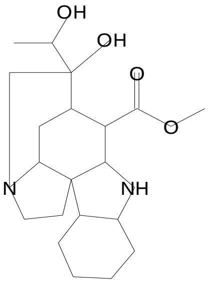

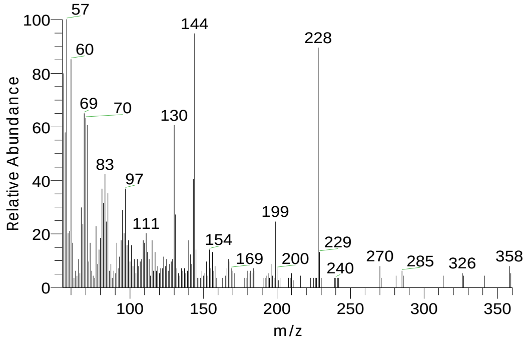

CURAN-17-OIC ACID, 19,20-DIHYDROXY-, METHYL ESTER, (19S)-
Formula C20H26N2O4, MW 358, CAS# 2111-90-2, Entry# 233681
METHYL 19,20-DIHYDROXYCURAN-17-OATE #

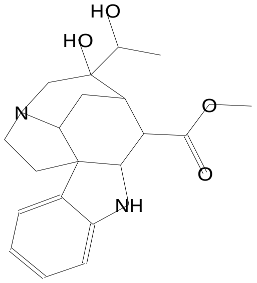

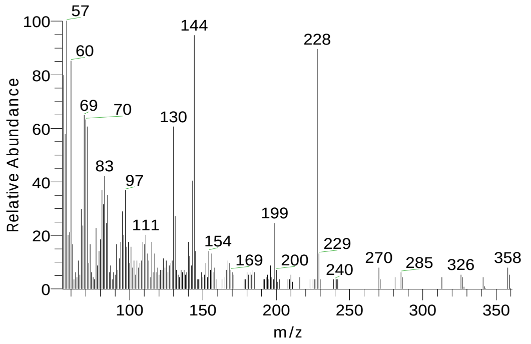

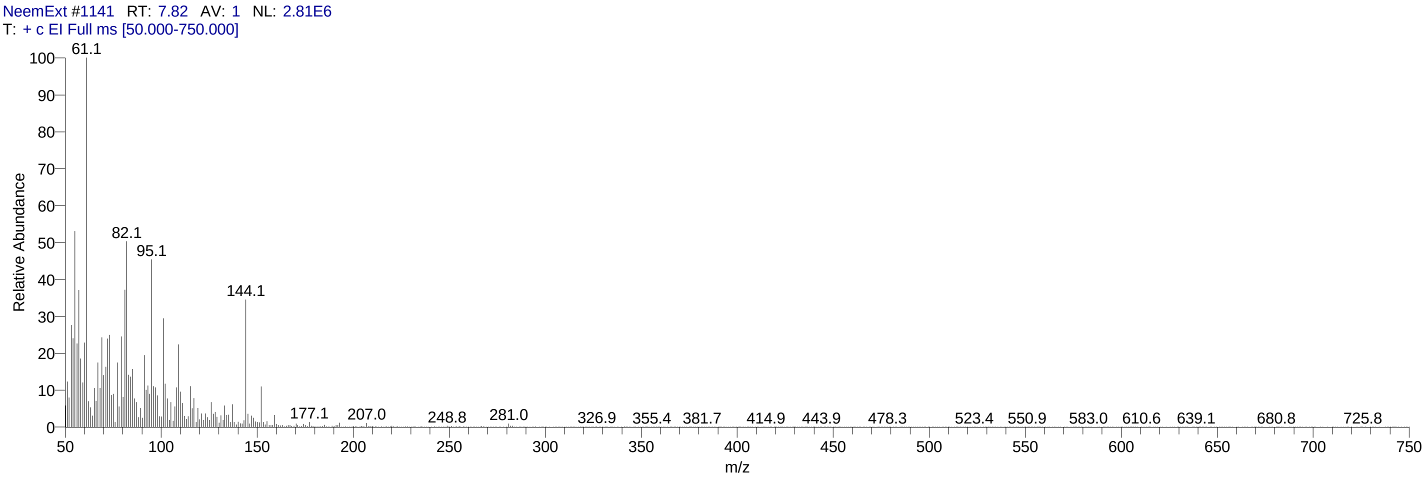

RT	Compound Name	Area %	MF	Molecular Formula	Molecular Weight	Cas #	Library
7.82	17-Octadecynoic acid	3.01	695	C18H32O2	280	34450-18-5	mainlib
7.82	TRIDECANEDIAL	3.01	691	C13H24O2	212	63521-76-6	WileyRegistry8e
7.82	Ethanol, 2-(9,12-octadecadienyloxy)-, (Z,Z)-	3.01	656	C20H38O2	310	17367-08-7	mainlib
7.82	ETHANOL, 2-(9,12-OCTADECADIENYLOXY)-, (Z,Z)-	3.01	656	C20H38O2	310	17367-08-7	WileyRegistry8e
7.82	Tridecanedial	3.01	686	C13H24O2	212	63521-76-6	mainlib

My GC-MS Report

Compound Structure | Hit Spectrum

17-Octadecynoic acid
Formula C18H32O2, MW 280, CAS# 34450-18-5, Entry# 20510
$:28DZIILFGADWDKMF-UHFFFAOYSA-N

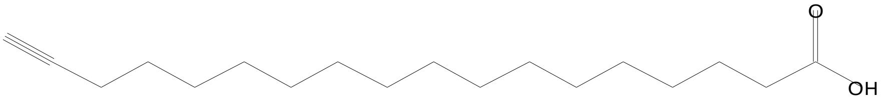

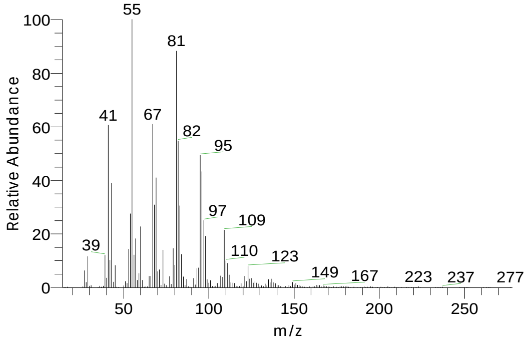

TRIDECANEDIAL
Formula C13H24O2, MW 212, CAS# 63521-76-6, Entry# 364130

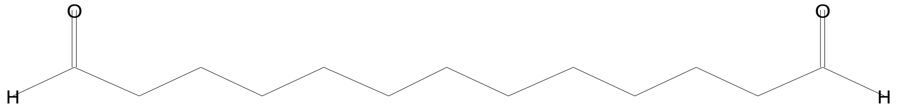

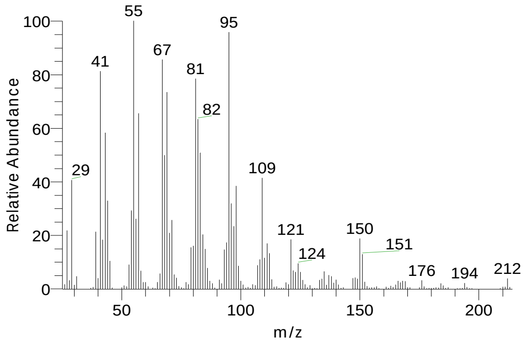

Ethanol, 2-(9,12-octadecadienyloxy)-, (Z,Z)-
Formula C20H38O2, MW 310, CAS# 17367-08-7, Entry# 32811
2-cis,cis-9,12-Octadecadienyloxyethanol

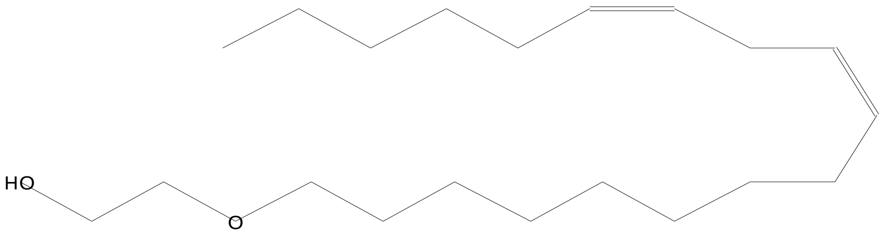

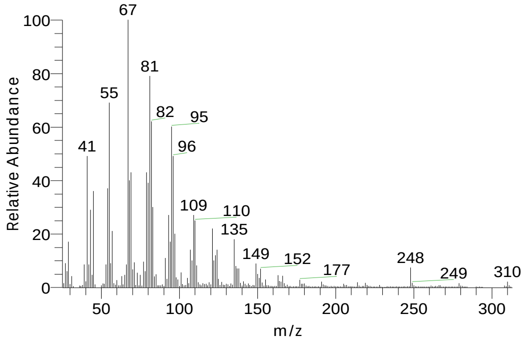

ETHANOL, 2-(9,12-OCTADECADIENYLOXY)-, (Z,Z)-
Formula C20H38O2, MW 310, CAS# 17367-08-7, Entry# 198568
2-[(9Z,12Z)-9,12-OCTADECADIENYLOXY]ETHANOL #

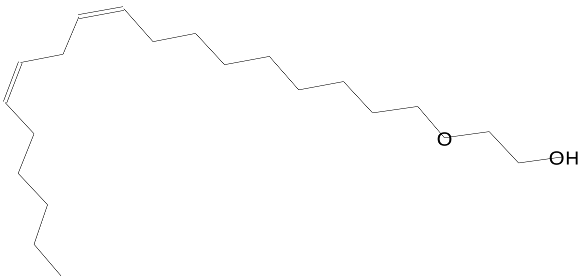

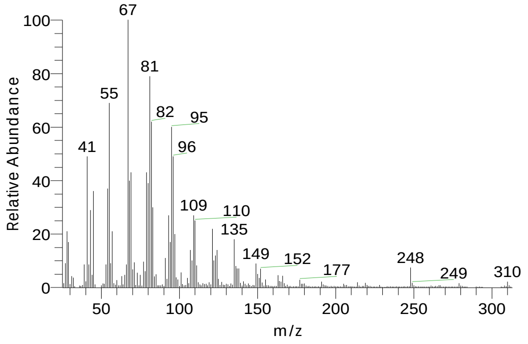

My GC-MS Report

Compound Structure

Hit Spectrum

Tridecanedial
Formula C13H24O2, MW 212, CAS# 63521-76-6, Entry# 21023
$:28LUUDAJQADHSYHX-UHFFFAOYSA-N

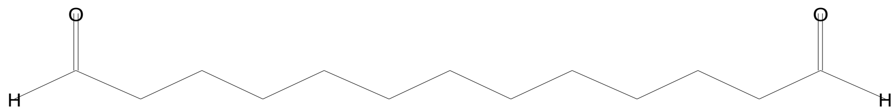

SI 655, RSI 686, mainlib, Entry# 21023, CAS# 63521-76-6, Tridecanedial

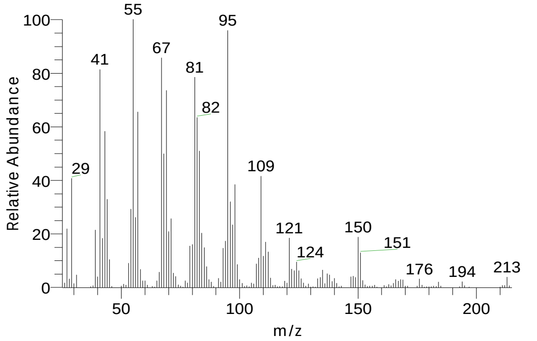

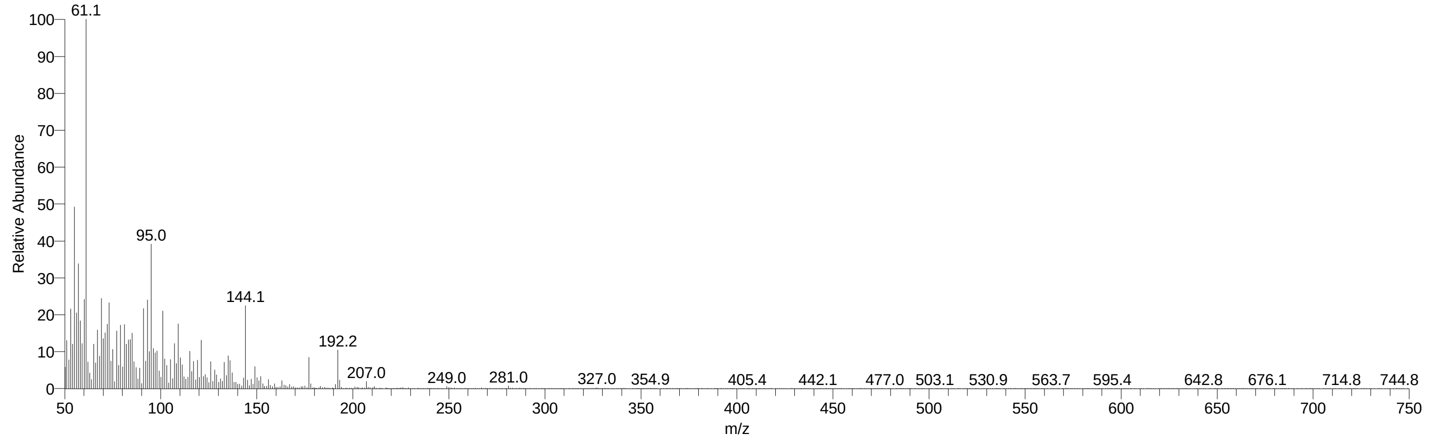

RT	Compound Name	Area %	MF	Molecular Formula	Molecular Weight	Cas #	Library
8.25	2-AMINOETHANETHIOL HYDROGEN SULFATE (ESTER)	1.11	749	C2H7NO3S2	157	2937-53-3	WileyRegistry8e
8.25	NEROLIDOL-EPOXYACETATE	1.11	707	C17H28O4	296	NA	WileyRegistry8e
8.25	1-Heptatriacotanol	1.11	681	C37H76O	536	105794-58-9	mainlib
8.25	5à-Androstan-16-one, cyclic ethylene mercaptole	1.11	734	C21H34S2	350	2759-86-6	mainlib
8.25	5à-ANDROSTAN-16-ONE, CYCLIC ETHYLENE MERCAPTOLE	1.11	734	C21H34S2	350	2759-86-6	WileyRegistry8e

Compound Structure

Hit Spectrum

2-AMINOETHANETHIOL HYDROGEN SULFATE (ESTER)
Formula C2H7NO3S2, MW 157, CAS# 2937-53-3, Entry# 41029
2-AMINOETHANETHIOLSULFURIC ACID

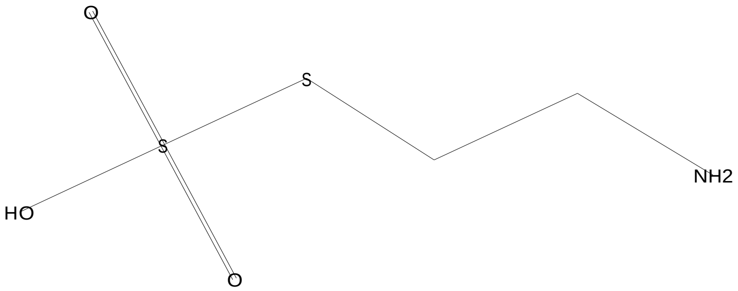

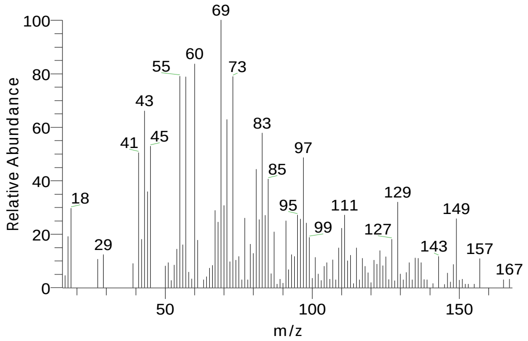

My GC-MS Report

Compound Structure | Hit Spectrum

NEROLIDOL-EPOXYACETATE
Formula C17H28O4, MW 296, CAS# NA, Entry# 185753

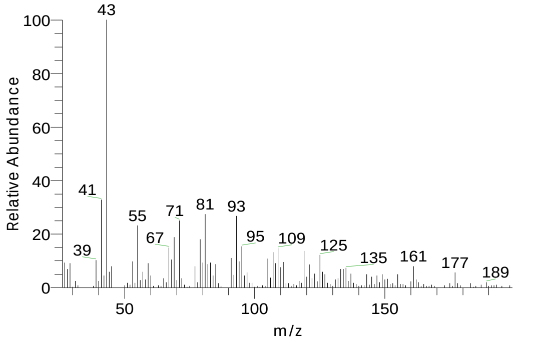

1-Heptatriacotanol
Formula C37H76O, MW 536, CAS# 105794-58-9, Entry# 7279
1-Heptatriacontanol #

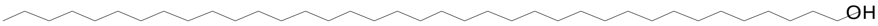

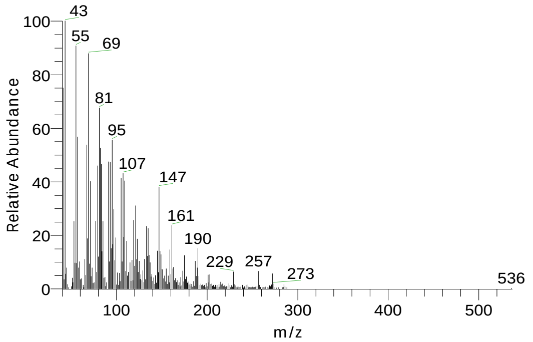

5à-Androstan-16-one, cyclic ethylene mercaptole
Formula C21H34S2, MW 350, CAS# 2759-86-6, Entry# 19678
$:28ZOLPVFXOXCUNAF-UHFFFAOYSA-N

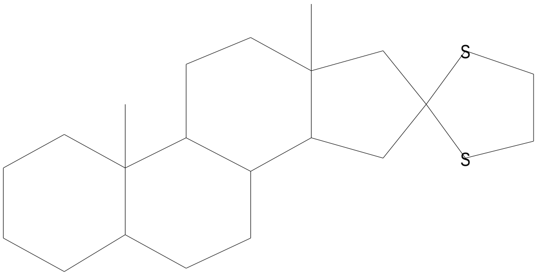

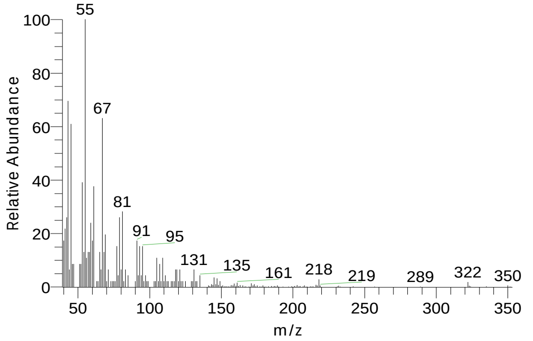

5à-ANDROSTAN-16-ONE, CYCLIC ETHYLENE MERCAPTOLE
Formula C21H34S2, MW 350, CAS# 2759-86-6, Entry# 228761
5ALPHA-ANDROSTAN-16-ONE, CYCLIC ETHYLENE MERCAPTOLE

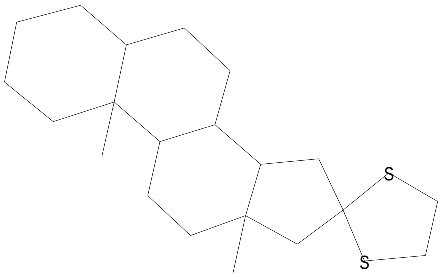

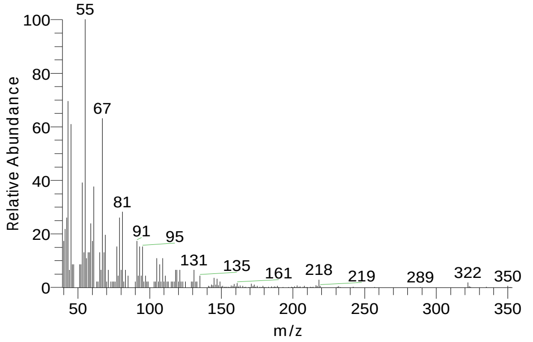

My GC-MS Report

NeemExt #1301 RT: 8.36 AV: 1 NL: 2.17E6
T: + c EI Full ms [50.000-750.000]

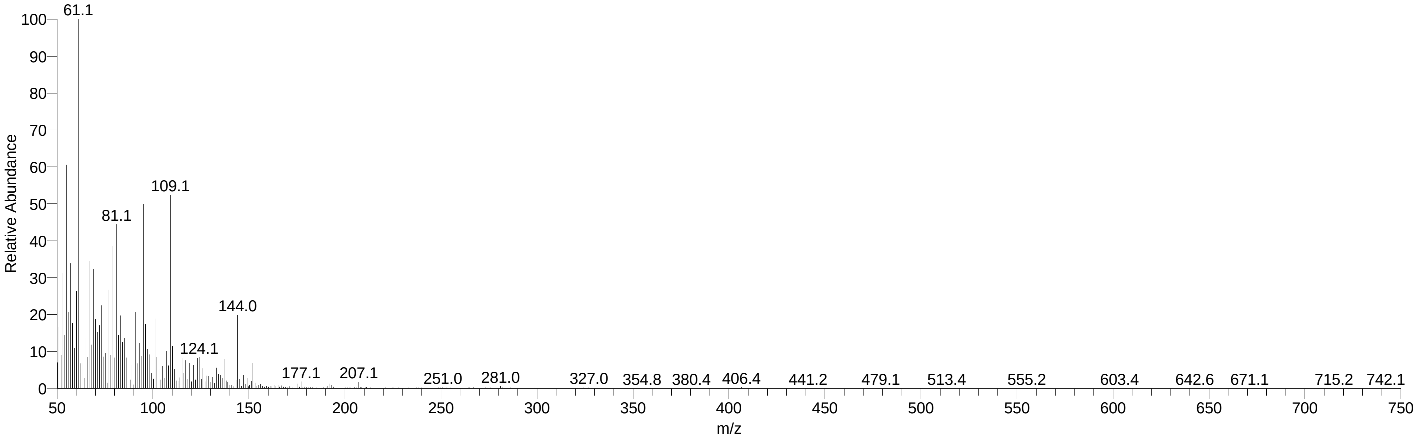

RT	Compound Name	Area %	MF	Molecular Formula	Molecular Weight	Cas #	Library
8.36	1b,5,5,6a-Tetramethyl-octahydro-1-oxa-cyclopropa[a]inden-6-one	3.02	778	C13H20O2	208	NA	mainlib
8.36	1B,5,5,6A-TETRAMETHYL-OCTAHYDRO-1-OXA-CYCLOPROPA[A]INDEN-6-ONE	3.02	778	C13H20O2	208	NA	WileyRegistry8e
8.36	HEXADECADIENOIC ACID, METHYL ESTER	3.02	705	C17H30O2	266	29961-54-4	WileyRegistry8e
8.36	Cyclopropaneoctanoic acid, 2-[[2-[(2-ethylcyclopropyl)methyl]cyclopropyl]methyl]-, methyl ester	3.02	725	C22H38O2	334	10152-71-3	mainlib
8.36	CYCLOPROPANEOCTANOIC ACID, 2-[[2-[(2-ETHYLCYCLOPROPYL)METHYL]CYCLOPROPYL]METHYL]-, METHYL ESTER	3.02	724	C22H38O2	334	10152-71-3	WileyRegistry8e

Compound Structure | Hit Spectrum

1b,5,5,6a-Tetramethyl-octahydro-1-oxa-cyclopropa[a]inden-6-one
Formula C13H20O2, MW 208, CAS# NA, Entry# 2373
1b,5,5,6a-Tetramethyloctahydro-6H-indeno[1,2-b]oxiren-6-one #

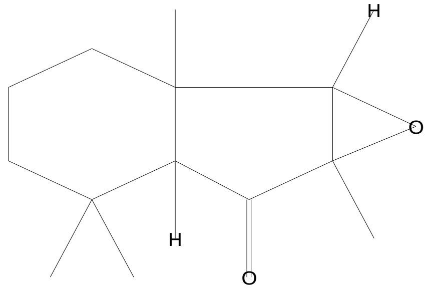

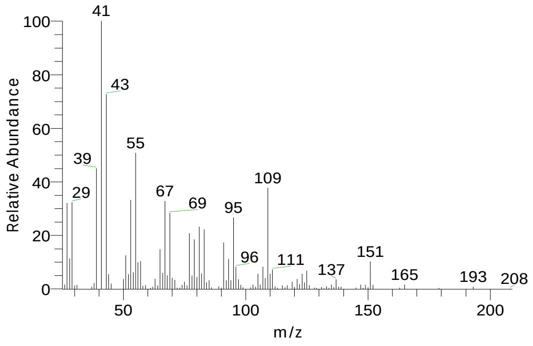

1B,5,5,6A-TETRAMETHYL-OCTAHYDRO-1-OXA-CYCLOPROPA[A]INDEN-6-ONE
Formula C13H20O2, MW 208, CAS# NA, Entry# 367674
1B,5,5,6A-TETRAMETHYLOCTAHYDRO-6H-INDENO[1,2-B]OXIREN-6-ONE

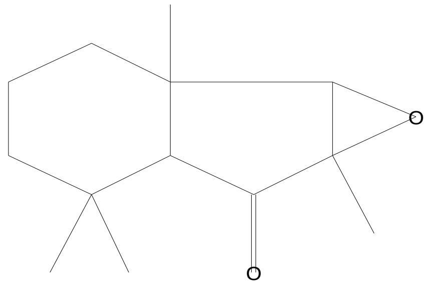

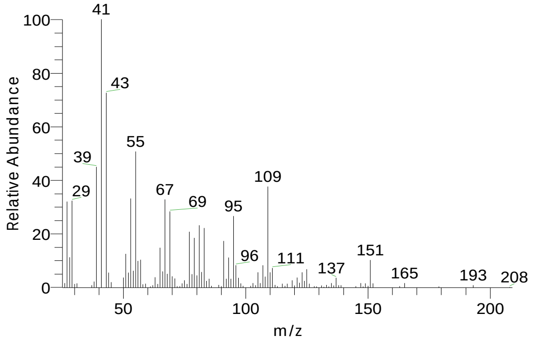

My GC-MS Report

Compound Structure | Hit Spectrum

HEXADECADIENOIC ACID, METHYL ESTER
Formula C17H30O2, MW 266, CAS# 29961-54-4, Entry# 157129
METHYL HEXADECADIENOATE

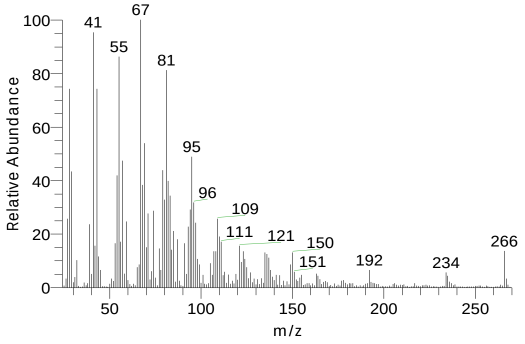

Formula C22H38O2, MW 334, CAS# 10152-71-3, Entry# 2765
Methyl 8-[2-((2-[(2-ethylcyclopropyl)methyl]cyclopropyl)methyl)cyclopropyl]octanoate #

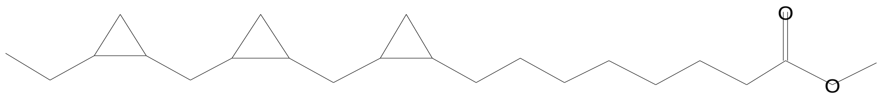

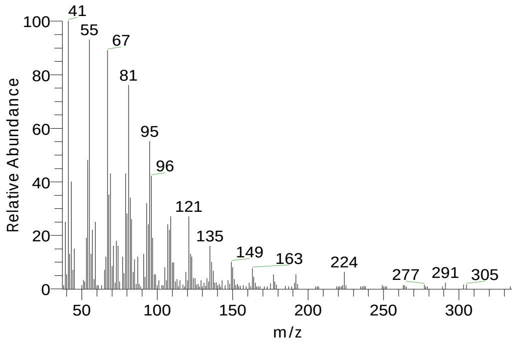

Formula C22H38O2, MW 334, CAS# 10152-71-3, Entry# 217974

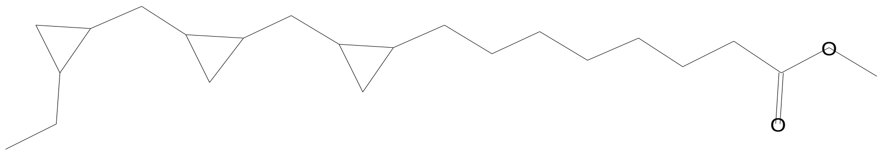

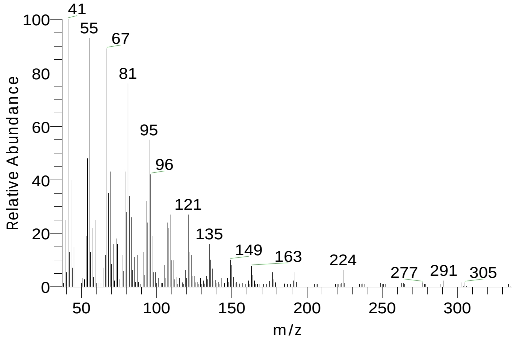

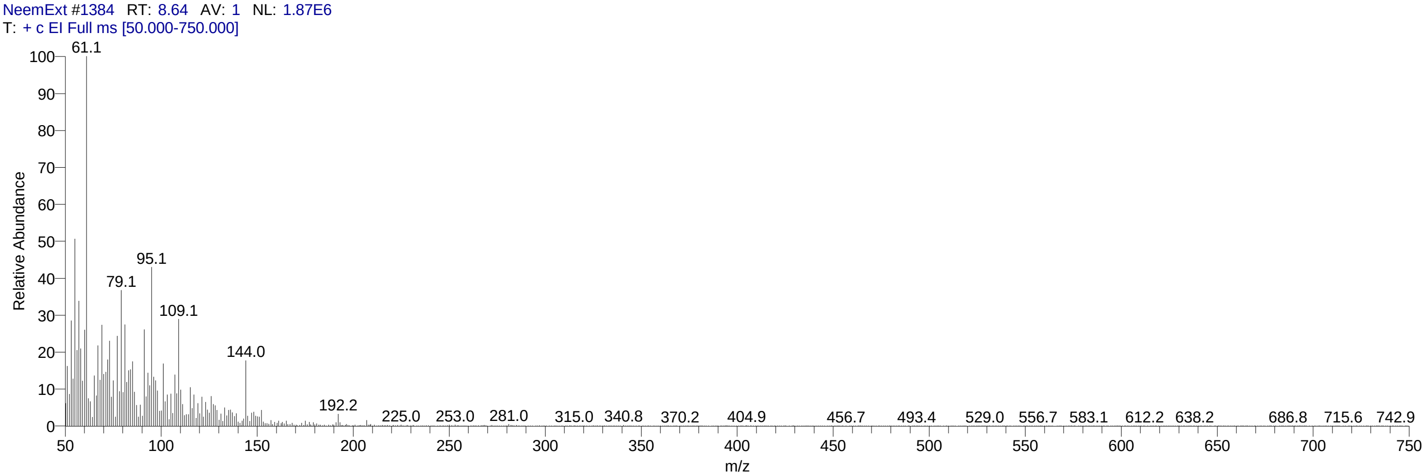

RT	Compound Name	Area %	MF	Molecular Formula	Molecular Weight	Cas #	Library
8.64	6-Acetyl-á-d-mannose	0.55	725	C8H14O7	222	NA	mainlib
8.64	Caryophyllene oxide	0.55	755	C15H24O	220	1139-30-6	mainlib

My GC-MS Report

RT	Compound Name	Area %	MF	Molecular Formula	Molecular Weight	Cas #	Library
8.64	4,12,12-TRIMETHYL-9-METHYLENE-5-OXATRICYCLO[8.2.0.0~4,6~]DODECANE	0.55	755	C15H24O	220	NA	WileyRegistry8e
8.64	n-Propyl 9,12-hexadecadienoate	0.55	727	C19H34O2	294	NA	mainlib
8.64	cis-p-mentha-1(7),8-dien-2-ol	0.55	736	C10H16O	152	NA	mainlib

Compound Structure | Hit Spectrum

6-Acetyl-á-d-mannose
Formula C8H14O7, MW 222, CAS# NA, Entry# 5869
6-O-Acetylhexopyranose #

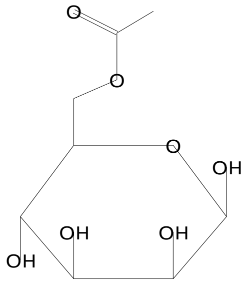

SI 681, RSI 725, mainlib, Entry# 5869, CAS# NA, 6-Acetyl-á-d-mannose

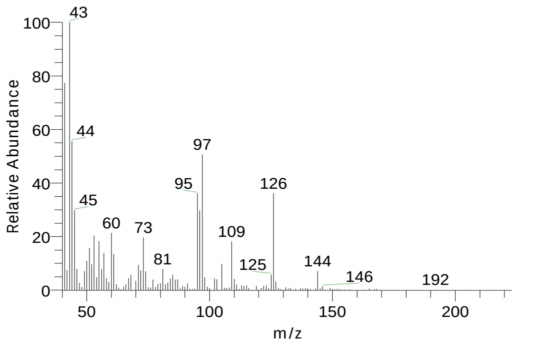

Caryophyllene oxide
Formula C15H24O, MW 220, CAS# 1139-30-6, Entry# 6247

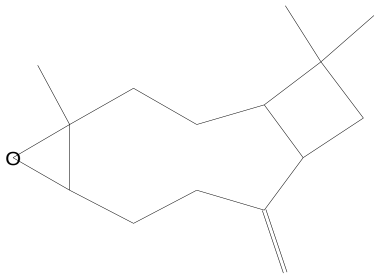

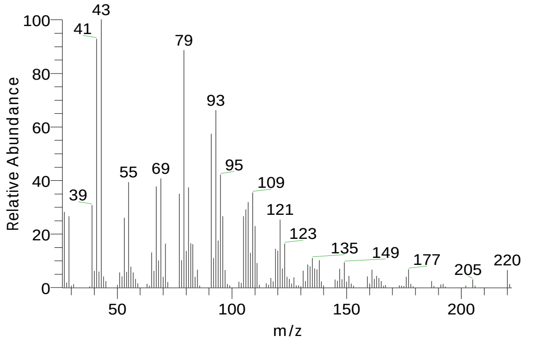

4,12,12-TRIMETHYL-9-METHYLENE-5-OXATRICYCLO[8.2.0.0~4,6~]DODECANE
Formula C15H24O, MW 220, CAS# NA, Entry# 388109
CARYOPHYLLENOXID

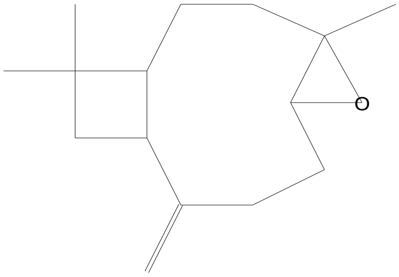

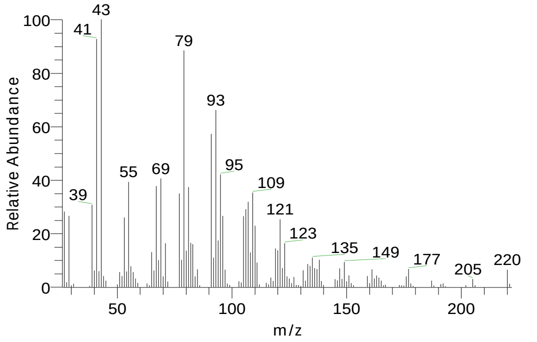

n-Propyl 9,12-hexadecadienoate
Formula C19H34O2, MW 294, CAS# NA, Entry# 32803
$:28ZWJZACIXBOTFLS-HZJYTTRNSA-N

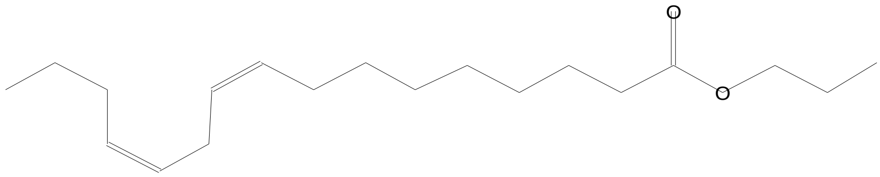

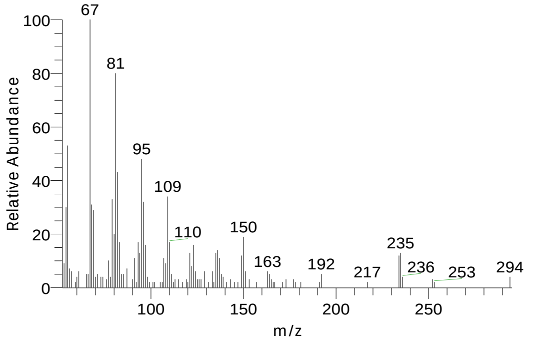

My GC-MS Report

Compound Structure | Hit Spectrum

cis-p-mentha-1(7),8-dien-2-ol
Formula C10H16O, MW 152, CAS# NA, Entry# 2915
$:28PNVTXOFNJFHXOK-UHFFFAOYSA-N

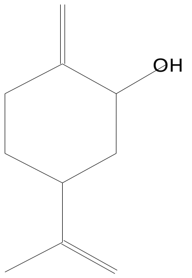

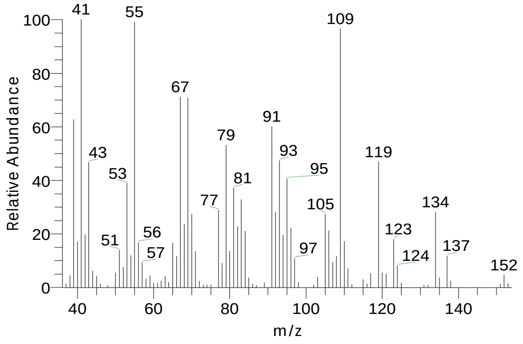

NeemExt #1553 RT: 9.21 AV: 1 NL: 1.52E6
T: + c EI Full ms [50.000-750.000]

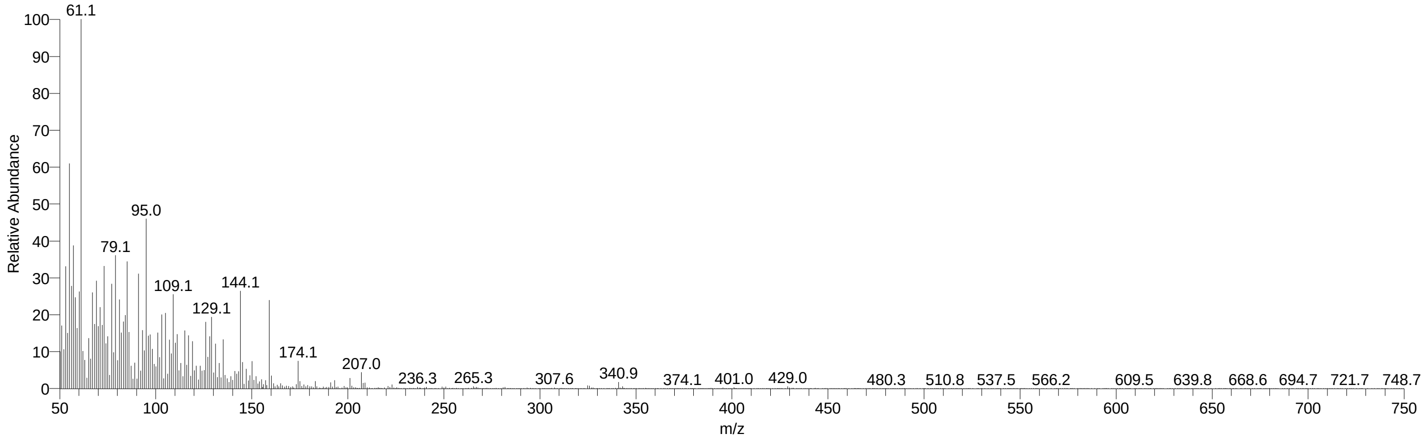

RT	Compound Name	Area %	MF	Molecular Formula	Molecular Weight	Cas #	Library
9.21	Retinal	1.85	800	C20H28O	284	116-31-4	mainlib
9.21	RETINAL	1.85	799	C20H28O	284	116-31-4	WileyRegistry8e
9.21	5à-Androstan-16-one, cyclic ethylene mercaptole	1.85	725	C21H34S2	350	2759-86-6	mainlib
9.21	5à-ANDROSTAN-16-ONE, CYCLIC ETHYLENE MERCAPTOLE	1.85	725	C21H34S2	350	2759-86-6	WileyRegistry8e
9.21	6-Acetyl-á-d-mannose	1.85	711	C8H14O7	222	NA	mainlib

Compound Structure | Hit Spectrum

Retinal
Formula C20H28O, MW 284, CAS# 116-31-4, Entry# 60749
Vitamin A aldehyde

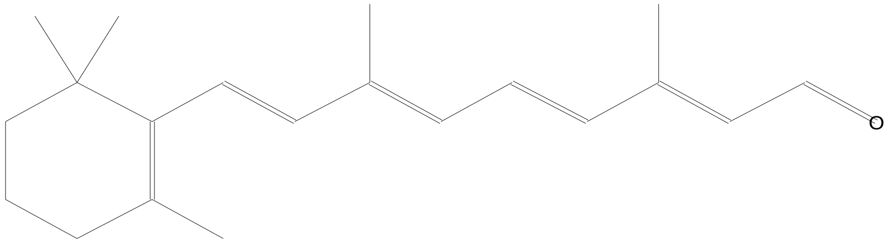

SI 677, RSI 800, mainlib, Entry# 60749, CAS# 116-31-4, Retinal

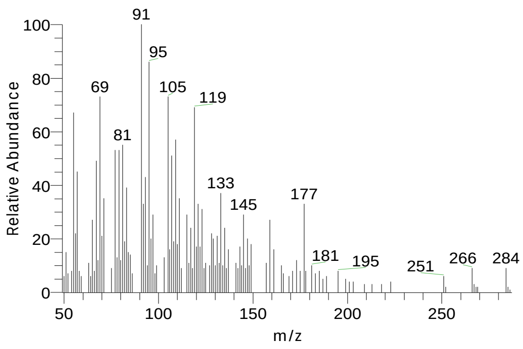

My GC-MS Report

Compound Structure | Hit Spectrum

RETINAL
Formula C20H28O, MW 284, CAS# 116-31-4, Entry# 175130
à-RETINENE

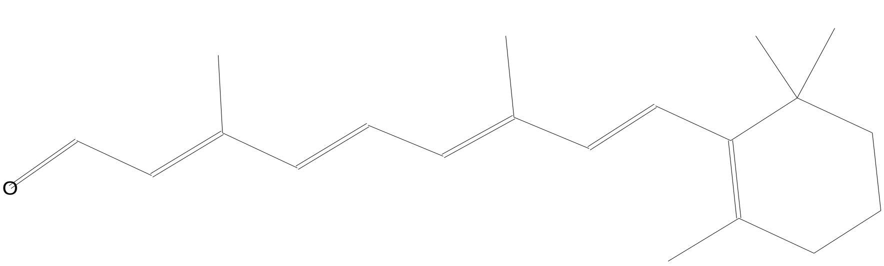

SI 677, RSI 799, WileyRegistry8e, Entry# 175130, CAS# 116-31-4, RETINAL

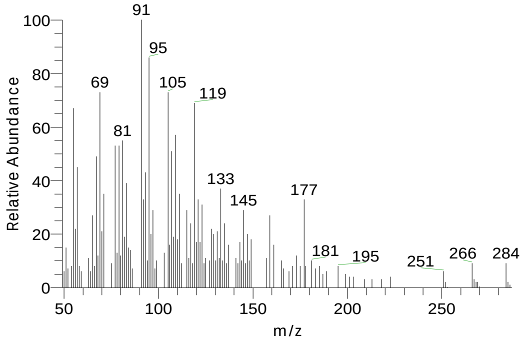

5à-Androstan-16-one, cyclic ethylene mercaptole
Formula C21H34S2, MW 350, CAS# 2759-86-6, Entry# 19678
$:28ZOLPVFXOXCUNAF-UHFFFAOYSA-N

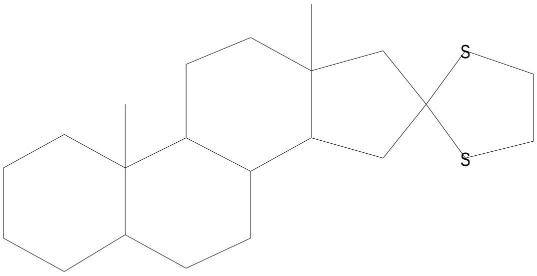

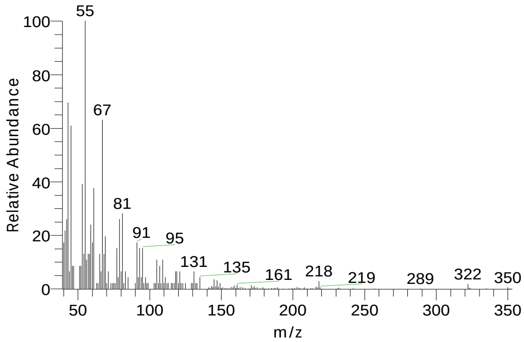

5à-ANDROSTAN-16-ONE, CYCLIC ETHYLENE MERCAPTOLE
Formula C21H34S2, MW 350, CAS# 2759-86-6, Entry# 228761
5ALPHA-ANDROSTAN-16-ONE, CYCLIC ETHYLENE MERCAPTOLE

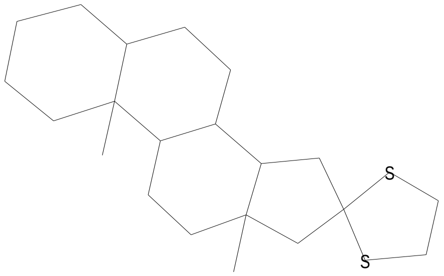

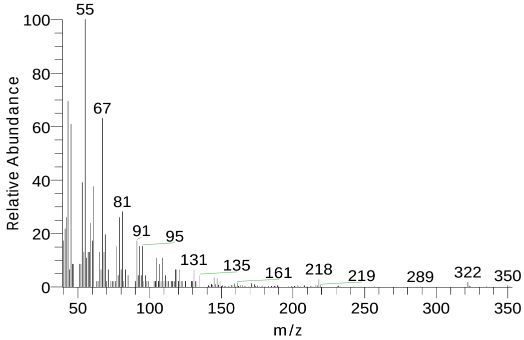

6-Acetyl-á-d-mannose
Formula C8H14O7, MW 222, CAS# NA, Entry# 5869
6-O-Acetylhexopyranose #

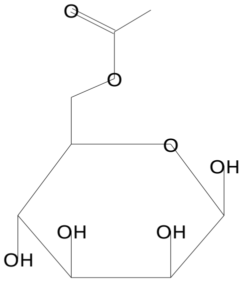

SI 642, RSI 711, mainlib, Entry# 5869, CAS# NA, 6-Acetyl-á-d-mannose

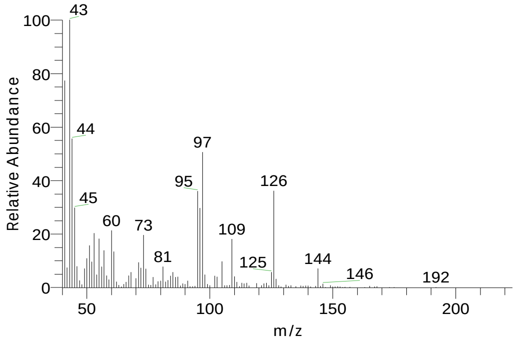

My GC-MS Report

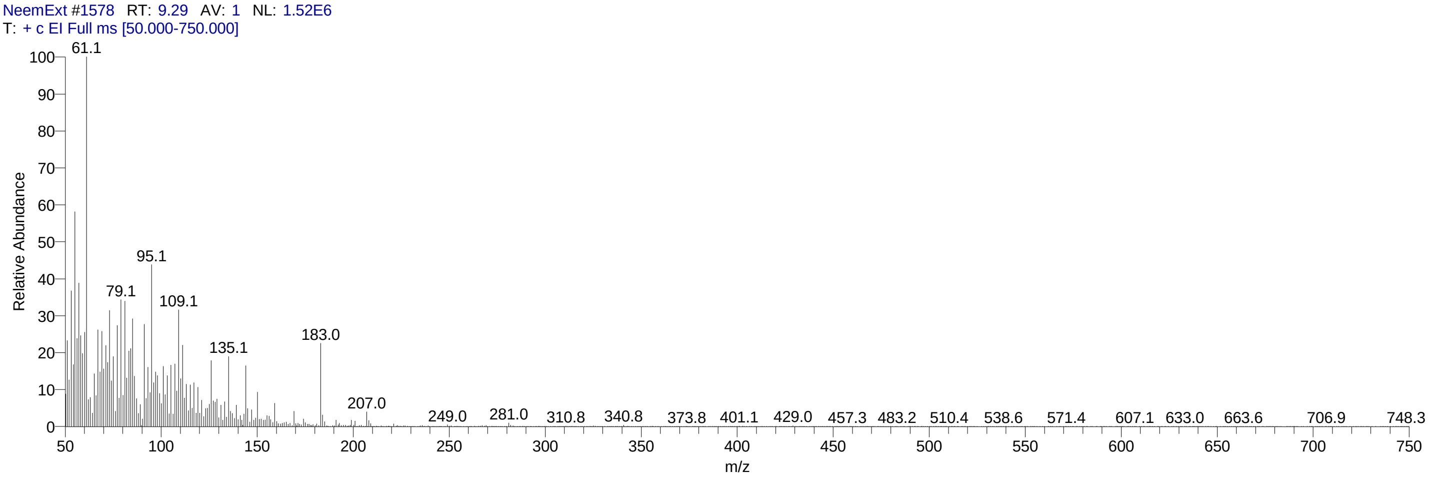

RT	Compound Name	Area %	MF	Molecular Formula	Molecular Weight	Cas #	Library
9.29	9,12,15-Octadecatrienoic acid, 2,3-bis(acetyloxy)propyl ester, (Z,Z,Z)-	1.52	788	C25H40O6	436	55320-02-0	mainlib
9.29	9,12,15-OCTADECATRIENOIC ACID, 2,3-BIS(ACETYLOXY)PROPYL ESTER, (Z,Z,Z)-	1.52	788	C25H40O6	436	55320-02-0	WileyRegistry8e
9.29	10-Heptadecen-8-ynoic acid, methyl ester, (E)-	1.52	712	C18H30O2	278	16714-85-5	mainlib
9.29	10-HEPTADECEN-8-YNOIC ACID, METHYL ESTER, (E)-	1.52	712	C18H30O2	278	16714-85-5	WileyRegistry8e
9.29	MALONIC ACID, 6-HEPTYNYL-	1.52	723	C10H14O4	198	90927-19-8	WileyRegistry8e

Compound Structure | Hit Spectrum

9,12,15-Octadecatrienoic acid, 2,3-bis(acetyloxy)propyl ester, (Z,Z,Z)-
Formula C25H40O6, MW 436, CAS# 55320-02-0, Entry# 7161
2,3-Bis(acetyloxy)propyl (9E,12E,15E)-9,12,15-octadecatrienoate #

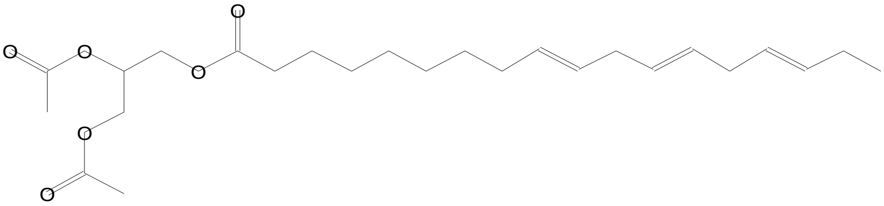

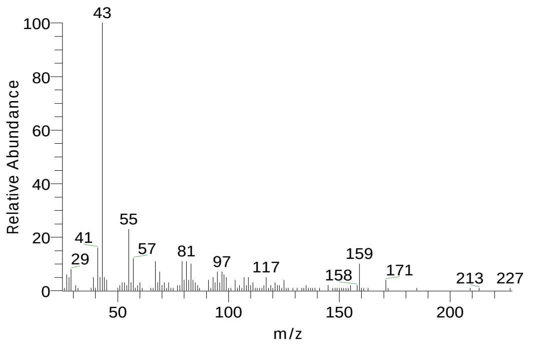

9,12,15-OCTADECATRIENOIC ACID, 2,3-BIS(ACETYLOXY)PROPYL ESTER, (Z,Z,Z)-
Formula C25H40O6, MW 436, CAS# 55320-02-0, Entry# 270143
2,3-BIS(ACETYLOXY)PROPYL (9E,12E,15E)-9,12,15-OCTADECATRIENOATE #

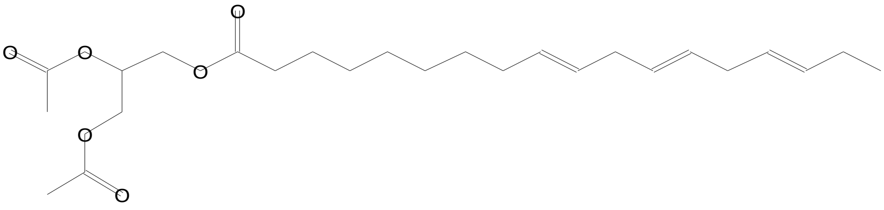

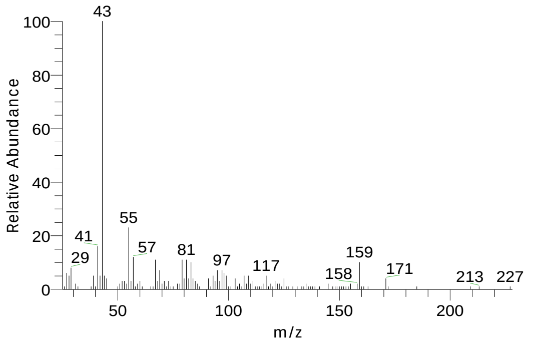

My GC-MS Report

Compound Structure | Hit Spectrum

10-Heptadecen-8-ynoic acid, methyl ester, (E)-
Formula C18H30O2, MW 278, CAS# 16714-85-5, Entry# 48853
Methyl (10E)-10-heptadecen-8-ynoate #

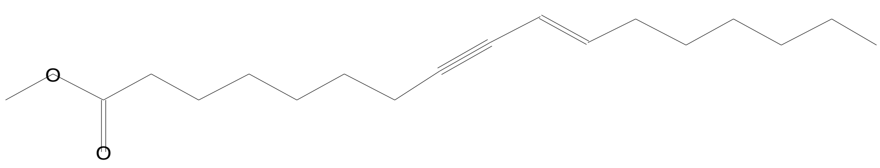

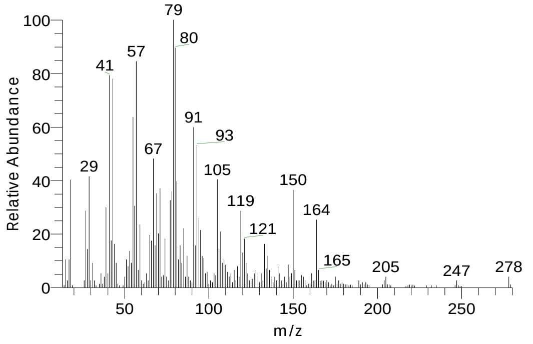

10-HEPTADECEN-8-YNOIC ACID, METHYL ESTER, (E)-
Formula C18H30O2, MW 278, CAS# 16714-85-5, Entry# 169022
METHYL (10E)-10-HEPTADECEN-8-YNOATE #

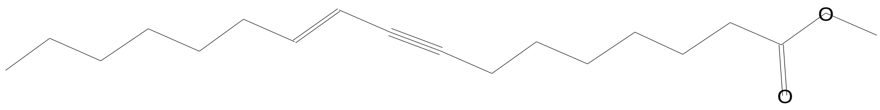

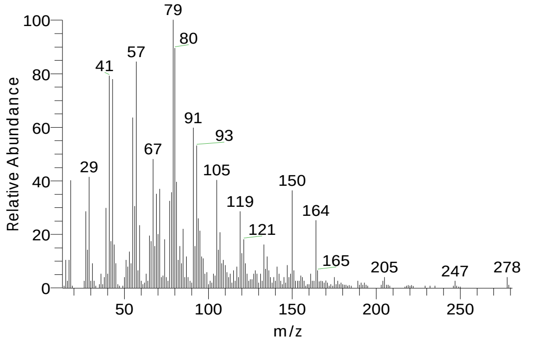

MALONIC ACID, 6-HEPTYNYL-
Formula C10H14O4, MW 198, CAS# 90927-19-8, Entry# 81740
HEPT-6-YNYL MALONIC ACID

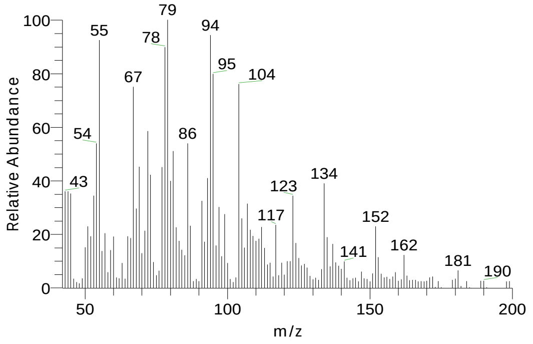

NeemExt #1718 RT: 9.76 AV: 1 NL: 1.77E6
T: + c EI Full ms [50.000-750.000]

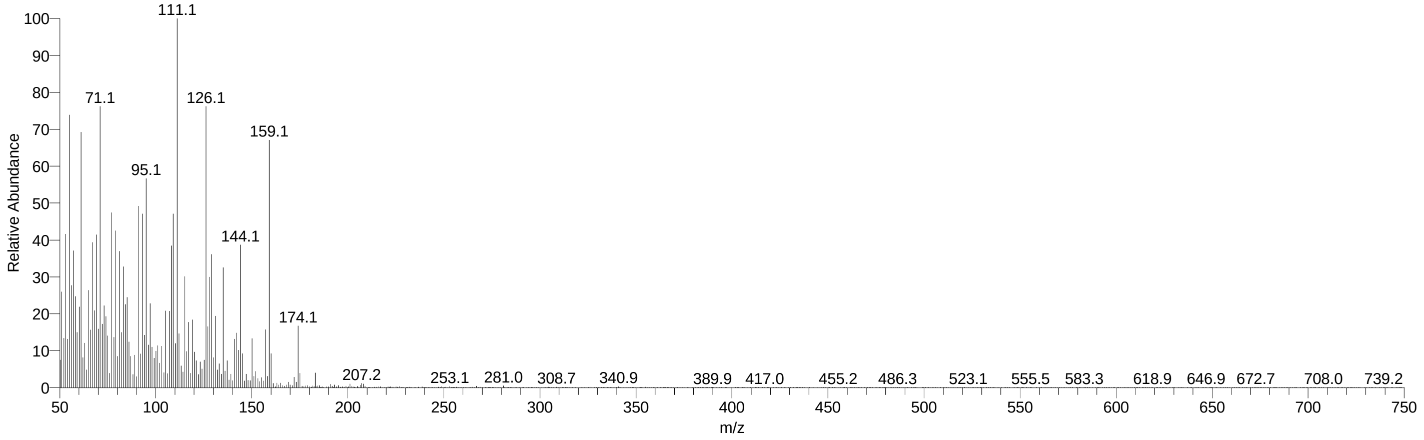

RT	Compound Name	Area %	MF	Molecular Formula	Molecular Weight	Cas #	Library
9.76	NEPETALACTOL	9.39	741	C10H16O2	168	NA	WileyRegi stry8e
9.76	Isocalamendiol	9.39	654	C15H26O2	238	NA	mainlib
9.76	10-Methyl-8-tetradecen-1-ol acetate	9.39	642	C17H32O2	268	NA	mainlib
9.76	E-8-Methyl-7-dodecen-1-ol acetate	9.39	643	C15H28O2	240	NA	mainlib

My GC-MS Report

RT	Compound Name	Area %	MF	Molecular Formula	Molecular Weight	Cas #	Library
9.76	5-HYDROXY-6-METHYL-12,13-DIOXA-TRICYCLO[7.3.1.0 1,6]TRIDECANE-10-CARBOXYLIC ACID METHYL ESTER	9.39	636	C14H22O5	270	NA	WileyRegistry8e

Compound Structure | Hit Spectrum

NEPETALACTOL
Formula C10H16O2, MW 168, CAS# NA, Entry# 51362

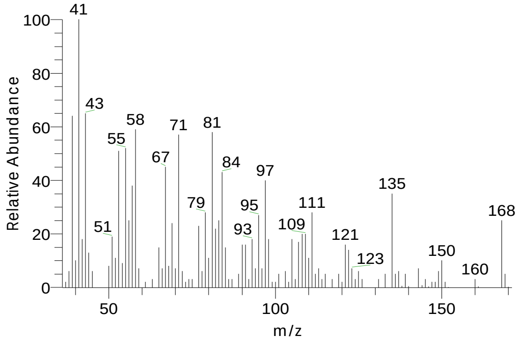

Isocalamendiol
Formula C15H26O2, MW 238, CAS# NA, Entry# 12737
$:28AHNGXHRYFGQWSL-UHFFFAOYSA-N

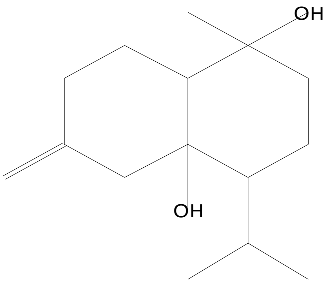

SI 647, RSI 654, mainlib, Entry# 12737, CAS# NA, Isocalamendiol

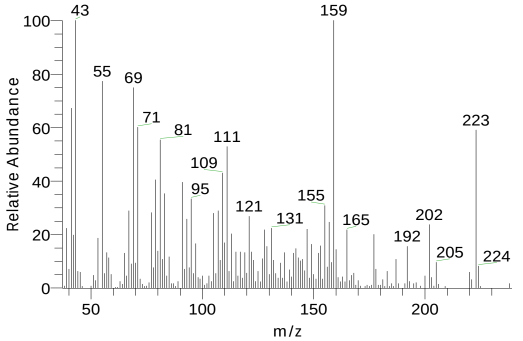

10-Methyl-8-tetradecen-1-ol acetate
Formula C17H32O2, MW 268, CAS# NA, Entry# 7048
(8E)-10-Methyl-8-tetradecenyl acetate #

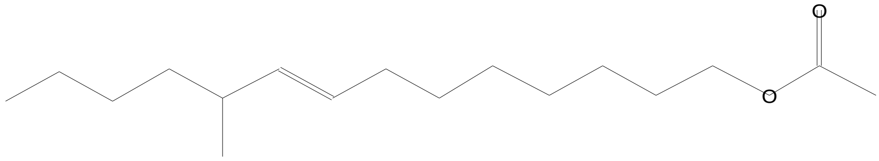

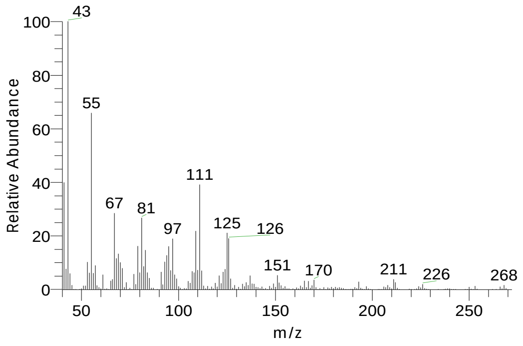

E-8-Methyl-7-dodecen-1-ol acetate
Formula C15H28O2, MW 240, CAS# NA, Entry# 7046
(7E)-8-Methyl-7-dodecenyl acetate #

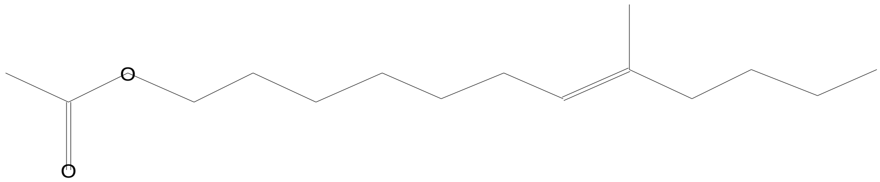

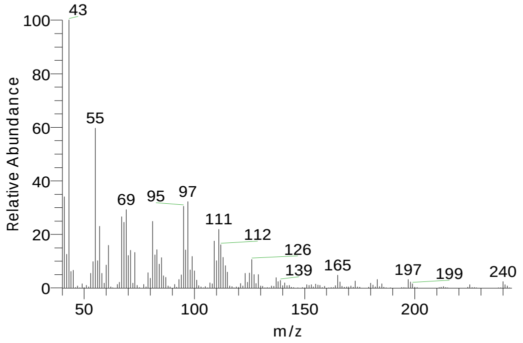

My GC-MS Report

Compound Structure | Hit Spectrum

Formula C14H22O5, MW 270, CAS# NA, Entry# 367937

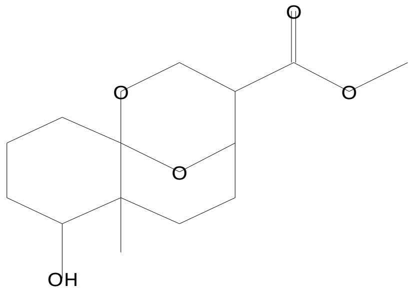

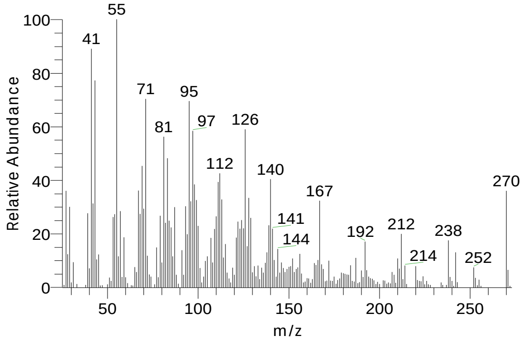

NeemExt #1987 RT: 10.66 AV: 1 NL: 2.02E6
T: + c EI Full ms [50.000-750.000]

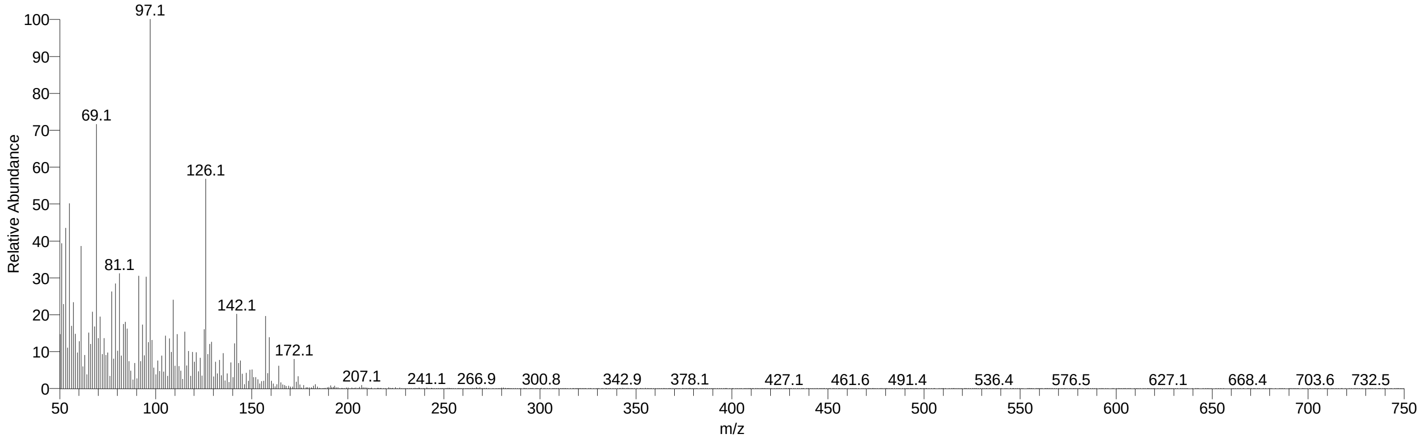

RT	Compound Name	Area %	MF	Molecular Formula	Molecular Weight	Cas #	Library
10.66	7-Methyl-Z-tetradecen-1-ol acetate	7.59	716	C17H32O2	268	NA	mainlib
10.66	4,4'-DIHYDROXYDIPHENYLSULPHONE	7.59	695	C12H10O4S	250	80-09-1	WileyRegistry8e
10.66	E-8-Methyl-7-dodecen-1-ol acetate	7.59	678	C15H28O2	240	NA	mainlib
10.66	16-Nitrobicyclo[10.4.0]hexadecan-1-ol-13-one	7.59	651	C16H27NO4	297	79880-69-6	mainlib
10.66	4A-HYDROXY-4-NITROTETRADECAHYDROBENZO[A]CYCLODODECEN-1(2H)-ONE	7.59	651	C16H27NO4	297	79880-69-6	WileyRegistry8e

Compound Structure | Hit Spectrum

7-Methyl-Z-tetradecen-1-ol acetate
Formula C17H32O2, MW 268, CAS# NA, Entry# 7041
(8Z)-7-Methyl-8-tetradecenyl acetate #

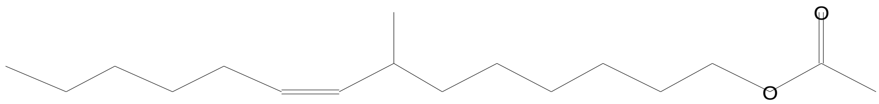

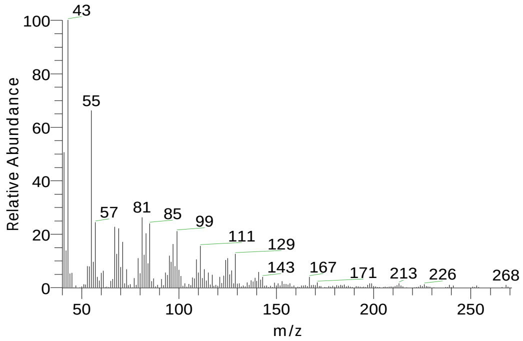

My GC-MS Report

Compound Structure | Hit Spectrum

4,4'-DIHYDROXYDIPHENYLSULPHONE
Formula C12H10O4S, MW 250, CAS# 80-09-1, Entry# 139459
PHENOL, 4,4'-SULFONYLBIS-

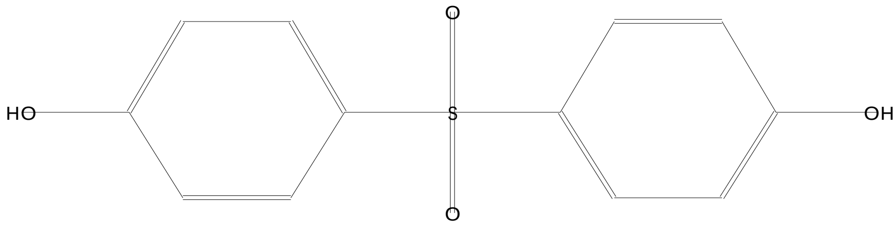

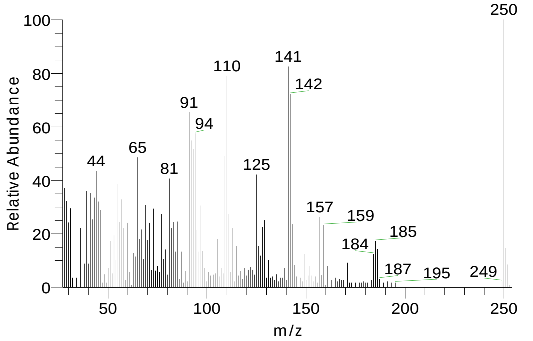

E-8-Methyl-7-dodecen-1-ol acetate
Formula C15H28O2, MW 240, CAS# NA, Entry# 7046
(7E)-8-Methyl-7-dodecenyl acetate #

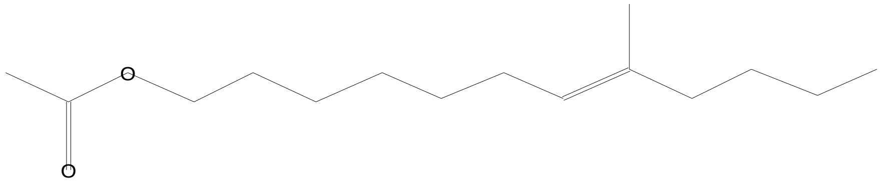

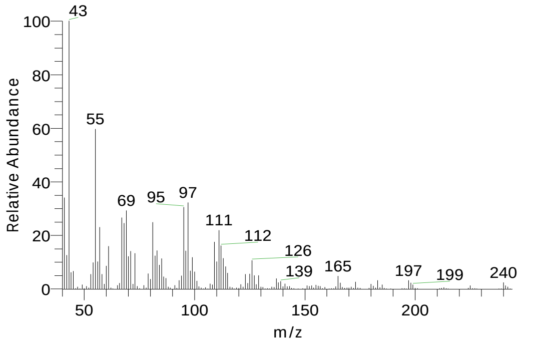

16-Nitrobicyclo[10.4.0]hexadecan-1-ol-13-one
Formula C16H27NO4, MW 297, CAS# 79880-69-6, Entry# 21183
4a-Hydroxy-4-nitrotetradecahydrobenzo[a]cyclododecen-1(2H)-one #

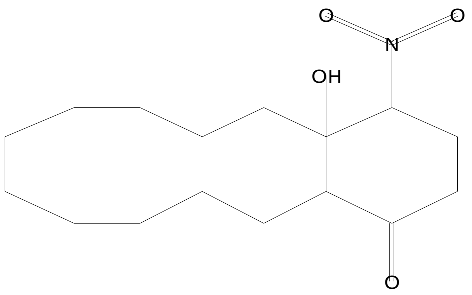

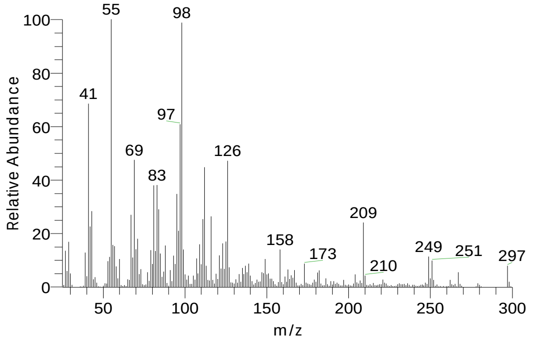

4A-HYDROXY-4-NITROTETRADECAHYDROBENZO[A]CYCLODODECEN-1(2H)-ONE
Formula C16H27NO4, MW 297, CAS# 79880-69-6, Entry# 364572

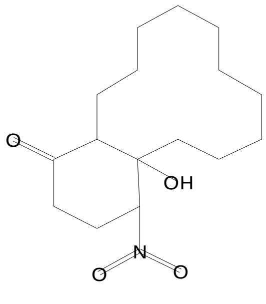

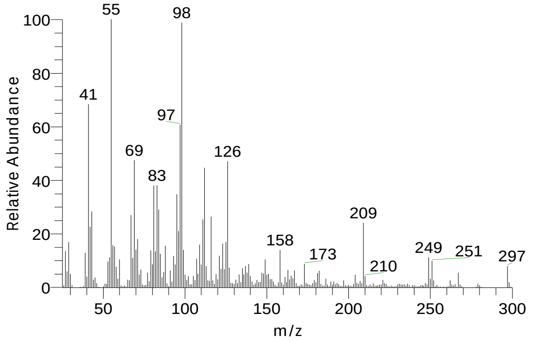

My GC-MS Report

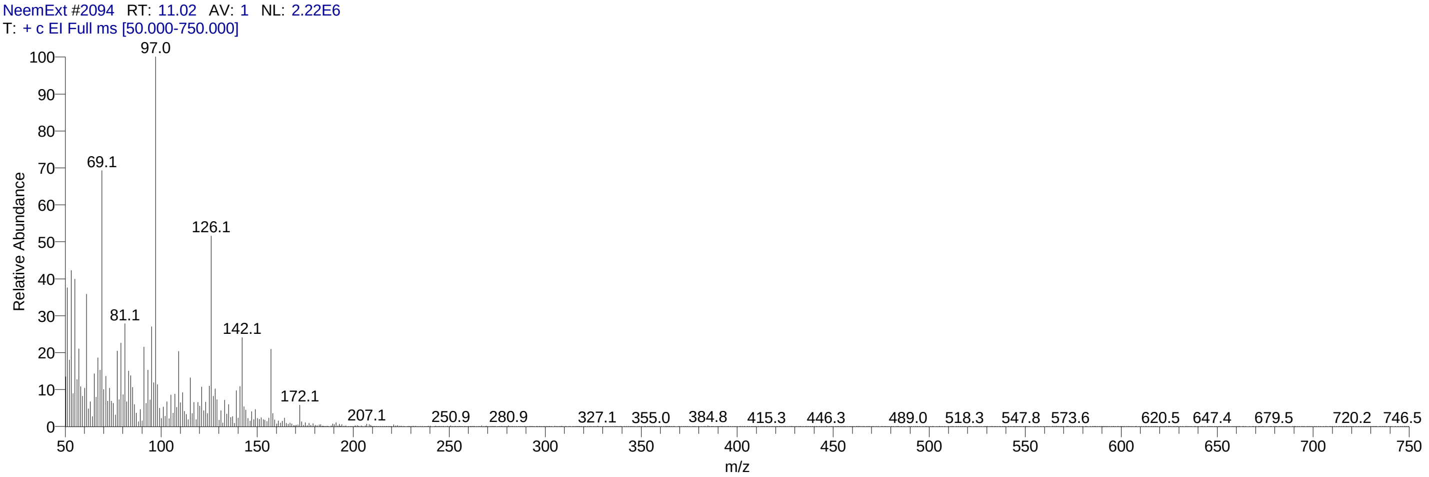

RT	Compound Name	Area %	MF	Molecular Formula	Molecular Weight	Cas #	Library
11.02	16-Nitrobicyclo[10.4.0]hexadecan-1-ol-13-one	1.50	664	C16H27NO4	297	79880-69-6	mainlib
11.02	4A-HYDROXY-4-NITROTETRADECAHYDROBENZO[A]CYCLODODECEN-1(2H)-ONE	1.50	664	C16H27NO4	297	79880-69-6	WileyRegistry8e
11.02	Oxiraneoctanoic acid, 3-octyl-, cis-	1.50	678	C18H34O3	298	24560-98-3	replib
11.02	Oxiraneoctanoic acid, 3-octyl-, cis-	1.50	659	C18H34O3	298	24560-98-3	mainlib
11.02	OXIRANEOCTANOIC ACID, 3-OCTYL-, CIS-	1.50	676	C18H34O3	298	24560-98-3	WileyRegistry8e

Compound Structure	Hit Spectrum

16-Nitrobicyclo[10.4.0]hexadecan-1-ol-13-one
Formula C16H27NO4, MW 297, CAS# 79880-69-6, Entry# 21183
4a-Hydroxy-4-nitrotetradecahydrobenzo[a]cyclododecen-1(2H)-one #

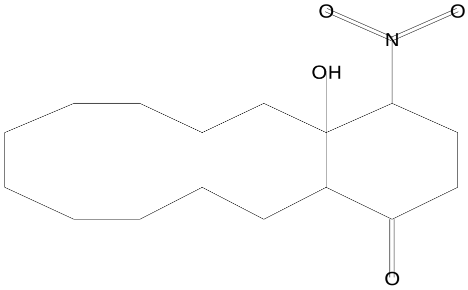

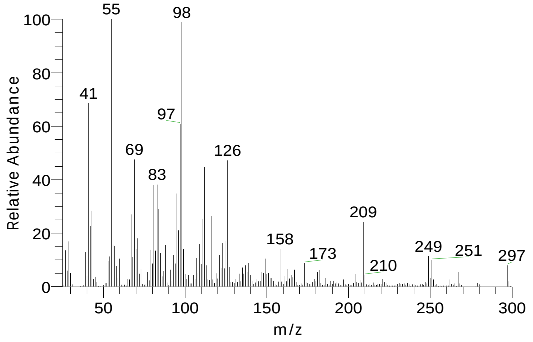

4A-HYDROXY-4-NITROTETRADECAHYDROBENZO[A]CYCLODODECEN-1(2H)-ONE
Formula C16H27NO4, MW 297, CAS# 79880-69-6, Entry# 364572

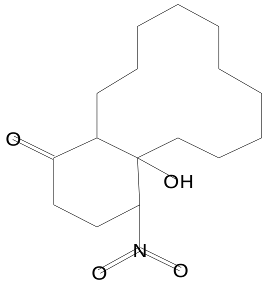

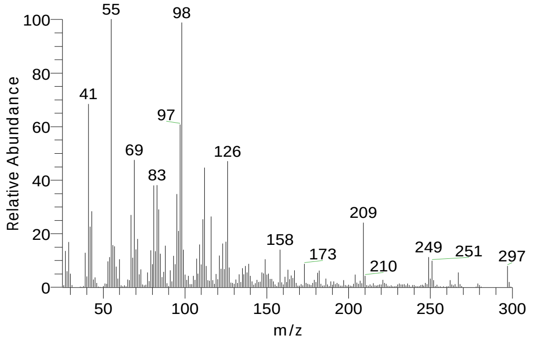

My GC-MS Report

Compound Structure | Hit Spectrum

Oxiraneoctanoic acid, 3-octyl-, cis-
Formula C18H34O3, MW 298, CAS# 24560-98-3, Entry# 4626
Octadecanoic acid, 9,10-epoxy-, cis-

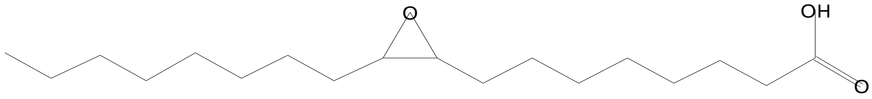

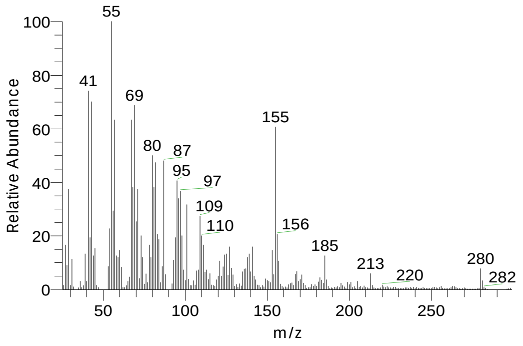

Oxiraneoctanoic acid, 3-octyl-, cis-
Formula C18H34O3, MW 298, CAS# 24560-98-3, Entry# 144282
Octadecanoic acid, 9,10-epoxy-, cis-

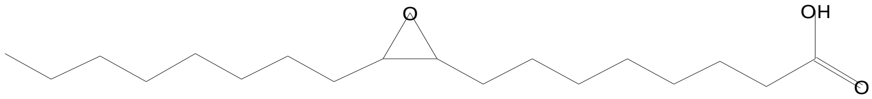

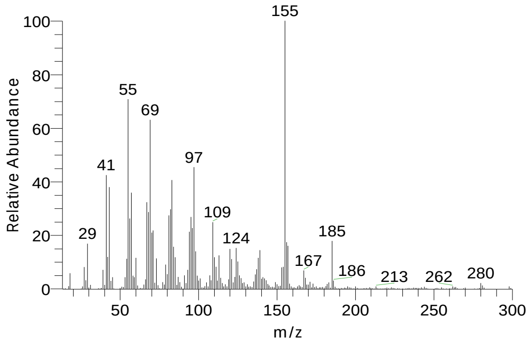

OXIRANEOCTANOIC ACID, 3-OCTYL-, CIS-
Formula C18H34O3, MW 298, CAS# 24560-98-3, Entry# 187808
8-(3-OCTYL-2-OXIRANYL)OCTANOIC ACID #

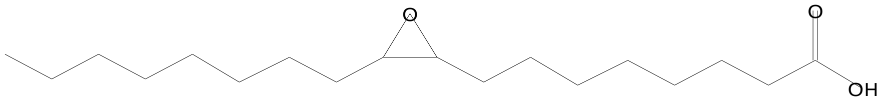

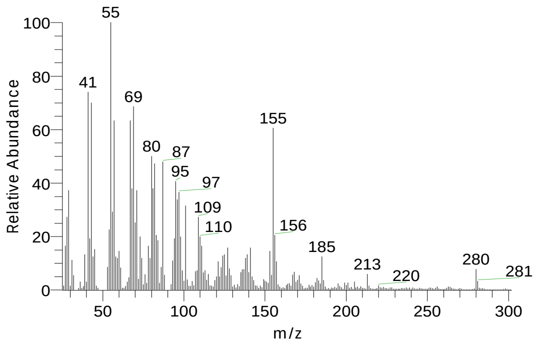

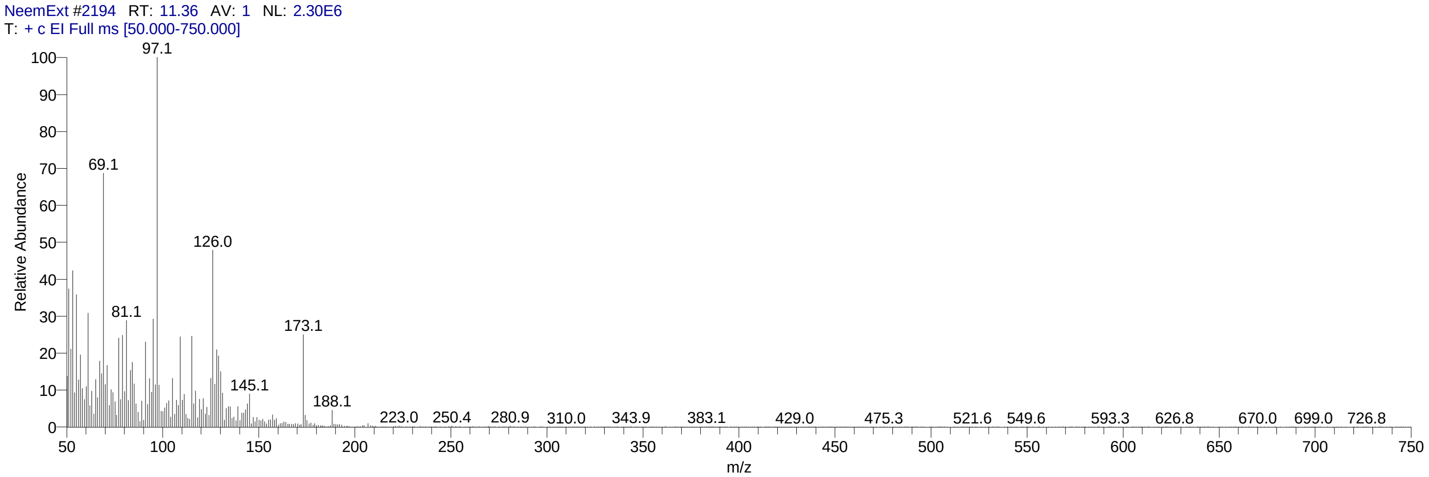

RT	Compound Name	Area %	MF	Molecular Formula	Molecular Weight	Cas #	Library
11.36	1,3,5-TRIAZINE-2,4-DIAMINE, 6-CHLORO-N-ETHYL-	2.72	759	C5H8ClN5	173	1007-28-9	WileyRegistry8e
11.36	2-AMINO-5-GUANIDINO-PENTANOIC ACID	2.72	679	C6H14N4O2	174	74-79-3	WileyRegistry8e

My GC-MS Report

RT	Compound Name	Area %	MF	Molecular Formula	Molecular Weight	Cas #	Library
11.36	1-Dodecanol, 3,7,11-trimethyl-	2.72	682	C15H32O	228	6750-34-1	replib
11.36	1-DODECANOL, 3,7,11-TRIMETHYL-	2.72	682	C15H32O	228	6750-34-1	WileyRegistry8e
11.36	15-Deoxy-.DELTA.12,14-prostaglandin J2-biotinamide	2.72	676	C35H54N4O4S	626	NA	nist_msms

Compound Structure

Hit Spectrum

1,3,5-TRIAZINE-2,4-DIAMINE, 6-CHLORO-N-ETHYL-
Formula C5H8ClN5, MW 173, CAS# 1007-28-9, Entry# 56163
1,3, 5-TRIAZINE-2,4-DIAMINE, 6-CHLORO-N-ETHYL-

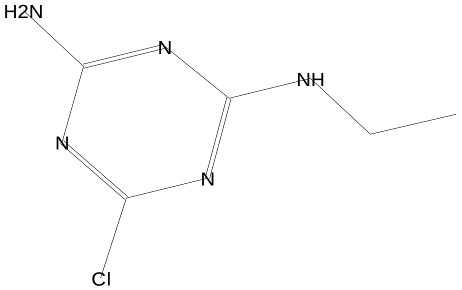

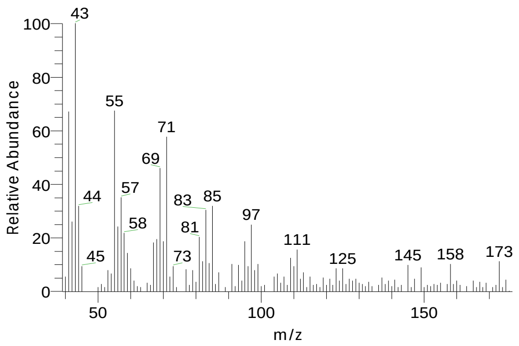

2-AMINO-5-GUANIDINO-PENTANOIC ACID
Formula C6H14N4O2, MW 174, CAS# 74-79-3, Entry# 56662
(L)-ARGININE

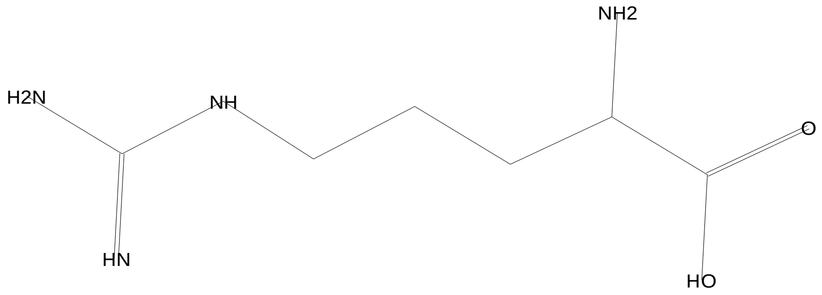

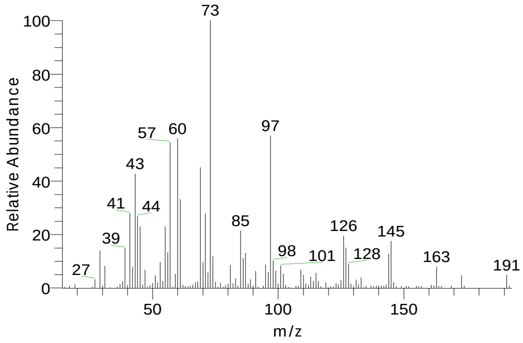

1-Dodecanol, 3,7,11-trimethyl-
Formula C15H32O, MW 228, CAS# 6750-34-1, Entry# 6352
Hexa-hydro-farnesol

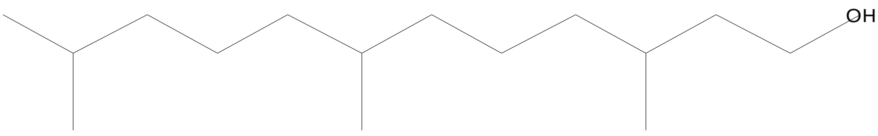

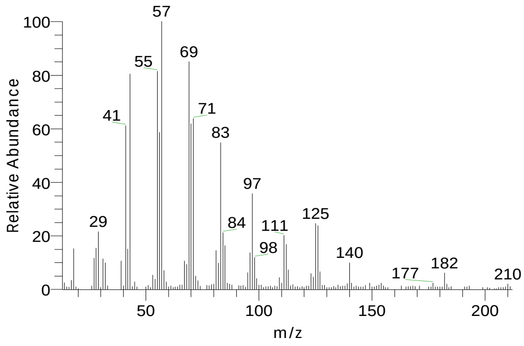

1-DODECANOL, 3,7,11-TRIMETHYL-
Formula C15H32O, MW 228, CAS# 6750-34-1, Entry# 116851
3,7,11-TRIMETHYL-1-DODECANOL

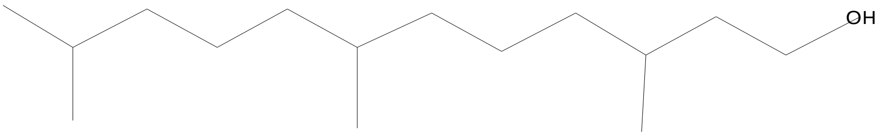

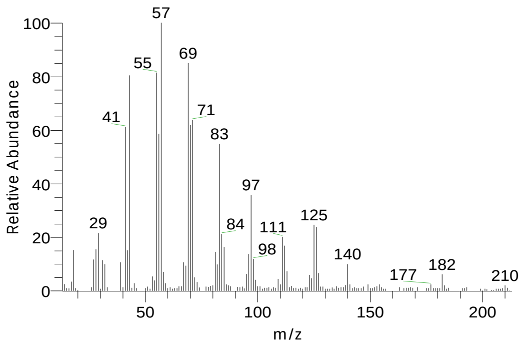

My GC-MS Report

Compound Structure | Hit Spectrum

15-Deoxy-.DELTA.12,14-prostaglandin J2-biotinamide
Formula C35H54N4O4S, MW 626, CAS# NA, Entry# 10288
$:03[M+H]+

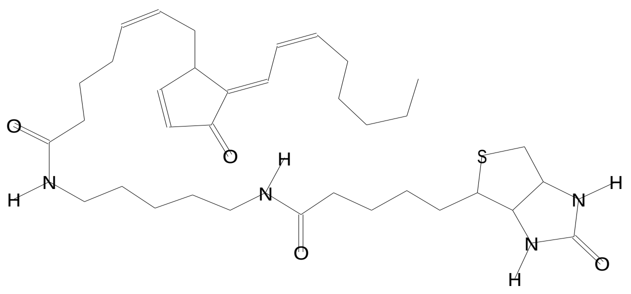

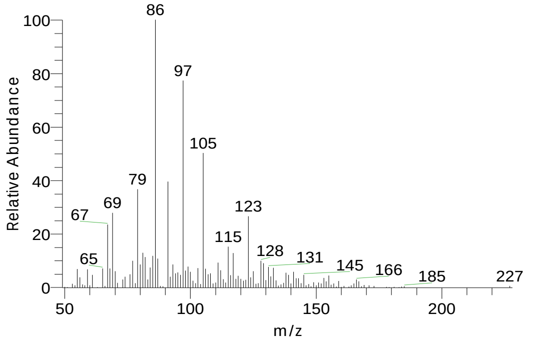

NeemExt #2312 RT: 11.75 AV: 1 NL: 2.08E6
T: + c EI Full ms [50.000-750.000]

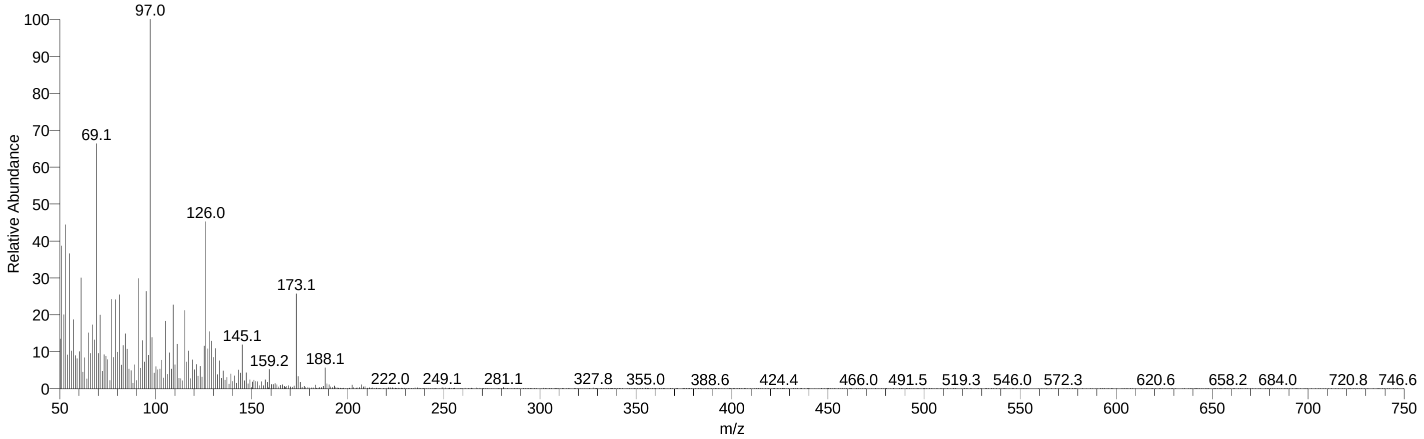

RT	Compound Name	Area %	MF	Molecular Formula	Molecular Weight	Cas #	Library
11.75	1,3,5-TRIAZINE-2,4-DIAMINE, 6-CHLORO-N-ETHYL-	3.99	768	C5H8ClN5	173	1007-28-9	WileyRegistry8e
11.75	15-Deoxy-.DELTA.12,14-prostaglandin J2-biotinamide	3.99	703	C35H54N4O4S	626	NA	nist_msms
11.75	15-Deoxy-.DELTA.12,14-prostaglandin J2-biotinamide	3.99	701	C35H54N4O4S	626	NA	nist_msms
11.75	Oct-3-ene-1,5-diyne, 3-t-butyl-7,7-dimethyl-	3.99	678	C14H20	188	NA	mainlib
11.75	3-TERT-BUTYL-7,7-DIMETHYL-3-OCTENE-1,5-DIYNE	3.99	678	C14H20	188	NA	WileyRegistry8e

Compound Structure | Hit Spectrum

1,3,5-TRIAZINE-2,4-DIAMINE, 6-CHLORO-N-ETHYL-
Formula C5H8ClN5, MW 173, CAS# 1007-28-9, Entry# 56163
1,3, 5-TRIAZINE-2,4-DIAMINE, 6-CHLORO-N-ETHYL-

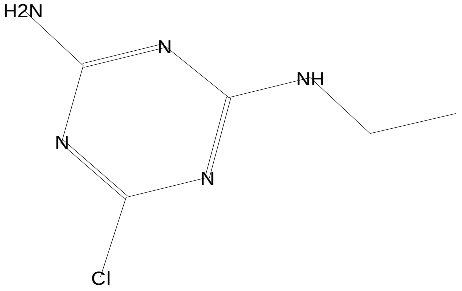

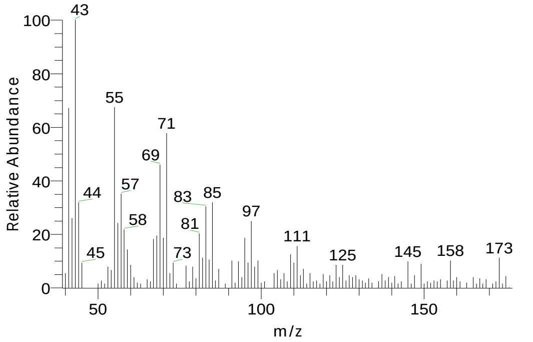

My GC-MS Report

Compound Structure | Hit Spectrum

15-Deoxy-.DELTA.12,14-prostaglandin J2-biotinamide
Formula C35H54N4O4S, MW 626, CAS# NA, Entry# 10310
$:03[M+2H]2+

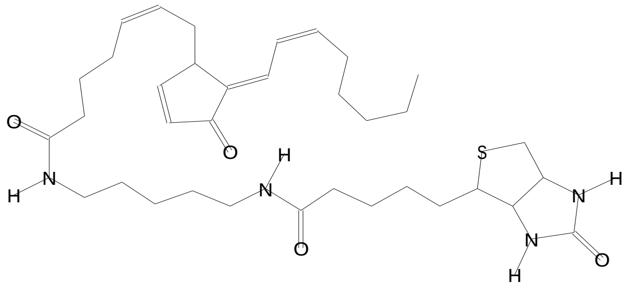

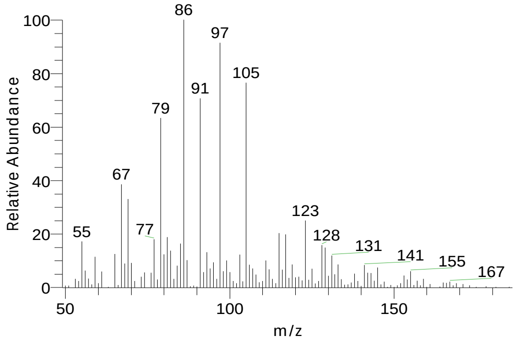

15-Deoxy-.DELTA.12,14-prostaglandin J2-biotinamide
Formula C35H54N4O4S, MW 626, CAS# NA, Entry# 10309
$:03[M+2H]2+

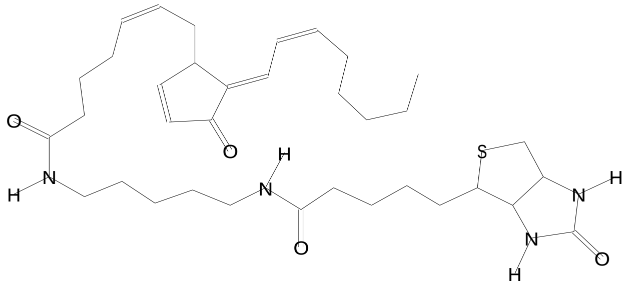

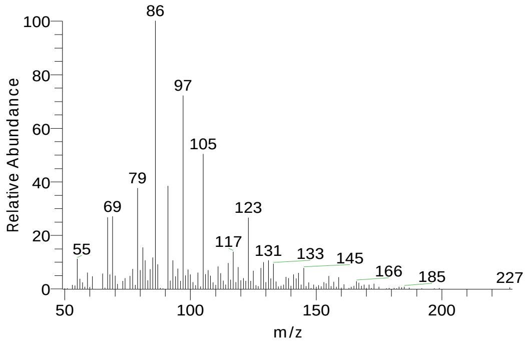

Oct-3-ene-1,5-diyne, 3-t-butyl-7,7-dimethyl-
Formula C14H20, MW 188, CAS# NA, Entry# 3102
(3E)-3-tert-Butyl-7,7-dimethyl-3-octene-1,5-diyne #

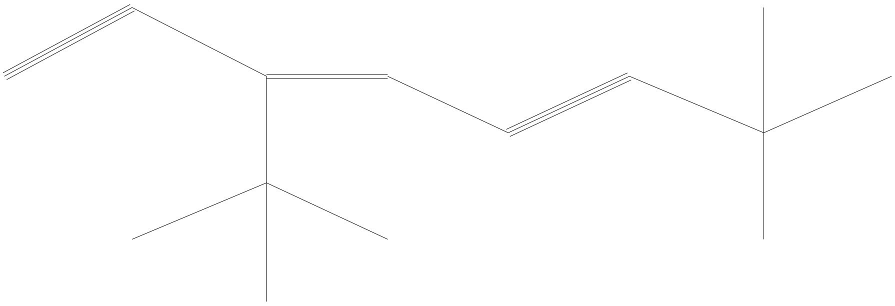

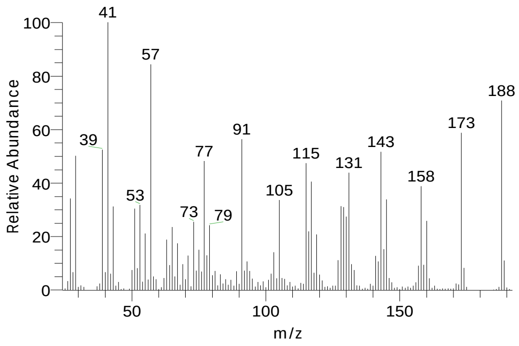

3-TERT-BUTYL-7,7-DIMETHYL-3-OCTENE-1,5-DIYNE
Formula C14H20, MW 188, CAS# NA, Entry# 361090

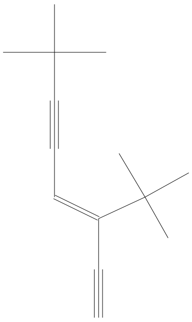

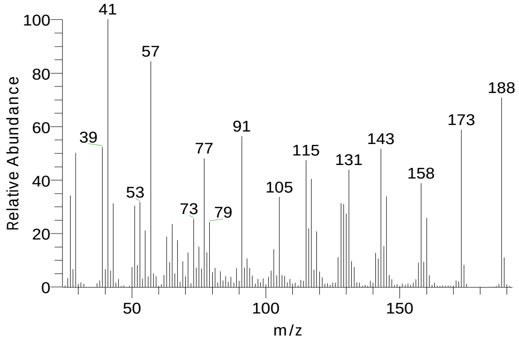

My GC-MS Report

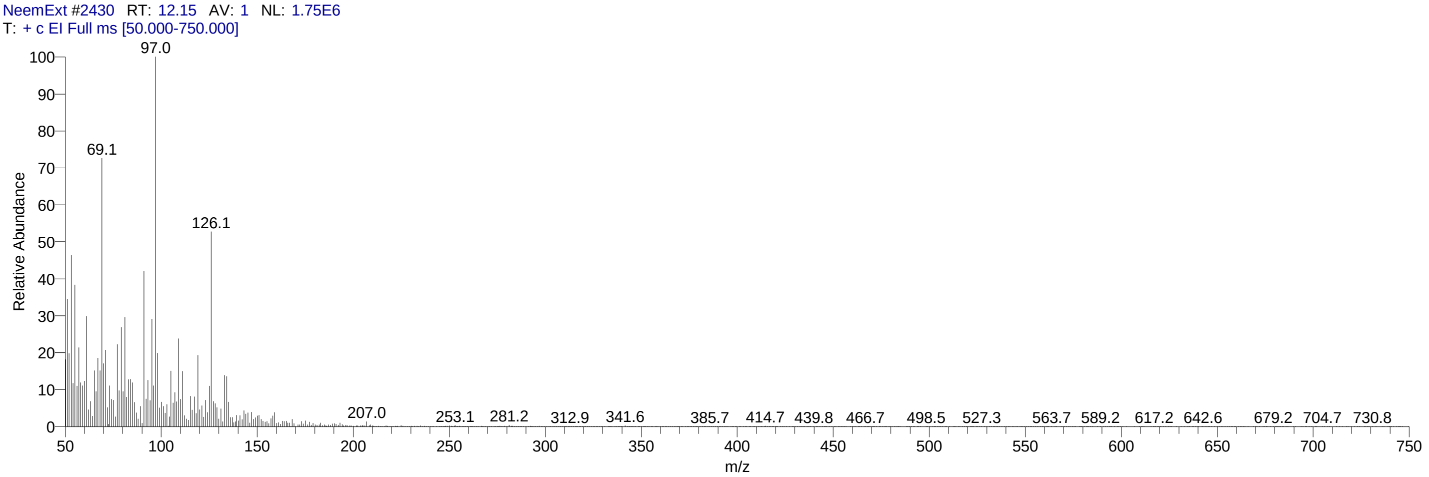

RT	Compound Name	Area %	MF	Molecular Formula	Molecular Weight	Cas #	Library
12.15	Retinal	1.24	794	C20H28O	284	116-31-4	mainlib
12.15	RETINAL	1.24	794	C20H28O	284	116-31-4	WileyRegistry8e
12.15	6-Acetyl-á-d-mannose	1.24	756	C8H14O7	222	NA	mainlib
12.15	Nona-2,3-dienoic acid, ethyl ester	1.24	722	C11H18O2	182	NA	mainlib
12.15	NONA-2,3-DIENOIC ACID ETHYL ESTER	1.24	722	C11H18O2	182	NA	WileyRegistry8e

Compound Structure | Hit Spectrum

Retinal
Formula C20H28O, MW 284, CAS# 116-31-4, Entry# 60749
Vitamin A aldehyde

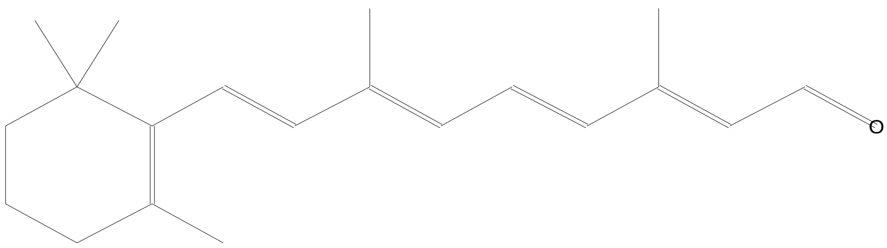

SI 699, RSI 794, mainlib, Entry# 60749, CAS# 116-31-4, Retinal

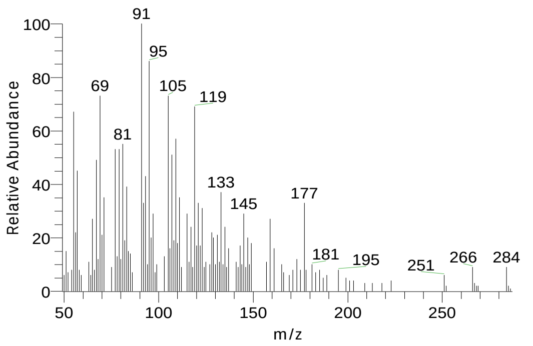

RETINAL
Formula C20H28O, MW 284, CAS# 116-31-4, Entry# 175130
à-RETINENE

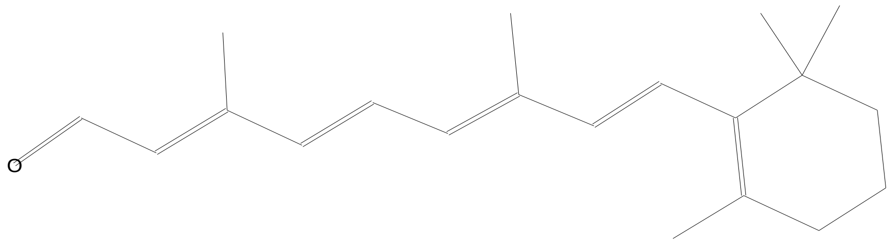

SI 698, RSI 794, WileyRegistry8e, Entry# 175130, CAS# 116-31-4, RETINAL

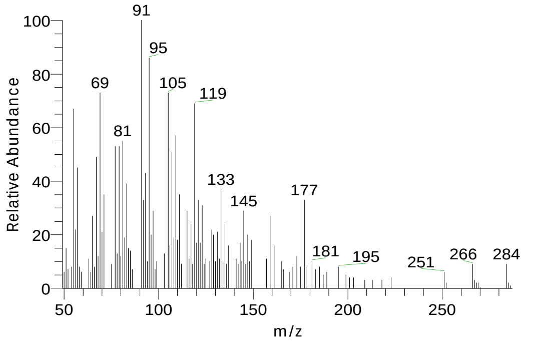

My GC-MS Report

Compound Structure	Hit Spectrum

6-Acetyl-á-d-mannose
Formula C8H14O7, MW 222, CAS# NA, Entry# 5869
6-O-Acetylhexopyranose #

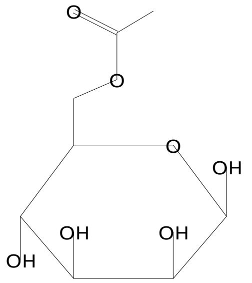

SI 694, RSI 756, mainlib, Entry# 5869, CAS# NA, 6-Acetyl-á-d-mannose

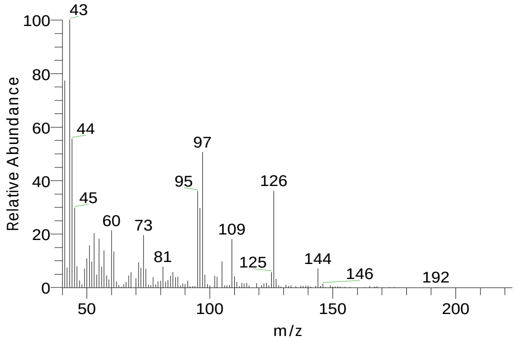

Nona-2,3-dienoic acid, ethyl ester
Formula C11H18O2, MW 182, CAS# NA, Entry# 482
Ethyl 2,3-nonadienoate #

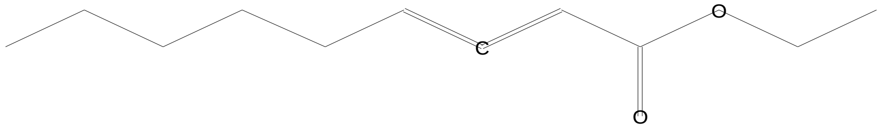

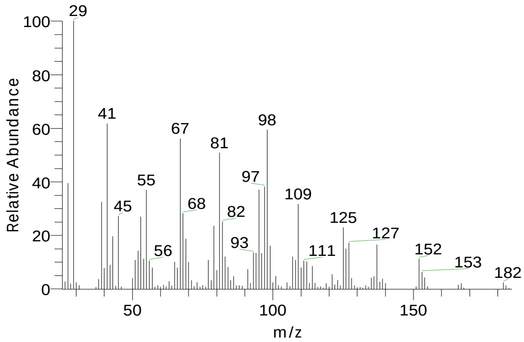

NONA-2,3-DIENOIC ACID ETHYL ESTER
Formula C11H18O2, MW 182, CAS# NA, Entry# 374502
ETHYL 2,3-NONADIENOATE

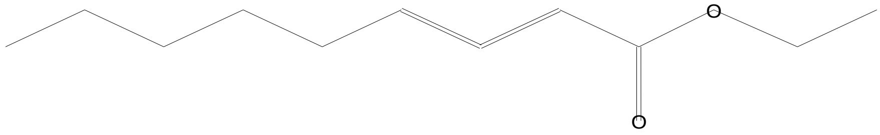

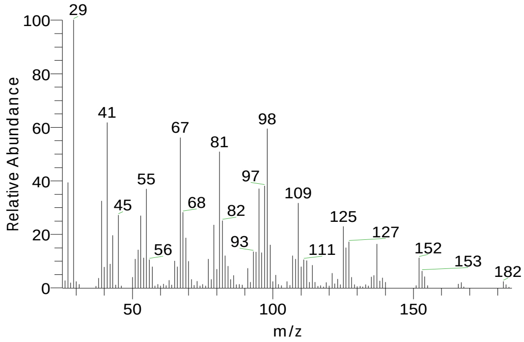

NeemExt #2550 RT: 12.55 AV: 1 NL: 1.63E6
T: + c EI Full ms [50.000-750.000]

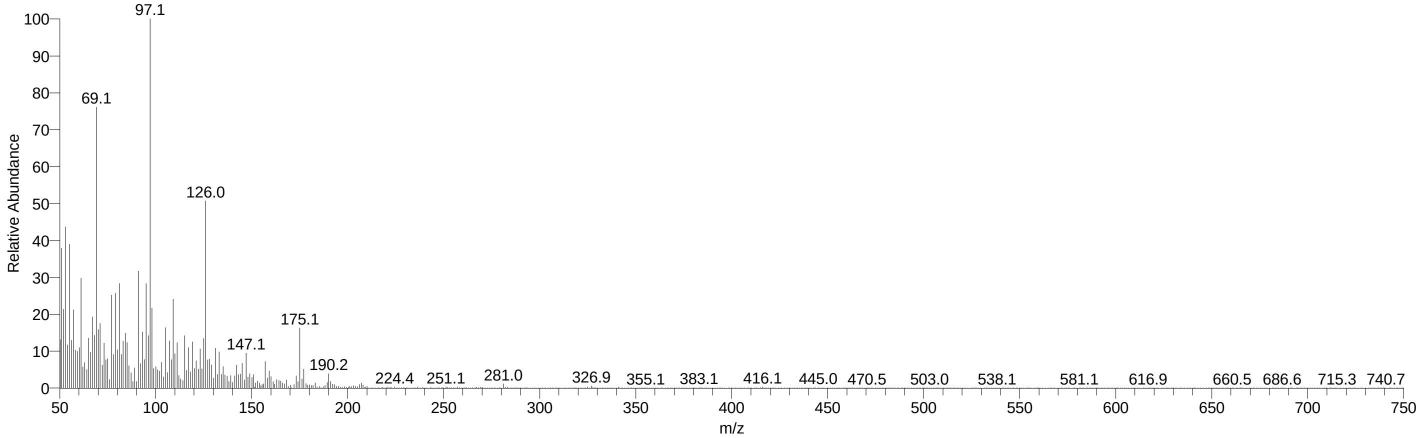

RT	Compound Name	Area %	MF	Molecular Formula	Molecular Weight	Cas #	Library
12.55	10-Methyl-8-tetradecen-1-ol acetate	1.30	694	C17H32O2	268	NA	mainlib
12.55	6-Acetyl-á-d-mannose	1.30	747	C8H14O7	222	NA	mainlib
12.55	16-Nitrobicyclo[10.4.0]hexadecan-1-ol-13-one	1.30	670	C16H27NO4	297	79880-69-6	mainlib

My GC-MS Report

RT	Compound Name	Area %	MF	Molecular Formula	Molecular Weight	Cas #	Library
12.55	4A-HYDROXY-4-NITROTETRADECAHYDROBENZO[A]CYCLODODECEN-1(2H)-ONE	1.30	670	C16H27NO4	297	79880-69-6	WileyRegistry8e
12.55	1b,5,5,6a-Tetramethyl-octahydro-1-oxa-cyclopropa[a]inden-6-one	1.30	740	C13H20O2	208	NA	mainlib

Compound Structure | Hit Spectrum

10-Methyl-8-tetradecen-1-ol acetate
Formula C17H32O2, MW 268, CAS# NA, Entry# 7048
(8E)-10-Methyl-8-tetradecenyl acetate #

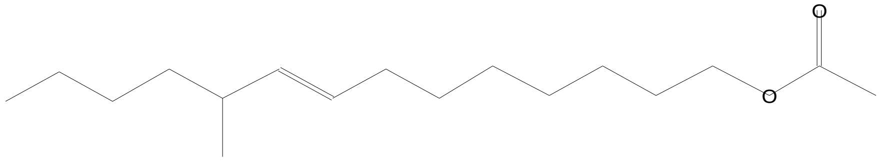

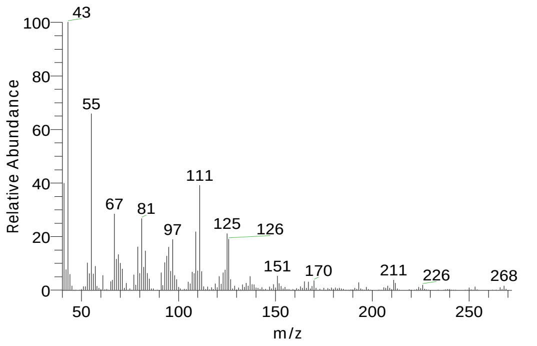

SI 675, RSI 747, mainlib, Entry# 5869, CAS# NA, 6-Acetyl-á-d-mannose

6-Acetyl-á-d-mannose
Formula C8H14O7, MW 222, CAS# NA, Entry# 5869
6-O-Acetylhexopyranose #

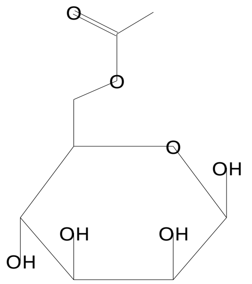

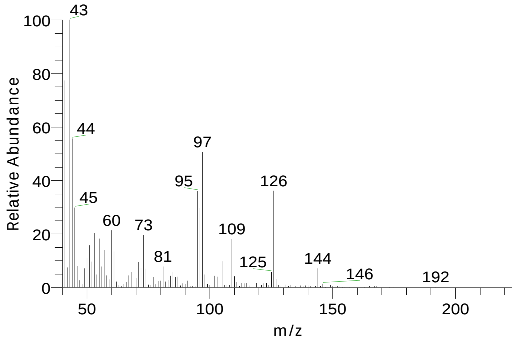

16-Nitrobicyclo[10.4.0]hexadecan-1-ol-13-one
Formula C16H27NO4, MW 297, CAS# 79880-69-6, Entry# 21183
4a-Hydroxy-4-nitrotetradecahydrobenzo[a]cyclododecen-1(2H)-one #

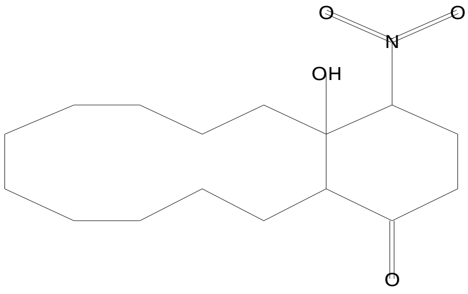

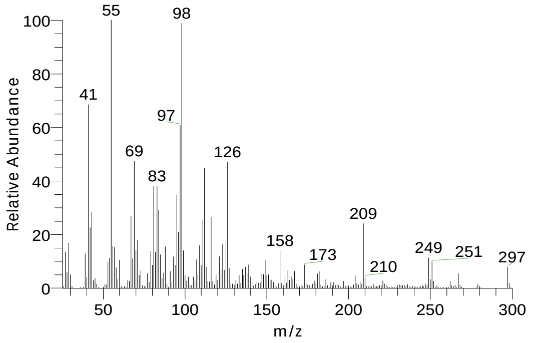

4A-HYDROXY-4-NITROTETRADECAHYDROBENZO[A]CYCLODODECEN-1(2H)-ONE
Formula C16H27NO4, MW 297, CAS# 79880-69-6, Entry# 364572

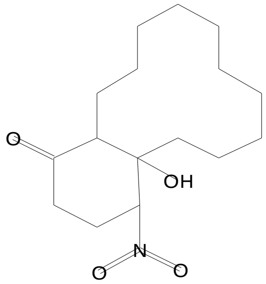

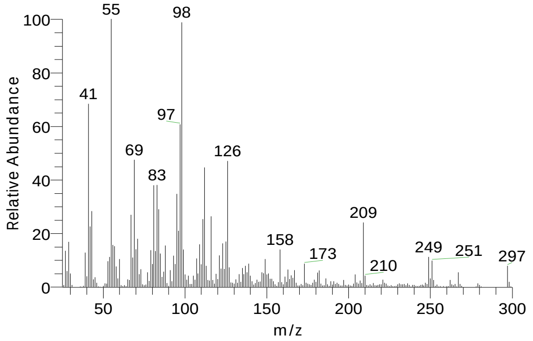

My GC-MS Report

Compound Structure | Hit Spectrum

1b,5,5,6a-Tetramethyl-octahydro-1-oxa-cyclopropa[a]inden-6-one
Formula C13H20O2, MW 208, CAS# NA, Entry# 2373
1b,5,5,6a-Tetramethyloctahydro-6H-indeno[1,2-b]oxiren-6-one #

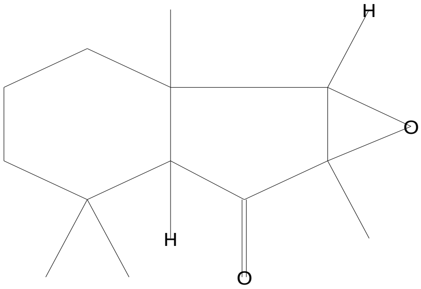

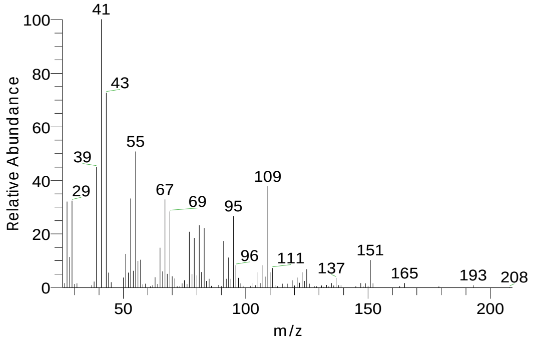

NeemExt #2711 RT: 13.09 AV: 1 NL: 1.61E6
T: + c EI Full ms [50.000-750.000]

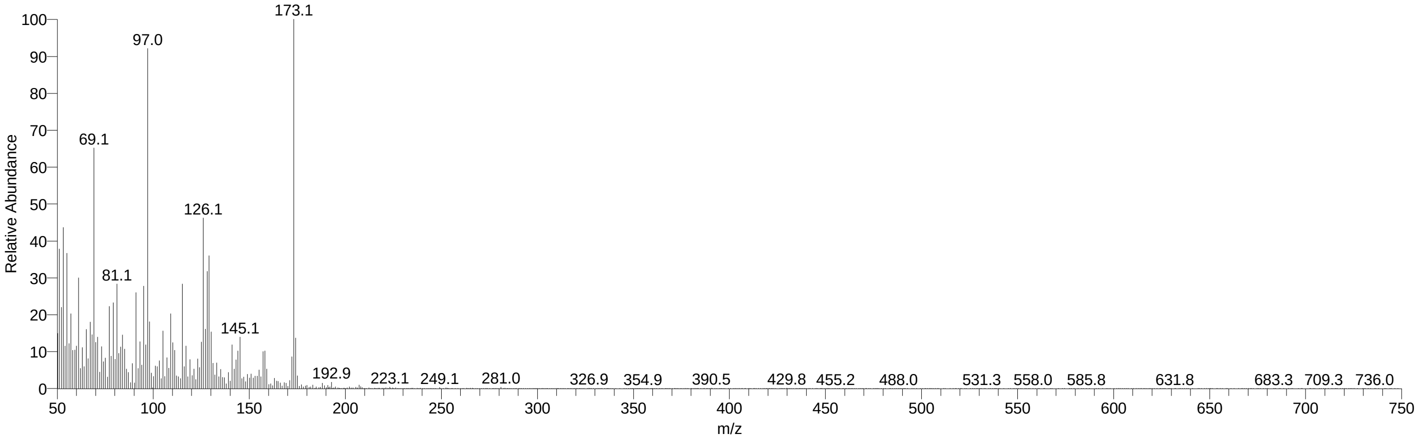

RT	Compound Name	Area %	MF	Molecular Formula	Molecular Weight	Cas #	Library
13.09	NAPHTHALENE, 1-NITRO-	3.56	717	C10H7NO2	173	86-57-7	WileyRegistry8e
13.09	2,9-Heptadecadiene-4,6-diyn-8-ol, (Z,E)-	3.56	696	C17H24O	244	50816-77-8	mainlib
13.09	(2Z,9E)-2,9-HEPTADECADIENE-4,6-DIYN-8-OL #	3.56	696	C17H24O	244	50816-77-8	WileyRegistry8e
13.09	1H-Inden-1-one, 2,3-dihydrotetramethyl-	3.56	665	C13H16O	188	89907-33-5	mainlib
13.09	2,2,3,3-TETRAMETHYL-1-INDANONE #	3.56	665	C13H16O	188	89907-33-5	WileyRegistry8e

Compound Structure | Hit Spectrum

NAPHTHALENE, 1-NITRO-
Formula C10H7NO2, MW 173, CAS# 86-57-7, Entry# 56365
1-NITRONAPHTHALENE

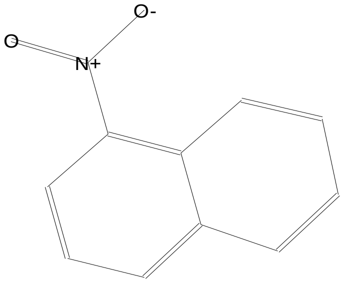

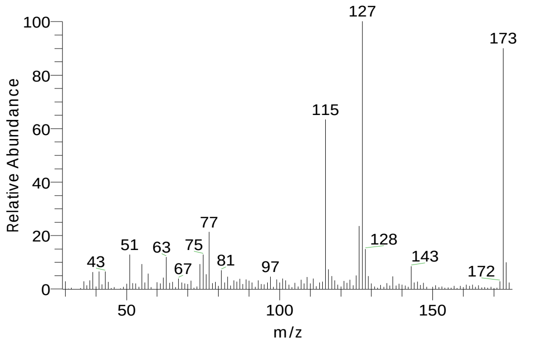

My GC-MS Report

Compound Structure | Hit Spectrum

2,9-Heptadecadiene-4,6-diyn-8-ol, (Z,E)-
Formula C17H24O, MW 244, CAS# 50816-77-8, Entry# 59012
(2Z,9E)-2,9-Heptadecadiene-4,6-diyn-8-ol #

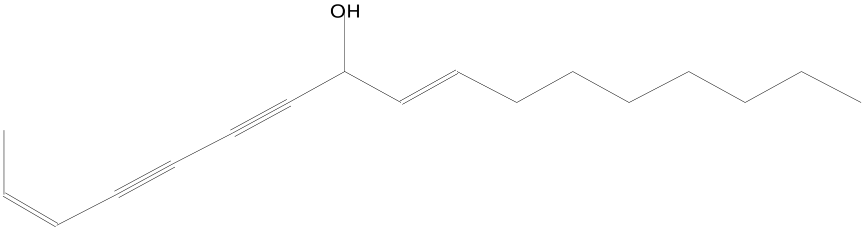

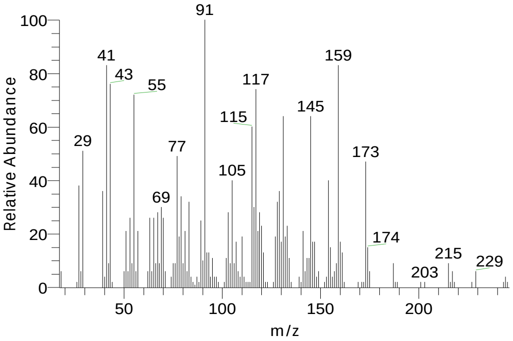

(2Z,9E)-2,9-HEPTADECADIENE-4,6-DIYN-8-OL #
Formula C17H24O, MW 244, CAS# 50816-77-8, Entry# 134143
(2Z,9E)-2,9-HEPTADECADIENE-4,6-DIYN-8-OL

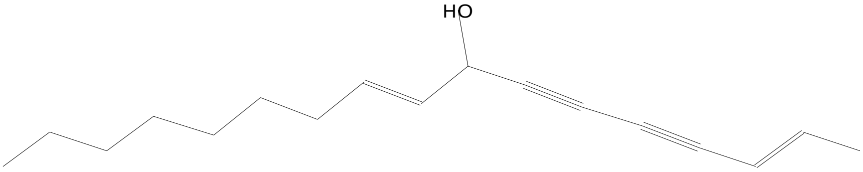

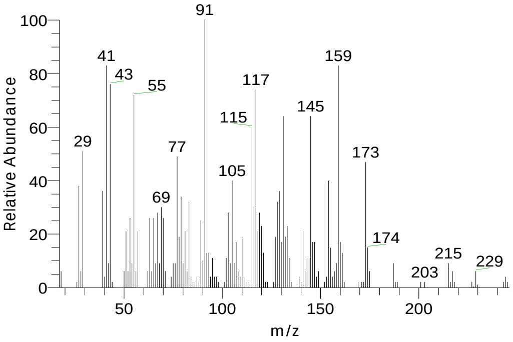

1H-Inden-1-one, 2,3-dihydrotetramethyl-
Formula C13H16O, MW 188, CAS# 89907-33-5, Entry# 162184
2,2,3,3-Tetramethyl-1-indanone #

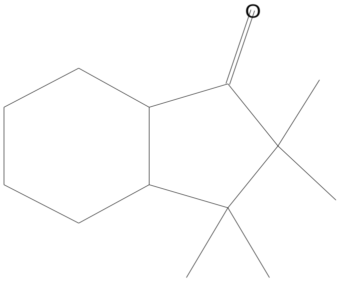

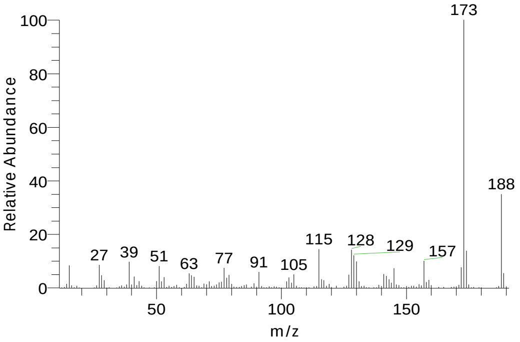

2,2,3,3-TETRAMETHYL-1-INDANONE #
Formula C13H16O, MW 188, CAS# 89907-33-5, Entry# 71727
1H-INDEN-1-ONE, 2,3-DIHYDROTETRAMETHYL-

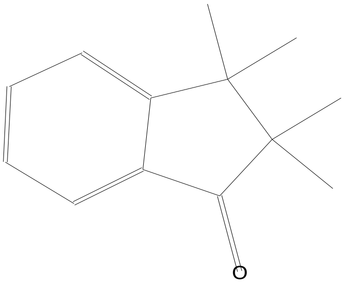

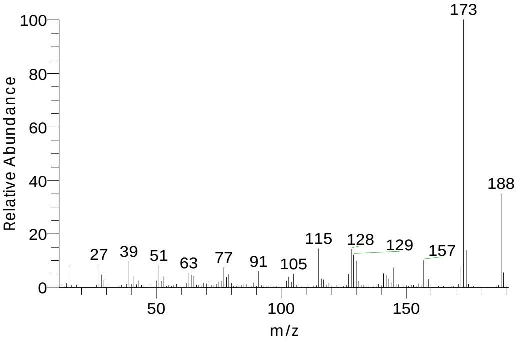

My GC-MS Report

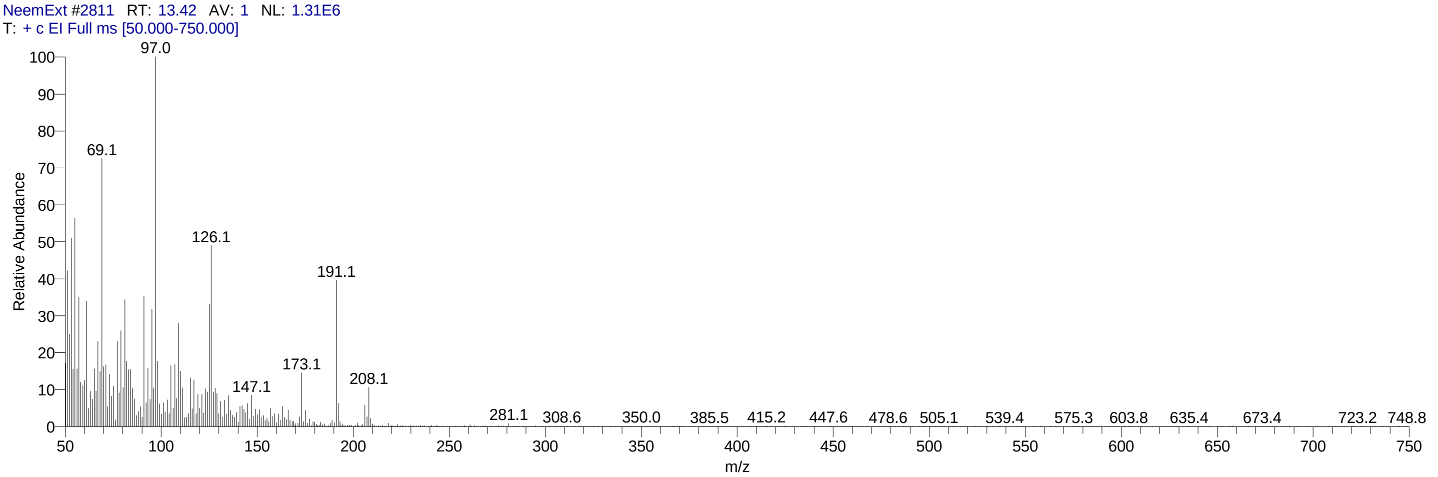

RT	Compound Name	Area %	MF	Molecular Formula	Molecular Weight	Cas #	Library
13.42	1-Oxaspiro[2.5]octane, 5,5-dimethyl-4-(3-methyl-1,3-butadienyl)-	1.16	709	C14H22O	206	NA	mainlib
13.42	5,5-DIMETHYL-4-[3-METHYL-1,3-BUTADIENYL]-1-OXASPIRO[2.5]OCTANE	1.16	709	C14H22O	206	NA	WileyRegistry8e
13.42	11-Isopropylidene-tricyclo[4.3.1.1(2,5)]undecan-10-ol	1.16	709	C14H22O	206	NA	mainlib
13.42	11-(1-METHYLETHYLIDENE)TRICYCLO[4.3.1.1~2,5~]UNDECAN-10-OL	1.16	709	C14H22O	206	NA	WileyRegistry8e
13.42	16-Nitrobicyclo[10.4.0]hexadecan-1-ol-13-one	1.16	659	C16H27NO4	297	79880-69-6	mainlib

Compound Structure | Hit Spectrum

1-Oxaspiro[2.5]octane, 5,5-dimethyl-4-(3-methyl-1,3-butadienyl)-
Formula C14H22O, MW 206, CAS# NA, Entry# 3796
5,5-Dimethyl-4-[(1E)-3-methyl-1,3-butadienyl]-1-oxaspiro[2.5]octane #

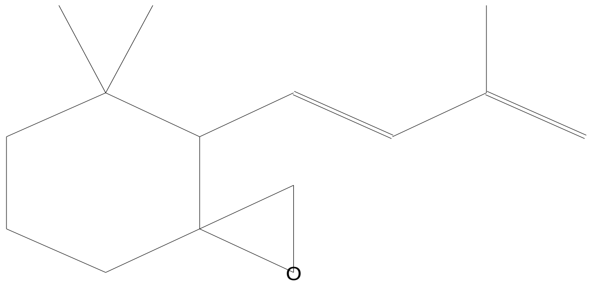

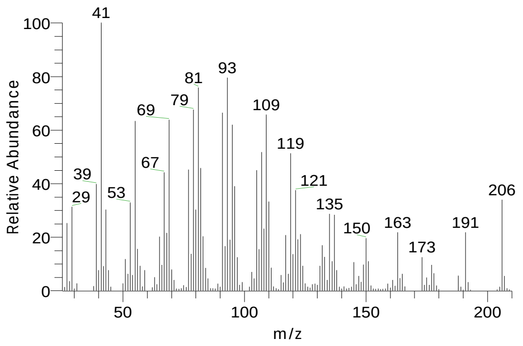

5,5-DIMETHYL-4-[3-METHYL-1,3-BUTADIENYL]-1-OXASPIRO[2.5]OCTANE
Formula C14H22O, MW 206, CAS# NA, Entry# 365893

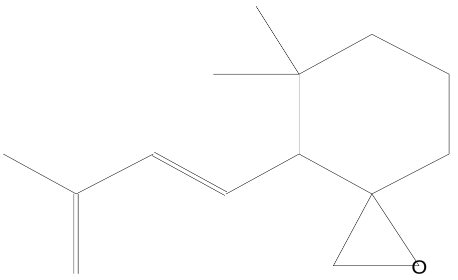

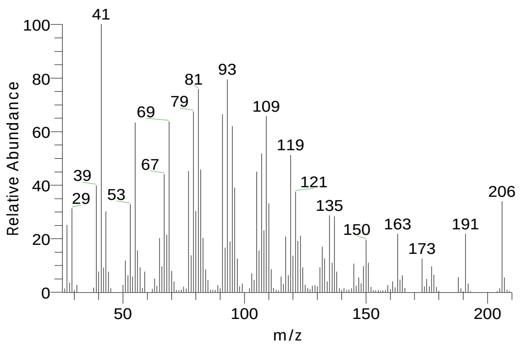

My GC-MS Report

Compound Structure	Hit Spectrum

11-Isopropylidene-tricyclo[4.3.1.1(2,5)]undecan-10-ol
Formula C14H22O, MW 206, CAS# NA, Entry# 10974
$:28GGDOGXNULLPYLK-UHFFFAOYSA-N

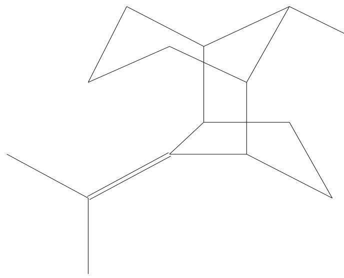

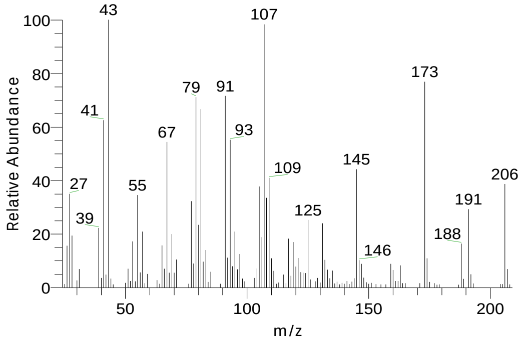

11-(1-METHYLETHYLIDENE)TRICYCLO[4.3.1.1~2,5~]UNDECAN-10-OL
Formula C14H22O, MW 206, CAS# NA, Entry# 366886
11-ISOPROPYLIDENE-TRICYCLO[4.3.1.1 2,5]UNDECAN-10-OL

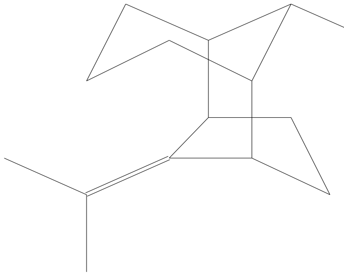

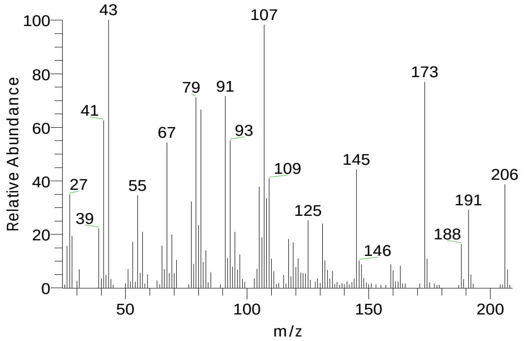

16-Nitrobicyclo[10.4.0]hexadecan-1-ol-13-one
Formula C16H27NO4, MW 297, CAS# 79880-69-6, Entry# 21183
4a-Hydroxy-4-nitrotetradecahydrobenzo[a]cyclododecen-1(2H)-one #

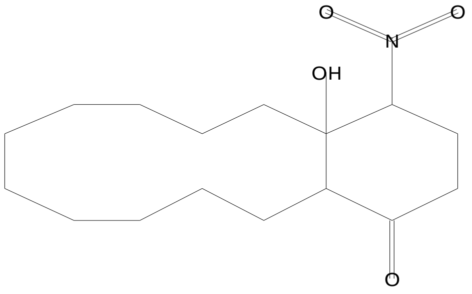

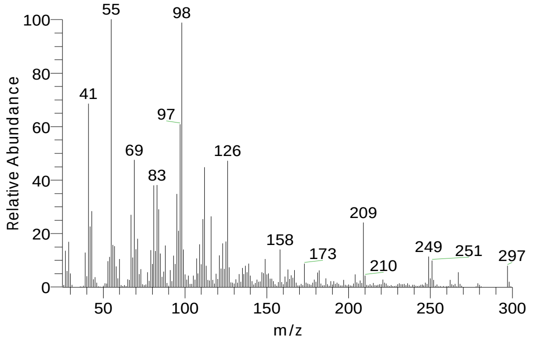

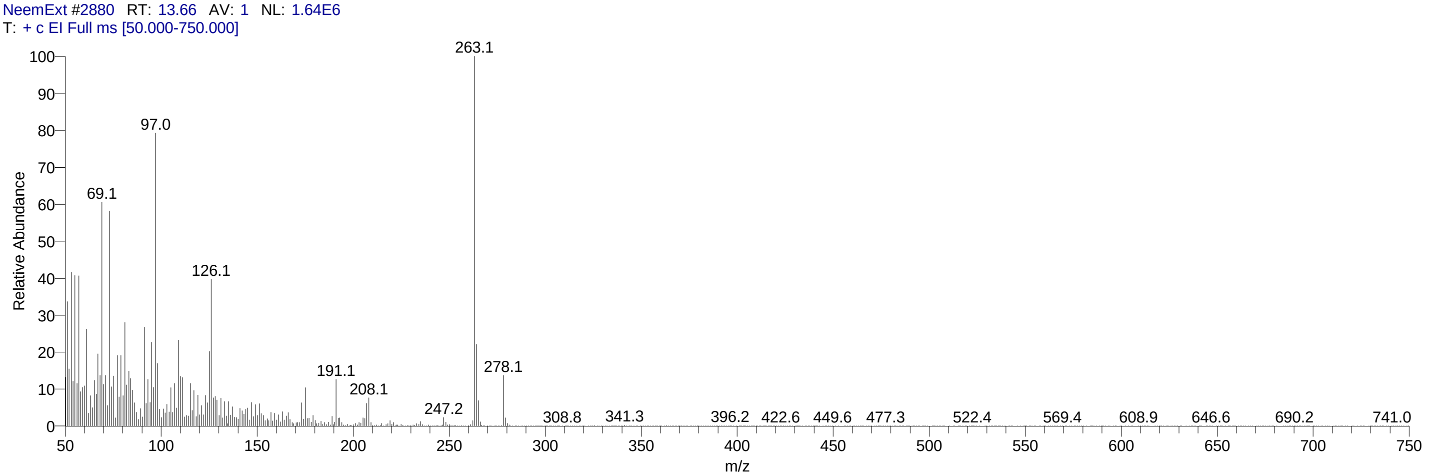

RT	Compound Name	Area %	MF	Molecular Formula	Molecular Weight	Cas #	Library
13.66	4-PENTEN-1-ONE, 2-[BIS(METHYLTHIO)METHYLEN E]-4-METHYL-1-PHENYL-	1.93	873	C15H18OS2	278	116145 -44-9	WileyRegi stry8e

My GC-MS Report

RT	Compound Name	Area %	MF	Molecular Formula	Molecular Weight	Cas #	Library
13.66	1H,4H,5H,8H-3A,4A,7A,8A-TETRAAZACYCLOPENTA[DEF]FLUORENE, HEXAHYDRO-2,2,4,6,6,8-HEXAMETHYL-, (4à,8à,8Bà,8C.A LPHA.)-	1.93	877	C16H30N4	278	122763-31-9	WileyRegistry8e
13.66	(+-)-5-HYDROXY-6-(1-HYDROXYETHYL)-2,7-DIMETHOXYNAPHTHOQUINONE	1.93	860	C14H14O6	278	NA	WileyRegistry8e
13.66	17-OXAANDROSTAN-3-OL, (3á,5à)-	1.93	845	C18H30O2	278	83632-45-5	WileyRegistry8e
13.66	4-[(4-AMINOPHENYL)SULFONYL]-1,2-BENZENEDIAMINE #	1.93	780	C12H13N3O2S	263	17828-44-3	WileyRegistry8e

Compound Structure	Hit Spectrum

4-PENTEN-1-ONE, 2-[BIS(METHYLTHIO)METHYLENE]-4-METHYL-1-PHENYL-
Formula C15H18OS2, MW 278, CAS# 116145-44-9, Entry# 168414
2-BIS(METHYLTHIO)METHYLENE-1-PHRENYL-4-METHYL-4-PENTEN-1-ONE

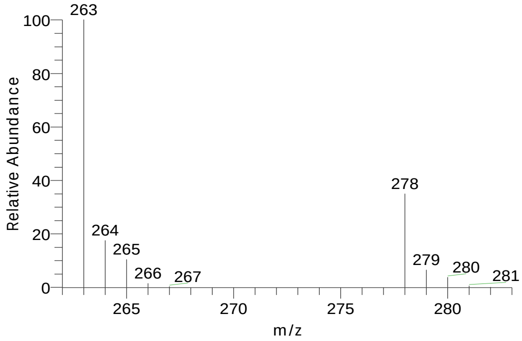

Formula C16H30N4, MW 278, CAS# 122763-31-9, Entry# 168749

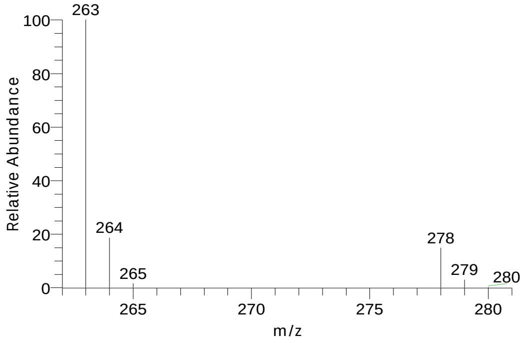

(+-)-5-HYDROXY-6-(1-HYDROXYETHYL)-2,7-DIMETHOXYNAPHTHOQUINONE
Formula C14H14O6, MW 278, CAS# NA, Entry# 168333

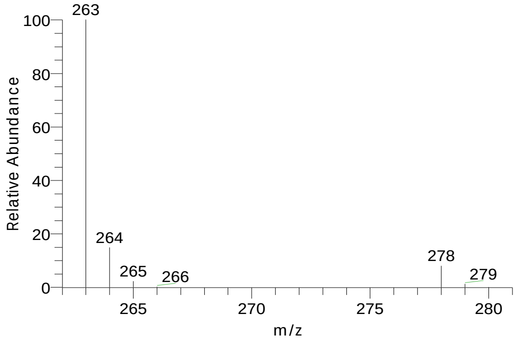

My GC-MS Report

Compound Structure | Hit Spectrum

17-OXAANDROSTAN-3-OL, (3á,5à)-
Formula C18H30O2, MW 278, CAS# 83632-45-5, Entry# 169047
17-OXA-5à-ANDROSTAN-3á-OL

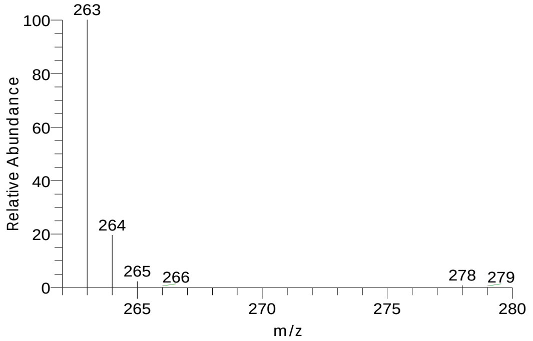

4-[(4-AMINOPHENYL)SULFONYL]-1,2-BENZENEDIAMINE #
Formula C12H13N3O2S, MW 263, CAS# 17828-44-3, Entry# 153292
3,4,4'-TRIAMINODIPHENYLSULFONE

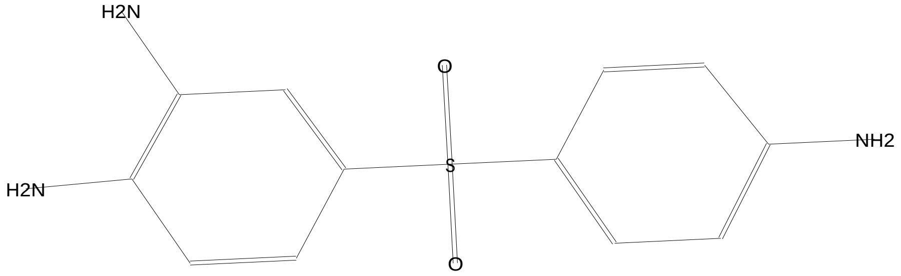

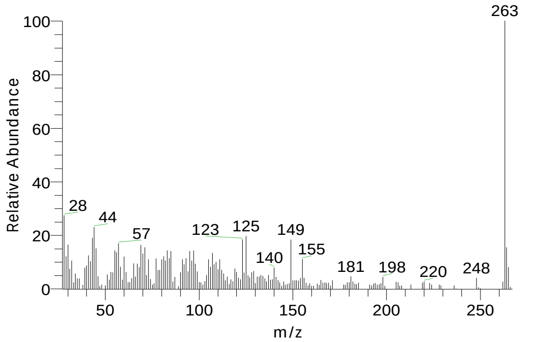

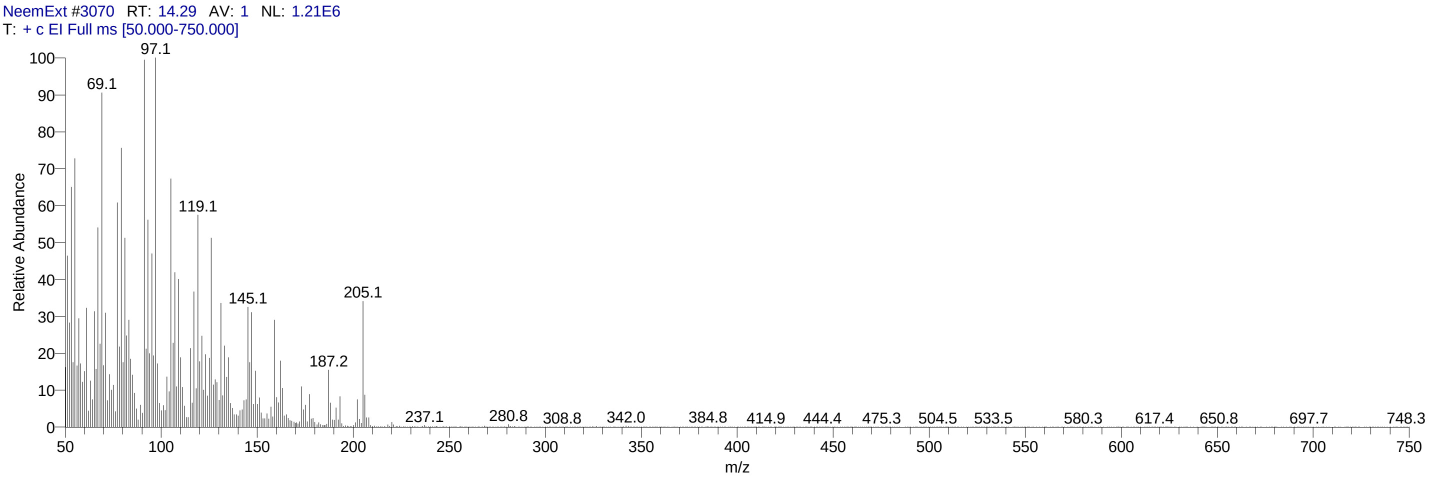

RT	Compound Name	Area %	MF	Molecular Formula	Molecular Weight	Cas #	Library
14.29	1H-Cycloprop[e]azulen-7-ol, decahydro-1,1,7-trimethyl-4-methylene-, [1ar-(1aà,4aà,7á,7aá,7bà)]-	6.01	881	C15H24O	220	6750-60-3	replib
14.29	Ledene oxide-(II)	6.01	795	C15H24O	220	NA	mainlib
14.29	1,4A,7,7-TETRAMETHYLDECAHYDROCYCLOPROPA[7,8]AZULENO[3A,4-B]OXIRENE	6.01	795	C15H24O	220	NA	WileyRegistry8e
14.29	Caryophyllene oxide	6.01	816	C15H24O	220	1139-30-6	replib
14.29	"NEOCLOVENOXID-ALKOHOL"	6.01	814	C15H24O	220	NA	WileyRegistry8e

My GC-MS Report

Compound Structure | Hit Spectrum

Formula C15H24O, MW 220, CAS# 6750-60-3, Entry# 1920
(1aR,4aR,7S,7aR,7bR)-1,1,7-Trimethyl-4-methylenedecahydro-1H-cyclopropa[e]azulen-7-ol

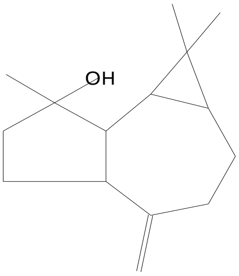

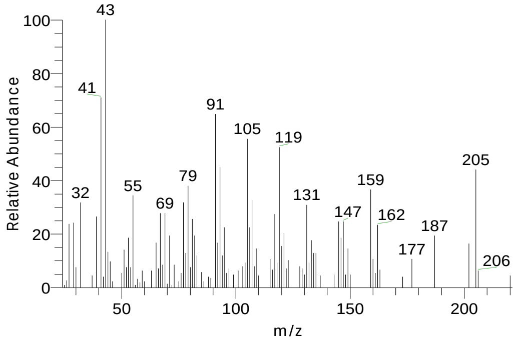

SI 783, RSI 795, mainlib, Entry# 5698, CAS# NA, Ledene oxide-(II)

Ledene oxide-(II)
Formula C15H24O, MW 220, CAS# NA, Entry# 5698
1,4a,7,7-Tetramethyldecahydrocyclopropa[7,8]azuleno[3a,4-b]oxirene #

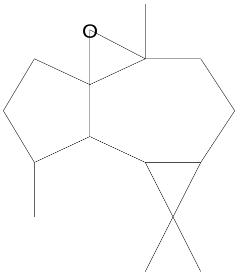

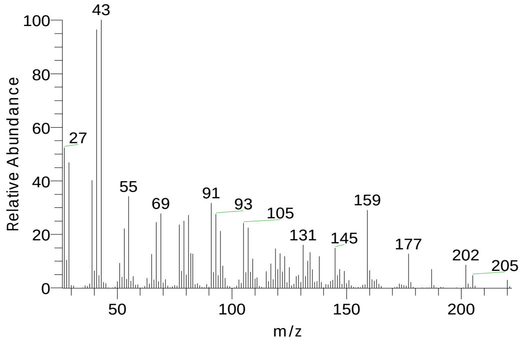

1,4A,7,7-TETRAMETHYLDECAHYDROCYCLOPROPA[7,8]AZULENO[3A,4-B]OXIRENE
Formula C15H24O, MW 220, CAS# NA, Entry# 381993
LEDENOXID-(II)

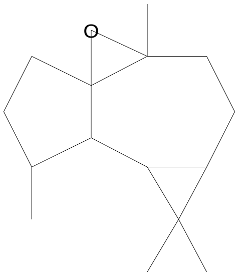

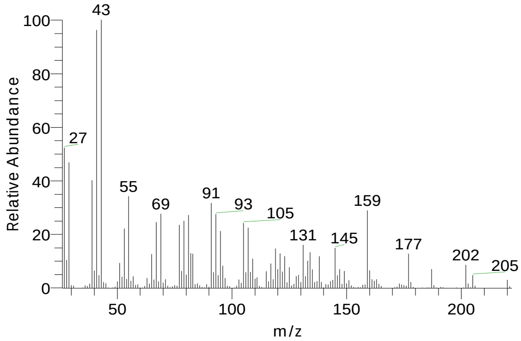

Caryophyllene oxide
Formula C15H24O, MW 220, CAS# 1139-30-6, Entry# 915

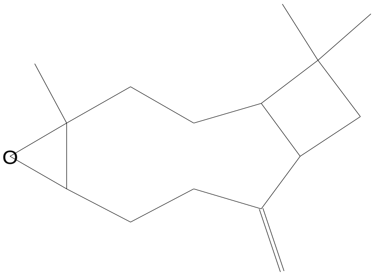

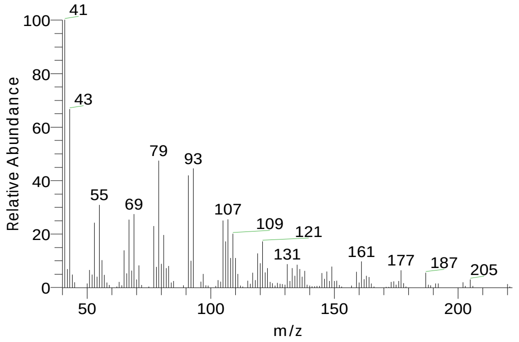

My GC-MS Report

Compound Structure | Hit Spectrum

"NEOCLOVENOXID-ALKOHOL"
Formula C15H24O, MW 220, CAS# NA, Entry# 381985

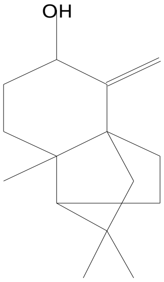

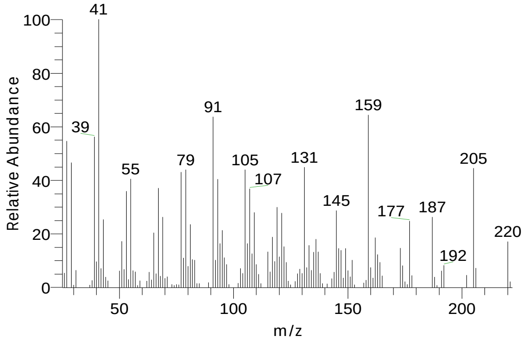

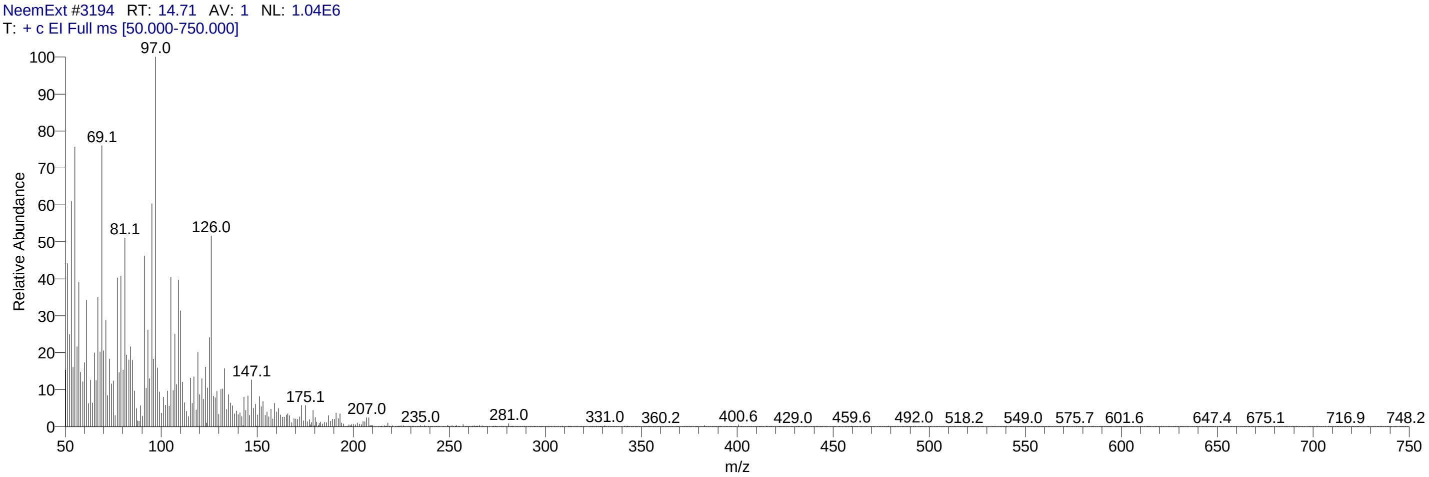

RT	Compound Name	Area %	MF	Molecular Formula	Molecular Weight	Cas #	Library
14.71	27-DEOXYWITHAFERINE-A	1.29	754	C28H38O5	454	NA	WileyRegistry8e
14.71	16-Nitrobicyclo[10.4.0]hexadecan-1-ol-13-one	1.29	691	C16H27NO4	297	79880-69-6	mainlib
14.71	4A-HYDROXY-4-NITROTETRADECAHYDROBENZO[A]CYCLODODECEN-1(2H)-ONE	1.29	691	C16H27NO4	297	79880-69-6	WileyRegistry8e
14.71	Caryophyllene oxide	1.29	743	C15H24O	220	1139-30-6	mainlib
14.71	4,12,12-TRIMETHYL-9-METHYLENE-5-OXATRICYCLO[8.2.0.0~4,6~]DODECANE	1.29	743	C15H24O	220	NA	WileyRegistry8e

Compound Structure | Hit Spectrum

27-DEOXYWITHAFERINE-A
Formula C28H38O5, MW 454, CAS# NA, Entry# 275441

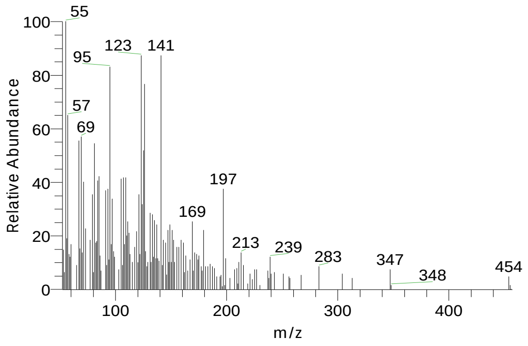

My GC-MS Report

Compound Structure | Hit Spectrum

16-Nitrobicyclo[10.4.0]hexadecan-1-ol-13-one
Formula C16H27NO4, MW 297, CAS# 79880-69-6, Entry# 21183
4a-Hydroxy-4-nitrotetradecahydrobenzo[a]cyclododecen-1(2H)-one #

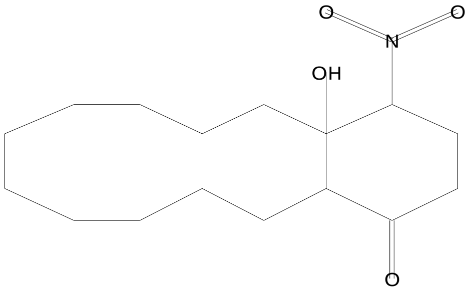

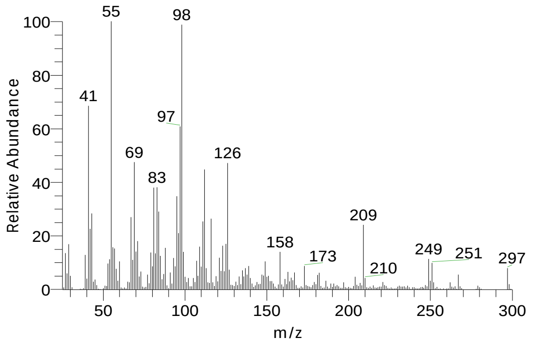

4A-HYDROXY-4-NITROTETRADECAHYDROBENZO[A]CYCLODODECEN-1(2H)-ONE
Formula C16H27NO4, MW 297, CAS# 79880-69-6, Entry# 364572

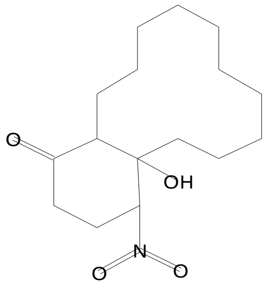

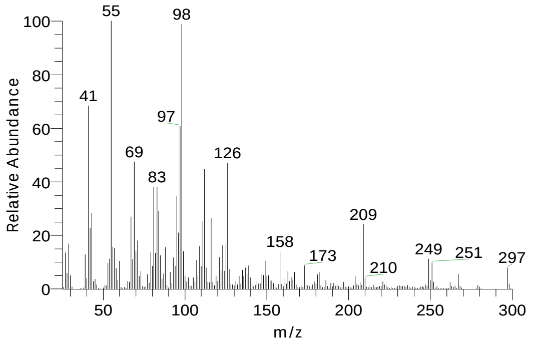

Caryophyllene oxide
Formula C15H24O, MW 220, CAS# 1139-30-6, Entry# 6247

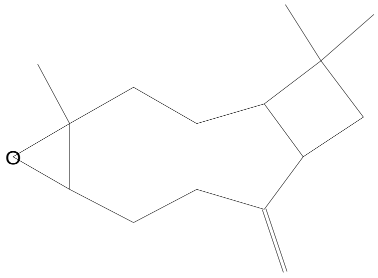

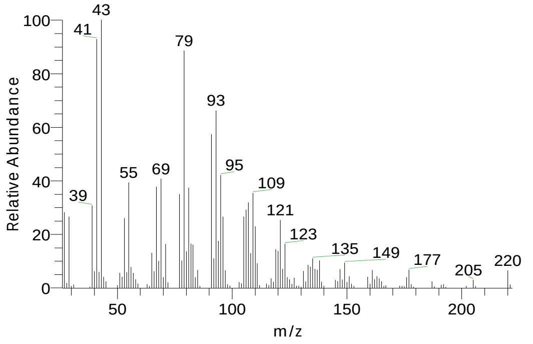

4,12,12-TRIMETHYL-9-METHYLENE-5-OXATRICYCLO[8.2.0.0~4,6~]DODECANE
Formula C15H24O, MW 220, CAS# NA, Entry# 388109
CARYOPHYLLENOXID

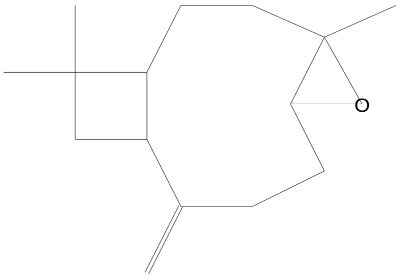

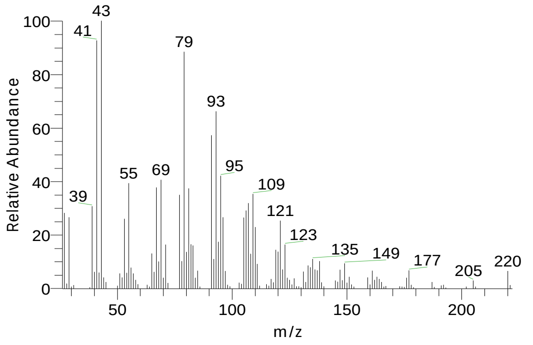

My GC-MS Report

NeemExt #3369 RT: 15.30 AV: 1 NL: 8.51E5
T: + c EI Full ms [50.000-750.000]

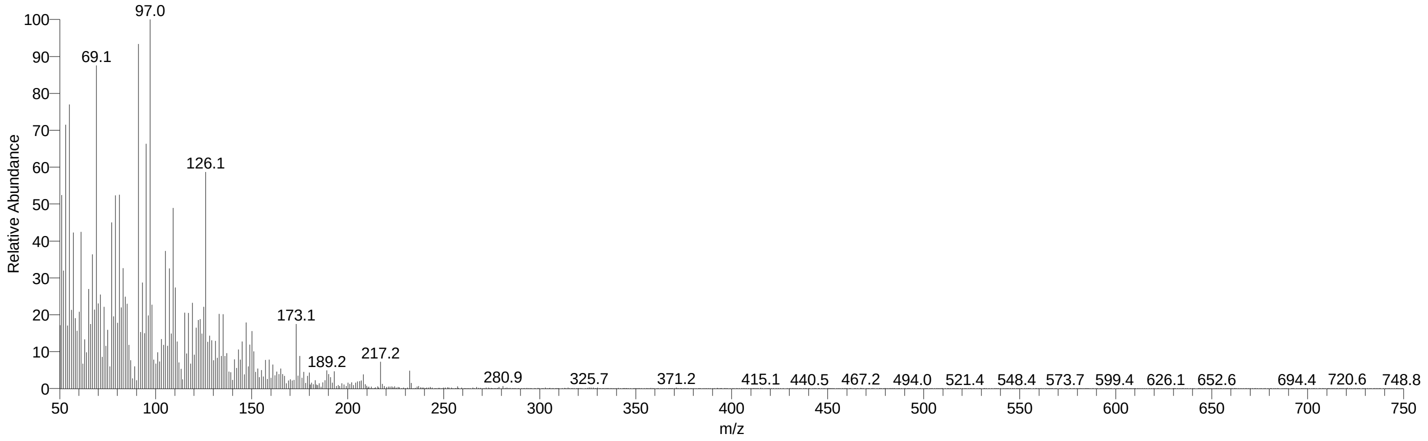

RT	Compound Name	Area %	MF	Molecular Formula	Molecular Weight	Cas #	Library
15.30	1-Heptatriacotanol	0.66	774	C37H76O	536	105794-58-9	mainlib
15.30	Retinal	0.66	859	C20H28O	284	116-31-4	mainlib
15.30	RETINAL	0.66	858	C20H28O	284	116-31-4	WileyRegistry8e
15.30	3H-Cyclodeca[b]furan-2-one, 4,9-dihydroxy-6-methyl-3,10-dimethylene-3a,4,7,8,9,10,11,11a-octahydro-	0.66	756	C15H20O4	264	NA	mainlib
15.30	3H-CYCLODECA[B]FURAN-2-ONE, 4,9-DIHYDROXY-6-METHYL-3,10-DIMETHYLENE-3A,4,7,8,9,10,11,11A-OCTAHYDRO-	0.66	756	C15H20O4	264	NA	WileyRegistry8e

Compound Structure | Hit Spectrum

1-Heptatriacotanol
Formula C37H76O, MW 536, CAS# 105794-58-9, Entry# 7279
1-Heptatriacontanol #

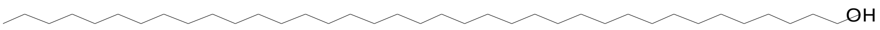

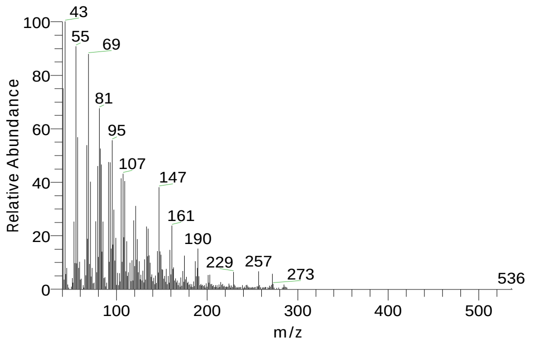

SI 740, RSI 859, mainlib, Entry# 60749, CAS# 116-31-4, Retinal

Retinal
Formula C20H28O, MW 284, CAS# 116-31-4, Entry# 60749
Vitamin A aldehyde

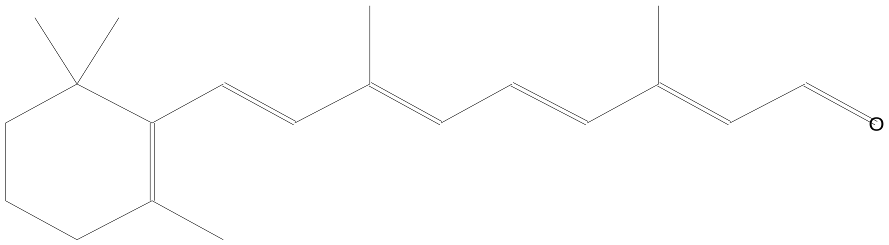

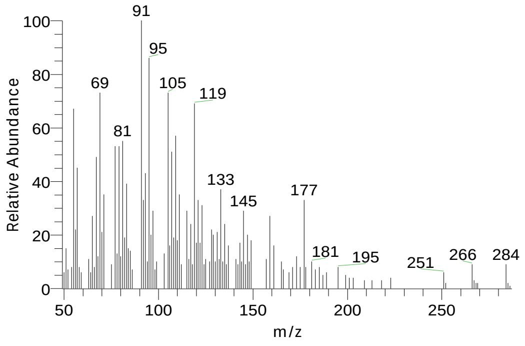

My GC-MS Report

Compound Structure | Hit Spectrum

RETINAL
Formula C20H28O, MW 284, CAS# 116-31-4, Entry# 175130
à-RETINENE

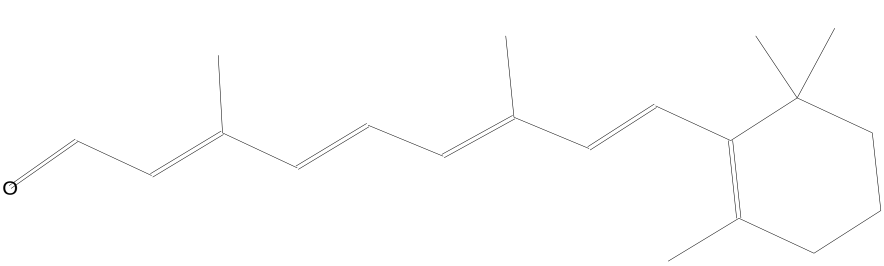

SI 740, RSI 858, WileyRegistry8e, Entry# 175130, CAS# 116-31-4, RETINAL

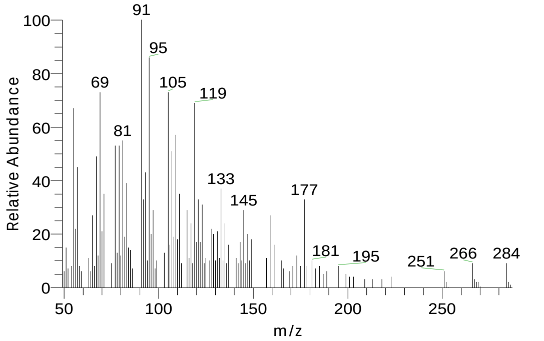

Formula C15H20O4, MW 264, CAS# NA, Entry# 2397
$:28KNEQPJSDSYNUHP-SOFGYWHQSA-N

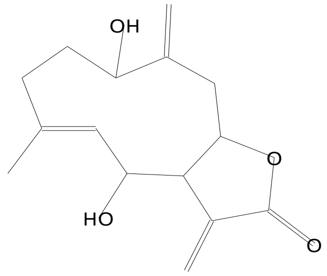

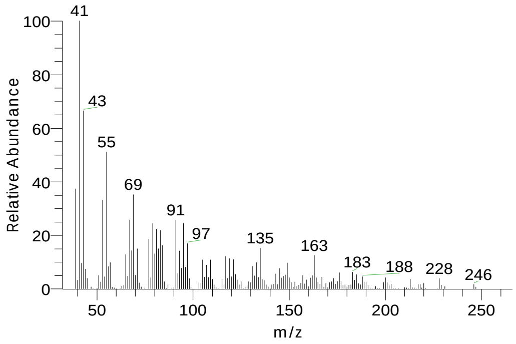

Formula C15H20O4, MW 264, CAS# NA, Entry# 329682

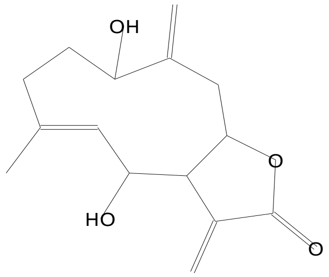

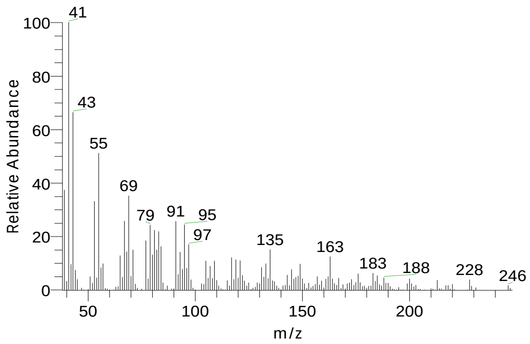

NeemExt #3386 RT: 15.35 AV: 1 NL: 1.03E6
T: + c EI Full ms [50.000-750.000]

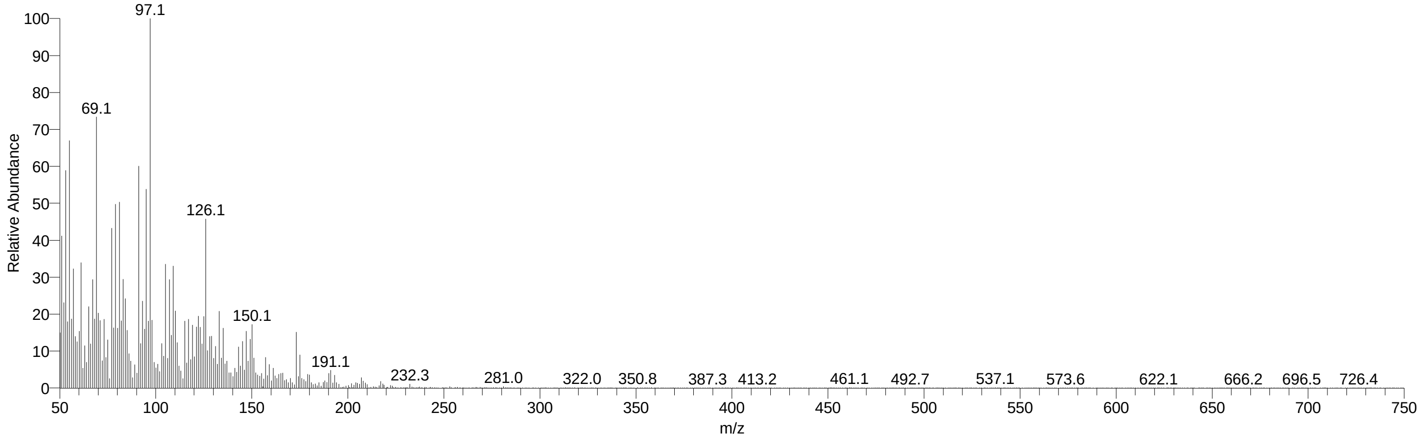

RT	Compound Name	Area %	MF	Molecular Formula	Molecular Weight	Cas #	Library
15.35	Retinal	0.97	853	C20H28O	284	116-31-4	mainlib
15.35	RETINAL	0.97	853	C20H28O	284	116-31-4	WileyRegistry8e

My GC-MS Report

RT	Compound Name	Area %	MF	Molecular Formula	Molecular Weight	Cas #	Library
15.35	1,3,5-TRIAZINE-2,4-DIAMINE, 6-CHLORO-N-ETHYL-	0.97	782	C5H8ClN5	173	1007-28-9	WileyRegistry8e
15.35	3H-Cyclodeca[b]furan-2-one, 4,9-dihydroxy-6-methyl-3,10-dimethylene-3a,4,7,8,9,10,11,11a-octahydro-	0.97	740	C15H20O4	264	NA	mainlib
15.35	3H-CYCLODECA[B]FURAN-2-ONE, 4,9-DIHYDROXY-6-METHYL-3,10-DIMETHYLENE-3A,4,7,8,9,10,11,11A-OCTAHYDRO-	0.97	740	C15H20O4	264	NA	WileyRegistry8e

Compound Structure — Hit Spectrum

Retinal
Formula C20H28O, MW 284, CAS# 116-31-4, Entry# 60749
Vitamin A aldehyde

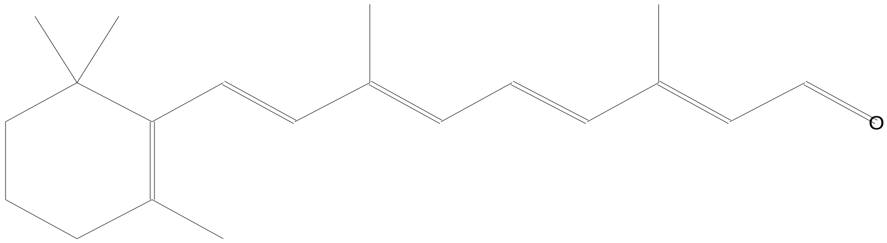

SI 743, RSI 853, mainlib, Entry# 60749, CAS# 116-31-4, Retinal

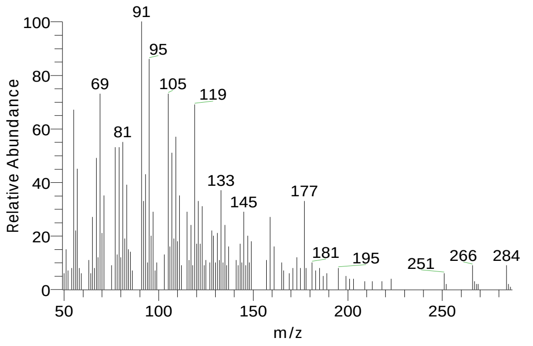

RETINAL
Formula C20H28O, MW 284, CAS# 116-31-4, Entry# 175130
à-RETINENE

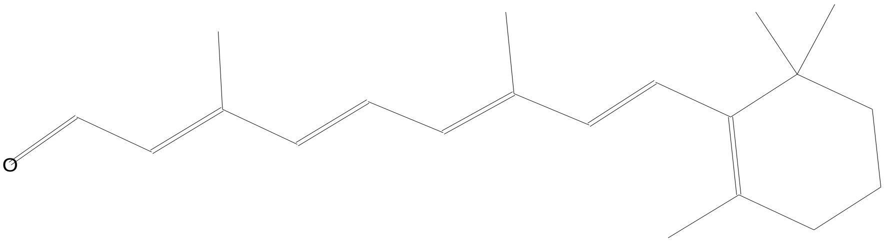

SI 742, RSI 853, WileyRegistry8e, Entry# 175130, CAS# 116-31-4, RETINAL

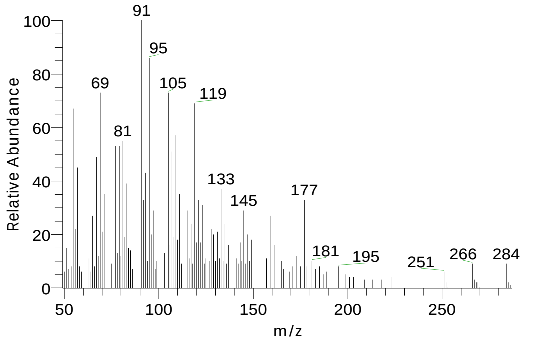

1,3,5-TRIAZINE-2,4-DIAMINE, 6-CHLORO-N-ETHYL-
Formula C5H8ClN5, MW 173, CAS# 1007-28-9, Entry# 56163
1,3, 5-TRIAZINE-2,4-DIAMINE, 6-CHLORO-N-ETHYL-

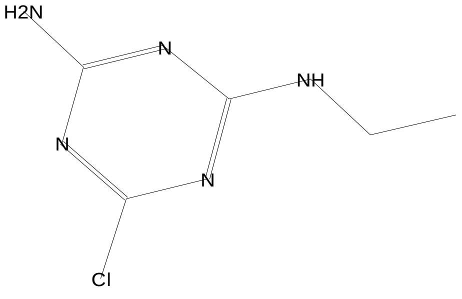

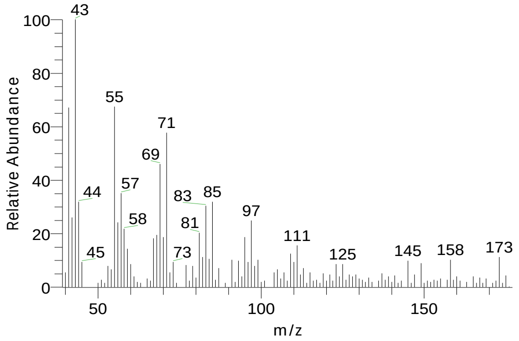

My GC-MS Report

Compound Structure | Hit Spectrum

Formula C15H20O4, MW 264, CAS# NA, Entry# 2397
$:28KNEQPJSDSYNUHP-SOFGYWHQSA-N

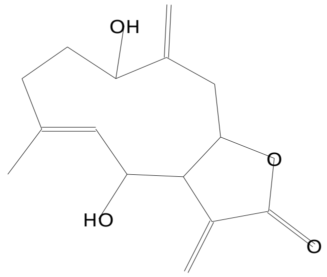

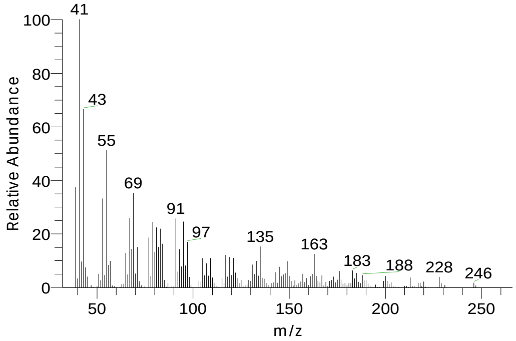

Formula C15H20O4, MW 264, CAS# NA, Entry# 329682

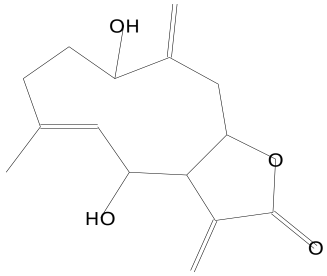

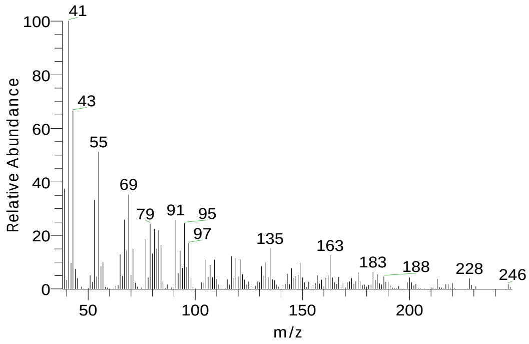

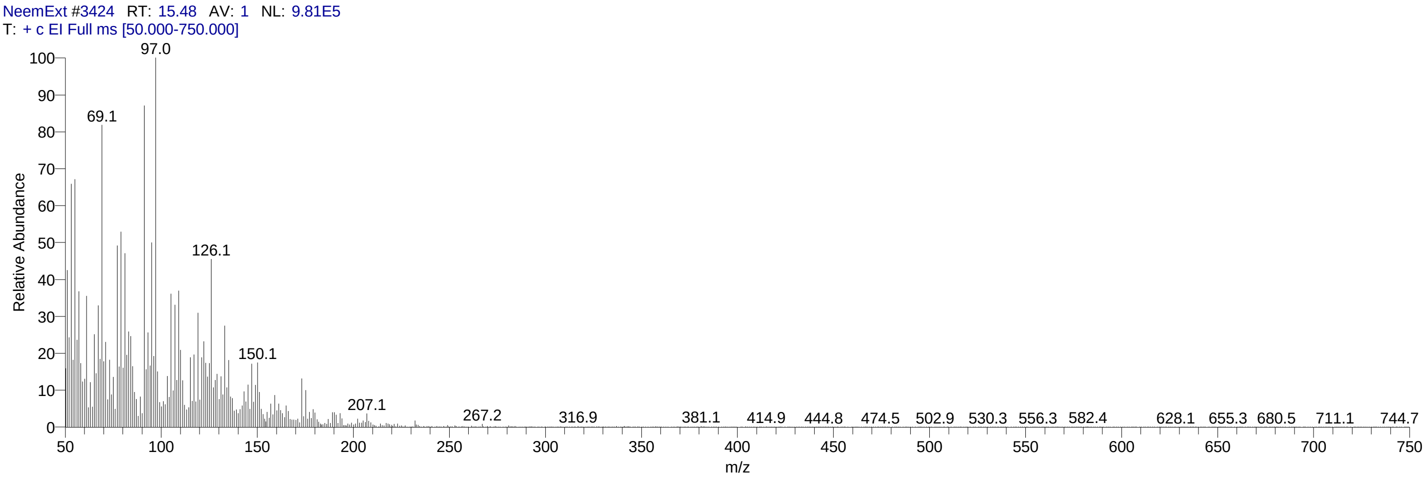

RT	Compound Name	Area %	MF	Molecular Formula	Molecular Weight	Cas #	Library
15.48	2-[4-methyl-6-(2,6,6-trimethylcyclohex-1-enyl)hexa-1,3,5-trienyl]cyclohex-1-en-1-carboxaldehyde	1.18	752	C23H32O	324	NA	mainlib
15.48	Retinal	1.18	860	C20H28O	284	116-31-4	mainlib
15.48	RETINAL	1.18	860	C20H28O	284	116-31-4	WileyRegistry8e
15.48	Picrotoxin	1.18	746	C15H16O6	292	124-87-8	mainlib
15.48	10,13-Octadecadiynoic acid, methyl ester	1.18	723	C19H30O2	290	18202-24-9	mainlib

My GC-MS Report

Compound Structure | Hit Spectrum

Formula C23H32O, MW 324, CAS# NA, Entry# 5913

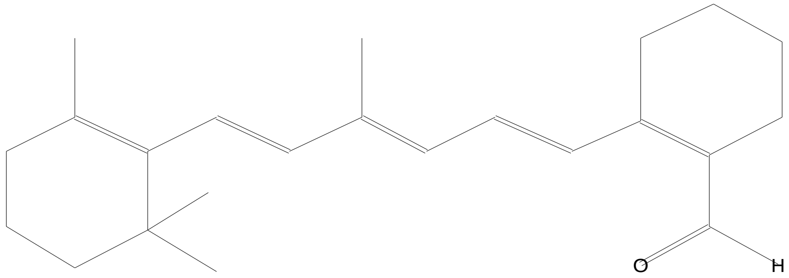

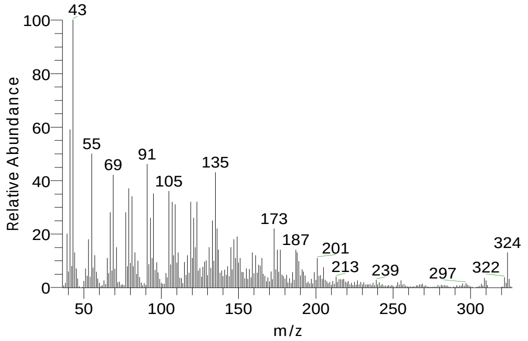

Retinal
Formula C20H28O, MW 284, CAS# 116-31-4, Entry# 60749
Vitamin A aldehyde

SI 747, RSI 860, mainlib, Entry# 60749, CAS# 116-31-4, Retinal

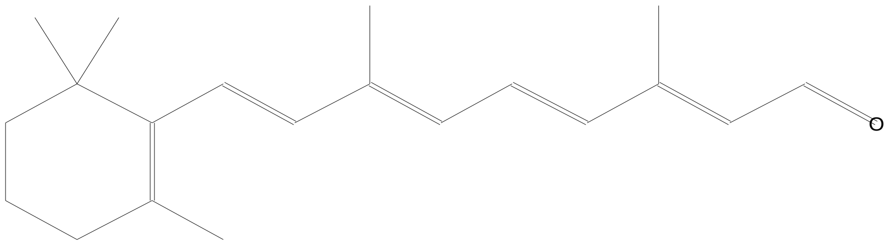

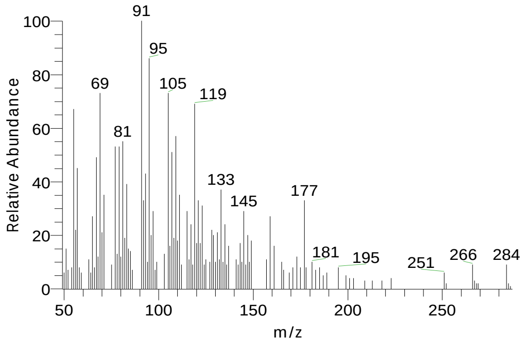

RETINAL
Formula C20H28O, MW 284, CAS# 116-31-4, Entry# 175130
à-RETINENE

SI 747, RSI 860, WileyRegistry8e, Entry# 175130, CAS# 116-31-4, RETINAL

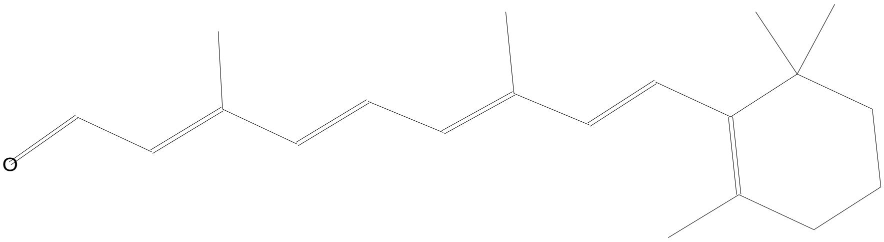

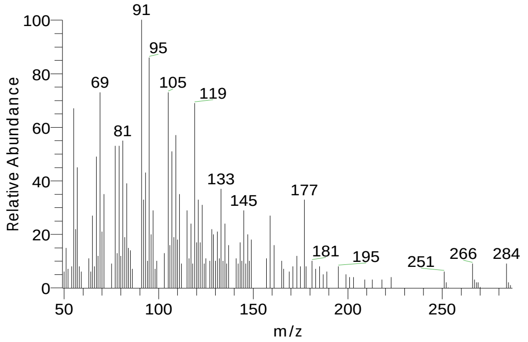

Picrotoxin
Formula C15H16O6, MW 292, CAS# 124-87-8, Entry# 67864

SI 729, RSI 746, mainlib, Entry# 67864, CAS# 124-87-8, Picrotoxin

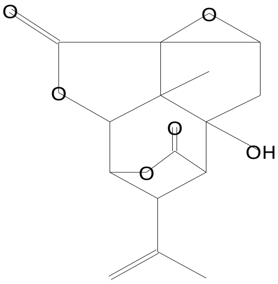

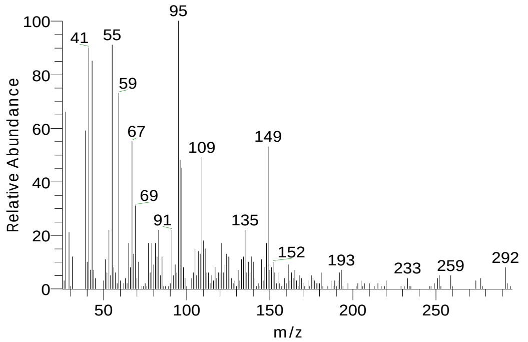

My GC-MS Report

Compound Structure | Hit Spectrum

10,13-Octadecadiynoic acid, methyl ester
Formula C19H30O2, MW 290, CAS# 18202-24-9, Entry# 60965
Methyl 10,13-octadecadiynoate #

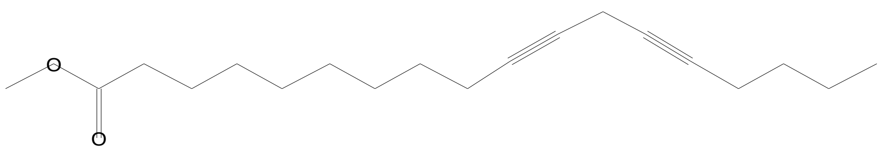

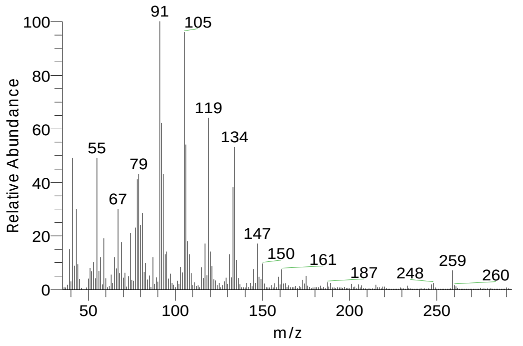

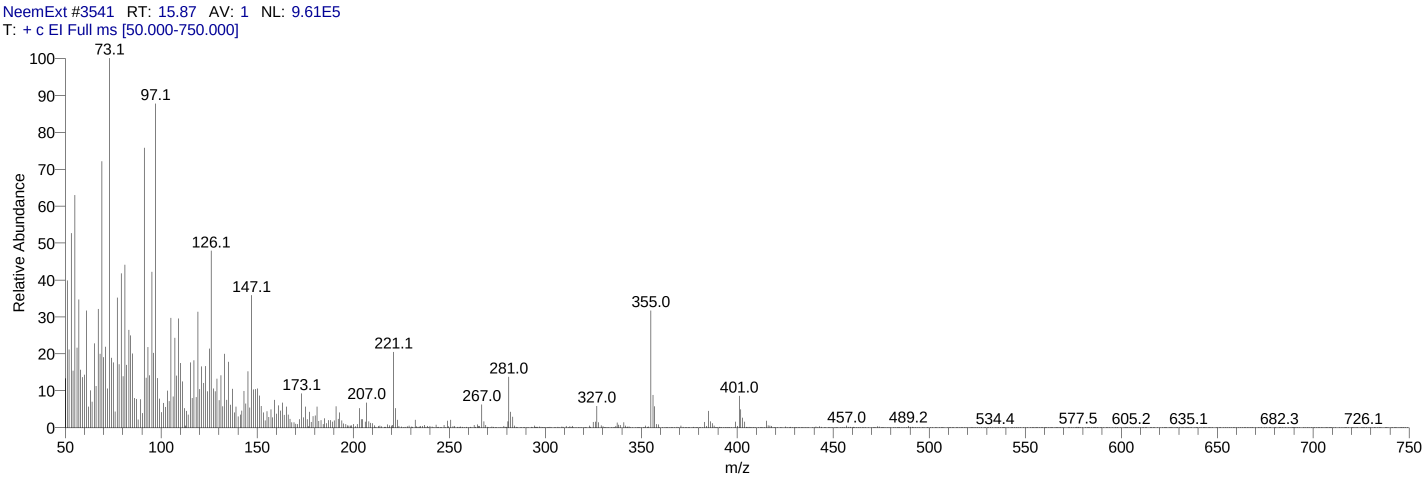

RT	Compound Name	Area %	MF	Molecular Formula	Molecular Weight	Cas #	Library
15.87	Sarreroside	6.48	710	C30H42O10	562	NA	mainlib
15.87	TERT-BUTYL-{2-[3-(2,2-DIMETHYL-6-METHYLENE-CYCLOHEXYL)-PROPYL]-[1,3]DITHIAN-2-YL}-DIMETHYL-SILANE	6.48	679	C22H42S2Si	398	NA	WileyRegistry8e
15.87	t-Butyl-(2-[3-(2,2-dimethyl-6-methylene-cyclohexyl)-propyl]-[1,3]dithian-2-yl)-dimethyl-silane	6.48	678	C22H42S2Si	398	95472-42-7	mainlib
15.87	4H-1-BENZOPYRAN-4-ONE, 2-(3,4-DIHYDROXYPHENYL)-6,8-DI-á-D-GLUCOPYRANOSYL-5,7-DIHYDROXY-	6.48	626	C27H30O16	610	29428-58-8	WileyRegistry8e
15.87	(S,S,S,S)-1,1'-Bicyclopentyl-2,2'-dicarboxaldehyde	6.48	772	C12H18O2	194	NA	mainlib

Compound Structure | Hit Spectrum

Sarreroside
Formula C30H42O10, MW 562, CAS# NA, Entry# 44550

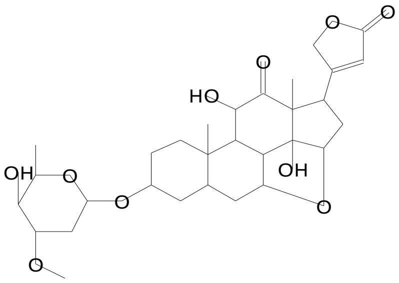

SI 680, RSI 710, mainlib, Entry# 44550, CAS# NA, Sarreroside

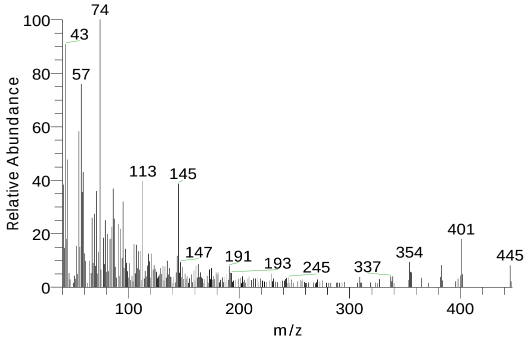

My GC-MS Report

Compound Structure | Hit Spectrum

Formula C22H42S2Si, MW 398, CAS# NA, Entry# 369252

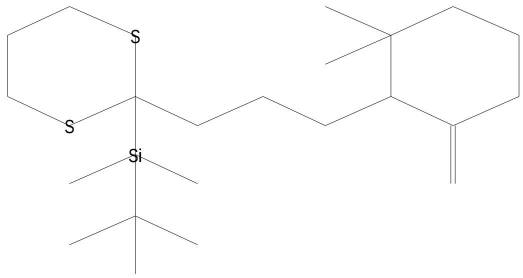

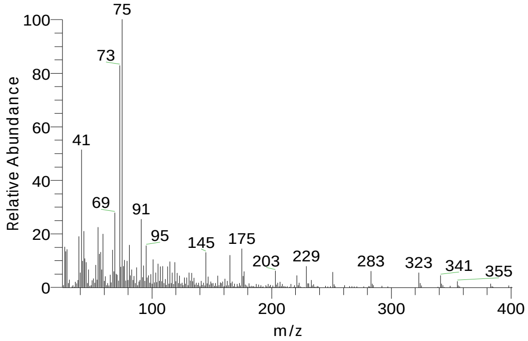

t-Butyl-(2-[3-(2,2-dimethyl-6-methylene-cyclohexyl)-propyl]-[1,3]dithian-2-yl)-dimethyl-silane
Formula C22H42S2Si, MW 398, CAS# 95472-42-7, Entry# 45252
tert-Butyl(2-[3-(2,2-dimethyl-6-methylenecyclohexyl)propyl]-1,3-dithian-2-yl)dimethylsilane #

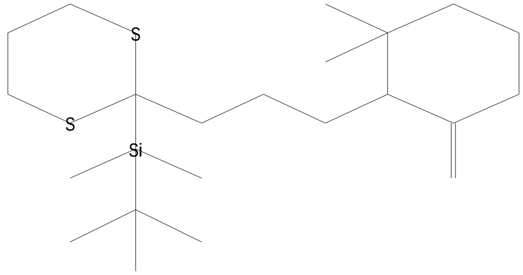

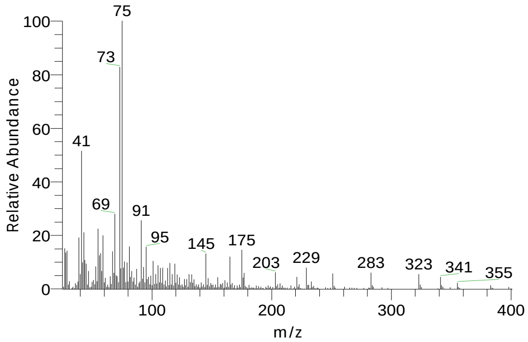

Formula C27H30O16, MW 610, CAS# 29428-58-8, Entry# 297453
6,8-DI-C-á-GLUCOSYLLUTEOLIN

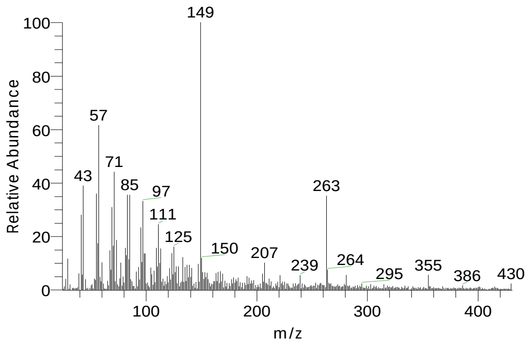

(S,S,S,S)-1,1'-Bicyclopentyl-2,2'-dicarboxaldehyde
Formula C12H18O2, MW 194, CAS# NA, Entry# 32501
$:28KERACRHKVZQPLQ-DDHJBXDOSA-N

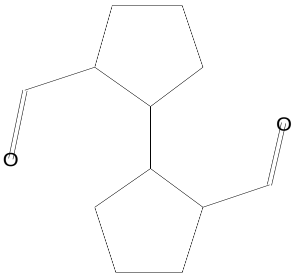

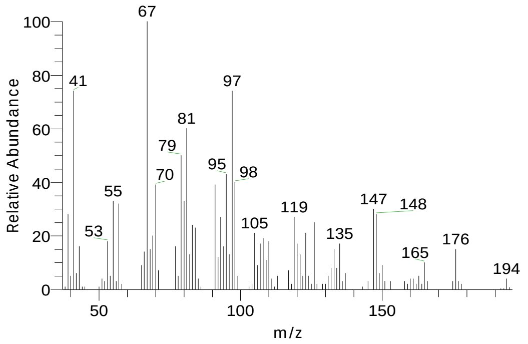

My GC-MS Report

NeemExt #3890 RT: 17.04 AV: 1 NL: 6.00E5
T: + c EI Full ms [50.000-750.000]

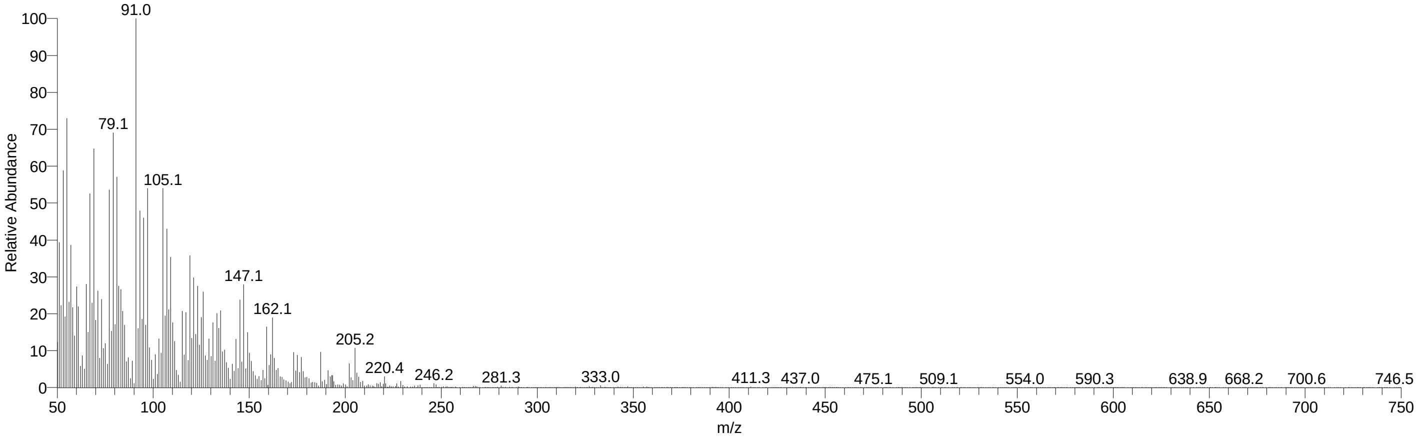

RT	Compound Name	Area %	MF	Molecular Formula	Molecular Weight	Cas #	Library
17.04	Caryophyllene oxide	1.69	830	C15H24O	220	1139-30-6	replib
17.04	Aromadendrene oxide-(2)	1.69	824	C15H24O	220	NA	mainlib
17.04	AROMADENDRENOXID-(2)	1.69	824	C15H24O	220	NA	WileyRegistry8e
17.04	2-[4-methyl-6-(2,6,6-trimethylcyclohex-1-enyl)hexa-1,3,5-trienyl]cyclohex-1-en-1-carboxaldehyde	1.69	765	C23H32O	324	NA	mainlib
17.04	ALLOAROMADENDRENOXID-(1)	1.69	815	C15H24O	220	85710-39-0	WileyRegistry8e

Compound Structure	Hit Spectrum

Caryophyllene oxide
Formula C15H24O, MW 220, CAS# 1139-30-6, Entry# 915

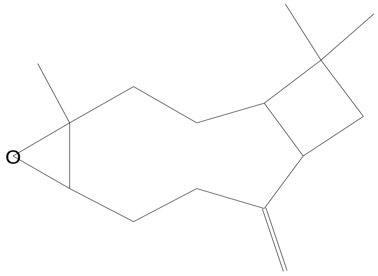

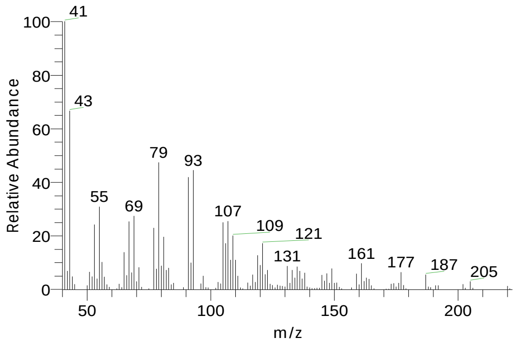

Aromadendrene oxide-(2)
Formula C15H24O, MW 220, CAS# NA, Entry# 2891
$:28XPGWKKLDFXNBPJ-UHFFFAOYSA-N

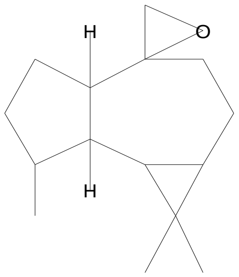

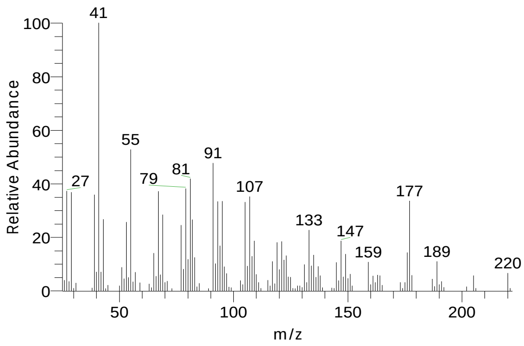

My GC-MS Report

Compound Structure | Hit Spectrum

AROMADENDRENOXID-(2)
Formula C15H24O, MW 220, CAS# NA, Entry# 387063

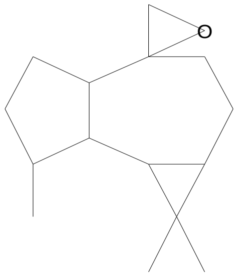

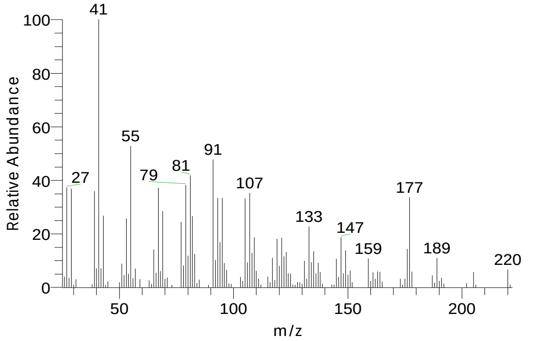

Formula C23H32O, MW 324, CAS# NA, Entry# 5913

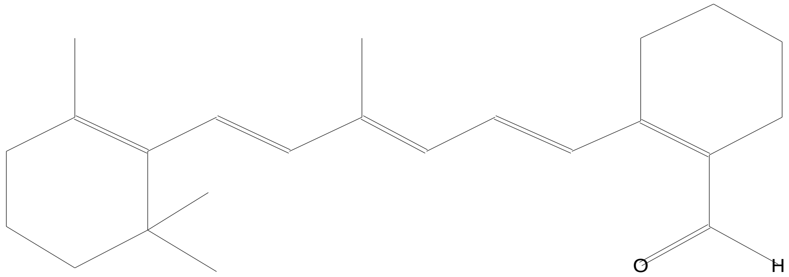

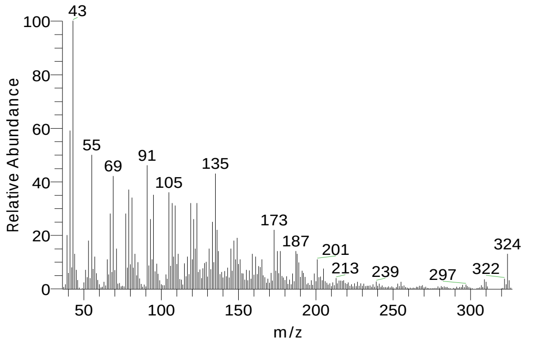

ALLOAROMADENDRENOXID-(1)
Formula C15H24O, MW 220, CAS# 85710-39-0, Entry# 107243
AROMADENDRENEPOXIDE-(I)

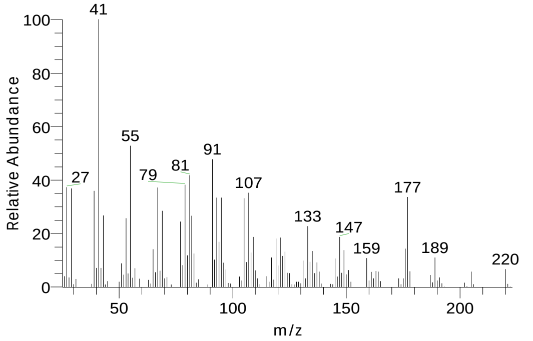

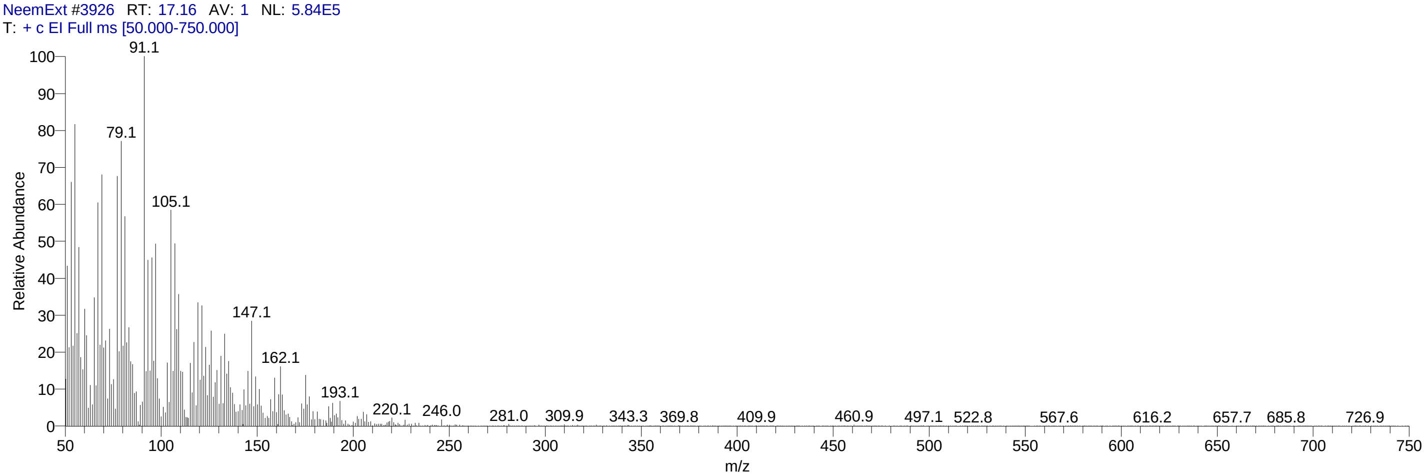

RT	Compound Name	Area %	MF	Molecular Formula	Molecular Weight	Cas #	Library
17.16	2-[4-methyl-6-(2,6,6-trimethylcyclohex-1-enyl)hexa-1,3,5-trienyl]cyclohex-1-en-1-carboxaldehyde	4.78	767	C23H32O	324	NA	mainlib
17.16	Caryophyllene oxide	4.78	821	C15H24O	220	1139-30-6	replib

My GC-MS Report

RT	Compound Name	Area %	MF	Molecular Formula	Molecular Weight	Cas #	Library
17.16	Methyl 7,10,13,16,19-docosapentaenoate	4.78	783	C23H36O2	344	NA	mainlib
17.16	Preg-4-en-3-one, 17à-hydroxy-17á-cyano-	4.78	779	C20H27NO2	313	NA	mainlib
17.16	Methyl 5,7-hexadecadiynoate	4.78	779	C17H26O2	262	NA	mainlib

Compound Structure	Hit Spectrum

Formula C23H32O, MW 324, CAS# NA, Entry# 5913

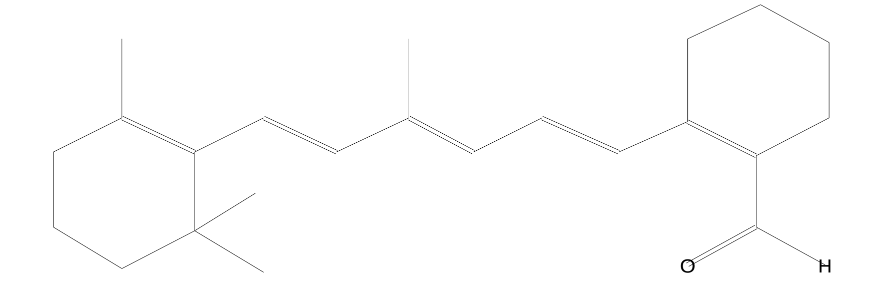

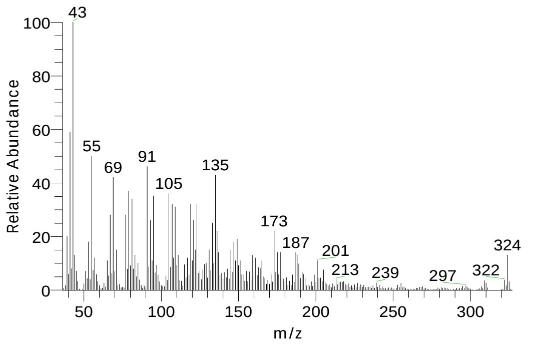

Caryophyllene oxide
Formula C15H24O, MW 220, CAS# 1139-30-6, Entry# 915

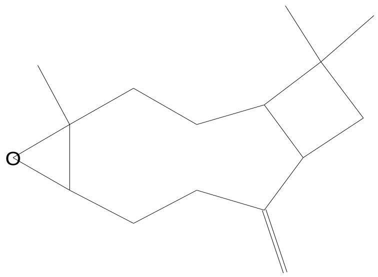

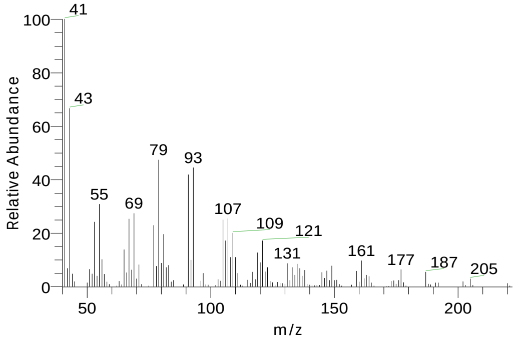

Methyl 7,10,13,16,19-docosapentaenoate
Formula C23H36O2, MW 344, CAS# NA, Entry# 48961
$:28PTFHIRHGARALFY-JEBPEJKESA-N

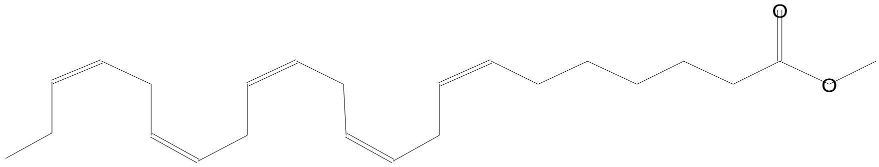

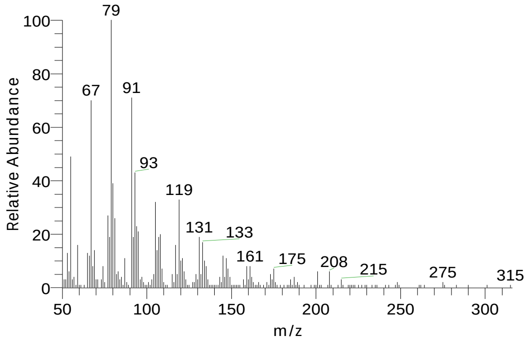

Preg-4-en-3-one, 17à-hydroxy-17á-cyano-
Formula C20H27NO2, MW 313, CAS# NA, Entry# 2652
17-Hydroxy-3-oxoandrost-4-ene-17-carbonitrile #

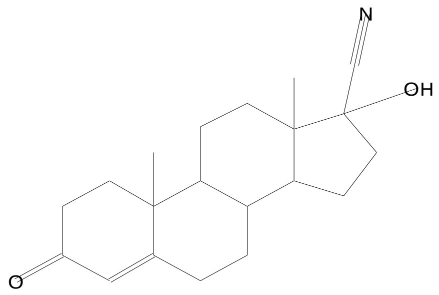

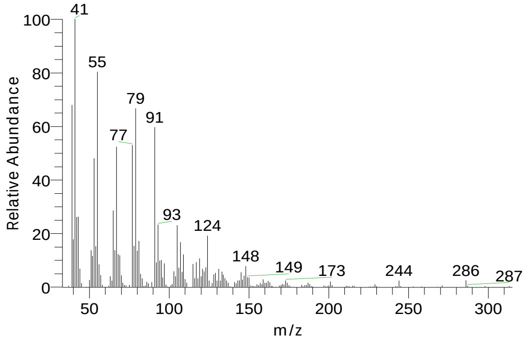

My GC-MS Report

Compound Structure | Hit Spectrum

Methyl 5,7-hexadecadiynoate
Formula C17H26O2, MW 262, CAS# NA, Entry# 60889
$:28FRDFHSWMSUAMBJ-UHFFFAOYSA-N

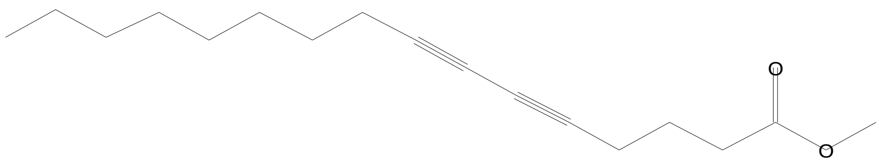

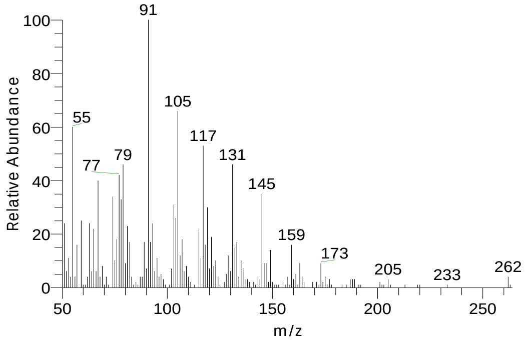

NeemExt #4380 RT: 18.69 AV: 1 NL: 1.17E6
T: + c EI Full ms [50.000-750.000]

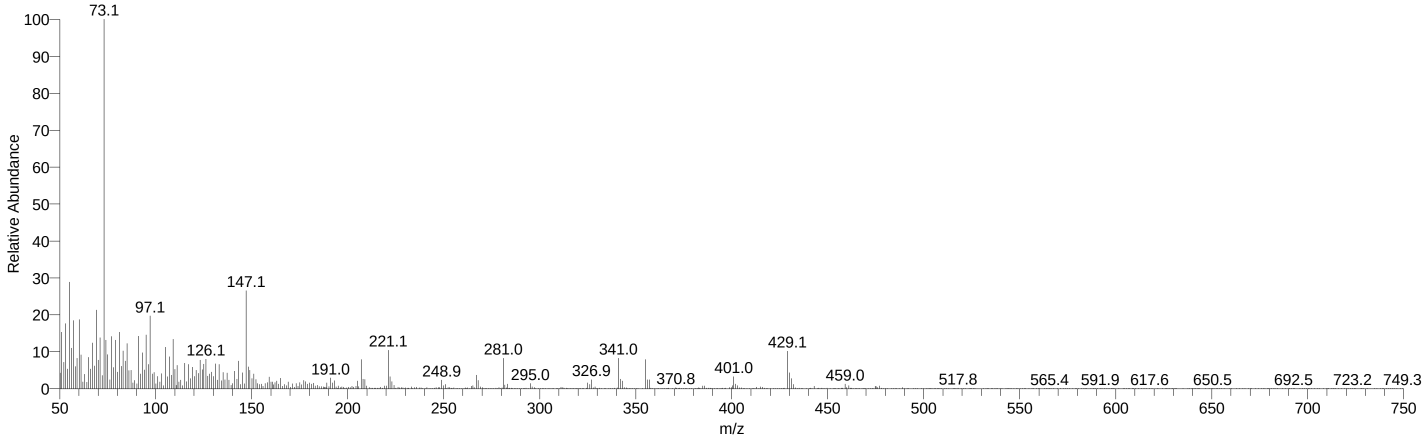

RT	Compound Name	Area %	MF	Molecular Formula	Molecular Weight	Cas #	Library
18.69	BISTRIMETHYLSILYL N-ACETYL EICOSASPHINGA-4,11-DIENINE	1.18	621	C28H57NO3Si2	511	NA	WileyRegistry8e
18.69	2-AMINOETHANETHIOL HYDROGEN SULFATE (ESTER)	1.18	775	C2H7NO3S2	157	2937-53-3	WileyRegistry8e
18.69	Melezitose	1.18	710	C18H32O16	504	597-12-6	mainlib
18.69	3-O-HEXOPYRANOSYLHEX-2-ULOFURANOSYL HEXOPYRANOSIDE #	1.18	710	C18H32O16	504	597-12-6	WileyRegistry8e
18.69	SILICONE OIL	1.18	614	N/A	0	NA	WileyRegistry8e

Compound Structure | Hit Spectrum

BISTRIMETHYLSILYL N-ACETYL EICOSASPHINGA-4,11-DIENINE
Formula C28H57NO3Si2, MW 511, CAS# NA, Entry# 287247

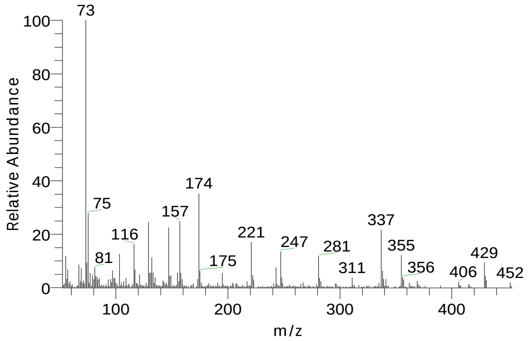

My GC-MS Report

Compound Structure | Hit Spectrum

2-AMINOETHANETHIOL HYDROGEN SULFATE (ESTER)
Formula C2H7NO3S2, MW 157, CAS# 2937-53-3, Entry# 41029
2-AMINOETHANETHIOLSULFURIC ACID

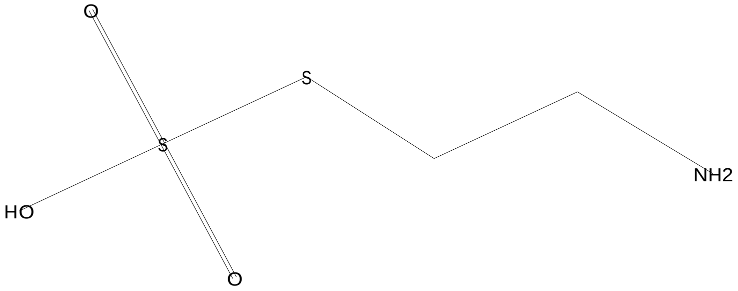

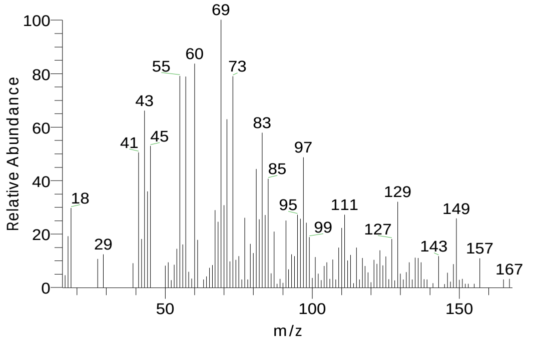

Melezitose
Formula C18H32O16, MW 504, CAS# 597-12-6, Entry# 41139
à-D-Glucopyranoside, O-à-D-glucopyranosyl-(1.fwdarw.3)-á-D-fructofuranosyl

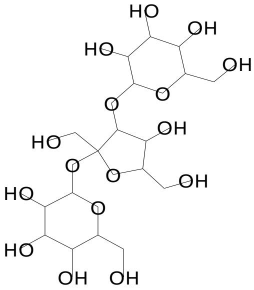

SI 583, RSI 710, mainlib, Entry# 41139, CAS# 597-12-6, Melezitose

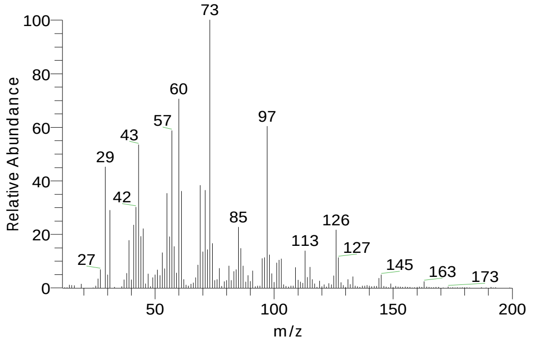

3-O-HEXOPYRANOSYLHEX-2-ULOFURANOSYL HEXOPYRANOSIDE #
Formula C18H32O16, MW 504, CAS# 597-12-6, Entry# 286042
à-D-GLUCOPYRANOSIDE, O-à-D-GLUCOPYRANOSYL-(1.->.3)-á-D-FRUCTOFURANOSYL

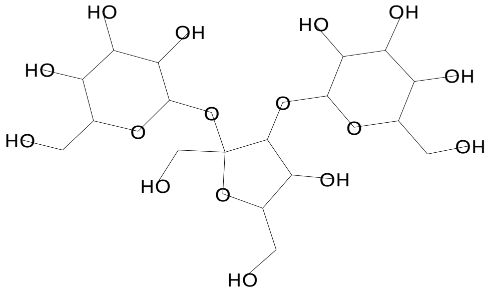

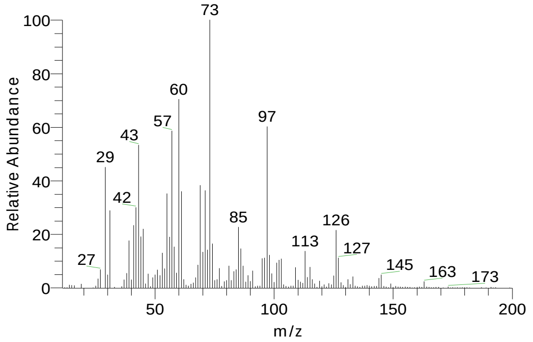

SILICONE OIL
Formula , MW 0, CAS# NA, Entry# 305490
SILIKONFETT SE30 (GREVELS)

SI 577, RSI 614, WileyRegistry8e, Entry# 305490, CAS# NA, SILICONE OIL

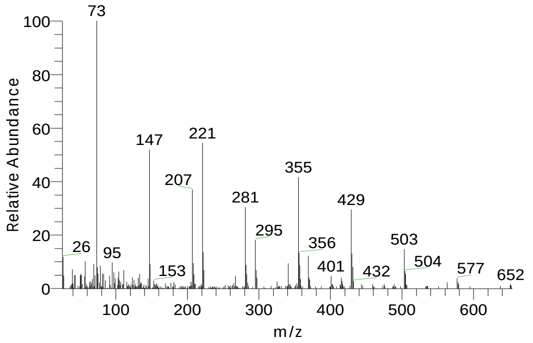

My GC-MS Report

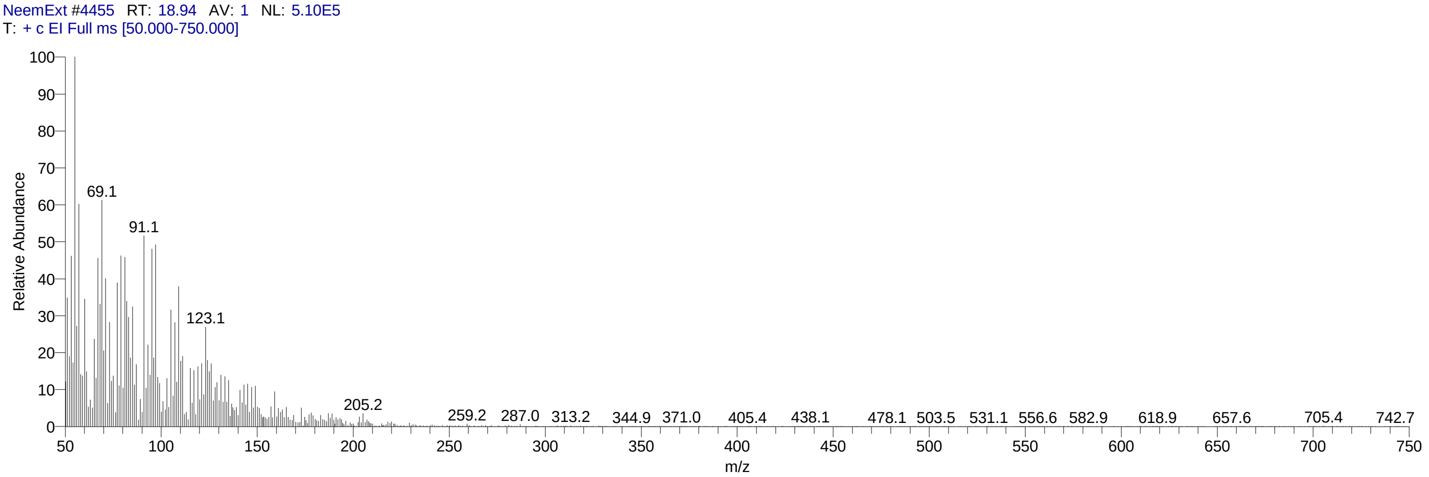

RT	Compound Name	Area %	MF	Molecular Formula	Molecular Weight	Cas #	Library
18.94	1-Heptatriacotanol	0.72	791	C37H76O	536	105794-58-9	mainlib
18.94	Retinal	0.72	858	C20H28O	284	116-31-4	mainlib
18.94	RETINAL	0.72	858	C20H28O	284	116-31-4	WileyRegistry8e
18.94	CHOLEST-5-EN-3-OL (3á)-	0.72	750	C27H46O	386	57-88-5	WileyRegistry8e
18.94	Oleic Acid	0.72	709	C18H34O2	282	112-80-1	replib

Compound Structure	Hit Spectrum

1-Heptatriacotanol
Formula C37H76O, MW 536, CAS# 105794-58-9, Entry# 7279
1-Heptatriacontanol #

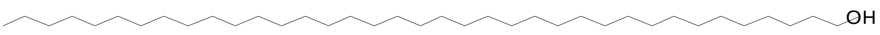

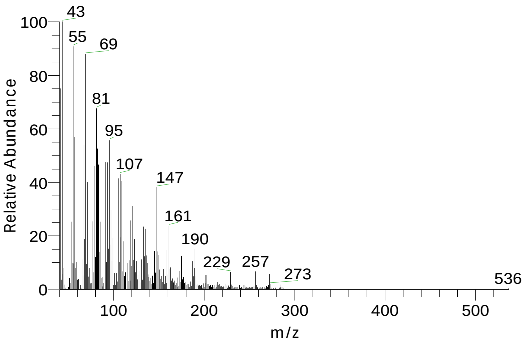

SI 757, RSI 858, mainlib, Entry# 60749, CAS# 116-31-4, Retinal

Retinal
Formula C20H28O, MW 284, CAS# 116-31-4, Entry# 60749
Vitamin A aldehyde

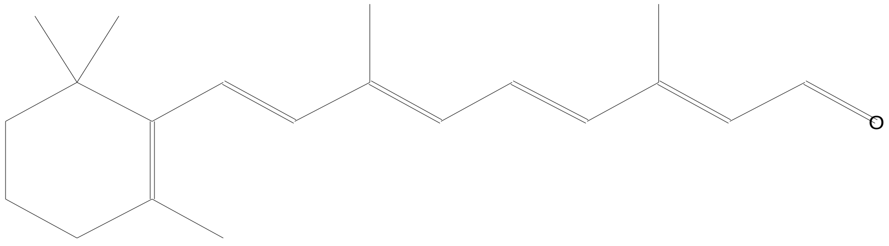

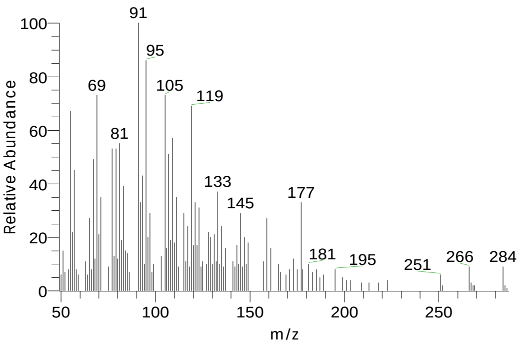

My GC-MS Report

Compound Structure | Hit Spectrum

RETINAL
Formula C20H28O, MW 284, CAS# 116-31-4, Entry# 175130
à-RETINENE

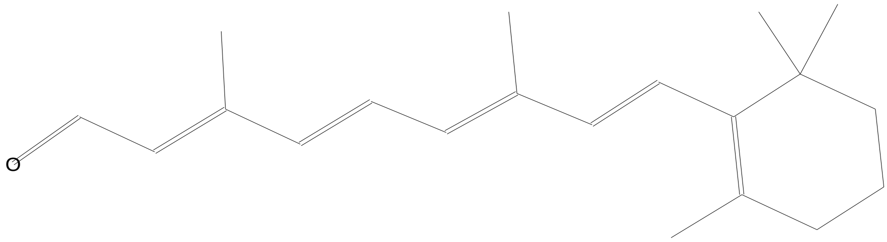

SI 757, RSI 858, WileyRegistry8e, Entry# 175130, CAS# 116-31-4, RETINAL

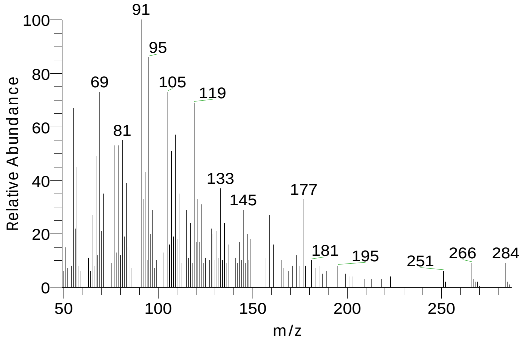

CHOLEST-5-EN-3-OL (3á)-
Formula C27H46O, MW 386, CAS# 57-88-5, Entry# 249636

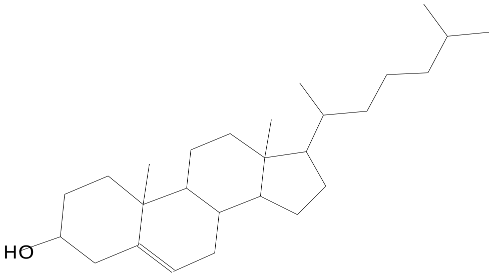

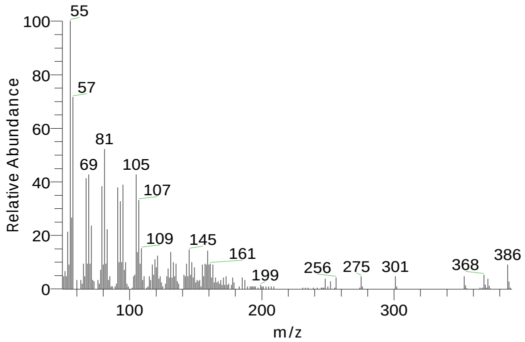

Oleic Acid
Formula C18H34O2, MW 282, CAS# 112-80-1, Entry# 4977
9-Octadecenoic acid (Z)-

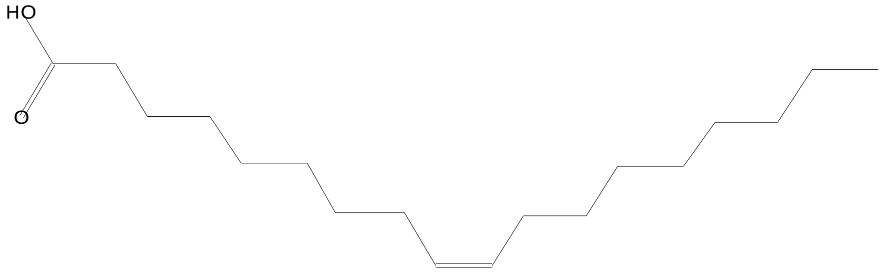

SI 707, RSI 709, replib, Entry# 4977, CAS# 112-80-1, Oleic Acid

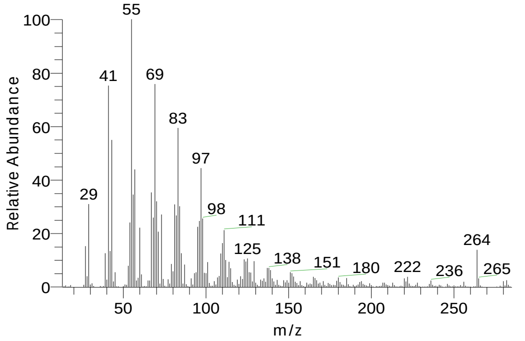

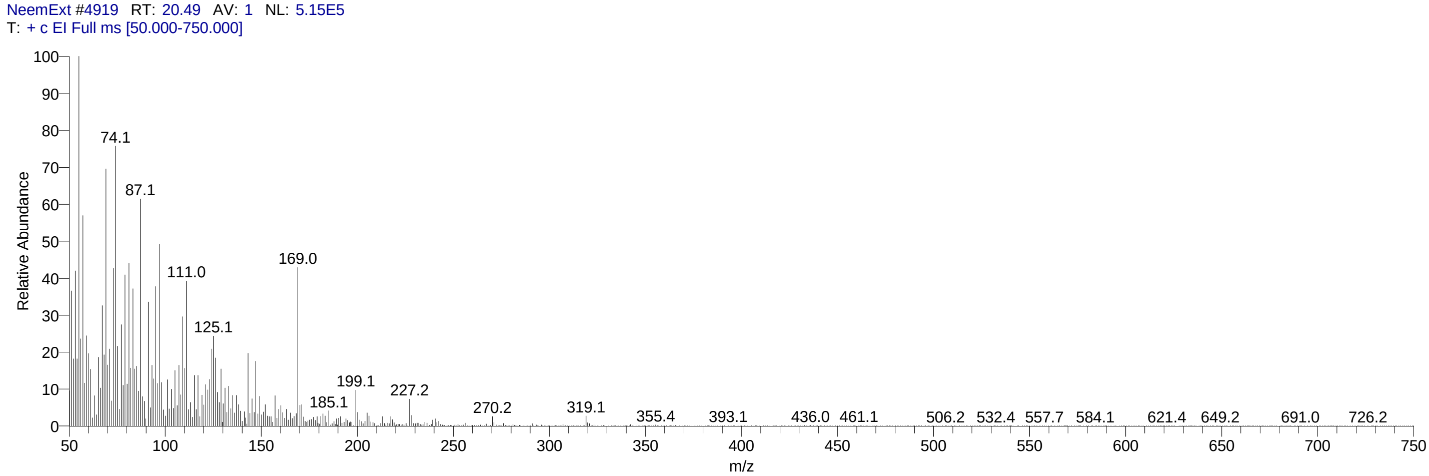

RT	Compound Name	Area %	MF	Molecular Formula	Molecular Weight	Cas #	Library
20.49	Cyclopropanedodecanoic acid, 2-octyl-, methyl ester	0.74	731	C24H46O2	366	10152-65-5	mainlib
20.49	Oxiraneundecanoic acid, 3-pentyl-, methyl ester, cis-	0.74	729	C19H36O3	312	38520-30-8	mainlib

My GC-MS Report

RT	Compound Name	Area %	MF	Molecular Formula	Molecular Weight	Cas #	Library
20.49	OXIRANEUNDECANOIC ACID, 3-PENTYL-, METHYL ESTER, CIS-	0.74	729	C19H36O3	312	38520-30-8	WileyRegistry8e
20.49	Oxiraneundecanoic acid, 3-pentyl-, methyl ester, trans-	0.74	722	C19H36O3	312	38520-31-9	mainlib
20.49	OXIRANEUNDECANOIC ACID, 3-PENTYL-, METHYL ESTER, TRANS-	0.74	722	C19H36O3	312	38520-31-9	WileyRegistry8e

Compound Structure | Hit Spectrum

Cyclopropanedodecanoic acid, 2-octyl-, methyl ester
Formula C24H46O2, MW 366, CAS# 10152-65-5, Entry# 2717
Methyl 12-(2-octylcyclopropyl)dodecanoate #

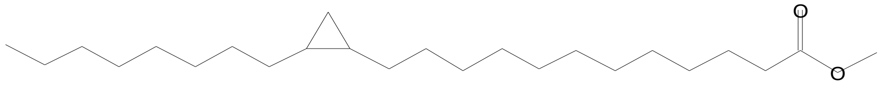

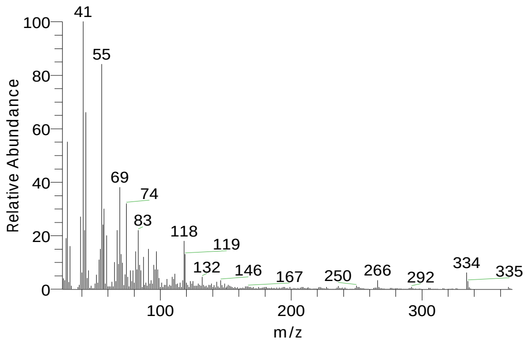

Oxiraneundecanoic acid, 3-pentyl-, methyl ester, cis-
Formula C19H36O3, MW 312, CAS# 38520-30-8, Entry# 19097
Methyl 11-(3-pentyl-2-oxiranyl)undecanoate, cis-

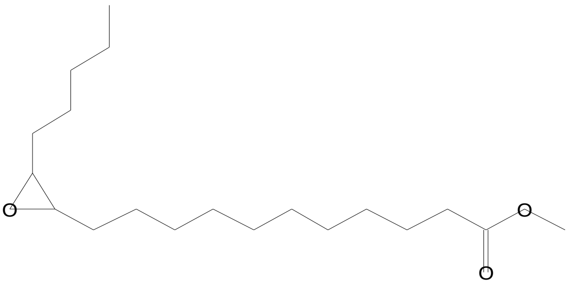

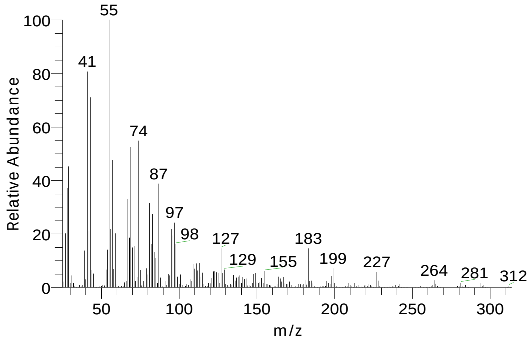

OXIRANEUNDECANOIC ACID, 3-PENTYL-, METHYL ESTER, CIS-
Formula C19H36O3, MW 312, CAS# 38520-30-8, Entry# 200210
METHYL 11-(3-PENTYL-2-OXIRANYL)UNDECANOATE #

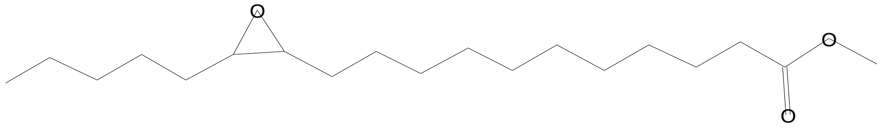

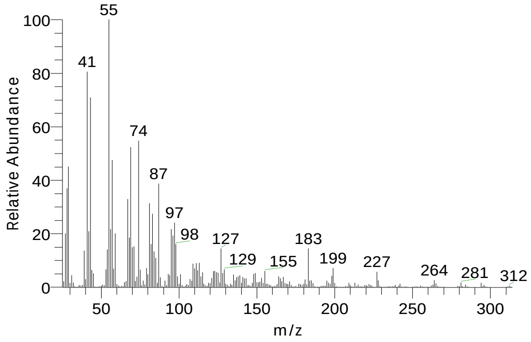

Oxiraneundecanoic acid, 3-pentyl-, methyl ester, trans-
Formula C19H36O3, MW 312, CAS# 38520-31-9, Entry# 18982
Methyl 11-(3-pentyl-2-oxiranyl)undecanoate, trans-

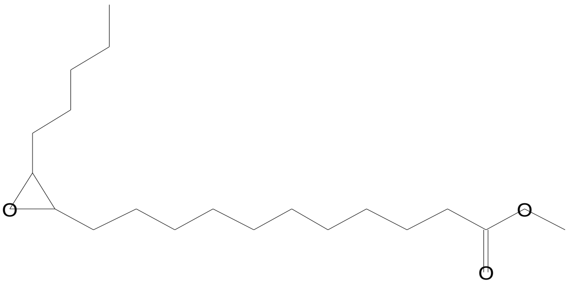

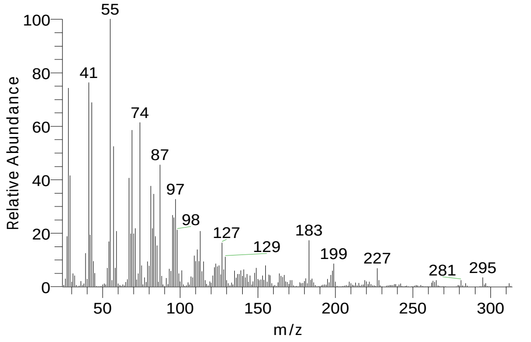

My GC-MS Report

Compound Structure | Hit Spectrum

OXIRANEUNDECANOIC ACID, 3-PENTYL-, METHYL ESTER, TRANS-
Formula C19H36O3, MW 312, CAS# 38520-31-9, Entry# 200212
METHYL 11-(3-PENTYL-2-OXIRANYL)UNDECANOATE #

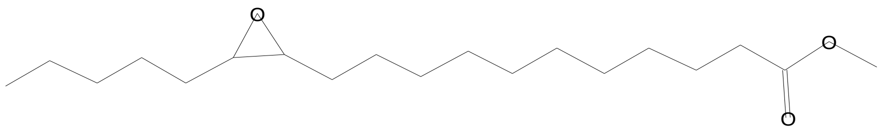

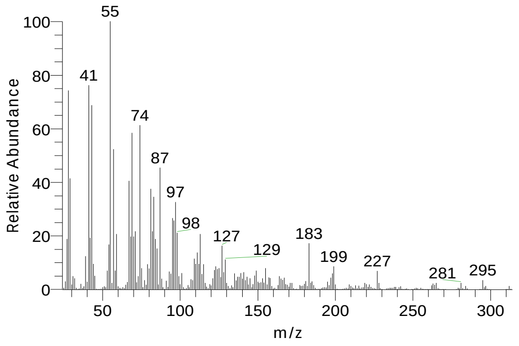

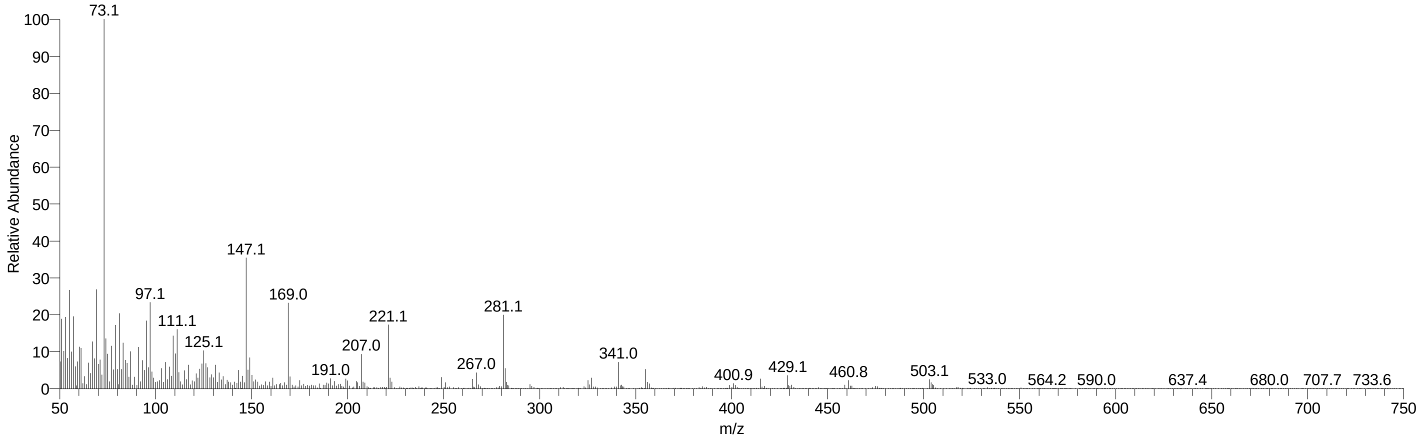

RT	Compound Name	Area %	MF	Molecular Formula	Molecular Weight	Cas #	Library
21.18	9,12,15-OCTADECATRIENOIC ACID, 2-[(TRIMETHYLSILYL)OXY]-1-[[(TRIMETHYLSILYL)OXY]METHYL]ETHYL ESTER, (Z,Z,Z)-	0.94	779	C27H52O4Si2	496	55521-23-8	WileyRegistry8e
21.18	9,12-OCTADECADIENOIC ACID (Z,Z)-, 2,3-BIS[(TRIMETHYLSILYL)OXY]PROPYL ESTER	0.94	706	C27H54O4Si2	498	54284-45-6	WileyRegistry8e
21.18	9-OCTADECENOIC ACID (Z)-, 2-[(TRIMETHYLSILYL)OXY]-1-[[(TRIMETHYLSILYL)OXY]METHYL]ETHYL ESTER	0.94	684	C27H56O4Si2	500	54284-48-9	WileyRegistry8e
21.18	GLYCINE, N-[(3à,5á)-24-OXO-3-[(TRIMETHYLSILYL)OXY]CHOLAN-24-YL]-, METHYL ESTER	0.94	672	C30H53NO4Si	519	57326-15-5	WileyRegistry8e
21.18	Glycine, N-[(3à,5á)-24-oxo-3-[(trimethylsilyl)oxy]cholan-24-yl]-, methyl ester	0.94	668	C30H53NO4Si	519	57326-15-5	mainlib

My GC-MS Report

Compound Structure | Hit Spectrum

Formula C27H52O4Si2, MW 496, CAS# 55521-23-8, Entry# 284835

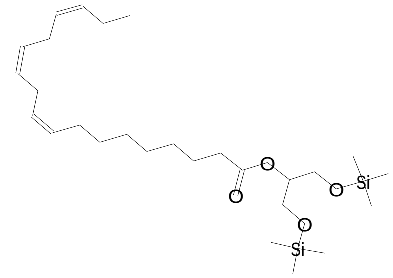

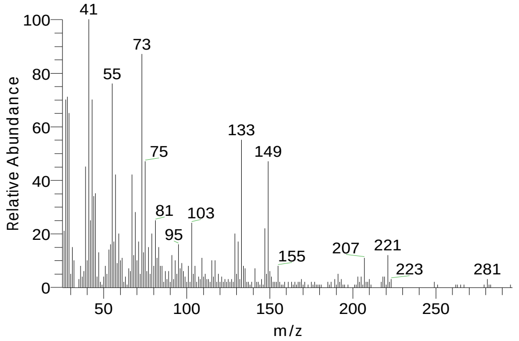

9,12-OCTADECADIENOIC ACID (Z,Z)-, 2,3-BIS[(TRIMETHYLSILYL)OXY]PROPYL ESTER
Formula C27H54O4Si2, MW 498, CAS# 54284-45-6, Entry# 285148
2,3-BIS[(TRIMETHYLSILYL)OXY]PROPYL (9Z,12Z)-9,12-OCTADECADIENOATE #

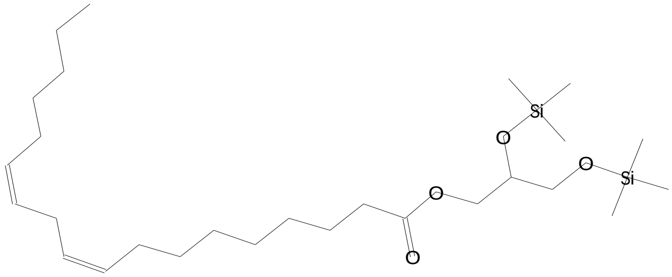

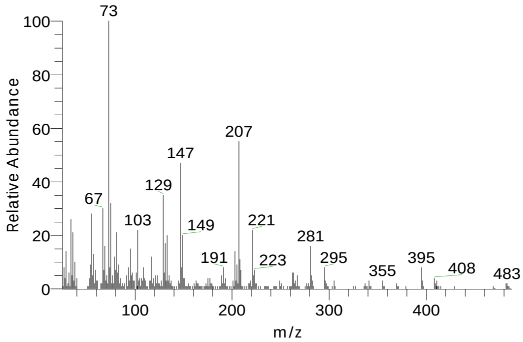

Formula C27H56O4Si2, MW 500, CAS# 54284-48-9, Entry# 285456
2-MONOOLEOYLGLYCEROL TRIMETHYLSILYL ETHER

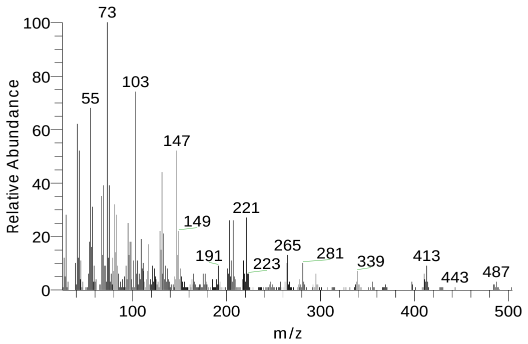

GLYCINE, N-[(3à,5á)-24-OXO-3-[(TRIMETHYLSILYL)OXY]CHOLAN-24-YL]-,METHYL ESTER
Formula C30H53NO4Si, MW 519, CAS# 57326-15-5, Entry# 288429
METHYL ((24-OXO-3-[(TRIMETHYLSILYL)OXY]CHOLAN-24-YL)AMINO)ACETATE #

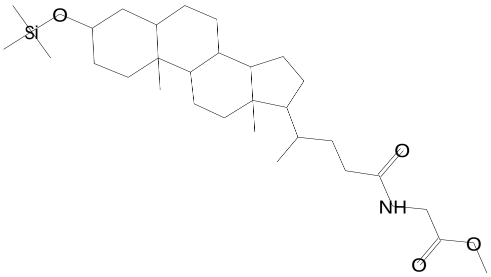

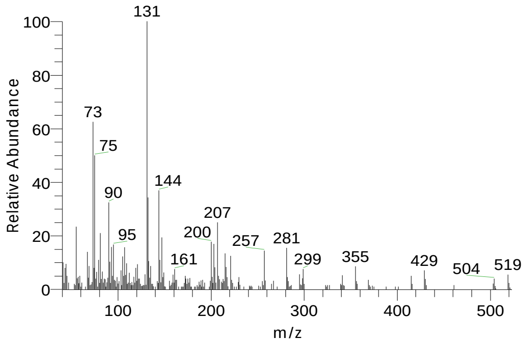

My GC-MS Report

Compound Structure | Hit Spectrum

Glycine, N-[(3à,5á)-24-oxo-3-[(trimethylsilyl)oxy]cholan-24-yl]-, methyl ester
Formula C30H53NO4Si, MW 519, CAS# 57326-15-5, Entry# 115521
Methyl ((24-oxo-3-[(trimethylsilyl)oxy]cholan-24-yl)amino)acetate #

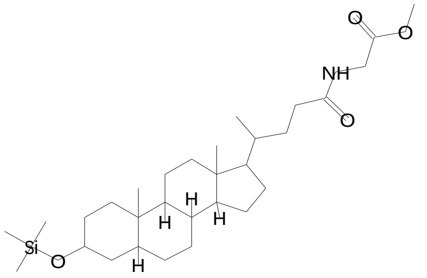

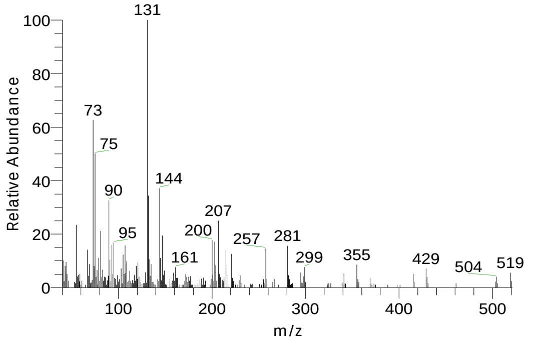

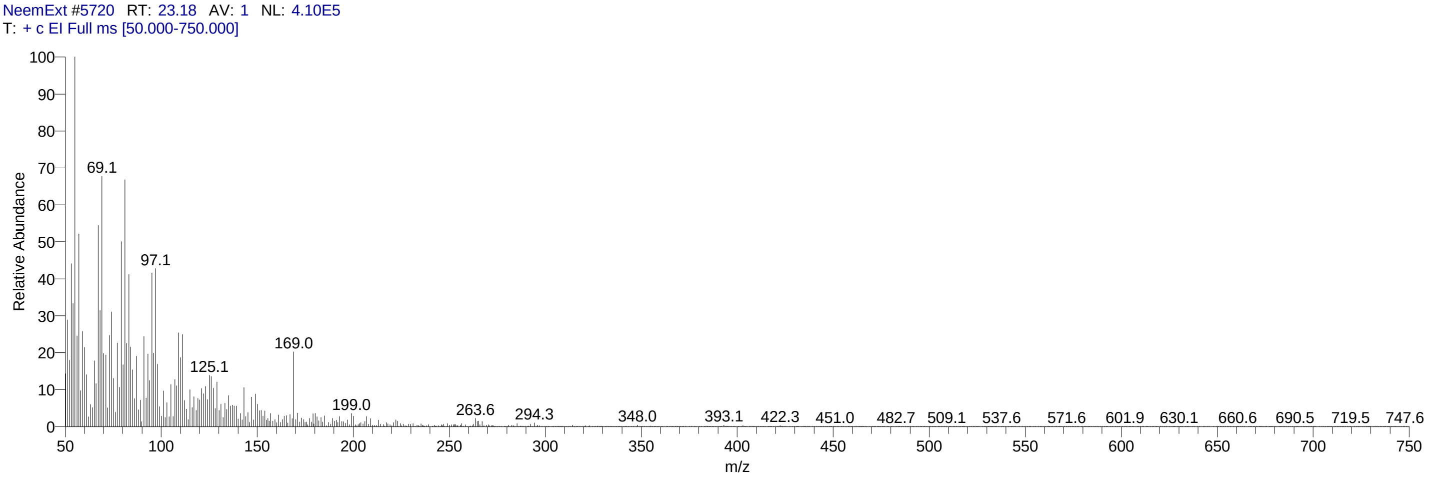

RT	Compound Name	Area %	MF	Molecular Formula	Molecular Weight	Cas #	Library
23.18	9-OCTADECENOIC ACID (Z)-	0.78	778	C18H34O2	282	112-80-1	WileyRegistry8e
23.18	9,12-Octadecadienoyl chloride, (Z,Z)-	0.78	795	C18H31ClO	298	7459-33-8	replib
23.18	(9E,12E)-9,12-OCTADECADIENOYL CHLORIDE #	0.78	795	C18H31ClO	298	7459-33-8	WileyRegistry8e
23.18	Z-(13,14-Epoxy)tetradec-11-en-1-ol acetate	0.78	763	C16H28O3	268	NA	mainlib
23.18	17-Octadecynoic acid	0.78	757	C18H32O2	280	34450-18-5	mainlib

Compound Structure | Hit Spectrum

9-OCTADECENOIC ACID (Z)-
Formula C18H34O2, MW 282, CAS# 112-80-1, Entry# 172910
OCTADEC-9-ENOIC ACID

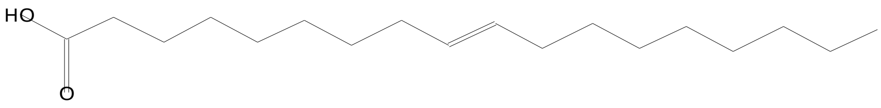

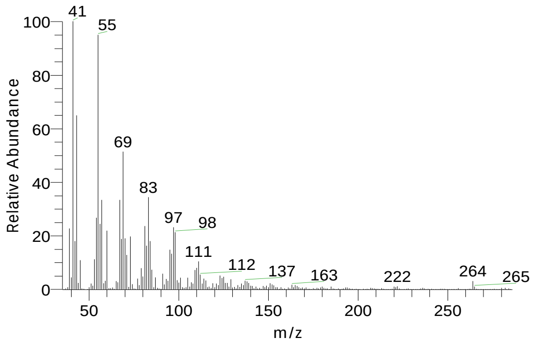

My GC-MS Report

Compound Structure | Hit Spectrum

9,12-Octadecadienoyl chloride, (Z,Z)-
Formula C18H31ClO, MW 298, CAS# 7459-33-8, Entry# 4940
Linoleoyl chloride

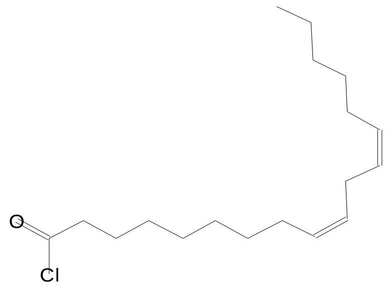

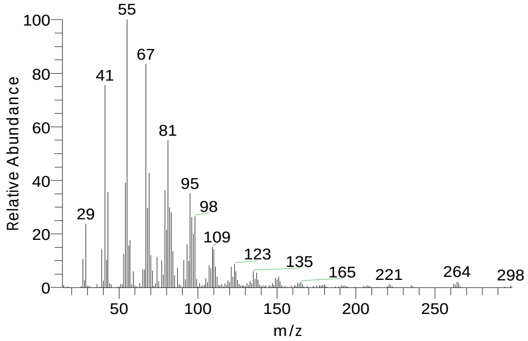

(9E,12E)-9,12-OCTADECADIENOYL CHLORIDE #
Formula C18H31ClO, MW 298, CAS# 7459-33-8, Entry# 187801
(9E,12E)-9,12-OCTADECADIENOYL CHLORIDE

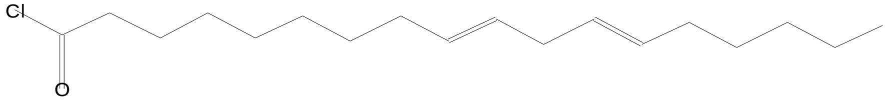

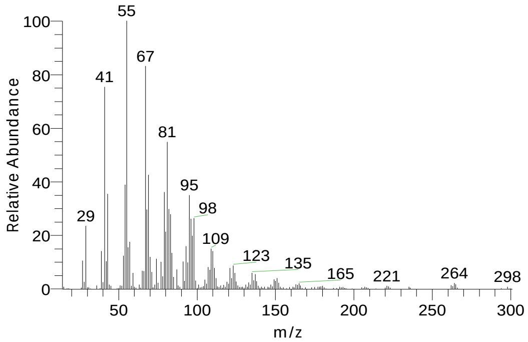

Z-(13,14-Epoxy)tetradec-11-en-1-ol acetate
Formula C16H28O3, MW 268, CAS# NA, Entry# 10459
(11Z)-12-(2-Oxiranyl)-11-dodecenyl acetate #

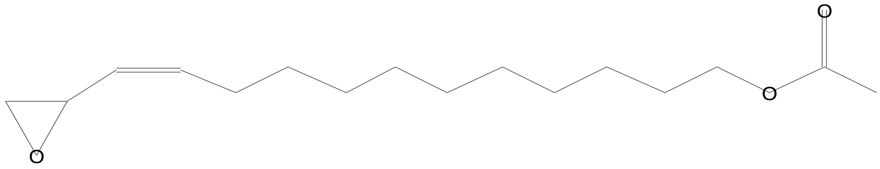

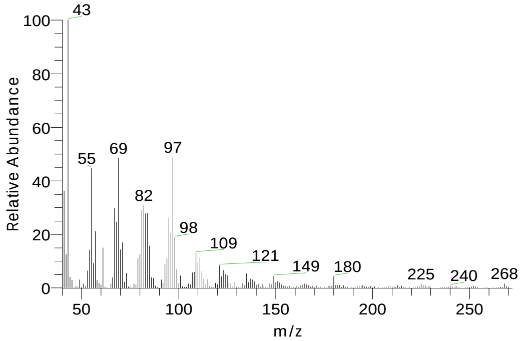

17-Octadecynoic acid
Formula C18H32O2, MW 280, CAS# 34450-18-5, Entry# 20510
$:28DZIILFGADWDKMF-UHFFFAOYSA-N

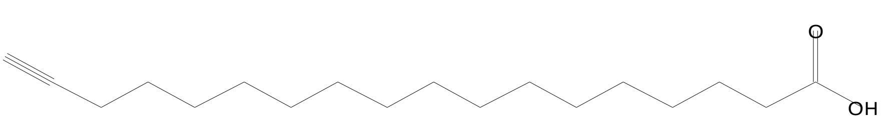

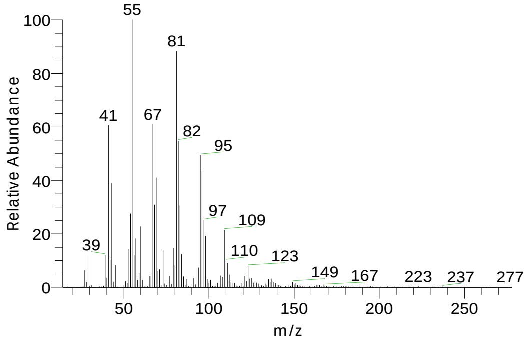

My GC-MS Report

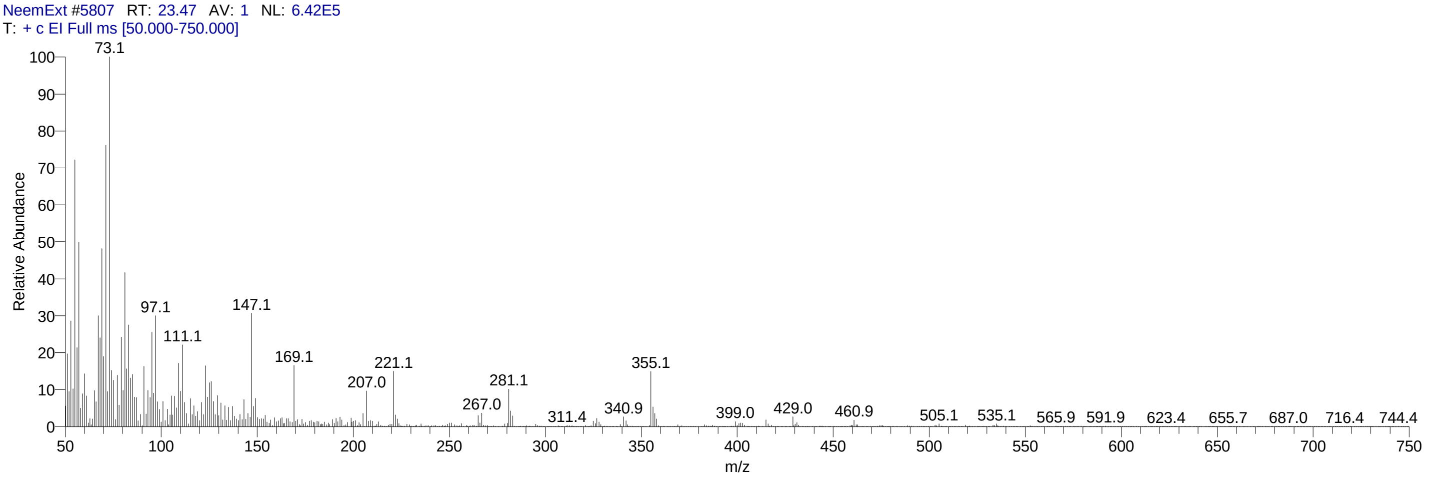

RT	Compound Name	Area %	MF	Molecular Formula	Molecular Weight	Cas #	Library
23.47	9,12,15-OCTADECATRIENOIC ACID, 2-[(TRIMETHYLSILYL)OXY]-1-[[(TRIMETHYLSILYL)OXY]METHYL]ETHYL ESTER, (Z,Z,Z)-	1.92	788	C27H52O4Si2	496	55521-23-8	WileyRegistry8e
23.47	4H-1-BENZOPYRAN-4-ONE, 2-(3,4-DIMETHOXYPHENYL)-3,5-DIHYDROXY-7-METHOXY-	1.92	738	C18H16O7	344	6068-80-0	WileyRegistry8e
23.47	Ethyl iso-allocholate	1.92	710	C26H44O5	436	NA	mainlib
23.47	ETHYL ISO-ALLOCHOLATE	1.92	710	C26H44O5	436	NA	WileyRegistry8e
23.47	01297107001 TETRANEURIN - A - DIOL	1.92	737	C15H20O5	280	NA	WileyRegistry8e

Compound Structure	Hit Spectrum

Formula C27H52O4Si2, MW 496, CAS# 55521-23-8, Entry# 284835

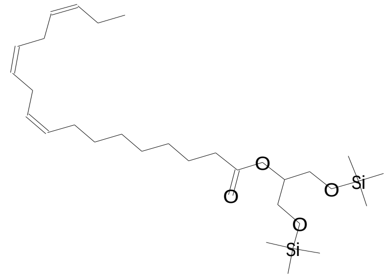

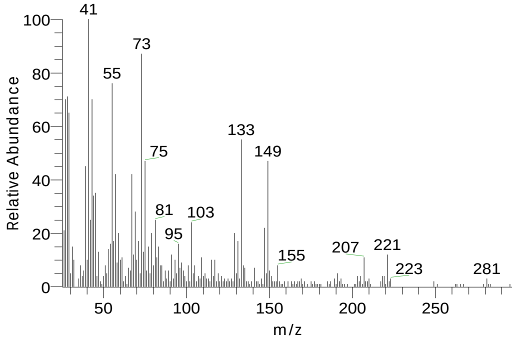

4H-1-BENZOPYRAN-4-ONE, 2-(3,4-DIMETHOXYPHENYL)-3,5-DIHYDROXY-7-METHOXY-
Formula C18H16O7, MW 344, CAS# 6068-80-0, Entry# 224392
3',4',7-TRIMETHYLQUERCETIN

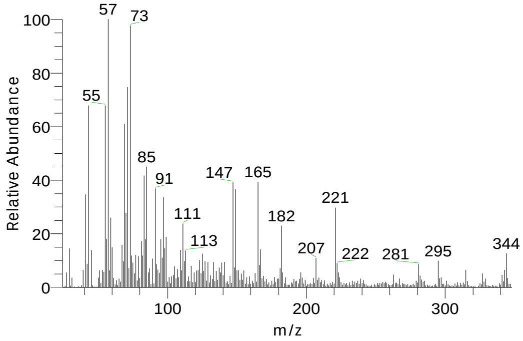

My GC-MS Report

Compound Structure

Hit Spectrum

Ethyl iso-allocholate
Formula C26H44O5, MW 436, CAS# NA, Entry# 7020
Ethyl 3,7,12-trihydroxycholan-24-oate #

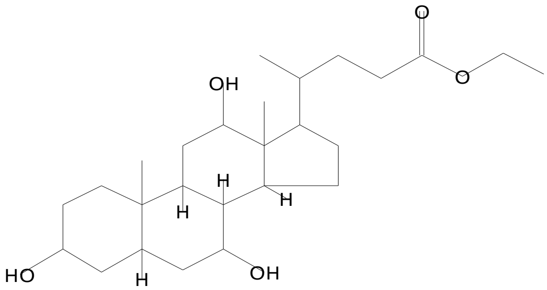

SI 693, RSI 710, mainlib, Entry# 7020, CAS# NA, Ethyl iso-allocholate

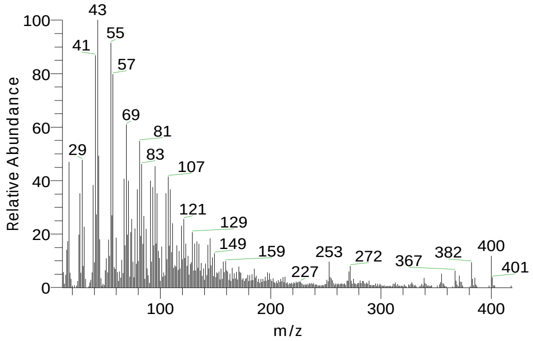

ETHYL ISO-ALLOCHOLATE
Formula C26H44O5, MW 436, CAS# NA, Entry# 270212

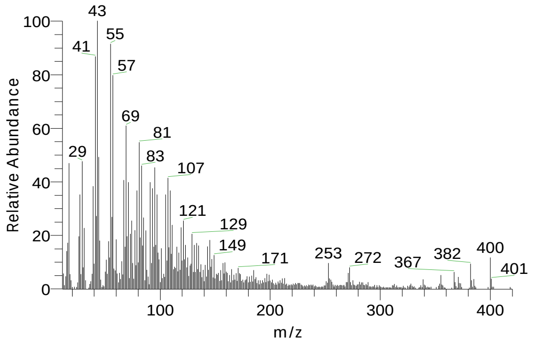

01297107001 TETRANEURIN - A - DIOL
Formula C15H20O5, MW 280, CAS# NA, Entry# 170378

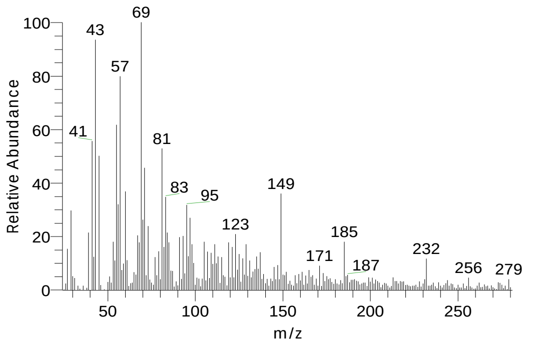

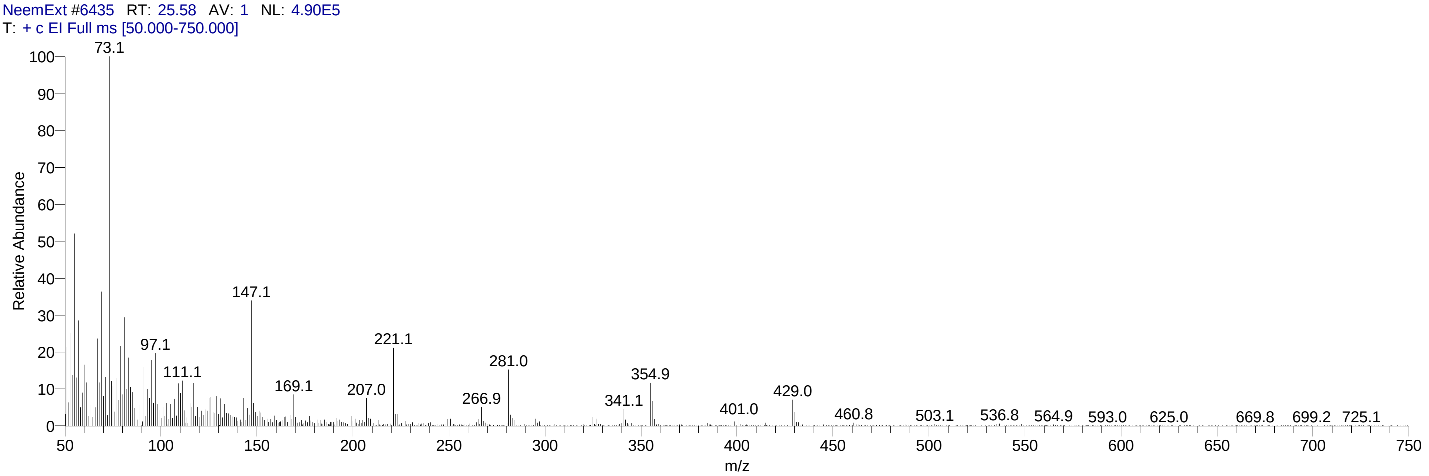

RT	Compound Name	Area %	MF	Molecular Formula	Molecular Weight	Cas #	Library
25.58	9,12,15-OCTADECATRIENOIC ACID, 2-[(TRIMETHYLSILYL)OXY]-1-[[(TRIMETHYLSILYL)OXY]METHYL]ETHYL ESTER, (Z,Z,Z)-	0.51	795	C27H52O4Si2	496	55521-23-8	WileyRegistry8e

My GC-MS Report

RT	Compound Name	Area %	MF	Molecular Formula	Molecular Weight	Cas #	Library
25.58	4H-1-BENZOPYRAN-4-ONE, 2-(3,4-DIMETHOXYPHENYL)-3,5-DIHYDROXY-7-METHOXY-	0.51	736	C18H16O7	344	6068-80-0	WileyRegistry8e
25.58	Ethyl iso-allocholate	0.51	703	C26H44O5	436	NA	mainlib
25.58	ETHYL ISO-ALLOCHOLATE	0.51	703	C26H44O5	436	NA	WileyRegistry8e
25.58	9-OCTADECENOIC ACID (Z)-, 2-[(TRIMETHYLSILYL)OXY]-1-[[(TRIMETHYLSILYL)OXY]METHYL]ETHYL ESTER	0.51	694	C27H56O4Si2	500	54284-48-9	WileyRegistry8e

Compound Structure | Hit Spectrum

Formula C27H52O4Si2, MW 496, CAS# 55521-23-8, Entry# 284835

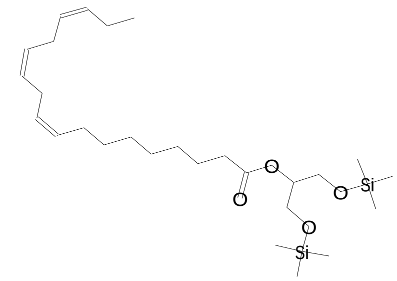

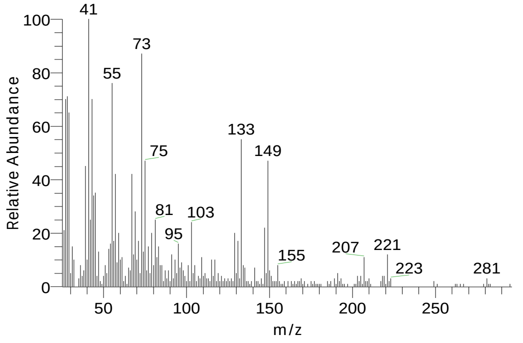

4H-1-BENZOPYRAN-4-ONE, 2-(3,4-DIMETHOXYPHENYL)-3,5-DIHYDROXY-7-METHOXY-
Formula C18H16O7, MW 344, CAS# 6068-80-0, Entry# 224392
3',4',7-TRIMETHYLQUERCETIN

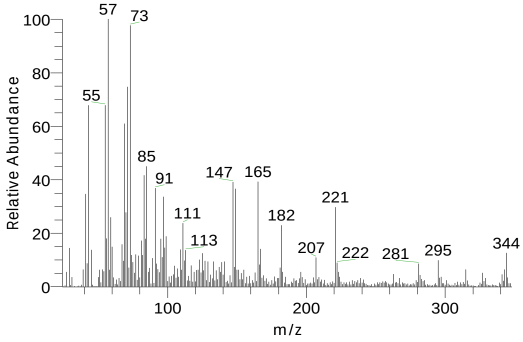

Ethyl iso-allocholate
Formula C26H44O5, MW 436, CAS# NA, Entry# 7020
Ethyl 3,7,12-trihydroxycholan-24-oate #

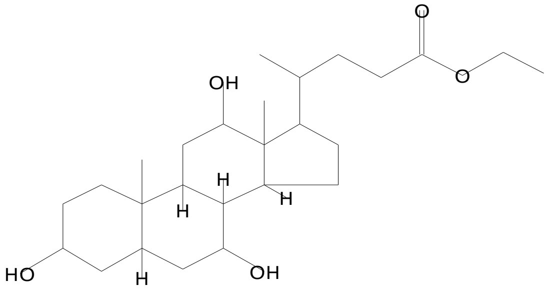

SI 680, RSI 703, mainlib, Entry# 7020, CAS# NA, Ethyl iso-allocholate

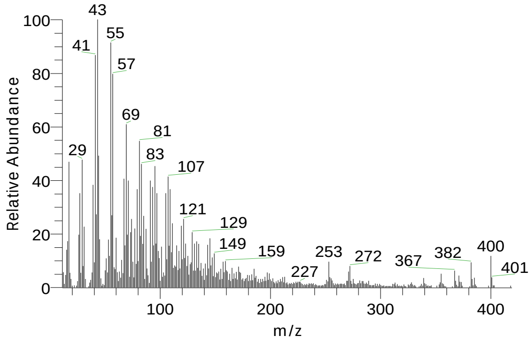

My GC-MS Report

Compound Structure | Hit Spectrum

ETHYL ISO-ALLOCHOLATE
Formula C26H44O5, MW 436, CAS# NA, Entry# 270212

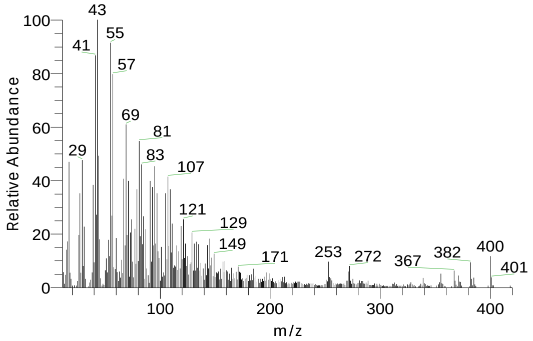

Formula C27H56O4Si2, MW 500, CAS# 54284-48-9, Entry# 285456
2-MONOOLEOYLGLYCEROL TRIMETHYLSILYL ETHER

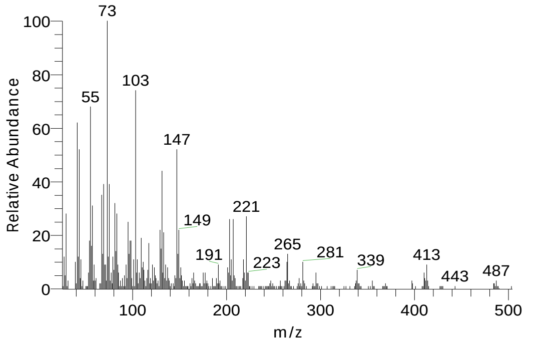

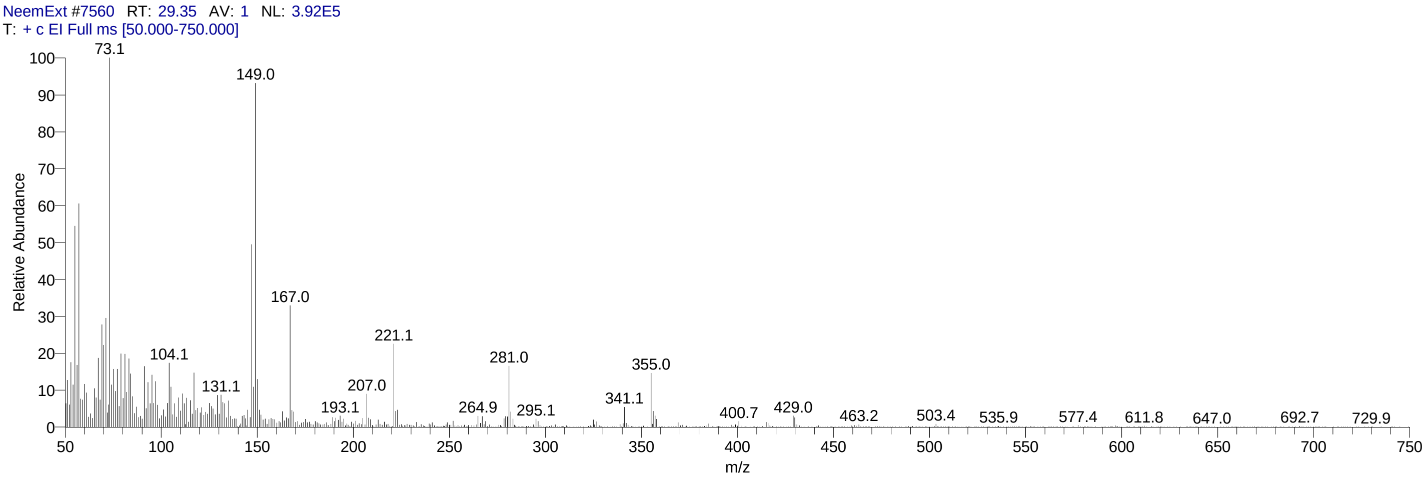

RT	Compound Name	Area %	MF	Molecular Formula	Molecular Weight	Cas #	Library
29.35	9,12,15-OCTADECATRIENOIC ACID, 2-[(TRIMETHYLSILYL)OXY]-1-[[(TRIMETHYLSILYL)OXY]METHYL]ETHYL ESTER, (Z,Z,Z)-	1.01	810	C27H52O4Si2	496	55521-23-8	WileyRegistry8e
29.35	4H-1-BENZOPYRAN-4-ONE, 2-(3,4-DIMETHOXYPHENYL)-3,5-DIHYDROXY-7-METHOXY-	1.01	727	C18H16O7	344	6068-80-0	WileyRegistry8e
29.35	9-OCTADECENOIC ACID (Z)-, 2-[(TRIMETHYLSILYL)OXY]-1-[[(TRIMETHYLSILYL)OXY]METHYL]ETHYL ESTER	1.01	698	C27H56O4Si2	500	54284-48-9	WileyRegistry8e
29.35	9,12-OCTADECADIENOIC ACID (Z,Z)-, 2,3-BIS[(TRIMETHYLSILYL)OXY]PROPYL ESTER	1.01	703	C27H54O4Si2	498	54284-45-6	WileyRegistry8e

My GC-MS Report

RT	Compound Name	Area %	MF	Molecular Formula	Molecular Weight	Cas #	Library
29.35	4H-1-BENZOPYRAN-4-ONE, 2-(3,4-DIHYDROXYPHENYL)-6,8-DI-á-D-GLUCOPYRANOSYL-5,7-DIHYDROXY-	1.01	674	C27H30O16	610	29428-58-8	WileyRegistry8e

Compound Structure | Hit Spectrum

Formula C27H52O4Si2, MW 496, CAS# 55521-23-8, Entry# 284835

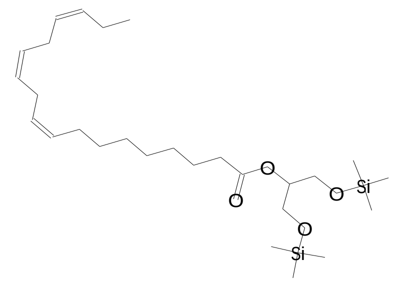

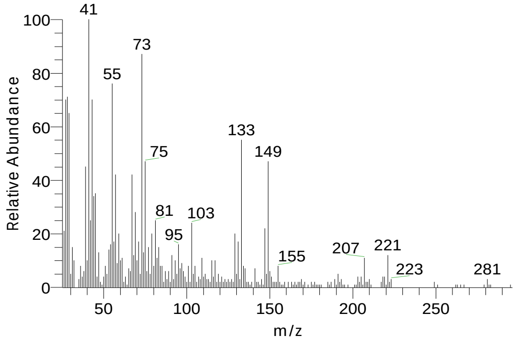

4H-1-BENZOPYRAN-4-ONE, 2-(3,4-DIMETHOXYPHENYL)-3,5-DIHYDROXY-7-METHOXY-
Formula C18H16O7, MW 344, CAS# 6068-80-0, Entry# 224392
3',4',7-TRIMETHYLQUERCETIN

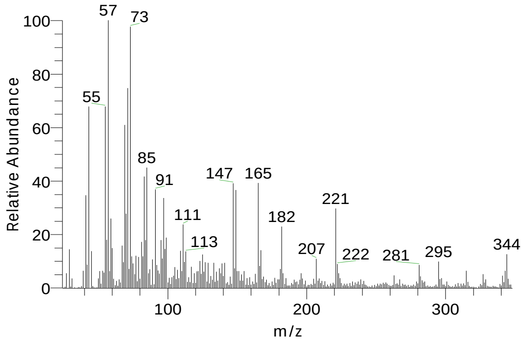

Formula C27H56O4Si2, MW 500, CAS# 54284-48-9, Entry# 285456
2-MONOOLEOYLGLYCEROL TRIMETHYLSILYL ETHER

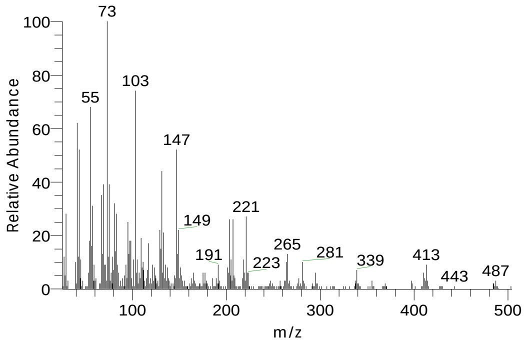

9,12-OCTADECADIENOIC ACID (Z,Z)-, 2,3-BIS[(TRIMETHYLSILYL)OXY]PROPYL ESTER
Formula C27H54O4Si2, MW 498, CAS# 54284-45-6, Entry# 285148
2,3-BIS[(TRIMETHYLSILYL)OXY]PROPYL (9Z,12Z)-9,12-OCTADECADIENOATE #

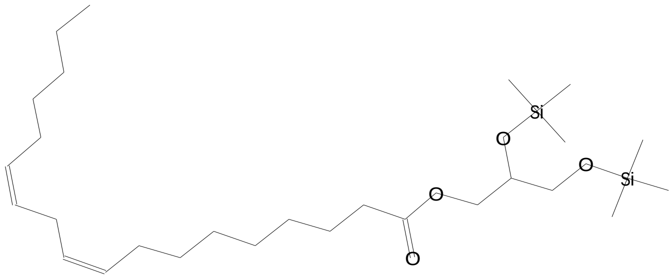

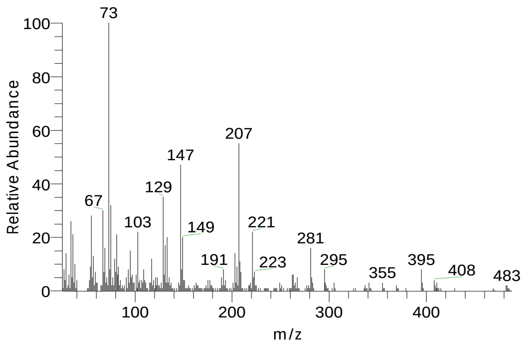

My GC-MS Report

Compound Structure

Hit Spectrum

Formula C27H30O16, MW 610, CAS# 29428-58-8, Entry# 297453
6,8-DI-C-á-GLUCOSYLLUTEOLIN

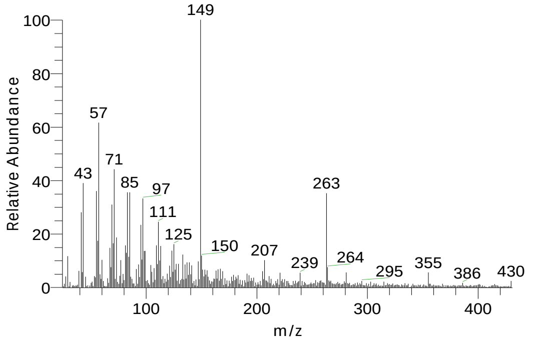

Supplement: Supplementary file 5 — Supplementary Information 5. [file 41598_2023_36121_MOESM5_ESM.pdf]
